# Dispirooxindoles Based on 2-Selenoxo-Imidazolidin-4-Ones: Synthesis, Cytotoxicity and ROS Generation Ability

**DOI:** 10.3390/ijms22052613

**Published:** 2021-03-05

**Authors:** Vladimir K. Novotortsev, Maxim E. Kukushkin, Viktor A. Tafeenko, Dmitry A. Skvortsov, Marina A. Kalinina, Roman V. Timoshenko, Nelly S. Chmelyuk, Liliya A. Vasilyeva, Boris N. Tarasevich, Petr V. Gorelkin, Alexander S. Erofeev, Alexander G. Majouga, Nikolai V. Zyk, Elena K. Beloglazkina

**Affiliations:** 1 Department of Chemistry, M. V. Lomonosov Moscow State University, Leninskie Gory, 1-3, 119991 Moscow, Russia; vladnov9216@rambler.ru (V.K.N.); lemmingg@mail.ru (M.E.K.); tafeenko-victor@yandex.ru (V.A.T.); skvorratd@mail.ru (D.A.S.); boris-tarasevich@yandex.ru (B.N.T.); erofeev@polly.phys.msu.ru (A.S.E.); alexander.majouga@gmail.com (A.G.M.); zyk@org.chem.msu.ru (N.V.Z.); 2 National University of Science and Technology “MISiS”, Leninskiy Prospect, 4, 119049 Moscow, Russia; timoshenkoroma@mail.ru (R.V.T.); nellichmelyuk@yandex.ru (N.S.C.); peter.gorelkin@gmail.com (P.V.G.); 3 Skolkovo Institute of Science and Technology, 4 Alfred Nobel Street, Skolkovo, 143025 Moscow, Russia; Marina.Kalinina@skoltech.ru; 4 Faculty of Bioengineering and Bioinformatics, Lomonosov Moscow State University, Leninskie Gory 1, 119991 Moscow, Russia; liljavasilyeva@gmail.com; 5 D. I. Mendeleev University of Chemical Technology of Russia, Miusskaya sq., 9, 125047 Moscow, Russia

**Keywords:** selenohydantoins, spiro compounds, indolinones, azomethine ylides, cytotoxicity, ROS generation, prooxidant properties

## Abstract

A regio- and diastereoselective synthesis of two types of dispiro derivatives of 2-selenoxoimidazolidin-4-ones, differing in the position of the nitrogen atom in the central pyrrolidine ring of the spiro-fused system—namely, 2-selenoxodispiro[imidazolidine-4,3′-pyrrolidine-2′,3″-indoline]-2″,5-diones (**5a-h**) and 2-senenoxodispiro[imidazolidine-4,3′-pyrrolidine-4′,3″-indoline]-2″,5-diones (**6a-m**)—were developed based on a 1,3-dipolar cycloaddition of azomethine ylides generated from isatin and sarcosine or formaldehyde and sarcosine to 5-arylidene or 5-indolidene-2-selenoxo-tetrahydro-4H-imidazole-4-ones. Selenium-containing dispiro indolinones generally exhibit cytotoxic activity near to the activity of the corresponding oxygen and sulfur-containing derivatives. Compounds **5b**, **5c**, and **5e** demonstrated considerable in vitro cytotoxicity in the 3-(4,5-dimethylthiazol-2-yl)2,5-diphenyl tetrazolium bromide (MTT) test (concentration of compounds that caused 50% death of cells (CC_50_) 7.6–8.7 μM) against the A549 cancer cell line with the VA13/A549 selectivity index 5.2–6.9; some compounds (**5** and **6**) increased the level of intracellular reactive oxygen species (ROS) in the experiment on A549 and PC3 cells using platinized carbon nanoelectrode. The tests for p53 activation for compounds **5** and **6** on the transcriptional reporter suggest that the investigated compounds can only have an indirect p53-dependent mechanism of action. For the compounds **5b**, **6b**, and **6l**, the ROS generation may be one of the significant mechanisms of their cytotoxic action.

## 1. Introduction

Hydantoin and thiohydantoin derivatives containing the spiro-linked indolinone fragments showed significant cytotoxic activity in the in vitro experiments [1,2,3]. The most potent dispiro-thiohydantoins from this series exhibited an IC_50_ value in the range of 4.88–10.46 μM against the well-differentiated hepatocellular carcinoma perpetual cell line (HepG2), human embryonic kidney 293 cells (Hek293), breast cancer cell line (MCF-7), human cervical cancer cells (SiHa), and human colon cancer cells (HCT116); for their oxo-analogs (dispiro-hydantoins), the IC_50_ values were in the range of 6.6–24.1 μM against the HEK293, MCF7, and human lung cancer (A549) cell lines. Based on the data presented in [1,2,4], this activity may be associated with the ability of the dispiro-imidazolone-oxindoles to affect the p53–MDM2 protein–protein interaction [5,6,7,8,9,10,11,12], as well as nonsteroidal antiandrogen properties [13,14,15].

Taking into account that the variation of the exocyclic chalcogen substituent (oxygen or sulfur) of the imidazolone fragment of dispiroimidazolones in the works cited above [1,2,4] did not lead to a significant change in cytotoxicity, and considering the fact that selenium-containing compounds are an attractive class of compounds in search for new cytotoxic anticancer agents [16,17,18,19], in this work, we present a further structural modification of spiro-linked 2-halcogen-imidazolone derivatives, introducing into their molecular structure C=Se groups. It may be noted that some organoselenium compounds are currently evaluated in clinics as anticancer drugs, including L-selenomethionine and Se-methylselenocysteine, by the National Cancer Institute [20,21]; others [22,23,24,25,26,27,28,29] are being evaluated in preclinical studies and are showing relevant antiproliferative and proapoptotic activity in micromolar doses against a number of cancer types (MCF-T [20], FaDu and A253 [21], some human melanoma cell lines [22], SW480 and RKO colon cancer during in vitro and in vivo investigations [24], MCF-7 (breast carcinoma), HT-29 (colon carcinoma) and PC-3 (prostate carcinoma) cells [25], and orthotopic breast cancer model MDA-231-M2 in vitro and in vivo [28]), including hormone-dependent cancer cell lines LNCaP and MCF-7 [20]. Note, also, that the selenium-containing derivative S,S-1,4- phenylenebis(1,2-ethanediyl)bis-isoselenourea (PBISe) has been shown to be a more effective therapeutic agent than its sulfur analog S,S-1,4-phenylenebis(1,2-ethanediyl)bis-isothiourea (PBIT) during in vitro and in vivo studies. PBISe killed melanoma cells >10-fold more effectively than PBIT, and cultured cancer cells were two- to five-fold more sensitive than normal cells [30].

Selenium-containing organic compounds may also enhance the chemotherapy/radiotherapy efficacy [30,31,32]. Therefore, a highly effective modulation of the therapeutic efficacy and selectivity of anticancer drugs Taxol and Cisplatin in nude mice bearing human tumor xenografts of colon carcinoma (HCT-8 and HT-29) and squamous cell carcinoma of the head and neck (FaDu and A253) using 5-methylselenocysteine or Se-methyl-l-selenocysteine was demonstrated [31]. Ethaselen (1,2-(bis[1,2-benzisoselenazolone-3(2H)-ketone])ethane; BBSKE), a selenium-containing thioredoxin reductase inhibitor, was found to suppress irradiation-induced NF-kB activation when using A549 cells stably transfected with a NF-kB luciferase reporter [32]; it also showed a radiosensitizing effect in NSCLC cell lines and tumor models. BBSKE enhanced the efficacy of radiation therapy both in vivo and in vitro without observable toxicity. It has been shown in experiments on rats bearing advanced Ward colorectal carcinoma and in nude mice bearing human squamous cell carcinoma of the head and neck, FaDu, and A253 xenografts that Se-methylselenocysteine offers selective protection against organ-specific toxicity induced by clinically active agents and enhances further the antitumor activity, resulting in an improved therapeutic index, which allows this selenium compound to be considered as selective modulator of the antitumor activity and selectivity of anticancer drugs [21]. 

Moreover, Se-containing compounds were shown to effectively block some angiogenic factors, thereby suppressing cancer-associated angiogenesis [33,34,35]. Se-methylselenocysteine led to a significant reduction of intra-tumoral microvessel density in HUVEC mammary carcinomas [33] and FaDu cell lines [34] in the experiments in vitro and in vivo; methyl Se-containing intermediates, such as methylselenol, demonstrated apoptogenic activities without genotoxicity and inhibitory activities for angiogenic switching in the experiments with the HUVEC, MDA-MB-468, MCF-7, and DU145 cell lines [35]. The antiangiogenic attributes reported in this study and the growth arrest make the methylselenol precursors attractive chemopreventive agents for considerations in humans. Selenium derivatives have also been demonstrated to inhibit crucial signaling pathways and proteins deeply implicated in cancer grow and progression; these include calcium-insensitive nitric oxide synthase and the nitrogen-activated protein kinase [31], Akt3 kinase, and histone deacetylases [32], as well as melanin biosynthesis by melanocytes [34].

For a long time, Se-containing compounds were mainly tested in relation to protecting healthy cells from reactive oxygen species (ROS)-induced oxidative damage. However, recent studies suggest that Se seems to be a potential redox modulator with a dual role in oxidative stress, acting as a pro-oxidant or antioxidant, depending on the incorporation type of the organoselenium compound, as well as the dose [17,29].

Based on this data, it can be assumed that the combination of the spiro-linked indolinone and imidazolone with an organoselenium moiety in a single molecule could favorably affect the antitumor activity of synthesized compounds. 

Recently, we synthesized and evaluated a series of novel 2-thioxo-4H-dispiro[imidazolidine-4,3-pyrrolidine-2,3-indole]-2,5(1H)-diones, which have been identified as potent anticancer compounds [1,2,4]. Several substances showed a prominent anticancer activity against the A549, MCF-7, HepG2, SiHa, and HCT116 cell lines associated predominantly with their effect on the p53–MDM2 interaction. Therefore, one of the key points of the presented paper was the evaluation of two series of the Se-contained analogs of thiohydantoins described previously to assess their anticancer potency compared to the parent S-containing molecules. A preliminary study of the cytotoxicity, p53 activation ability, and pro/antioxidant properties of the obtained compounds was undertaken.

It should be especially noted that even the well-established methods of sulfur-containing compounds cannot be always used directly for the synthesis of their selenium-containing analogs due to the frequent problems of the precursor synthesis and the lower stability of selenium-containing derivatives compared to sulfur-containing ones. Thus, herein, we report a convenient synthetic method for the preparation of dispiro derivatives of selenoindolinones.

**Figure 1:**
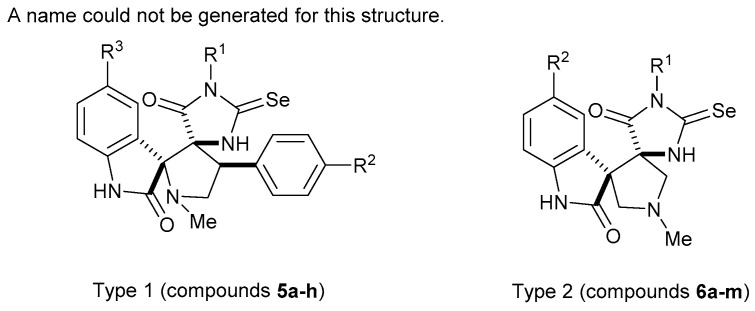
Structural types of the synthesized dispiroselenohydantoins.

The structural types of the synthesized compounds are shown in Figure 1. The synthesis of the type 1 compounds are firstly described here; compounds of type 2 were recently presented by us in a short communication of a single example (R^1^ = 4-EtO-C_6_H_4_) [36].

## 2. Results and Discussion

### 2.1. Synthesis

**Scheme 1:**
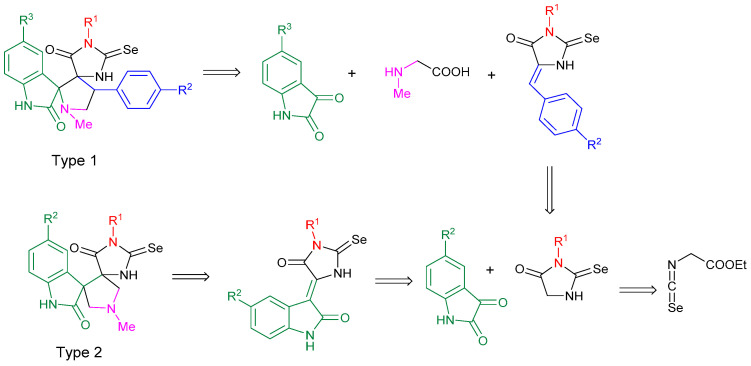
The retrosynthetic scheme for the type 1 and type 2 dispiroselenohydantoins.

A retrosynthetic scheme for the target compounds’ preparation is shown in Scheme 1.

**Scheme 2:**
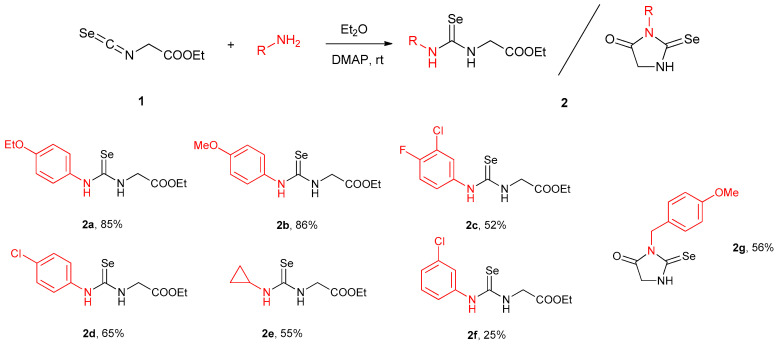
Structures and yields of the reaction products of ethyl 2-isoselenocyanatoacetate **1** with amines.

The starting compounds for the synthesis of both types of spiroselenohydantoins was ethyl 2-isoselenocyanatoacetate **1** synthesized according to the modified literature procedure [37]. Compound **1** then reacted with commercially available secondary aryl or alkyl amines (substituted anilines, cyclopropylamine, and 4-methoxybenzylamine; Scheme 2). A catalytic amount (1 mol%) of 4-dimethylaminopyridine was added to the reaction mixtures; in its absence, the reaction proceeded with a low yield and the formation of significant amounts of byproducts. Diethyl ether was used as a solvent; the reaction products were practically insoluble in this solvent and crystallize from the reaction mixtures. The reaction products in all cases were selenoureas **2a-f**, with the exception of the reaction with 4-methoxybenzylamine, the interaction of which with isoselenocyanate yielded selenohydantoin **2g**.

**Scheme 3:**
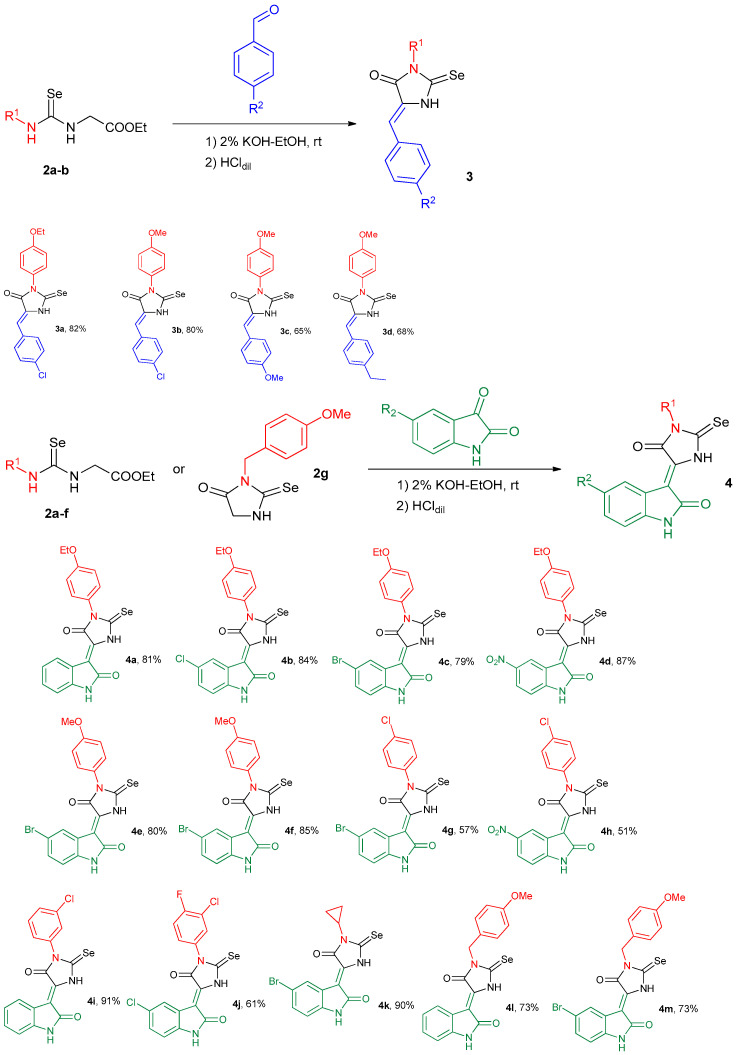
Synthesis of 5-arylidene and 5-indolinylidene-substituted selenohydantoins **3a–d** and **4a–m**.

The obtained selenium derivatives **2a-g** were further introduced into the Knoevenagel condensation with substituted benzaldehydes (to obtain 5-arylidene-2-selenoxohydantoins **3a-d**) or isatins (to obtain 5-indolinylidene-2-selenoxohydantoins **4a-m**) under KOH catalysis (Scheme 3).

Arylidene-substituted selenohydantoins **3a-d** and 5-indoliden-2-selenohydantoins **4a-m** were obtained according to a modified procedure described for sulfur analogs [38,39,40] optimized for maximum yield of target compounds. The conditions for the synthesis of arylidene and indolinylidene-substituted selenohydantoins **3a-d** and **4a-m** were similar, except for the reaction time: during the compounds **3a-d** preparations, the reactions were carried out for three h; for the preparation of compounds **4a-m**, one h was enough. The yields of compounds **3a-d** and **4a-m** are given in Scheme 3.

All the derivatives **3a-d** and **4a-m** were obtained as single geometric isomers with a Z-configuration of the C=C double bonds. The Z-configuration was attributed to products **3a-g** based on the chemical shifts of the vinylic protons (6.8–6.7 ppm) in their ^1^H NMR spectra [41]. For compounds **4a-m**, the ^1^H and ^13^C NMR spectra contain a single set of signals; the Z-configuration was assigned to products based on the literature analogies [4]. The Z configuration of compound **4b** was also indirectly confirmed by the X-ray diffraction data for the product of its reaction with azomethine ylide (compound **6b** [36]). 

**Scheme 4:**
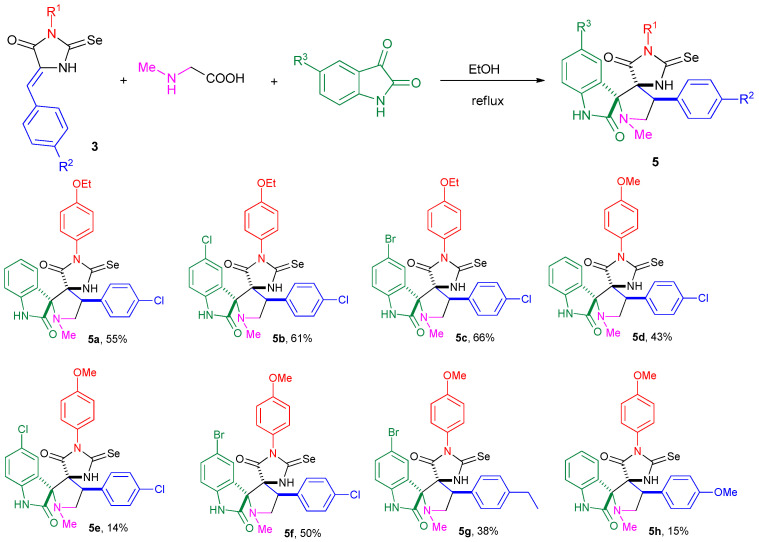
Synthesis of dispiroselenohydantoins **5a-h**.

**Scheme 5:**
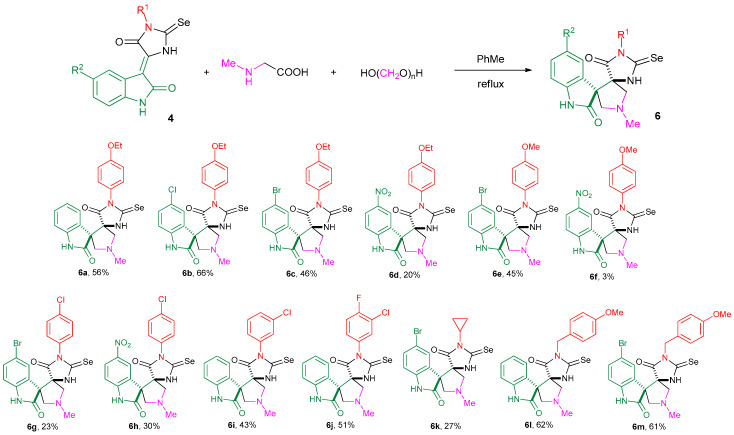
Synthesis of dispiroselenohydantoins **6**.

For the synthesis of the target dispiro-selenohydantoine-indolinones **5a-h** and **6a-m**, the interaction of azomethine ylides, generated from isatins and sarcosine (Scheme 4) or from formaldehyde and sarcosine (Scheme 5), with dipolarophiles **3a-d** and **4a-m** were used. These reactions provided the desired compounds **5a-h** and **6a-m** in a moderate-to-high yield.

The reactions of compounds **3a-d** with sarcosine and isatins (Scheme 4) were carried out in boiling ethanol; in most cases, they gave reasonable yields of the target compounds (14–66%). The products precipitated from the reaction mixture; after which, they were filtered and purified by flash chromatography. The conversion proceeded with good regio- and diastereoselectivity, giving only diastereomeric products with the relative (2′R*,5S*,4′S*) configuration. The formation of the only diastereomers of compounds **5a-h** was confirmed by the presence of a single set of signals in their ^1^H and ^13^C NMR spectra. For all the compound **5a-h** characteristics, there were three pseudo-triplets in their ^1^H NMR spectra at 4.60–3.40 ppm with J~9 Hz, corresponding to three hydrogen atoms of the central pyrrolidine cycle. We did not observe signals of any minor diastereomers in the NMR spectra of the reaction mixtures.

**Figure 2:**
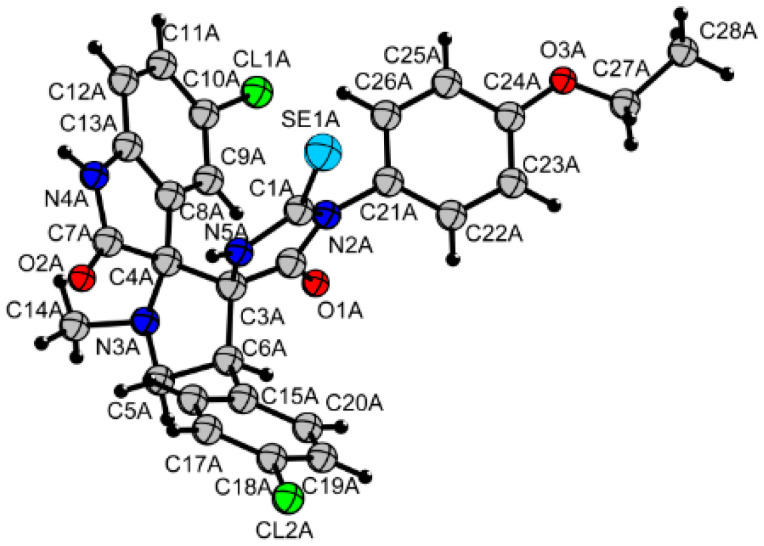
Molecular structure of compound **5b** (CCDC 1999058; one crystallographically independent molecule is shown; the designations of the second independent molecule in the Supplementary Information are similar (NN_B)).

The relative configuration of the stereo centers for compound **5b** was also proven using single-crystal X-ray diffraction data. The molecular structure of **5b** is shown in Figure 2. The neighboring spiro-conjugated cycles of the molecule are almost planar and near perpendicular to each other. A more detailed description of the X-ray data is provided in the Supplementary Information.

**Scheme 6:**
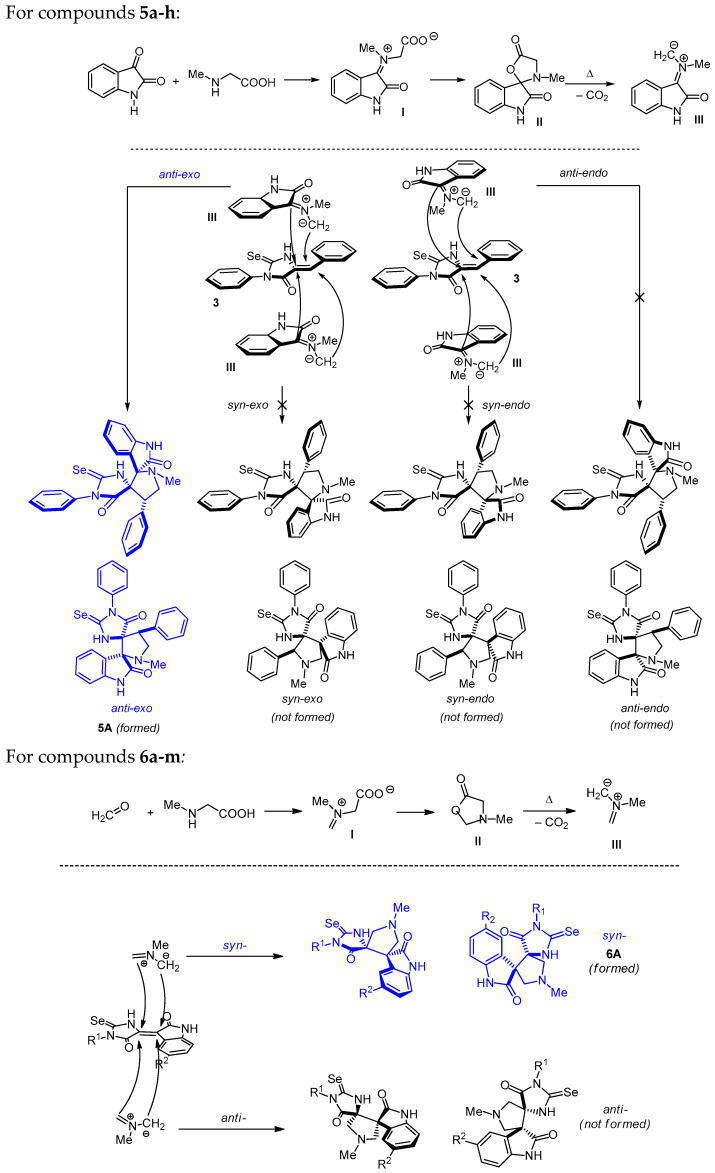
The supposed mechanism of the formation of dispiro derivatives **5a-h** and **6a-m**.

The mechanism of the spirocyclization reactions is presumably similar to that for the addition of azomethine ylides to N-substituted 5-arylidenethiohydantoins [4]. It involves the successive reactions of isatin with sarcosine to form an iminium intermediate **I** and further a lactone **II**, which loses the CO_2_ molecule to afford dipole **III** (Scheme 6). This dipole then regio- and diastereoselectively attacks the C=C double bond of 5-arylidenehydantoin **3**.

Note that the attack of dipole can proceed from both above and below the double-bond plane to result in a mixture of enantiomers. Possible ways of the azomethine ylide approach to the dipolarophile moiety are shown in Scheme 6. Based on the structure of the products, we can conclude that a cycloaddition reaction takes place only through the exo transition state, apparently due to an increase in the transition state energy as a result of electrostatic repulsion in the case of the cis-located carbonyl groups of azomethine.

Compounds **4a-m** were reacted with azomethine ylide formed by an interaction of sarcosine and paraform; as a result, dispiro derivatives **6a-m** were obtained (Scheme 5). The optimal reaction procedure turned the reagents co-boiling in toluene for six h; in methanol and ethanol, the reaction did not proceed. Sarcosine and paraform were put into the reaction in an amount of eight molar equivalents, since during the optimization of the procedure, it was shown that this leads to the highest product yields. The target compounds **6a-m** were isolated by column chromatography.

Compounds **6a-m** contained two stereo centers, and their formation proceeded diastereoselectively with the formation of the products with the (5R*, 4′R*) configuration. For dispiroindolinones **6**, the ^1^H NMR spectrum was characteristically in the range of 3.50–2.90 ppm, in which there were four doublets with J~10 Hz corresponding to the protons of the pyrrolidine fragments (see Supplementary Information). The (5R*, 4′R*) configuration of compound **6b** was proven by the X-ray diffraction data [36].

The formation of dispiroselenoindolinones **6a-m** apparently took place similarly to compounds **5a-h** through the following steps (Scheme 6): (1) the reaction between isatin and amino acid with the formation of an iminium intermediate, (2) the cyclization of the iminium intermediate, followed by the loss of CO_2_ and the formation of a 1,3-dipole, and (3) the regioselective addition of a 1,3-dipole at the 5-indolidene-selenohydantoin C=C double bond.

Thus, a convenient diastereoselective method for the synthesis of two types of selenohydantoin-dispiroindolinone differing in the position of the N atom in the central pyrrolilidine cycle were proposed based on the regio- and diastereoselective 1,3-dipolar cycloaddition of azomethine ylides to electron-deficient alkene systems based on 2-selenoxo-tetrahydro-4H-imidazole-4-ones. Therefore, we extended the reaction of 5-methylene substituted hydantoins and thiohydantoins spirocyclization to their C=Se-containing analogs by the coupling of 2-selenohydantoins **3a-d** and **4a-m** with azomethine ylides under easy conditions with reasonable yields.

### 2.2. Cytotoxicity and p53 Activation

Some of the obtained compounds were tested for cytotoxicity using the standard 3-(4,5-dimethylthiazol-2-yl)2,5-diphenyl tetrazolium bromide (MTT) test [42]. This study was realized using the cell lines of breast cancer MCF7, human lung carcinoma A549, non-cancer human embryonic kidney cell line HEK293T, and the noncancer lung fibroblast VA13 cell line, along with the results obtained for doxorubicin (Dox) as a known cytotoxic drug used in clinical practice. Lung cancers and breast cancers are the most common causes of tumor lesions and related deaths in the world, according to the WHO data [43]; thus they are of great interest for the search for novel anticancer molecules. A549 and MCF7 were selected for the lung tumor model and breast cancer, because (i) these cell lines are well-studied and often used in cytotoxicity investigations in the literature, and (ii) they have a high proliferation rate typical for cancer cells. HEK293T and VA13 cells both have nontumor etiology and were used as noncancer cells. We used immortalized cell lines, because they are stable and we could compare the data acquired in different experiments through the years. VA13 is a slow-growth cell line; otherwise, HEK293T has a growth rate compatible with A549 cells. 

This small set of four cell lines allowed us to make a preliminary suggestion about the selectivity against lung and breast cancer cells and if the selective toxicity is due to targeting one of the cell division mechanisms. Additionally, it was selected to compare the obtained data with data from previously investigated O- and S-analogs [1,2,3,4]. The results of these assays are shown in Table 1.

The most cytotoxic compounds **5b**, **5c**, and **5e** demonstrated cytotoxicity with IC_50_ values in the range of 7.6–8.7 µM against cell lines A549 and MCF7 (Table 1), which were significantly lower than the cytotoxicity of doxorubicin. However, a relevant selectivity was observed for the most active compounds among the cell types; therefore, compound **5c** turned out to be seven times more cytotoxic in relation to the cancer cell line A549A compared to the noncancer cell line VA13 (for doxorubicin, this ratio was eight). Comparing the obtained data with the previously obtained results for dispirohydantoins and thiohydantoins of a similar structure [1,2,4], it can be noted that the selenium-containing analogs generally exhibited cytotoxic activity near to the activity of the corresponding oxygen and sulfur-containing derivatives.

We measured cytotoxicity of leader compounds using fluorescent cells based cyto-toxicity assay [1], as well as cytotoxicity of some compounds with high, medium and no selectivity indexes in MTT assay by CalceinAM staining for key cell lines. Protocols of these studies and the measured cytotoxicities data are given in the Supplementary Information (Tables S1 and S2). The results obtained when measuring the cytotoxicity by alternative methods are generally consistent with the results of the MTT test.

The synthesized compounds were also tested for the activation of p53 in a transcriptional reporter activation test [44] in an effort to elucidate the possible mechanism of cytotoxic action of dispiroselenohydantoins and compare them with the previously investigated structurally similar hydantoins and thiohydantoins [1,2,4]. The results are presented in Table 2. Nutlin-3a, a known inhibitor of p53–MDM2 protein–protein interactions, was used as a positive control. We proceeded from the assumption that the mechanisms of the cytotoxicity of the studied compounds may include p53 activation. It is known that activated p53 induces the transcription of MDM2, which can directly interact with the transactivation domain of p53, thereby inhibiting its transcription activity by targeting it for polyubiquitination and further proteasome-mediated degradation [45]. In many cancer cells lines, including MCF7 and A549, the overexpression of MDM2 is actually observed, resulting in significant apoptosis attenuation [1]. Nutlin-3a causes a direct activation of p53 via the block of p53–MDM2 interactions and elevates the p53 activity up to 5–10 times in the reporter assay [46]. Four dilutions of compounds **5** and **6** (100 to 1.56 μM) were tested, and only for compound **6l** in a high concentration (>100 µM), the treatment of the cells led to a two-fold p53 activation; it was substantially less than for Nutlin-3a but comparable with indirect cisplatin action, which suggests that the investigated compounds can only have an indirect p53-dependent mechanism of action [47]. Consequently, despite the moderate cytotoxicity of some studied selenoimidazolones, their main toxic effect on the cell was not due to p53 protein activation.

### 2.3. ROS Generation Ability

ROS-mediated nuclease activity [47], mitochondrial membrane depolarization [48], and apoptosis induction [49] are all well-described cytotoxic mechanisms that are generally the consequences of reactive oxygen species formation.

**Figure 3:**
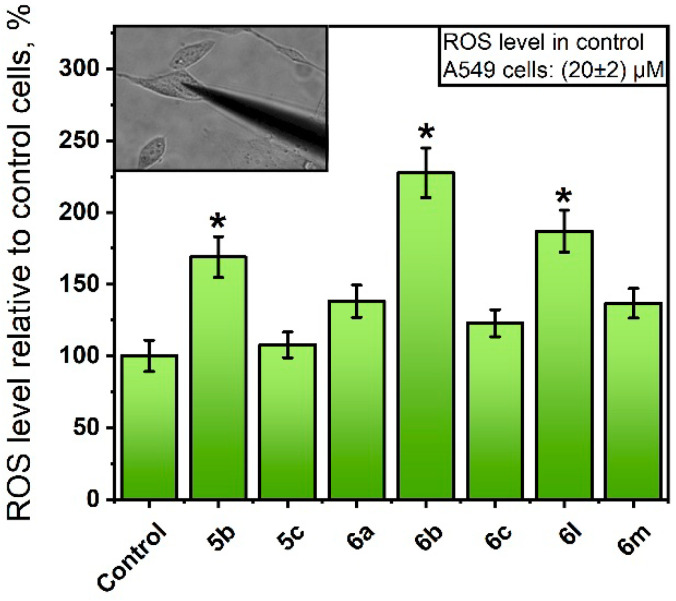
Comparison of the reactive oxygen species (ROS) level inside the A549 cells incubated with compounds **5** and **6**; *, *p* < 0.05 (one-way ANOVA).

We investigated the ROS production ability of the compounds **5b** and **5c** and **6a-c**, **6l**, and **6m**, which were tested for the activation of p53. To detect the ROS production in A549 tumor cells, we used an amperometric nanosensor system previously described in [50,51,52] that allowed us to measure the ROS level in single cells using 60–100-nm diameter Pt nanoelectrodes for the amperometric detection of hydrogen peroxide. This method is low-invasive and more accurate in comparison with other methods of ROS determination [51]. The setup allows manipulating the nanoelectrode and precisely penetrating it into a single cell with real-time recording of the differences of the current values between the solution and cell (Figure 3).

As seen from the obtained data (Figure 3), all the studied compounds, to one degree or another, turned out to be prooxidants that increased the ROS levels in the tested cells. Compounds **6b** and **6c** showed a higher level of ROS generation compared to the other investigated compounds, increasing the ROS intracellular level by about two times. There was no correlation between the activation of p53 and the ability to generate reactive oxygen species; compounds **6b** and **6c** showed the highest ROS-generating abilities but were not the best p53 activators.

Thus, at least for compounds **5c, 6a**, **6c**, and **6m**, ROS production cannot be accepted as the main mechanism of their cytotoxic actions, and this issue requires further study. At the same time, for compounds **5b**, **6b**, and **6l**, ROS generation may be one of the significant mechanisms of their cytotoxic actions.

We also investigated three representatives of each compounds **5a-h** and **6a-m** (with the highest. medium and lowest cytotoxicity) for their ability to detect ROS formation in PC3 tumor cells. These results are presented in the Supplementary Information (Figure S1); the least cytotoxic compounds **6l** showed the weakest ability of ROS generation. 

## 3. Materials and Methods 

### 3.1. General

All starting compounds were commercially available reagents and were purchased from Sigma-Aldrich. All used solvents were anhydrous and were purified according to the procedures described in [53]. 

^1^H and ^13^C NMR spectra were recorded on a Bruker Avance instrument with an operating frequency of 400 MHz for ^1^H NMR and 101 MHz for ^13^C NMR. Chemical shifts were given in parts per million on a scale of δ relative to hexamethyldisiloxane as an internal standard.

The IR spectra were measured on a TermoNicolet IR 200 Fourier spectromete (Termo Scientific, Waltham, MA, USA) (resolution 4 cm^–1^).

High-resolution mass spectra were recorded on an Orbitrap Elite mass spectrometer (Thermo Scientific). To enter solutions with a concentration of 0.1–9 μg/mL (in 1% formic acid in acetonitrile), direct injection into the ion source using a syringe pump (5 μL/min) was used. Spray voltage ± 3.5 kV, and capillary temperature 275 °C.

X-ray diffraction studies were performed on a Syntex P21 diffractometer (SYNTEC Incorcoration, HsinChu, China) at 293K (graphite monochromator, λ (MoKα) = 0.71073 Å, ω-scanning). The absorption was taken into account by measuring the intensities of the equivalent reflections (T_min_/T_max_). The structures were solved by the direct method (SHELXS-97) and refined in full-matrix anisotropic least squares by F2 for all nonhydrogen atoms (SHELXL-97). All hydrogen atoms were objectively localized and refined in the isotropic approximation.

#### 3.1.1. Cell Lines

The human breast cancer cell line MCF7 and human lung adenocarcinoma cell line A549 were kindly provided by Dr. S. Dmitriev, the immortalized human fibroblasts cell line VA13 was kindly provided by Dr. M. Rubtsova, and the human embryonic kidney HEK293T cell line was kindly provided by Dr. E. Knyazhanskaya. The MCF7, VA13, A549, and HEK293T cell lines were maintained in DMEM/F-12 (Thermo Fisher Scientific) culture medium containing 10% fetal bovine serum (Thermo Fisher Scientific), 50-µg/mL penicillin, and 0.05-mg/mL streptomycin at 37 °C (Thermo Fisher Scientific, USA) in 5% CO_2_. Medium F-12 (Paneco LLC, Moscow, Russia) containing 10% fetal bovine serum (Thermo Fisher Scientific, USA), 50-µg/mL penicillin, and 0.05-mg/mL streptomycin (Thermo Fisher Scientific, USA) was used instead of DMEM/F-12 in some cytotoxicity assays. The PC-3 cell line (ATCC) was cultured in RPMI-1640 medium supplemented with 10% fetal bovine serum (FBS) and 2-mM L-glutamine (Gibco, Carlsbad, CA, USA). Cells were maintained at 37 °C in a humidified incubator MCO-18AC (Sanyo, Osaka, Japan) supplied with 5% CO_2_. After attaining 80% confluence, the cells were harvested with TrypLE (Gibco) and sub-cultured 1:8. Cell cultures were tested for the absence of mycoplasma.

#### 3.1.2. In Vitro Survival Assay (MTT Assay)

The cytotoxicity of the substances was tested using the MTT (3-(4,5-dimethylthiazol-2-yl)2,5-diphenyl tetrazolium bromide) assay [42] with some modifications. Two thousand five hundred cells per well for the MCF7, HEK293T, and A549 cell lines or 4000 cells per well for the VA-13 cell line were plated out in 135 µL of DMEM-F12 media (Gibco) in 96-well plates and incubated in a 5% CO_2_ incubator for the first 16 h without treating. Then, 15 µL of DMSO media solutions of the tested substances was added to the cells (final DMSO concentrations in the media were 1% or less), and cells were treated 72 h with 50 nM–100 µM (eight dilutions) of our substances (triplicate each) and doxorubicin for the control substance. The MTT reagent (Paneco LLC) was then added to cells up to a final concentration of 0.5 g/L (10x stock solution in PBS was used) and incubated for 2 h at 37 °C in the incubator under an atmosphere of 5% CO_2_. The MTT solution was then discarded, and 140 µL of DMSO (PharmaMed LLC) was added. The plates were swayed on a shaker (60 rpm) to dissolve the formazan. The absorbance was measured using a microplate reader (VICTOR X5 Light Plate Reader, PerkinElmer, Waltham, MA, USA) at a wavelength of 565 nm (in order to measure the formazan concentration). The results were used to construct a dose–response graph and to estimate the CC_50_ value (concentration of compounds that caused 50% death of cells) with GraphPad Software [54]. The selectivity index was calculated according to [55,56].

#### 3.1.3. Transcriptional Assay for P53 Activation (ONPG-Test)

For testing, we used human adenocarcinoma cells A549 with a recombinant lentiviral insert LC5 (line A549/LC5 was kindly provided by Professor P. M. Chumakov). The b-galactosidase reporter construction equipped with the p53 promotor frame [47] was used to assess the p53 expression level in the p53wt A549 cell line. The compounds were tested in the concentration range of 1.5–100 µM with four times the dilution steps. The incubation time was 20 h. To take into account the toxic effect of the molecules, the output signal was normalized considering the quantity of the cells estimated by the MTT test with the same incubation tome (20 h) in a parallel assay. The output was significant if the background signal was exceeded two or more times. Cells were cultured at 37 °C and 5% CO_2_ in DMEM/F-12 medium supplemented with antibiotic serum and L-glutamine.

#### 3.1.4. Intracellular ROS Detection by Pt Nanoelectrodes

Commercially available disk-shaped carbon nanoelectrodes isolated in quartz (ICAPPIC Limited, London, UK) with diameters 60–100 nm were used to prepare Pt nanoelectrodes for the amperometric detection of ROS. Before the deposition of platinum on the carbon surface, the disk-shaped carbon was etched in a 0.1-M NaOH, 10-mM KCl solution during 40 cycles of 10 seconds (from 0 to +2200 mV) to create nanocavities (Figure S2). Further electrochemical deposition of platinum in nanocavities was achieved by cycling from 0 to 800 mV with a scan rate of 200 mV s^−1^ for 4 to 5 cycles in 2-mM H_2_PtCl_6_ solution in 0.1-M hydrochloric acid (Figure S3). Cyclic voltammetry in a 1-mM solution of ferrocene in methanol in PBS was used to control the electrode surface at all stages of fabrication [50,53]. A549 (3 × 10^5^) cells were seeded in 35-mm Petri dishes and incubated under the conditions described above, then treated 24 h. The complexes were dissolved in DMSO and diluted in culture medium. The final concentration of the compounds in the culture medium were IC_50_ with 1 h of incubation time. Untreated cells were used as a control, which was performed at the beginning and at the end of the experiment. Attached cells in Petri dishes were washed three times by using Hanks’ Balanced Salt solution to remove the media and traces of complexes. On average, about 10 cells were measured by 2–4 Pt electrodes in 2 independent Petri dishes for each complex. Prior to the measurements, each platinum electrode was calibrated using a series of standard H_2_O_2_ solutions. Levels of ROS in the cells were determined based on the calibration curve (Figure S4).

**Figure 4:**
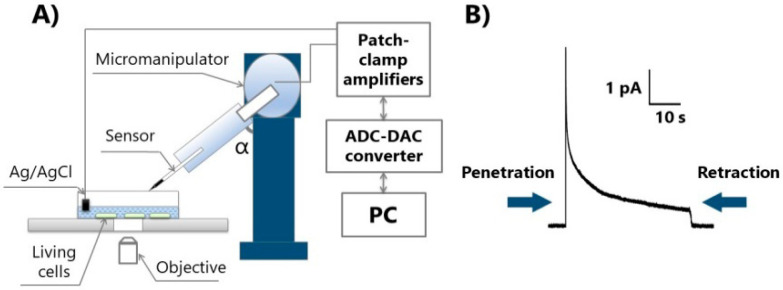
In vitro ROS measurements. (**A**) Scheme showing the setup for intracellular measurements. (**B**) ROS intracellular measurements. Recording a difference of the current value between the solution and a single cell.

The setup for amperometric measurements (Figure 4) included a PC that was connected to a system consisting of an ADC-DAC converter Axon Digidata 1550B (Axon Instruments, Burlingame, CA, USA) and patch-clamp amplifier MultiClamp 700B (Axon Instruments, USA). The working head of the amplifier was fixed on a PatchStar Micromanipulator (Scientifica, London, UK), which placed near an inverted optical microscope Nikon Eclipse TI-U (Nikon, Tokyo, Japan). A Pt nanoelectrode was fixed by a special holder on the working head of the amplifier. Thus, it was possible to change the position of the electrode with a micromanipulator and carry out an additional assessment in the optical microscope. A reference electrode (Ag/AgCl) was immersed in the buffer solutions (3-M KCl). The potential difference between the Pt nanoelectrode and the reference electrode was recorded with the pClamp 11 software suite (Molecular Devices, Silicon Valley, CA, USA) and processed with Origin 2018 software [57].

Statistical analyses were conducted using the ANOVA test. All plots show the mean values ± SE. All tests assumed a normal distribution, and the statistical significance threshold was set at *p* < 0.05.

### 3.2. Synthesis

#### 3.2.1. Glycine Ethyl Ester Hydrochloride

In a three-necked flask equipped with a magnetic stirrer, reflux condenser, and dropping funnel was 150 mL (2.50 mol) of ethanol cooled to −15 °C. With vigorous stirring, 25 mL (0.35 mol) of SOCl_2_ were added dropwise. Then, 25 g (0.33 mol) of glycine were added over 25 min. After this, the cooling was removed, the mixture was brought to a boil and boiled for 1 h. The hot solution was filtered and cooled to 0 °C. The formed precipitate was filtered off and washed with diethyl ether (2 × 50 mL), then dried to a constant weight over P_2_O_5_ and recrystallized from ethanol-ether mixture (1:1). The reaction yielded 22.5 g (48%) of glycine ethyl ester hydrochloride as white crystals. ^1^H NMR (400 MHz, DMSO-d_6_, δ, ppm): 8.59 (s, 3H, NH_3_^+^). 4.18 (q, J = 7.1 Hz, 2H, C(O)OCH_2_CH_3_), 3.74 (s, 2H, CH_2_), 1.22 (t, J = 7.1 Hz, 3H, C(O)OCH_2_CH_3_).

#### 3.2.2. N-formylglycine Ethyl Ester

To 90 mL of ethyl formate, 25 g (179.0 mmol) of glycine ethyl ester hydrochloride and 0.02 g (0.10 mmol) of p-TsOH were added and heated to boiling. Then, 25 mL (179.0 mmol) of Et_3_N were added, and the resulting solution was boiled within 20 h. Then, the reaction mixture was cooled to room temperature. The precipitate was filtered off, and the filtrate was evaporated. The filtrate was cooled to −15 °C. The formed precipitate was filtered off again. The filtrate was distilled under vacuum. The reaction gave 18.18 g (77%) of N-formylglycine ethyl ester as a clear colorless oil (110 °C/0.1 mm Hg). ^1^H NMR (400 MHz, CDCl_3_, δ, ppm): 8.22 (s, 1H, CHO), 6.62 (bs, 1H, NH). 4.20 (q, J = 7.2 Hz, 2H, C(O)OCH_2_CH_3_), 4.05 (d, J = 5.4 Hz, 2H, CH_2_), 1.26 (t, J = 7.2 Hz, 3H, C(O)OCH_2_CH_3_).

#### 3.2.3. Ethyl 2-Isocyanoacetate

To a solution of 14.57 g (111.1 mmol) of N-formylglycine ethyl ester and 38.5 mL (275.5 mmol) of Et_3_N in 115 mL of CH_2_Cl_2_, 10.2 mL (111.1 mmol) of POCl_3_ were added dropwise at 0 °C, and the reaction mixture was stirred for 1 h at 0 °C, then at 20–25 °C, and with vigorous stirring, a solution of 30 g of K_2_CO_3_ in 90 mL of H_2_O was slowly added. After which, the reaction mixture was stirred for 30 min at room temperature. The organic phase was then separated, and the aqueous phase was diluted to 225 mL and extracted with CH_2_Cl_2_ (2 × 55 mL). After washing all three organic phases with brine, they were combined and dried over Na_2_SO_4_, evaporated, and the residue was distilled under vacuum. The reaction gave 9.86 g (78%) of ethyl 2-isocyanoacetate as a clear yellowish oil (80–82 °C/12 mmHg). ^1^H NMR (400 MHz, DMSO-d_6_, δ, ppm): 4.64 (s, 2H, CH_2_), 4.18 (q, J = 7.2 Hz, 2H, C(O)OCH_2_CH_3_), 1.22 (t, J = 7.1 Hz, 3H, C(O)OCH_2_CH_3_).

#### 3.2.4. Ethyl Isoselenocyanatoacetate (1)

8.5 g (75.1 mmol) of ethyl 2-isocyanoacetate were added to 240 mL of tetrahydrofuran in an argon atmosphere. Thirteen point two milliliters (94.8 mmol) of Et_3_N and 6.37 g (80.6 mmol) of selenium powder were added then, and the reaction mixture was boiled for 6 h, then passed through celite, evaporated, and 120 mL of EtOAc were added. The resulting solution was washed with water (2 × 70 mL) and dried over Na_2_SO_4_. The product was isolated by column chromatography in a EtOAc:petroleum ether = 1:9 (*v*/*v*) system. As a result of the reaction. 7.64 g (53%) of compound **1** were obtained as a clear red liquid. ^1^H NMR (400 MHz, CDCl_3_, δ, ppm): 4.35 (s, 2H, CH_2_), 4.28 (q, J = 7.2 Hz, 2H, C(O)OCH_2_CH_3_), 1.32 (t, J = 7.2 Hz, 3H, C(O)OCH_2_CH_3_).

#### 3.2.5. Selenoureas and 2-Selenoxohydantoins **2a-g** (General Procedure)

Ethyl 2-isoselenocyanatoacetate **1** (1 eq.) was dissolved in diethyl ether. Then, amine (1 eq.) was added in portions to the solution. After which, 1 mol% of DMAP was added. The reaction mixture was stirred for 6 h. In the case of precipitation, the precipitate was filtered off and washed with diethyl ether and, if necessary, purified by column chromatography in MeOH:CHCl_3_ = 1:50 (*v*/*v*). If a homogeneous solution formed, it was evaporated, and the product was isolated by column chromatography and yielded 52–86%.

N-(4-ethoxyphenyl)-N′-carbethoxymethylselenourea (**2a**)

^1^H NMR (400 MHz, CDCl_3_, δ, ppm): 8.21 (s, 1H, NH), 7.19 (d, J = 8.8 Hz, 2H,Ar), 6.95 (d, J = 8.8 Hz, 2H,Ar), 6.68 (s, 1H, NH), 4.50 (d, J = 4.7 Hz, 2H, CH_2_), 4.21 (q, J = 7.2 Hz, 2H, C(O)OCH_2_CH_3_), 4.04 (q, J = 7.0 Hz, 2H, ArOCH_2_CH_3_), 1.43 (t, J = 7.0 Hz, 3H, ArOCH_2_CH_3_), 1.28 (t, J = 7.2 Hz, 3H, C(O)OCH_2_CH_3_). IR (pellet KBr): *ν*_max_ 3254, 3073, 3042, 3000, 2980, 2929, 2881, 1712, 1609, 1588, 1545, 1516, 1471, 1393, 1367, 1338, 1312, 1298, 1283, 1255, 1234, 1195, 1173, 1131, 1117, 1045, 1027, 956, 927, 919, 867, 844, 832, 817, 801, 779, 710, 667, 641, 607, 571, 532 cm^−1^.

N-(4-methoxyphenyl)-N′-carbethoxymethylselenourea (**2b**)

^1^H NMR (400 MHz, CDCl_3_, δ, ppm): 8.26 (s, 1H, NH), 7.21 (d, J = 8.8 Hz, 2H, Ar), 6.97 (d, J = 8.9 Hz, 2H, Ar), 6.69 (s, 1H, NH), 4.50 (d, J = 4.7 Hz, 2H, CH_2_), 4.21 (q, J = 7.2 Hz, 2H, C(O)OCH_2_CH_3_), 3.83 (s, 3H, OCH_3_), 1.28 (t, J = 7.2 Hz, 3H, C(O)OCH_2_CH_3_). IR (pellet KBr): *ν*_max_ 3334, 3145, 2976, 2932, 2914, 2836, 1723, 1607, 1549, 1515, 1473, 1444, 1411, 1391, 1369, 1345, 1299, 1251, 1231, 1183, 1171, 1126, 1108, 1028, 966, 938, 865, 843, 831, 792, 744, 680, 643, 634, 591, 548, 529 cm^−1^.

N-(3-chloro-4-fluorophenyl)-N′-carbethoxymethylselenourea (**2c**)

^1^H NMR (400 MHz, CDCl_3_, δ, ppm): 8.41 (s, 1H, NH), 7.39 (dd, J_1_ = 2.2 Hz, J_2_ = 6.3 Hz, 1H, Ar), 7.26–7.19 (m, 2H, Ar) 6.83 (s, 1H, NH), 4.50 (d, J = 4.6 Hz, 2H, CH_2_), 4.24 (q, J = 7.2 Hz, 2H, C(O)OCH_2_CH_3_), 1.30 (t, J = 7.2 Hz, 3H, C(O)OCH_2_CH_3_).

N-(4-chlorophenyl)-N′-carbethoxymethylselenourea (**2d**)

^1^H NMR (400 MHz, CDCl_3_, δ, ppm): 8.44 (s, 1H, NH), 7.44 (d, J = 8.4 Hz, 2H, Ar), 7.26 (d, J = 9.6 Hz, 2H, Ar), 6.95 (s, 1H, NH), 4.50 (d, J = 3.6 Hz, 2H, CH_2_), 4.22 (q, J = 7.0 Hz, 2H, C(O)OCH_2_CH_3_), 1.30 (t, J = 7.2 Hz, 3H, C(O)OCH_2_CH_3_). IR (pellet KBr): *ν*_max_ 3314, 3187, 3148, 3100, 2993, 2975, 2928, 2909, 2852, 2799, 2363, 2160, 1728, 1692, 1594, 1544, 1525, 1490, 1442, 1417, 1383, 1336, 1321, 1281, 1224, 1159, 1117, 1091, 1022, 1013, 974, 967, 936, 879, 856, 814, 720, 698, 689, 653, 620, 571, 547 cm^−1^.

N-cyclopropyl-N′-carbethoxymethylselenourea (**2e**)

^1^H NMR (400 MHz, CDCl_3_, δ, ppm): 7.54 (bs, 1H, NH), 7.35 (bs, 1H, NH), 4.51 (s, 2H, CH_2_), 4.27 (q, J = 7.2 Hz, 2H, C(O)OCH_2_CH_3_), 2.57 (s, 1H,CHPr), 1.33 (t, J = 7.2 Hz, 3H, C(O)OCH_2_CH_3_), 0.96–0.90 (m, 2H, Pr), 0.85–0.77 (m, 2H, Pr).

N-(3-chlorophenyl)-N′-carbethoxymethylselenourea (**2f**)

^1^H NMR (400 MHz, CDCl_3_, δ, ppm): 8.55 (bs, 1H, NH), 7.40 (t, J = 8.6 Hz, 1H,Ar), 7.33 (s, 1H, Ar), 7.33–7.30 (m, 1H, Ar), 7.23 (d, J = 8.3 Hz, 1H, Ar), 7.06 (bs, 1H, NH), 4.51 (s, 2H, CH_2_), 4.23 (q, J = 7.1 Hz, 2H, C(O)OCH_2_CH_3_), 1.30 (t, J = 7.2 Hz, 3H, C(O)OCH_2_CH_3_).

3-(4-methoxybenzyl)-2-selenoxoimidazolidin-4-one (**2g**)

^1^H NMR (400 MHz, CDCl_3_, δ, ppm): 7.78 (bs, 1H, NH), 7.52 (d, J = 8.7 Hz, 2H, Ar), 6.85 (d, J = 8.6 Hz, 2H, Ar), 5.05 (s, 2H, CH_2_), 3.89 (s, 2H, CH_2_), 3.79 (s, 3H, OCH_3_).

#### 3.2.6. 5-Arylidene-2-Selenoxohydantoins **3a-g** (General Procedure)

A 2% solution of KOH in ethanol (1 equivalent of KOH) was added to selenourea (or 2-selenoxohydantoin) **2** (1 equiv.). Then, aldehyde (1 equiv.) was added to the solution. The reaction mixture was stirred for 5 h. A dilute hydrochloric acid solution (H_2_O:HCl conc = 9:1) was added to the reaction mixture. The formed precipitate was filtered off and washed with water and diethyl ether. If no precipitation occurred, the solvent was removed from the reaction mixture, and then, the product was purified by column chromatography in MeOH:CHCl_3_ = 1:50 (*v*/*v*) and yielded 65–82%.

(Z)-3-(4-ethoxyphenyl)-5-(4-chlorobenzylidene)-2-selenoxoimidazolidin-4-one (**3a**)

^1^H NMR (400 MHz, DMSO-d_6_, δ, ppm): 13.12 (s, 1H, NH), 7.89 (d, J = 8.6 Hz, 2H, Ar), 7.52 (d, J = 8.5 Hz, 2H, Ar), 7.27 (d, J = 8.9 Hz, 2H, Ar), 7.03 (d, J = 8.9 Hz, 2H, Ar), 6.79 (s, 1H, vinyl), 4.08 (q, J = 6.9 Hz, 2H, ArOCH_2_CH_3_), 1.36 (t, J = 6.9 Hz, 3H, ArOCH_2_CH_3_).

(Z)-3-(4-methoxyphenyl)-5-(4-chlorobenzylidene)-2-selenoxoimidazolidin-4-one (**3b**)

^1^H NMR (400 MHz, DMSO-d_6_, δ, ppm): 13.13 (s, 1H, NH), 7.89 (d, J = 8.4 Hz, 2H, Ar), 7.52 (d, J = 8.5 Hz, 2H, Ar), 7.30 (d, J = 8.8 Hz, 2H, Ar), 7.05 (d, J = 8.9 Hz, 2H,Ar), 6.80 (s, 1H, vinyl), 3.82 (s, 3H, OCH_3_).

(Z)-3-(4-methoxyphenyl)-5-(4-methoxybenzylidene)-2-selenoxoimidazolidin-4-one (**3c**)

^1^H NMR (400 MHz, DMSO-d_6_, δ, ppm): 7.88 (s, 1H, NH), 7.29–7.16 (m, 4H, Ar), 7.05–6.98 (m, 4H, Ar), 6.75 (s, 1H, vinyl), 3.81 (s, 3H, OCH_3_), 3.80 (s, 3H, OCH_3_).

(Z)-3-(4-methoxyphenyl)-5-(4-ethylbenzylidene)-2-selenoxoimidazolidin-4-one (**3d**)

^1^H NMR (400 MHz, DMSO-d_6_, δ, ppm): 13.21–12.81 (m, 1H, NH), 7.81 (d, J = 8.1 Hz, 2H, Ar), 7.31 (d, J = 7.8 Hz, 2H, Ar), 7.29 (d, J = 8.9 Hz, 2H, Ar), 7.05 (d, J = 9.0 Hz, 2H, Ar), 6.78 (s, 1H, vinyl), 3.81 (s, 1H, OCH_3_), 2.64 (q, J = 7.6 Hz, 2H, CH_2_CH_3_), 1.21 (t, J = 7.6 Hz, 3H, CH_2_CH_3_).

#### 3.2.7. 5-Indolinyliden-2-Selenoxohydantoins **4a-m** (General Procedure)

A 2% solution of KOH in ethanol (1 equivalent of KOH) was added to selenourea (or 2-selenoxohydantoin) **2** (1 equiv.). Isatin (1 equiv.) was added to the solution. The reaction mixture was stirred for 1 h; then, a dilute hydrochloric acid solution (H_2_O:HCl conc = 9:1) was added to the reaction mixture. The formed precipitate was filtered off and washed with water and diethyl ether. If no precipitation occurred, the product is isolated by column chromatography in MeOH:CHCl_3_ = 1:20 (*v*/*v*) and yielded 57–91%.

(Z)-3-(3-(4-ethoxyphenyl)-4-oxo-2-selenoxoimidazolidin-5-ylidene)indolin-2-one (**4a**)

^1^H NMR (400 MHz, DMSO-d_6_, δ, ppm): 12.07–11.66 (m, 1H, NH), 11.21 (bs, 1H, NH), 8.53 (d, J = 7.7 Hz, 1H, isatin), 7.39–7.33 (m, 1H, isatin), 7.35 (d, J = 8.9 Hz, 2H, Ar), 7.06 (d, J = 8.9 Hz, 2H, Ar), 7.02 (t, J = 8.2 Hz, 1H, isatin), 6.94 (d, J = 7.8 Hz, 1H, isatin), 4.,09 (q, J = 6.9 Hz, 2H, ArOCH_2_CH_3_), 1.36 (t, J = 6.9 Hz, 3H, ArOCH_2_CH_3_). IR (pellet KBr): *ν*_max_ 3314, 3189, 2980, 2938, 2885, 2161, 1775, 1727, 1691, 1668, 1643, 1614, 1587, 1516, 1477, 1465, 1414, 1352, 1301, 1258, 1228, 1203, 1172, 1117, 1099, 1046, 975, 926, 886, 820, 793, 767, 748, 681, 628 cm^−1^.

(Z)-3-(3-(4-ethoxyphenyl)-4-oxo-2-selenoxoimidazolidin-5-ylidene)-5-chloroindolin-2-one (**4b**)

^1^H NMR (400 MHz, DMSO-d6, δ, ppm): 11.95 (bs, 1H, NH), 11.28 (s, 1H, NH), 8.57 (d, J = 1.9 Hz, 1H, isatin), 7.40 (dd, J_1_ = 2.0 Hz, J_2_ = 8.3 Hz, 1H, isatin), 7.34 (d, J = 8.8 Hz, 2H, Ar), 7.07 (d, J = 8.8 Hz, 2H, Ar), 6.94 (d, J = 8.4 Hz, 1H, isatin), 4.10 (q, J = 6.9 Hz, 2H, ArOCH_2_CH_3_), 1.37 (t, J = 6.9 Hz, 3H, ArOCH_2_CH_3_). IR (pellet KBr): *ν*_max_ 3430, 3351, 3288, 3173, 3110, 3077, 2983, 2933, 1777, 1741, 1732, 1701, 1694, 1636, 1629, 1611, 1588, 1515, 1476, 1458, 1433, 1419, 1395, 1377, 1293, 1258, 1225, 1190, 1171, 1148, 1117, 1073, 1046, 984, 957, 923, 901, 894, 874, 839, 828, 777, 757, 729, 699, 685, 650, 637, 620, 590, 574 cm^−1^.

(Z)-3-(3-(4-ethoxyphenyl)-4-oxo-2-selenoxoimidazolidin-5-ylidene)-5-bromoindolin-2-one (**4c**)

^1^H NMR (400 MHz, DMSO-d6, δ, ppm): 11.96 (s, 1H, NH), 11.30 (s, 1H, NH), 8.71 (d, J = 1.9 Hz, 1H, isatin), 7.53 (dd, J_1_ = 2.0 Hz, J_2_ = 8,4 Hz, 1H, isatin), 7.34 (d, J = 8.9 Hz, 2H, Ar), 7.07 (d, J = 9.9 Hz, 2H, Ar), 6.90 (d, J = 8.3, 1H, isatin), 4.10 (q, J = 7.0 Hz, 2H, ArOCH_2_CH_3_), 1.37 (t, J = 7.0 Hz, 3H, ArOCH_2_CH_3_). IR (pellet KBr): *ν*_max_ 3415, 3349, 3287, 3173, 3109, 3075, 2983, 2934, 2884, 2162, 1776, 1741, 1700, 1628, 1609, 1589, 1514, 1475, 14561435, 1418, 1394, 1292, 1254, 1190, 1171, 1116, 1046, 978, 957, 923, 902, 871, 825, 775, 756, 733, 682, 620, 589 cm^−1^.

(Z)-3-(3-(4-ethoxyphenyl)-4-oxo-2-selenoxoimidazolidin-5-ylidene)-5-nitroindolin-2-one (**4d**)

^1^H NMR (400 MHz, DMSO-d6, δ, ppm): 12.11 (m, 1H, NH), 11.80 (s, 1H, NH), 9.48 (s, 1H, isatin), 8.29 (dd, J_1_ = 2.0 Hz, J_2_ = 8.8 Hz, 1H, isatin), 7.37 (d, J = 8.8 Hz, 2H, Ar), 7.14 (d, J = 8.8 Hz, 1H, isatin), 7.08 (d, J = 8.8 Hz, 2H, Ar), 4.11 (q, J = 6.9 Hz, 2H, ArOCH_2_CH_3_), 1.37 (t, J = 6.9 Hz, 3H, ArOCH_2_CH_3_).

(Z)-3-(3-(4-methoxyphenyl)-4-oxo-2-selenoxoimidazolidin-5-ylidene)-5-bromoindolin-2-one (**4e**)

^1^H NMR (400 MHz, DMSO-d6, δ, ppm): 10.98 (bs, 1H, NH), 8.83 (s, 1H, isatin), 7.43 (d, J = 7.6 Hz, 1H, isatin), 7.29 (d, J = 8.6 Hz, 2H, Ar), 7.05 (d, J = 8.7 Hz, 2H, Ar), 6.81 (d, J = 8.5 Hz, 1H, isatin), 3.82 (s, 3H, OCH_3_).

(Z)-3-(3-(4-methoxyphenyl)-4-oxo-2-selenoxoimidazolidin-5-ylidene)-5-nitroindolin-2-one (**4f**)

^1^H NMR (400 MHz, DMSO-d6, δ, ppm): 12.04 (bs, 1H, NH), 11.80 (s, 1H, NH), 9.48 (d, J = 2.3 Hz, 1H, isatin), 8.29 (dd, J_1_ = 2.4 Hz, J_2_ = 8.7 Hz, 1H, isatin), 7.39 (d, J = 8.8 Hz, 2H, Ar), 7.15–7.12 (m, 1H, isatin), 7.11 (d, J = 9.1 Hz, 2H, Ar), 3.84 (s, 3H, OCH_3_).

(Z)-3-(3-(4-chlorophenyl)-4-oxo-2-selenoxoimidazolidin-5-ylidene)-5-bromoindolin-2-one (**4g**)

^1^H NMR (400 MHz, DMSO-d6, δ, ppm): 12.06 (s, 1H, NH), 11.31 (s, 1H, NH), 8.70 (d, J = 1.7 Hz, 1H, isatin), 7.65 (d, J = 8,6 Hz, 2H,Ar), 7,55–7,52 (m, 1H,isatin), 7,51 (d, J = 8,7 Hz, 2H,Ar), 6,90 (d, J = 8,3 Hz, 1H,isatin).

(Z)-3-(3-(4-chlorophenyl)-4-oxo-2-selenoxoimidazolidin-5-ylidene)-5-nitroindolin-2-one (**4h**)

^1^H NMR (400 MHz, DMSO-d6, δ, ppm): 12.19 (bs, 1H, NH), 11.81 (s, 1H, NH), 9.46 (d, J = 2.2 Hz, 1H, isatin), 8.29 (dd, J_1_ = 2.3 Hz, J_2_ = 8.7 Hz, 1H, isatin), 7.66 (d, J = 8.6 Hz, 2H, Ar), 7.52 (d, J = 8.6 Hz, 2H, Ar), 7.12 (d, J = 8.7 Hz, 1H, isatin).

(Z)-3-(3-(3-chlorophenyl)-4-oxo-2-selenoxoimidazolidin-5-ylidene)indolin-2-one (**4i**)

^1^H NMR (400 MHz, DMSO-d6, δ, ppm): 12.03 (s, 1H, NH), 11.20 (s, 1H, NH), 8.53 (d, J = 7.7 Hz, 1H, isatin), 7.64–7.57 (m, 3H, Ar), 7.50–7.45 (m, 1H, Ar), 7.37 (t, J = 7.7 Hz, 1H, isatin), 7.02 (t, J = 7.7 Hz, 1H, isatin), 6.95 (d, J = 7.8 Hz, 1H, isatin).

(Z)-3-(3-(3-chloro-4-fluorophenyl)-4-oxo-2-selenoxoimidazolidin-5-ylidene)-5-chloroindolin-2-one (**4j**)

^1^H NMR (400 MHz, DMSO-d6, δ, ppm): 12.14 (s, 1H, NH), 11.31 (s, 1H, NH), 8.57 (d, J = 1.8 Hz, 1H, Ar), 7.79 (dd, J_1_ = 2.3 Hz, J_2_ = 6.9 Hz, 1H, Ar), 7.66 (t, J = 8.9 Hz, 1H, Ar), 7.58–7.51 (m, 1H, Ar), 7.43 (dd, J_1_ = 2.1 Hz, J_2_ = 8.3 Hz, 1H, Ar), 6.96 (d, J = 8.3 Hz, 1H, Ar).

(Z)-3-(3-cyclopropyl-4-oxo-2-selenoxoimidazolidin-5-ylidene)-5-bromoindolin-2-one (**4k**)

^1^H NMR (400 MHz, DMSO-d6, δ, ppm): 11.67 (s, 1H, NH), 11.24 (s, 1H, NH), 8.71 (d, J = 2.0 Hz, 1H, isatin), 7.52 (dd, J_1_ = 2.0 Hz, J_2_ = 8.3 Hz, 1H, isatin), 6.87 (d, J = 8.3 Hz, 1H, isatin), 2.90–2.84 (m, 1H, Pr), 1.08–1.01 (m, 4H, Pr).

(Z)-3-(3-(4-methoxybenzyl)-4-oxo-2-selenoxoimidazolidin-5-ylidene)indolin-2-one (**4l**)

^1^H NMR (400 MHz, DMSO-d6, δ, ppm): 11.85 (bs, 1H, NH), 11.14 (s, 1H, NH), 8.56 (d, J = 7.8 Hz, 1H, isatin), 7.39–7.34 (m, 1H, isatin), 7.36 (d, J = 8.7 Hz, 2H, Ar), 7.03 (t, J = 7.6 Hz, 1H, isatin), 6.91 (d, J = 7.1 Hz, 1H, isatin), 6.90 (d, J = 8.8 Hz, 2H, Ar), 5.08 (s, 2H, CH_2_), 3.72 (s, 3H, OCH_3_). IR (pellet KBr): *ν*_max_ 3254, 2958, 2835, 1772, 1724, 1694, 1635, 1612, 1586, 1514, 1464, 1435, 1405, 1340, 1308, 1248, 1197, 1177, 1146, 1097, 1034, 962, 908, 848, 819, 774, 755, 681, 631 cm^−1^. IR (pellet KBr): *ν*_max_ 3335, 3195, 3110, 3002, 2958, 2935, 2836, 1777, 1728, 1695, 1644, 1612, 1587, 1515, 1456, 1433, 1404, 1342, 1305, 1249, 1197, 1176, 1145, 1108, 1033, 979, 937, 903, 877, 844, 816, 789, 773, 746, 715, 688, 681, 626, 578 cm^−1^.

(Z)-3-(3-(4-methoxybenzyl)-4-oxo-2-selenoxoimidazolidin-5-ylidene)-5-bromoindolin-2-one (**4m**)

^1^H NMR (400 MHz, DMSO-d6, δ, ppm): 11.87 (s, 1H, NH), 11.25 (s, 1H, NH), 8.71 (s, 1H, isatin), 7.51 (dd, J_1_ = 1.4 Hz, J_2_ = 8.4 Hz, 1H, isatin), 7.37 (d, J = 8.5 Hz, 2H, Ar), 6.90 (d, J = 8.7 Hz, 2H, Ar), 6.87 (d, J = 8.5 Hz, 1H, isatin), 5.08 (s, 2H, CH_2_), 3.72 (s, 3H, OCH_3_).

#### 3.2.8. Synthesis of Dispiroindolinones of Type I (Compounds **5a**-**h**; General Procedure)

Ethanol was added to 5-arylidene-2-selenoxohydantoin **3** (1 equiv.) and sarcosine (2 equiv.), and the mixture was brought to a boil. Then, isatin (2 equiv.) was added. The reaction mixture was refluxed for 5 h. The precipitate formed was filtered off and purified by flash chromatography (eluent MeOH: CHCl_3_ = 1:50 (*v*/*v*)) and yielded 14–66%.

3-(4-ethoxyphenyl)-4′-(4-chlorophenyl)-1′-methyl-2-selenoxodispiro[imidazolidine-5.3′-pyrrolidine-2′.3″-indoline]-2″.4-dione (**5a**)

R*_f_* = 0.84 (MeOH:CHCl_3_ = 1:50).

^1^H NMR (400 MHz, DMSO-d6, δ, ppm): 10.94 (s, 1H, NH), 10.69 (s, 1H, NH), 7.47 (d, J = 8.,6 Hz, 2H, Ar), 7.42 (d, J = 8.6 Hz, 2H, Ar), 7.33 (d, J = 7.5 Hz, 1H, isatin), 7.30 (t, J = 7.6 Hz, 1H, isatin), 7.01 (t, J = 7.6 Hz, 1H, isatin), 6.88 (d, J = 8.9 Hz, 2H, Ar), 6.84 (d, J = 7.6 Hz, 1H, isatin), 6.51 (d, J = 8.8 Hz, 2H, Ar), 4.32 (t, J = 9.1 Hz, 1H, pyrrolidine), 4.00 (q, J = 7.0 Hz, ArOCH_2_CH_3_), 3.98 (t, J = 8.9 Hz, pyrrolidine), 3.47 (t, J = 8.8 Hz, 1H, pyrrolidine), 2.13 (s, 3H, NCH_3_), 1.30 (t, J = 6.9 Hz, 3H, ArOCH_2_CH_3_).^13^C NMR (101 MHz, DMSO-d6, δ, ppm): 182.2, 174.8, 171.8, 158.6, 142.7, 133.7, 132.3, 131.3, 130.0, 129.5, 128.4, 126.9, 125.7, 123.6, 121.6, 114.3, 109.9, 77.8, 76.9, 63.3, 56.2, 49.5, 34.6, 14.5. IR (pellet KBr): *ν*_max_ 3203, 3085, 2980, 2944, 2878, 2795, 1748, 1713, 1620, 1512, 1469, 1398, 1327, 1296, 1249, 1218, 1160, 1113, 1090, 1045, 1016, 834, 773, 759 cm^−1^. HRMS (ESI, *m*/*z*): calculated (C_28_H_25_ClN_4_O_3_Se, [M+H]^+^): 581.0853, found ([M+H]^+^: 581.0860.

5″-chloro-3-(4-ethoxyphenyl)-4′-(4-chlorophenyl)-1′-methyl-2-selenoxodispiro[imidazolidine-5,3′-pyrrolidine-2′,3″-indoline]-2″,4-dione (**5b**)

R*_f_* = 0.81 (MeOH:CHCl_3_ = 1:50).

^1^H NMR (400 MHz, DMSO-d6, δ, ppm): 11.24 (s, 1H, NH), 10.81 (s, 1H, NH), 7.47 (d, J = 8.6 Hz, 2H, Ar), 7.41 (d, J = 8.3 Hz, 2H, Ar), 7.43–7.39 (m, 1H, isatin), 7.36 (dd, J_1_ = 2.1 Hz, J_2_ = 8.3 Hz, 1H, isatin), 6.90 (d, J = 8.9 Hz, 2H, Ar), 6.86 (d, J = 8.3 Hz, 1H, isatin), 6.54 (d, J = 8.8 Hz, 2H, Ar), 4.34 (t, J = 9.0 Hz, 1H, pyrrolidine), 4.01 (q, J = 6.9 Hz, ArOCH_2_CH_3_), 3.94 (t, J = 9.7 Hz, 1H, pyrrolidine), 3.50 (t, J = 8.6 Hz, 1H, pyrrolidine), 2.16 (s, 3H, NCH_3_), 1.31 (t, J = 6.9 Hz, 3H, ArOCH_2_CH_3_).^13^C NMR (101 MHz, DMSO-d6, δ, ppm): 182.1, 174.4, 171.5, 158.7, 141.5, 133.5, 132.4, 131.5, 129.9, 129.5, 128.4, 126.9, 125.9, 125.8, 125.7, 114.5, 111.4, 78.0, 77.1, 63.4, 56.0, 49.6, 34.8, 14.6. IR (pellet KBr): *ν*_max_ 3424, 3249, 2979, 2933, 2867, 1716, 1618, 1513, 1495, 1470, 1447, 1300, 1251, 1220, 1167, 1115, 1091, 1043, 831, 820, 778 cm^−1^. HRMS (ESI, *m*/*z*): calculated (C_28_H_24_Cl_2_N_4_O_3_Se, [M+H]^+^): 615.0463, found [M+H]^+^: 615.0479.

5″-bromo-3-(4-ethoxyphenyl)-4′-(4-chlorophenyl)-1′-methyl-2-selenoxodispiro[imidazolidine-5,3′-pyrrolidine-2′,3″-indoline]-2″,4-dione (**5c**)

R*_f_* = 0.82 (MeOH:CHCl_3_ = 1:50).

^1^H NMR (400 MHz, DMSO-d6, δ, ppm): 11.18 (s, 1H, NH), 10.82 (s, 1H, NH), 7.51–7.44 (m, 4H, Ar+isatin), 7.41 (d, J = 8.6 Hz, 2H, Ar), 6.90 (d, J = 8.9 Hz, 2H, Ar), 6.81 (d, J = 8.8 Hz, 1H, isatin), 6.55 (d, J = 8.8 Hz, 2H, Ar), 4.32 (t, J = 9.2 Hz, 1H, pyrrolidine), 4.01 (q, J = 7.0 Hz, ArOCH_2_CH_3_), 3.95 (t, J = 9.1 Hz, 1H, pyrrolidine), 3.50 (t, J = 9.1 Hz, 1H, pyrrolidine), 2.16 (s, 3H, NCH_3_), 1.31 (t, J = 6.9 Hz, 3H, ArOCH_2_CH_3_).^13^C NMR (101 MHz, DMSO-d6, δ, ppm): 182.1, 174.2, 171.6, 158.7, 141.9, 133.5, 132.8, 132.4, 131.4, 129.5, 129.4, 128.4, 126.2, 125.7, 123.2, 114.5, 113.6, 111.9, 77.0, 63.4, 49.5, 34.7, 14.6. HRMS (ESI, *m*/*z*): calculated (C_28_H_24_BrClN_4_O_3_Se, [M+H]^+^): 658.9958, found [M+H]^+^: 658.9949.

3-(4-methoxyphenyl)-4′-(4-chlorophenyl)-1′-methyl-2-selenoxodispiro[imidazolidine-5,3′-pyrrolidine-2′,3″-indoline]-2″,4-dione (**5d**)

R*_f_* = 0.88 (MeOH:CHCl_3_ = 1:50).

^1^H NMR (400 MHz, DMSO-d6, δ, ppm): 10.96 (s, 1H, NH), 10.69 (s, 1H, NH), 7.47 (d, J = 8.6 Hz, 2H, Ar), 7.42 (d, J = 8.5 Hz, 2H, Ar), 7.32 (d, J = 7.3 Hz, 1H, isatin), 7.30 (t, J = 7.6 Hz, 1H, isatin), 7.01 (t, J = 7.5 Hz, 1H, isatin), 6.90 (d, J = 9.1 Hz, 2H, Ar), 6.85 (d, J = 7.6 Hz, 1H, isatin), 6.52 (d, J = 8.8 Hz, 2H, Ar), 4.32 (t, J = 9.1 Hz, 1H, pyrrolidine), 3.98 (t, J = 9.3 Hz, 1H, pyrrolidine), 3.74 (s, 3H, OCH_3_), 3.47 (t, J = 8.7 Hz, 1H, pyrrolidine), 2.13 (s, 3H, NCH_3_). ^13^C NMR (101 MHz, DMSO-d6, δ, ppm): 182.2, 174.9, 171.9, 159.3, 142.8, 133.8, 132.3, 131.4, 130.0, 129.6, 128.4, 126.9, 125.9, 123.6, 121.6, 114.0, 109.9, 77.9, 76.9, 56.3, 55.4, 49.5, 34.6. HRMS (ESI, *m*/*z*): calculated (C_27_H_23_ClN_4_O_3_Se, [M+H]^+^): 567.0697, found [M+H]^+^: 567.0709.

5″-chloro-3-(4-methoxyphenyl)-4′-(4-chlorophenyl)-1′-methyl-2-selenoxodispiro[imidazolidine-5,3′-pyrrolidine-2′,3″-indoline]-2″,4-dione (**5e**)

R*_f_* = 0.84 (MeOH:CHCl_3_ = 1:50).

^1^H NMR (400 MHz, DMSO-d6, δ, ppm): 11.24 (s, 1H, NH), 10.81 (s, 1H, NH), 7.47 (d, J = 8.7 Hz, 2H, Ar), 7.41 (d, J = 8.6 Hz, 2H, Ar), 7.40 (s, 1H, isatin), 7.36 (dd, J_1_ = 2.1 Hz, J_2_ = 8.3 Hz, 1H, isatin), 6.92 (d, J = 9.0 Hz, 2H, Ar), 6.86 (d, J = 8.2 Hz, 1H, isatin), 6.56 (d, J = 8.8 Hz, 2H, Ar), 4.34 (t, J = 8.8 Hz, 1H, pyrrolidine), 3.94 (t, J = 9.2 Hz, 1H, pyrrolidine), 3.75 (s, 3H, OCH_3_), 3.50 (t, J = 9.1 Hz, 1H, pyrrolidine), 2.16 (s, 3H, NCH_3_). ^13^C NMR (101 MHz, DMSO-d6, δ, ppm): 182.0, 174.3, 171.5, 159.4, 141.4, 133.4, 132.4, 131.4, 129.9, 129.4, 128.4, 126.8, 125.9, 125.8, 114.1, 111.4, 77.9, 77.1, 56.0, 55.4, 49.6, 34.7. HRMS (ESI, *m*/*z*): calculated (C_27_H_22_Cl_2_N_4_O_3_Se, [M+H]^+^): 601.0307, found [M+H]^+^: 601.0317.

5″-bromo-3-(4-methoxyphenyl)-4′-(4-chlorophenyl)-1′-methyl-2-selenoxodispiro[imidazolidine-5,3′-pyrrolidine-2′,3″-indoline]-2″,4-dione (**5f**)

R*_f_* = 0.87 (MeOH:CHCl_3_ = 1:50).

^1^H NMR (400 MHz, DMSO-d6, δ, ppm): 11.20 (s, 1H, NH), 10.82 (s, 1H, NH), 7.52–7.45 (m, 4H, Ar+isatin), 7.41 (d, J = 8.7 Hz, 2H, Ar), 6.92 (d, J = 8.7 Hz, 2H, Ar), 6.81 (d, J = 8.7 Hz, 1H, isatin), 6.56 (d, J = 8.7 Hz, 2H, Ar), 4.32 (t, J = 8.9 Hz, 1H, pyrrolidine), 3.95 (t, J = 9.2 Hz, 1H, pyrrolidine), 3.75 (s, 3H, OCH_3_), 3.50 (t, J = 9.2 Hz, 1H, pyrrolidine), 2.16 (s, 3H, NCH_3_). ^13^C NMR (101 MHz, DMSO-d6, δ, ppm): 182.1, 174.2, 171.7, 159.4, 141.9, 133.5, 132.8, 132.4, 131.5, 129.5, 129.4, 128.4, 126.2, 125.8, 114.1, 114.0, 113.6, 111.9, 78.0, 77.0, 56.1, 55.4, 49.5, 34.7. IR (pellet KBr): *ν*_max_ 3114, 2866, 1764, 1758, 1709, 1694, 1616, 1512, 1478, 1442, 1411, 1313, 1252, 1217, 1204, 1187, 1164, 1090, 1030, 1014, 941, 895, 831, 819, 775, 733, 694 cm^−1^. HRMS (ESI, *m*/*z*): calculated (C_27_H_22_BrClN_4_O_3_Se, [M+H]^+^): 644.9802, found [M+H]^+^: 644.9789.

5″-bromo-3-(4-methoxyphenyl)-4′-(4-ethylphenyl)-1′-methyl-2-selenoxodispiro[imidazolidine-5,3′-pyrrolidine-2′,3″-indoline]-2″,4-dione (**5g**)

R*_f_* = 0.90 (MeOH:CHCl_3_ = 1:50).

^1^H NMR (400 MHz, DMSO-d6, δ, ppm): 11.02 (s, 1H, NH), 10.83 (s, 1H NH), 7.52–7.45 (m, 2H, isatin), 7.33 (d, J = 6.6 Hz, 2H, Ar), 7.19 (d, J = 6.6 Hz, 2H, Ar), 6.91 (d, J = 7.7 Hz, 2H, Ar), 6.82 (d, J = 8.2 Hz, 1H, isatin), 6.53 (d, J = 7.5 Hz, 2H, Ar), 4.26 (t, J = 8.8 Hz, 1H, pyrrolidine), 3.96 (t, J = 8.9 Hz, 1H, pyrrolidine), 3.74 (s, 3H, OCH_3_), 3.55–3.50 (m, 1H, pyrrolidine), 2.59 (q, J = 7.2 Hz, 2H, ArCH_2_CH_3_), 2.16 (s, 3H, NCH_3_), 1.16 (t, J = 7.1 Hz, 3H, ArCH_2_CH_3_).^13^C NMR (101 MHz, DMSO-d6, δ, ppm): 182.0, 174.5, 171.8, 159.4, 143.2, 142.0, 132.8, 131.6, 129.6, 129.4, 127.9, 126.5, 126.0, 114.1, 113.6, 112.0, 78.2, 77.2, 56.2, 55.5, 50.2, 34.8, 27.9, 15.6. R (pellet KBr): *ν*_max_ 3183, 2963, 2835, 1751, 1725, 1617, 1605, 1511, 1468, 1399, 1300, 1250, 1215, 1185, 1163, 1021, 833, 779, 759, 738, 648, 633 cm^−1^. HRMS (ESI, *m*/*z*): calculated (C_29_H_27_BrN_4_O_3_Se, [M+H]^+^): 639.0342, found [M+H]^+^: 639.0506.

3-(4-methoxyphenyl)-4′-(4-methoxyphenyl)-1′-methyl-2-selenoxodispiro[imidazolidine-5,3′-pyrrolidine-2′,3″-indoline]-2″,4-dione (**5h**)

R*_f_* = 0.80 (MeOH:CHCl_3_ = 1:50).

^1^H NMR (400 MHz, DMSO-d6, δ, ppm): 10.78 (s, 1H, NH), 10.68 (s, 1H, NH), 7.40–7.32 (m, 3H, Ar+isatin), 7.29 (t, J = 7.0 Hz, 1H, isatin), 7.01 (t, J = 6.4 Hz, 1H, isatin), 6.95–6.86 (m, 4H, Ar), 6.84 (d, J = 7.2 Hz, 1H, isatin), 6.51 (d, J = 7.5 Hz, 2H, Ar), 4.28 (t, J = 8.3 Hz, 1H, pyrrolidine), 3.95 (t, J = 8.9 Hz, 1H, pyrrolidine), 3.74 (s, 3H, OCH_3_), 3.73 (s, 3H, OCH_3_), 3.43 (t, J = 8.7 Hz, 1H, pyrrolidine), 2.14 (s, 3H, NCH_3_). ^13^C NMR (101 MHz, DMSO-d6, δ, ppm): 181.9, 175.0, 171.8, 159.4, 142.6, 133.3, 132.5, 130.6, 130.0, 129.6, 127.1, 126.4, 125.6, 123.5, 121.6, 114.0, 113.9, 109.9, 78.0, 76.9, 56.5, 55.4, 55.1, 34.8. HRMS (ESI, *m*/*z*): calculated (C_28_H_26_N_4_O_4_Se, [M+H]^+^): 562.1119, found [M+H]^+^: 562.1191.

#### 3.2.9. Synthesis of Dispiroindolinones of Type I (Compounds **6a-m**; General Procedure)

Toluene was added to 5-indolinylidene-2-selenoxohydantoin (1 equiv.) and sarcosine (8 equiv.), and the mixture was brought to a boil. Paraform (8 eq.) was then added. The reaction mixture was refluxed for 6 h, then evaporated, and the residue was purified by column chromatography (eluent MeOH:CHCl_3_ = 1:50 (*v*/*v*)) and yielded 3–66%.

1′-methyl-3-(4-ethoxyphenyl)-2-selenoxodispiro[imidazolidine-5,3′-pyrrolidine-4′,3″-indoline]-2″,4-dione (**6a**)

R*_f_* = 0.42 (MeOH:CHCl_3_ = 1:50).

^1^H NMR (400 MHz, DMSO-d6, δ, ppm): 11.27 (s, 1H, NH), 10.63 (s, 1H, NH), 7.27 (t, J = 7.8 Hz, 1H, isatin), 7.13 (d, J = 7.5 Hz, 1H, isatin), 6.95–6.92 (m, 1H, isatin), 6.93 (d, J = 8.6 Hz, 2H, Ar), 6.86 (d, J = 7.6 Hz, 1H, isatin), 6.73 (d, J = 8.6 Hz, 2H, Ar), 4.03 (q, J = 7.0 Hz, 2H, ArOCH_2_CH_3_), 3.50 (d, J = 10.0 Hz, 1H, pyrrolidine), 3.34 (m, 1H, pyrrolidine), 3.33–3.29 (m, 1H, pyrrolidine), 3.08 (d, J = 10.1 Hz, 1H, pyrrolidine), 2.48 (s, 3H, NCH_3_), 1.32 (t, J = 6.9 Hz, 3H, ArOCH_2_CH_3_).^13^C NMR (101 MHz, DMSO-d6, δ, ppm): 183.5, 176.6, 172.7, 158.6, 143.0, 129.9, 129.5, 126.0, 125.0, 124.9, 121.4, 114.3, 109.6, 74.5, 63.3, 60.3, 58.8, 57.6, 42.0, 14.6. IR (pellet KBr): *ν*_max_ 3284, 3090, 2925, 2853, 2794, 2033, 1748, 1736, 1726, 1699, 1615, 1513, 1483, 1470, 1402, 1326, 1303, 1246, 1216, 1197, 1160, 1146, 1114, 1105, 1086, 1039, 939, 919, 870, 825, 789, 756, 735, 694, 674, 637, 615, 579, 554, 529 cm^−1^. HRMS (ESI, *m*/*z*): calculated (C_22_H_22_N_4_O_3_Se, [M+H]^+^): 471.0930, found [M+H]^+^: 471.0923.

5″-chloro-1′-methyl-3-(4-ethoxyphenyl)-2-selenoxodispiro[imidazolidine-5,3′-pyrrolidine-4′,3″-indoline]-2″,4-dione (**6b**)

R*_f_* = 0.34 (MeOH:CHCl_3_ = 1:50).

^1^H NMR (400 MHz, DMSO-d6, δ, ppm): 11.39 (s, 1H, NH), 10.78 (s, 1H, NH), 7.33 (dd, J_1_ = 2.0 Hz, J_2_ = 8,3 Hz, 1H isatin), 7.13 (d, J = 1.8 Hz, 1H, isatin), 6.94 (d, J = 8.8 Hz, 2H, Ar), 6.87 (d, J = 8.3 Hz, 1H, isatin), 6.76 (d, J = 8.6 Hz, 2H, Ar), 4.04 (q, J = 6.9 Hz, 2H, ArOCH_2_CH_3_), 3.42 (d, J = 11.7 Hz, 1H, pyrrolidine), 3.39 (d, J = 10.7 Hz, 1H, pyrrolidine), 3.29 (m, 1H, pyrrolidine), 3.10 (d, J = 10.1 Hz, 1H, pyrrolidine), 2.48 (s, 3H, NCH_3_), 1.33 (t, J = 6.9 Hz, 3H, ArOCH_2_CH_3_). ^13^C NMR (101 MHz, DMSO-d6, δ, ppm): 183.3, 175.8, 172.5, 158.7, 141.8, 129.8, 129.2, 127.4, 126.0, 125.4, 125.0, 114.4, 111.0, 74.5, 63.4, 60.1, 59.1, 57.6, 41.8, 14.6. IR (pellet KBr): *ν*_max_ 3093, 2982, 2944, 2859, 2799, 1759, 1737, 1699, 1611, 1591, 1514, 1486, 1475, 1456, 1404, 1333, 1315, 1304, 1280, 1257, 1212, 1197, 1163, 1116, 1090, 1042, 952, 926, 894, 869, 825, 789, 746, 737, 679, 587, 565c m^−1^. HRMS (ESI, *m*/*z*): calculated (C_22_H_21_ClN_4_O_3_Se, [M+H]^+^): 505.0540, found [M+H]^+^: 505.0547.

5″-bromo-1′-methyl-3-(4-ethoxyphenyl)-2-selenoxodispiro[imidazolidine-5,3′-pyrrolidine-4′,3″-indoline]-2″,4-dione (**6c**)

R*_f_* = 0.35 (MeOH:CHCl_3_ = 1:50).

^1^H NMR (400 MHz, DMSO-d6, δ, ppm): 11.42 (s, 1H, NH), 10.81(s, 1H, NH), 7.46 (dd, J_1_ = 1.4 Hz, J_2_ = 8.3 Hz, 1H, isatin), 7.24 (s, 1H, isatin), 6.94 (d, J = 8.6 Hz, 2H, Ar), 6.83 (d, J = 8.3 Hz, 1H, isatin), 6.77 (d, J = 8.4 Hz, 2H, Ar), 4.04 (q, J = 6.9 Hz, 2H, ArOCH_2_CH_3_), 3.43 (d, J = 10.3 Hz, 1H, pyrrolidine), 3.39 (d, J = 10.5 Hz, 1H, pyrrolidine), 3.32 (m, 1H, pyrrolidine), 3.10 (d, J = 10.1 Hz, 1H, pyrrolidine), 2.48 (s, 3H, NCH_3_), 1.33 (t, J = 6.9 Hz, 3H, ArOCH_2_CH_3_). ^13^C NMR (101 MHz, DMSO-d6, δ, ppm): 206.9, 183.3, 175.7, 172.5, 158.7, 142.3, 142.2, 132.0, 129.8, 127.7, 125.9, 114.4, 113.1, 111.5, 74.4, 63.3, 60.0, 59.0, 57.5, 41.9, 14.6. IR (pellet KBr): *ν*_max_3 089, 2984, 2930, 2852, 2799, 1760, 1738, 1699, 1613, 1591, 1514, 1489, 1471, 1456, 1407, 1315, 1305, 1280, 1256, 1212, 1196, 1165, 1116, 1086, 1042, 951, 929, 893, 865, 824, 788, 736 cm^−1^. HRMS (ESI, *m*/*z*): calculated (C_22_H_21_BrN_4_O_3_Se, [M+H]^+^): 549.0035, found [M+H]^+^: 549.0037.

5″-nitro-1′-methyl-3-(4-ethoxyphenyl)-2-selenoxodispiro[imidazolidine-5,3′-pyrrolidine-4′,3″-indoline]-2″,4-dione (**6d**)

R*_f_* = 0.21 (MeOH:CHCl_3_ = 1:50).

^1^H NMR (400 MHz, DMSO-d6, δ, ppm): 11.55 (s, 1H, NH), 11.38 (s, 1H, NH), 8.23 (dd, J_1_ = 2.1 Hz, J_2_ = 8.7 Hz, 1H, isatin), 8.06 (s, 1H, isatin), 7.06 (dd, J_1_ = 1.6 Hz, J_2_ = 8.6 Hz, 1H, isatin), 6.89 (d, J = 8.9 Hz, 2H, Ar), 6.74 (d, J = 8.6 Hz, 2H,Ar), 4.02 (q, J = 6.6 Hz, 2H, ArOCH_2_CH_3_), 3.50 (d, J = 10.9 Hz, 1H, pyrrolidine), 3.38–3.30 (m, 2H, pyrrolidine), 3.17 (d, J = 9.6 Hz, 1H, pyrrolidine), 2.50 (s, 3H, NCH_3_), 1.32 (t, J = 6.9 Hz, 3H, ArOCH_2_CH_3_). ^13^C NMR (101 MHz, DMSO-d6, δ, ppm): 183.0, 175.9, 172.0, 158.6, 149.1, 141.7, 129.6, 126.9, 126.5, 125.8, 121.0, 114.3, 109.8, 74.3, 63.3, 60.6, 59.1, 58.4, 41.6, 14.6. IR (pellet KBr): *ν*_max_ 3270, 3103, 2975, 2940, 2865, 2844, 2805, 1739, 1681, 1625, 1600, 1512, 1477, 1455, 1401, 1339, 1305, 1274, 1251, 1213, 1197, 1148, 1099, 1042, 979, 935, 916, 836, 788, 757, 741, 701, 606, 564 cm^−1^. HRMS (ESI, *m*/*z*): calculated (C_22_H_21_N_5_O_5_Se, [M+H]^+^): 516.0781, found [M+H]^+^: 516.0781.

5′’-bromo-1′-methyl-3-(4-methoxyphenyl)-2-selenoxodispiro[imidazolidine-5,3′-pyrrolidine-4′,3″-indoline]-2″,4-dione (**6e**)

R*_f_* = 0.40 (MeOH:CHCl_3_ = 1:50).

^1^H NMR (400 MHz, DMSO-d6, δ, ppm): 11.41 (s, 1H, NH), 10.81 (s, 1H, NH), 7.47 (d, J = 8.3 Hz, 1H, isatin), 7.25 (s, 1H, isatin), 6.97 (d, J = 8.4 Hz, 2H, Ar), 6.83 (d, J = 8.4 Hz, 1H, isatin), 6.79 (d, J = 8.5 Hz, 2H, Ar), 3.78 (s, 3H, OCH_3_), 3.44 (d, J = 10.1 Hz, 1H, pyrrolidine), 3.39 (d, J = 10.4 Hz, 1H, pyrrolidine), 3.31 (m, 1H, pyrrolidine), 3.10 (d, J = 10.5 Hz, 1H, pyrrolidine), 2.48 (s, 3H, NCH_3_). ^13^C NMR (101 MHz, DMSO-d6, δ, ppm): 206.9, 184.2, 175.7, 172.5, 159.4, 142.3, 132.0, 129.8, 127.7, 126.1, 114.0, 113.1, 111.5, 74.5, 63.7, 60.0, 59.0, 57.5, 55.4, 41.9. IR (pellet KBr): *ν*_max_ 3291, 3094, 2936, 2837, 2800, 1734, 1615, 1513, 1477, 1408, 1301, 1288, 1252, 1221, 1179, 1163, 1129, 1029, 952, 930, 891, 825, 788, 745, 733 cm^−1^. HRMS (ESI, *m*/*z*): calculated (C_21_H_19_BrN_4_O_3_Se, [M+H]^+^): 534.9876, found [M+H]^+^: 534.9879.

5″-nitro-1′-methyl-3-(4-methoxyphenyl)-2-selenoxodispiro[imidazolidine-5,3′-pyrrolidine-4′,3″-indoline]-2″,4-dione (**6f**)

R*_f_* = 0.23 (MeOH:CHCl_3_ = 1:50).

^1^H NMR (400 MHz, DMSO-d6, δ, ppm): 11.48 (bs, 1H, NH), 11.34 (s, 1H, NH), 8.42 (d, J = 2.4 Hz, 1H, isatin), 8.29 (dd, J_1_ = 2.4 Hz, J_2_ = 8.6 Hz, 1H, isatin), 7.05 (d, J = 9.0 Hz, 1H, isatin), 6.98 (d, J = 9.0 Hz, 2H, Ar), 6.91 (d, J = 9.0 Hz, 2H, Ar), 3.77 (s, 3H, OCH_3_), 3.45 (d, J = 11.0 Hz, 1H, pyrrolidine), 3.37 (m, 1H, pyrrolidine), 3.30 (m, 1H, pyrrolidine), 3.23 (d, J = 9.4 Hz, 1H, pyrrolidine), 2.54 (s, 3H, NCH_3_). HRMS (ESI, *m*/*z*): calculated (C_21_H_19_N_5_O_5_Se, [M+H]^+^): 502.0624, found [M+H]^+^: 502.0616.

5″-bromo-1′-methyl-3-(4-chlorophenyl)-2-selenoxodispiro[imidazolidine-5,3′-pyrrolidine-4′,3″-indoline]-2″,4-dione (**6g**)

R*_f_* = 0.37 (MeOH:CHCl_3_ = 1:50).

^1^H NMR (400 MHz, DMSO-d6, δ, ppm): 11.57 (s, 1H, NH), 10.83 (s, 1H, NH), 7.53 (d, J = 8.8 Hz, 2H, Ar), 7.46 (dd, J_1_ = 1.8 Hz, J = 8.3 Hz, 1H, isatin), 7.22 (s, 1H, isatin), 6.92 (d, J = 8.5 Hz, 2H, Ar), 6.83 (d, J = 8.3 Hz, 1H, isatin), 47 (d, J = 9.8 Hz, 1H, pyrrolidine), 3.43 (d, J = 10.2 Hz, 1H, pyrrolidine), 3.31–3.25 (m, 1H, pyrrolidine), 3.14 (d, J = 9.8 Hz, 1H, pyrrolidine), 2.49 (s, 3H, NCH_3_). ^13^C NMR (101 MHz, DMSO-d6, δ, ppm): 182.3, 175.6, 172.1, 142.2, 133.7, 132.5, 132.1, 130.6, 128.9, 127.6, 127.5, 113.1, 111.6, 74.7, 59.9, 59.0, 57.4, 41.9. IR (pellet KBr): *ν*_max_ 3311, 3088, 2941, 2852, 2798, 1746, 1733, 1616, 1505, 1493, 1478, 1406, 1299, 1287, 1260, 1220, 1179, 1165, 1127, 1090, 1016, 950, 890, 826, 819, 789, 742, 734, 719, 694,648 cm^−1^. HRMS (ESI, *m*/*z*): calculated (C_20_H_16_BrClN_4_O_2_Se, [M+H]^+^): 538.9383, found [M+H]^+^: 538.9379.

5″-nitro-1′-methyl-3-(4-chlorophenyl)-2-selenoxodispiro[imidazolidine-5,3′-pyrrolidine-4′,3″-indoline]-2″,4-dione (**6h**)

R*_f_* = 0.21 (MeOH:CHCl_3_ = 1:50).

^1^H NMR (400 MHz, DMSO-d6, δ, ppm): 11.74 (bs, 1H, NH), 11.48 (s, 1H, NH), 8.25 (dd, J_1_ = 2.4 Hz, J_2_ = 8.6 Hz, 1H, isatin), 8.02 (d, J = 2.0 Hz, 1H, isatin), 7.49 (d, J = 8.6 Hz, 2H, Ar), 7.,09 (d, J = 9.0 Hz, 1H, isatin), 6.92 (d, J = 8.2 Hz, 2H, Ar), 3.72–3.63 (m, 1H, pyrrolidine), 3.53–3.42 (m, 2H, pyrrolidine), 3.28–3.23 (m, 1H, pyrrolidine), 2.61 (bs, 3H, NCH_3_). ^13^C NMR (101 MHz, DMSO-d6, δ, ppm): 206.5, 175.8, 171.8, 149.3, 141.8, 133.7, 132.2, 130.4, 128.9, 126.8, 120.8, 110.0, 74.3, 58.9, 42.1, 30.7. IR (pellet KBr): *ν*_max_ 3274, 3095, 2931, 2853, 2801, 1732, 1623, 1600, 1524, 1495, 1474, 1403, 1339, 1306, 1270, 1201, 1092, 1016, 915, 833, 785, 754, 735, 698, 561 cm^−1^. HRMS (ESI, *m*/*z*): calculated (C_20_H_16_ClN_5_O_4_Se, [M+H]^+^): 506.0129, found [M+H]^+^: 506.0119.

1′-methyl-3-(3-chlorophenyl)-2-selenoxodispiro[imidazolidine-5,3′-pyrrolidine-4′,3″-indoline]-2″,4-dione (**6i**)

R*_f_* = 0.43 (MeOH:CHCl_3_ = 1:50).

^1^H NMR (400 MHz, DMSO-d6, δ, ppm): 11.47 (s, 1H, NH), 10.67 (s, 1H, NH), 7.54–7.41 (m, 2H, Ar), 7.28 (m, 1H, Ar), 7.11 (m, 1H, Ar), 6.99–6.76 (m, 4H, Ar), 3.49 (m, 1H, pyrrolidine), 3.44–3.37 (m, 2H, pyrrolidine), 3.09 (m, 1H, pyrrolidine), 2.49 (m, 3H, NCH_3_). ^13^C NMR (101 MHz, DMSO-d6, δ, ppm): 206.9, 176.4, 143.1, 135.0, 132.7, 130.3, 129.5, 129.0, 128.9, 127.7, 125.0, 124.8, 121.3, 109.7, 74.9, 60.1, 58.9, 57.4, 41.9. IR (pellet KBr): *ν*_max_ 3499, 3406, 3178, 3074, 2933, 2858, 2801, 1756, 1732, 1694, 1619, 1591, 1483, 1471, 1448, 1432, 1388, 1352, 1338, 1320, 1306, 1266, 1256, 1217, 1203, 1159, 1147, 1084, 1032, 968, 935, 905, 876, 782, 753, 745, 727, 688, 636, 620, 608, 582, 546 cm^−1^. HRMS (ESI, *m*/*z*): calculated (C_20_H_17_ClN_4_O_2_Se, [M + H]^+^): 461.0278, found [M+H]^+^: 461.0270.

5″-chloro-1′-methyl-3-(3-chloro-4-fluorophenyl)-2-selenoxodispiro[imidazolidine-5,3′-pyrrolidine-4′,3″-indoline]-2″,4-dione (**6j**)

R*_f_* = 0.31 (MeOH:CHCl_3_ = 1:50).

^1^H NMR (400 MHz, DMSO-d6, δ, ppm): 11.62 (s, 1H, NH), 10.81 (s, 1H, NH), 7.57 (t, J = 9.0 Hz, 1H,Ar), 7.36 (dd, J_1_ = 1.8 Hz, J_2_ = 8.3 Hz, 1H,Ar), 7.12 (m, 1H,Ar), 7.10 (m, 1H,Ar), 6.96 (m, 1H,Ar), 6.90 (d, J = 8.3 Hz, 1H,Ar), 3.43 (m, 2H, pyrrolidine), 3.32 (m, 1H, pyrrolidine), 3.12 (d, J = 10.2 Hz, 1H, pyrrolidine), 2.49 (s, 3H, NCH_3_),^13^C NMR (101 MHz, DMSO-d6, δ, ppm): 206.5, 175.7, 158.1, 156.4, 141.9, 130.9, 129.9, 129.2, 127.3, 125.3, 124.8, 119.5, 117.1, 111.1, 59.9, 59.2, 57.5, 41.8, 30.7, HRMS (ESI, *m*/*z*): calculated (C_20_H_15_Cl_2_FN_4_O_2_Se, [M+H]^+^): 512.9794, found [M+H]^+^: 512.9798.

5″-bromo-1′-methyl-3-cyclopropyl-2-selenoxodispiro[imidazolidine-5,3′-pyrrolidine-4′,3″-indoline]-2″,4-dione (**6k**)

R*_f_* = 0.48 (MeOH:CHCl_3_ = 1:50).

^1^H NMR (400 MHz, DMSO-d6, δ, ppm): 11.12 (bs, 1H, NH), 10.70 (s, 1H, NH), 7.40 (dd, J_1_ = 1.2 Hz, J_2_ = 8.2 Hz, 1H, isatin), 7.22 (d, J = 1.6 Hz, 1H, isatin), 6.75 (d, J = 8.2 Hz, 1H, isatin), 3.32–3.29 (m, 1H, pyrrolidine), 3.29–3.26 (m, 1H, pyrrolidine), 3.25 (d, J = 10.2 Hz, 1H, pyrrolidine), 3.09 (d, J = 9.8 Hz, 1H, pyrrolidine), 2.47 (s, 3H, NCH_3_), 0.91–0.76 (m, 3H, Pr), 0.68–0.60 (m, 1H, Pr), 0.20–0.12 (m, 1H, Pr). ^13^C NMR (101 MHz, DMSO-d6, δ, ppm): 184.2, 175.5, 173.1, 142.0, 131.8, 127.7, 127.5, 112.8, 111.3, 73.4, 60.3, 59.0, 57.8, 41.8, 24.8, 7.5, 5.7. R (pellet KBr): *ν*_max_ 3177, 2953, 2918, 2854, 2821, 2799, 1752, 1730, 1691, 1617, 1472, 1403, 1353, 1332, 1316, 1255, 1220, 1202, 1160, 1144, 1132, 1087, 1035, 955, 904, 876, 814, 789, 728, 700, 605, 580, 561, 545 cm^−1^. HRMS (ESI, *m*/*z*): calculated (C_17_H_17_BrN_4_O_2_Se, [M+H]^+^): 468.9773, found [M+H]^+^: 468.9767.

1′-methyl-3-(4-methoxybenzyl)-2-selenoxodispiro[imidazolidine-5,3′-pyrrolidine-4′,3″-indoline]-2″,4-dione (**6l**)

R*_f_* = 0.42 (MeOH:CHCl_3_ = 1:50).

^1^H NMR (400 MHz, DMSO-d6, δ, ppm): 11.16 (bs, 1H, NH), 10.55 (s, 1H, NH), 7.16 (t, J = 7.5 Hz, 1H, isatin), 7.06 (d, J = 7.3 Hz, 1H, isatin), 6.88 (d, J = 8.4 Hz, 2H, Ar), 6.77 (d, J = 7.7 Hz, 1H, isatin), 6.73 (d, J = 8.3 Hz, 2H, Ar), 6.70, (t, J = 7.2 Hz, 1H, isatin), 4.80 (d, J = 14.9 Hz, 1H, CH_2_), 4.67 (d, J = 14.9 Hz, 1H, CH_2_), 3.72 (s, 3H, OCH_3_), 3.36 (d, J = 10.1 Hz, 1H, pyrrolidine), 3.26 (d, J = 9.9 Hz, 1H, pyrrolidine), 3.19 (d, J = 10.1 Hz, 1H, pyrrolidine), 3.06 (d, J = 9.9 Hz, 1H, pyrrolidine), 2.44 (s, 3H, NCH_3_). ^13^C NMR (101 MHz, DMSO-d6, δ, ppm): 182.9, 176.2, 172.6, 158.3, 142.5, 129.0, 128.7, 128.6, 127.6, 125.3, 121.4, 113.5, 109.4, 73.6, 61.4, 58.9, 58.6, 55.0, 44.1, 42.6, 41.8. IR (pellet KBr): *ν*_max_ 3153, 3015, 2996, 2948, 2907, 2861, 2833, 2800, 1731, 1690, 1614, 1586, 1515, 1477, 1454, 1430, 1403, 1347, 1322, 1308, 1250, 1210, 1185, 1176, 1157, 1148, 1116, 1098, 1037, 964, 896, 849, 836, 823, 791, 777, 755, 730, 683, 637, 612, 600, 576, 559 cm^−1^. HRMS (ESI, *m*/*z*): calculated (C_22_H_22_N_4_O_3_Se, [M+H]^+^): 471.0930, found [M+H]^+^: 471.0930.

5″-bromo-1′-methyl-3-(4-methoxybenzyl)-2-selenoxodispiro[imidazolidine-5,3′-pyrrolidine-4′,3″-indoline]-2″,4-dione (**6m**)

R*_f_* = 0.41 (MeOH:CHCl_3_ = 1:50).

^1^H NMR (400 MHz, DMSO-d6, δ, ppm): 11.25 (s, 1H, NH), 10.68 (s, 1H, NH), 7.37 (dd, J_1_ = 2.0 Hz, J_2_ = 7.8 Hz, 1H, isatin), 7.34 (s, 1H, isatin), 6.82 (d, J = 7.8 Hz, 2H, Ar), 6.75–6.73 (m, 1H, isatin), 6.73 (d, J = 8.8 Hz, 2H, Ar), 4.80 (d, J = 14.7 Hz, 1H, CH_2_), 4.68 (d, J = 14.7 Hz, 1H, CH_2_), 3.72 (s, 3H, OCH_3_), 3.30 (m, 1H, pyrrolidine), 3.20 (d, J = 6.9 Hz, 1H, pyrrolidine), 3.17 (d, J = 7.8 Hz, 1H, pyrrolidine), 3.08 (d, J = 9.8 Hz, 1H, pyrrolidine), 2.43 (s, 3H, NCH_3_). ^13^C NMR (101 MHz, DMSO-d6, δ, ppm): 182.6, 175.2, 172.1, 158.3, 141.6, 131.7, 128.7, 128.6, 128.1, 127.5, 113.6, 113.2, 111.3, 73.7, 62.0, 59.7, 59.1, 55.0, 44.1, 41.5. IR (pellet KBr): *ν*_max_ 3206, 2998, 2934, 2833, 2804, 2030, 1749, 1729, 1689, 1615, 1587, 1515, 1470, 1428, 1398, 1342, 1306, 1250, 1203, 1177, 1150, 1120, 1036, 970, 907, 856, 821, 785, 729, 693, 612, 603, 583, 565, 545 cm^−1^. HRMS (ESI, *m*/*z*): calculated (C_22_H_21_BrN_4_O_3_Se, [M+H]^+^): 549.0035, found [M+H]^+^: 549.0045.

## 4. Conclusions

In the present study, two series of novel 2-selenohydantoin-based dispiro[pyrrolidine-oxindoles] were synthesized using the regio- and diastereoselective 1,3-dipolar cycloadditions of azomethine ylides to 5-arylidene and 5-indolidene-2-seleno-tetrahydro-4H-imidazole-4-ones. The synthesized seleno-containing oxindoles in the preliminary biotests demonstrated reasonable cytotoxic activity and the ability to generate ROS. For the compounds of **6**, the results obtained by measuring the ROS level in separate cells indicate the possibility of a correlation between their ability to generate reactive oxygen species and the effectiveness of their cytotoxic actions.

## Figures and Tables

**Table 1:** Cytotoxicity, µM (CC_50_ value—concentration of compounds that caused 50% death of cells), of some synthesized compounds **5** and **6** against different cell lines after 72 h of treatment, evaluated with the 3-(4,5-dimethylthiazol-2-yl)2,5-diphenyl tetrazolium bromide (MTT) test [42].

Compound	Cell Line				Selectivity Indexes	
	VA13	HEK293T	A549	MCF7	VA13/A549	HEK293T/A549
**5a**	79.9 ± 34.7	25.8 ± 3.6	15.6 ± 2.1	82.5 ± 18.3	5.1	1.7
**5b**	45.2 ± 23.3	19.8 ± 6	8.7 ± 2.5	20.9 ± 3.6	5.2	2.3
**5c**	57.6 ± 24.7	14.4 ± 5.4	8.4 ± 1.5	29.5 ± 9.1	6.9	1.7
**5d**	36.1 ± 4.5	33 ± 10.7	11.5 ± 2.5	54.3 ± 10.2	3.1	2.9
**5e**	31.3 ± 12.4	17.3 ± 7.4	10.1 ± 2.3	24.8 ± 5.1	3.1	1.7
**5f**	49 ± 8.4	16.2 ± 3.3	7.9 ± 0.9	33.1 ± 6.9	6.2	2.1
**6a**	20.4 ± 2.2	23.6 ± 3.3	20 ± 3.3	31.6 ± 7.1	1	1.2
**6b**	12.5 ± 1.5	14 ± 1.5	15.8 ± 1.5	20.4 ± 3.6	0.8	0.9
**6c**	19.3 ± 5.9	10.3 ± 1.3	11.9 ± 1.5	12.8 ± 2.3	1.6	0.9
**6d**	21.1 ± 2.4	27.07 ± 4.1	20.4 ± 1.8	35.1 ± 3	1	1.3
**6e**	11.8 ± 4.3	9.7 ± 5.2	7.6 ± 1.6	20.6 ± 1.4	1.6	1.3
**6g**	11.1 ± 0.6	15.3 ± 12.8	12 ± 6.7	16.9 ± 11.1	0.9	1.3
**6h**	8.5 ± 3.2	16.6 ± 2.7	11.3 ± 1.2	22.1 ± 2.7	0.8	1.5
**6i**	9.1 ± 0.4	13.1 ± 5.1	11 ± 0.4	17.2 ± 0.3	0.8	1.2
**6k**	45.7 ± 15.7	71.9 ± 27.5	54.9 ± 15	79.4 ± 5.9	0.8	1.3
**6l**	62.2 ± 21.4	43.2 ± 19	26.6 ± 0.9	>100	2.3	1.6
**6m**	16.1 ± 2.7	28.8 ± 5.1	14.7 ± 1.5	29 ± 5.7	1.1	2
dox	0.11 ± 0.04	0.020 ± 0.010	0.014 ± 0.005	0.037 ± 0.015	8.1	1.4

**Table 2:** p53 reporter activation test at the A549 cell line for some compounds of **5** and **6**. The “ONPG/MTT” columns indicate values of p53 activation normalized to the number of surviving cells (ONPG = ortho-Nitrophenyl-ß-galactoside). The ONPG/MTT parameter value ≥ 2 may indicate p53 activation [47].

Concentration, µM	100	25	6.25	1.56	100	25	6.25	1.56
Compound	ONPG/MTT				Survived Cells, %			
**Nutlin-3a**	8.62	6.83	3.82	1.97	77.34	103.42	103.41	106.33
**5b**	1.03	0.96	0.95	0.97	72.94	81.74	99.16	100.89
**5c**	0.96	0.86	0.97	0.78	72.54	66.33	68.71	89.37
**6a**	1.57	1.02	1.08	1.12	73.66	91.27	101.07	105.44
**6b**	1.22	1.31	1.11	1.09	45.20	52.40	83.34	80.78
**6c**	1.21	1.03	1.16	0.97	23.27	84.28	90.43	98.25
**6l**	2.08	0.92	0.94	1.15	50.91	79.87	94.15	93.47
**6m**	1.03	1.15	1.06	1.04	41.91	79.19	91.11	91.02

## Data Availability

The data presented in this study are available in the article or supplementary material.

## Supplementary Materials

The following are available online at https://www.mdpi.com/1422-0067/22/5/2613/s1.
