# Supplementary material for: Dispirooxindoles Based on 2-Selenoxo-Imidazolidin-4-Ones: Synthesis, Cytotoxicity and ROS Generation Ability
Source: Int J Mol Sci. 2021 Mar 5;22(5):2613. doi: 10.3390/ijms22052613 (PMC7961907; doi:10.3390/ijms22052613)
Supplement: Supplementary file 1 [file ijms-22-02613-s001.pdf]

Dispiroindolinones based on 2-selenoxoimidazolidin-4-ones: synthesis, cytotoxicity and ROS generation ability

Vladimir K. Novotortsev [1], Maxim E. Kukushkin [1,2], Viktor A. Tafeenko [1], Dmitry A. Skvortsov [1,3], Marina A. Kalinina [3], Roman V. Timoshenko [2], Nelly S. Chmelyuk [2], Liliya A. Vasilyeva [4], Boris N. Tarasevich [1], Petr V. Gorelkin [2], Alexander S. Erofeev [1,2], Alexander G. Majouga [1,2,5], Nikolai V. Zyk [1] and Elena K. Beloglazkina [1,*]

[1] Department of Chemistry, M.V.Lomonosov Moscow State University, Leninskie Gory, 1-3, 119991 Moscow, Russia; vladnov9216@rambler.ru (V.K.N.); lemmingg@mail.ru (M.E.K.); tafeenko-victor@yandex.ru (V.A.T.); skvorratd@mail.ru (D.A.S.); boris-tarasevich@yandex.ru (B.N.T.); erofeev@polly.phys.msu.ru (A.S.E.); alexander.majouga@gmail.com (A.G.M.); zyk@org.chem.msu.ru (N.V.Z.)

[2] National University of Science and Technology “MISiS”, Leninskiy Prospect, 4, 119049 Moscow, Russia; timoshenkoroma@mail.ru (R.V.T.); nellichmelyuk@yandex.ru (N.S.C.); peter.gorelkin@gmail.com (P.V.G.)

[3] Skolkovo Institute of Science and Technology, 4 Alfred Nobel Street, Skolkovo, 143025 Moscow, Russia; Marina.Kalinina@skoltech.ru

[4] Faculty of Bioengineering and Bioinformatics, Lomonosov Moscow State University, Leninskie Gory 1, 119991 Moscow, Russia; liljavasilyeva@gmail.com

[5] D. I. Mendeleev University of Chemical Technology of Russia, Miusskaya sq., 9, 125047 Moscow, Russia

Synthesis

General information

The reaction progress and the purity of substances were monitored by thin layer chromatography (TLC) on Silufol-UV254 plates with a fixed layer of silica gel. Melting points were determined in an open capillary block. The uncorrected values of melting points are given.

^{1}H and ^{13}C NMR NMR spectra were recorded on a Bruker Avance instrument with an operating frequency of 400 MHz for ^{1}H NMR, 101 MHz for ^{13}C NMR. The solvent used was deuterochloroform

and dimethyl sulfoxide-d6. Chemical shifts are given in parts per million on a scale of δ relative to hexamethyldisiloxane as an internal standard.

HRMS high-resolution mass spectra were recorded on an Orbitrap Elite mass spectrometer (Thermo Scientific) with IREP. To inject solutions with a concentration of 0.1–9 μg / ml (in 1% formic acid in acetonitrile), direct injection into the ion source using a syringe pump (5 μl / min) was used. Spray voltage ± 3.5 kV, capillary temperature 275℃.

X-ray diffraction studies were performed on a Syntex P21 diffractometer at 293 K (graphite monochromator, λ (MoKα) = 0.71073 Å, ω-scanning). The absorption was taken into account by measuring the intensities of equivalent reflections (Tmin / Tmax). The structures were solved by the direct method (SHELXS-97) and refined in full-matrix anisotropic least squares by F2 for all non-hydrogen atoms (SHELXL-97). All hydrogen atoms were objectively localized and refined in the isotropic approximation.

Glycine ethyl ester hydrochloride

In a three-necked flask equipped with a magnetic stirrer, reflux condenser and dropping funnel, 150 ml (2.50 mol) of ethanol are placed and cooled to -15 ° C. With vigorous stirring, 25 ml (0.35 mol) of $SOCl_2$ are added dropwise. Then, 25 g (0.33 mol) of glycine are added over 25 minutes. After this, the cooling is removed, the mixture is brought to a boil and boiled for 1 hour. The hot solution is filtered and cooled to 0 ° C. The precipitate was filtered, washed with diethyl ether (2 x 50 ml), dried to constant weight over P_2O_5, and recrystallized from ethanol-ether mixture (1: 1). The reaction yields 22.5 g (48%) of glycine ethyl ester hydrochloride as white crystals.

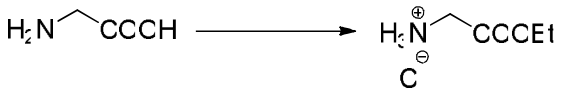

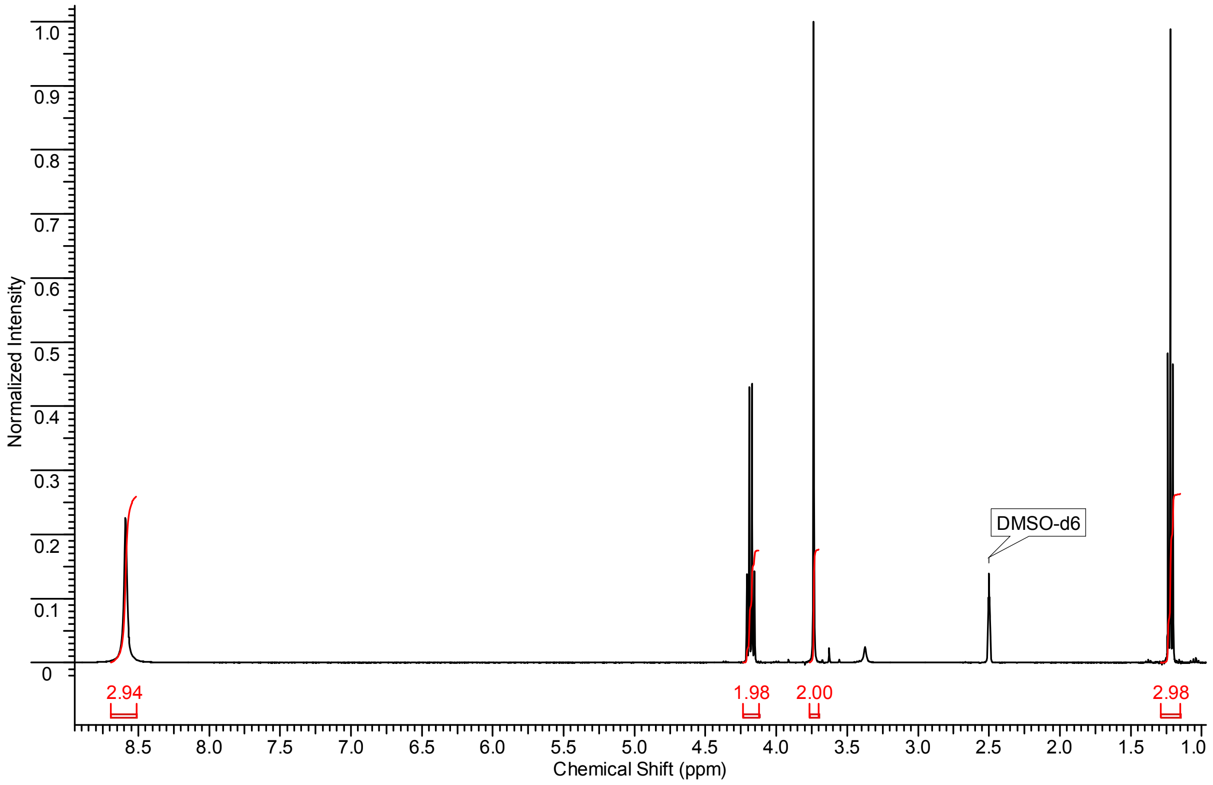

^{1}H NMR (400 MHz, DMSO-d_6, δ, ppm): 8.59 (s, 3H, NH_3^+), 4.18 (q, J=7.1 Hz, 2H, C(O)O$\underline{CH_2}$$CH_3$), 3.74 (s, 2H, CH_2), 1.22 (t, J=7.1 Hz, 3H, C(O)O$CH_2$$\underline{CH_3}$).

N-formyl glycine ethyl ester

To 90 ml of ethyl formate, 25 g (179.0 mmol) of glycine ethyl ester hydrochloride and 0.02 g (0.10 mmol) of p-TsOH are added, heated to boiling, then 25 ml (179.0 mmol) of Et_3N were added and boiled within 20 hours. Then the reaction mixture was cooled to room temperature, the precipitate was filtered off, and the filtrate was evaporated. Then the filtrate was cooled to -15 °C, the precipitate was filtered offagain, the filtrate was distilled under vacuum. The reaction gives 18.18 g (77%) of N-formyl glycine ethyl ester as a clear colorless oil (110°C / 0.1 mm Hg).

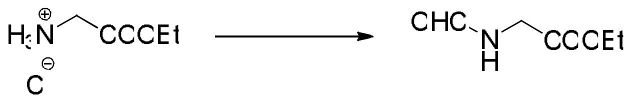

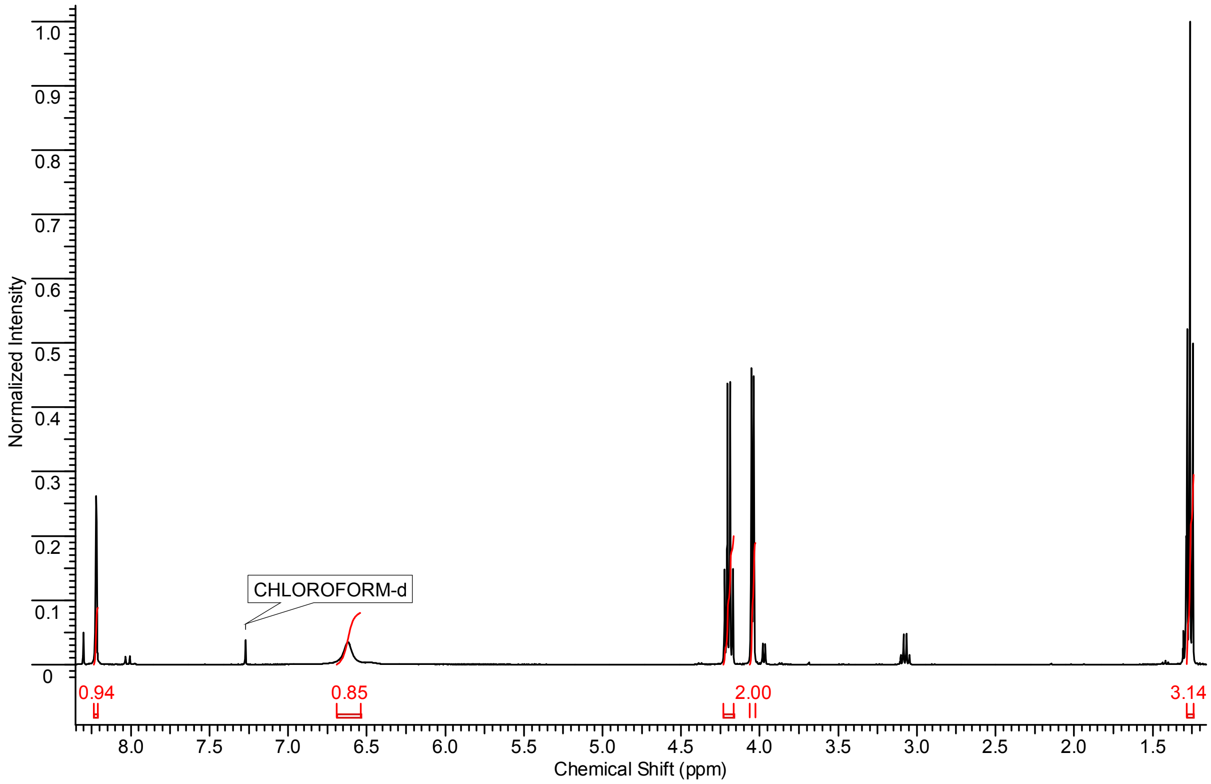

^{1}H NMR (400 MHz, $CDCl_3$, δ, ppm): 8.22 (s, 1H, CHO), 6.62 (bs, 1H, NH), 4.20 (q, J=7.2Hz, 2H, C(O)O<u>CH</u>$_2$$CH_3$), 4.05 (d, J=5.4Hz, 2H, CH_2), 1.26 (t, J=7.2Hz, 3H, C(O)OCH_2<u>CH</u>$_3$).

Ethyl 2-isocyanoacetate

To a solution of 14.57 g (111.1 mmol) of N-formyl glycine ethyl ester and 38.5 ml (275.5 mmol) of Et_3N in 115 ml of CH_2Cl_2, 10.2 ml (111.1 mmol) of $POCl_3$were added dropwise at 0 ° C. and stirred the reaction mixture for 1 hour at 0 ° C. Then at 20-25 °C and with vigorous stirring, a solution of 30 g of K_2CO_3 in 90 ml of H_2O was slowly added, after which the reaction mixture was stirred for 30 minutes at room temperature. The organic phase is then separated and the aqueous phase is diluted to 225 ml and extracted with CH_2Cl_2 (2 x 55 ml). After washing all three organic phases with brine, they are dried over Na_2SO_4, evaporated, and the residue was distilled under vacuum. The reaction gives 9.86 g (78%) of ethyl 2-isocyanoacetate as a clear yellowish oil (80-82 °C / 12 mmHg).

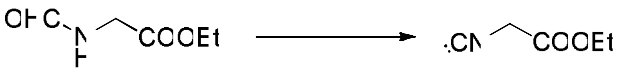

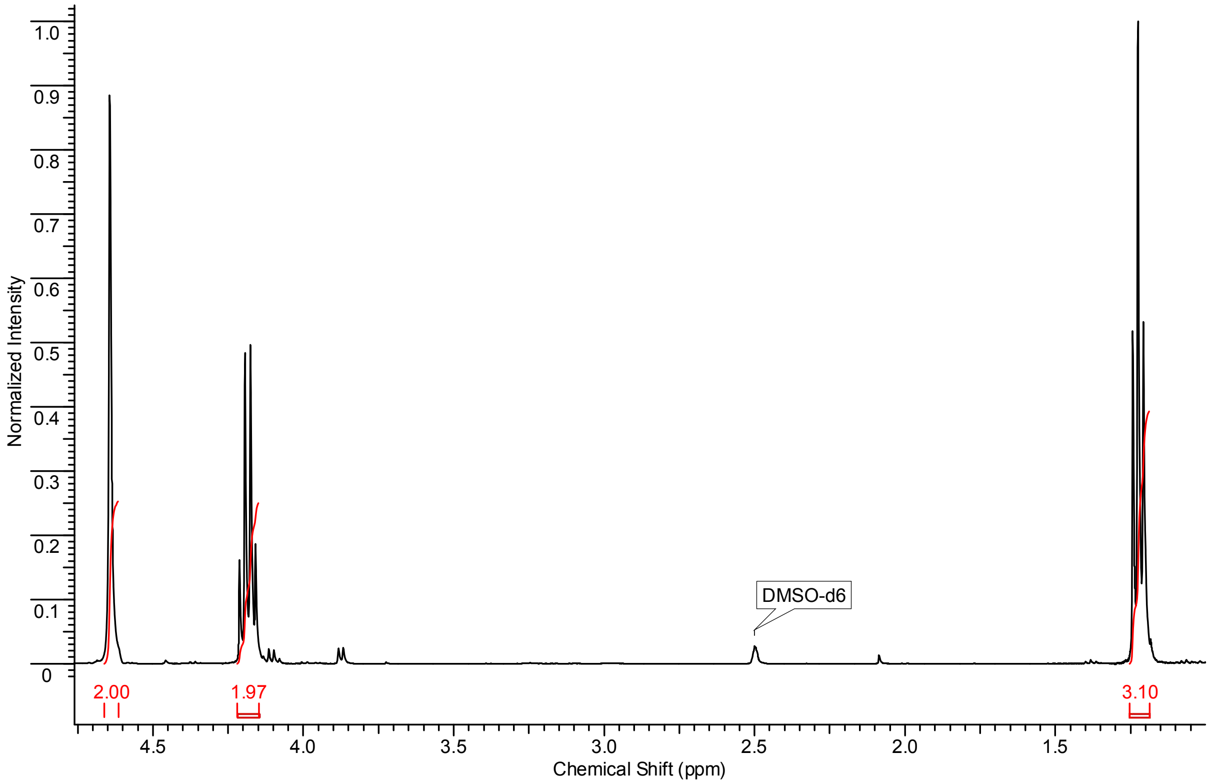

^{1}H NMR (400 MHz, DMSO-d_6, δ, ppm): 4.64 (s, 2H, CH_2), 4.18 (q, J=7.2 Hz, 2H, C(O)O$\underline{CH_2}$$CH_3$), 1.22 (t, J=7.1 Hz, 3H, C(O)O$CH_2$$\underline{CH_3}$).

Ethyl isoselenocyanatoacetate (1)

8.50 g (75.1 mmol) of Ethyl 2-isocyanoacetate were added to 240 ml of tetrahydrofuran in an argon atmosphere., 13.2 ml (94.8 mmol) Et_3N and 6.37 g (80.6 mmol) of selenium powder. The reaction mixture was boiled for 6 hours, then passed through Celite, evaporated and 120 ml of EtOAc are added. Then, the solution was washed with water (2 x 70 ml), dried over Na_2SO_4. The product was isolated by column chromatography in EtOAc: petroleum ether = 1: 9 system. As a result of the reaction, 7.64 g (53%) of compound **1** were obtained as a clear red liquid.

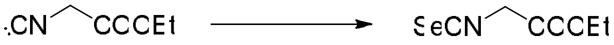

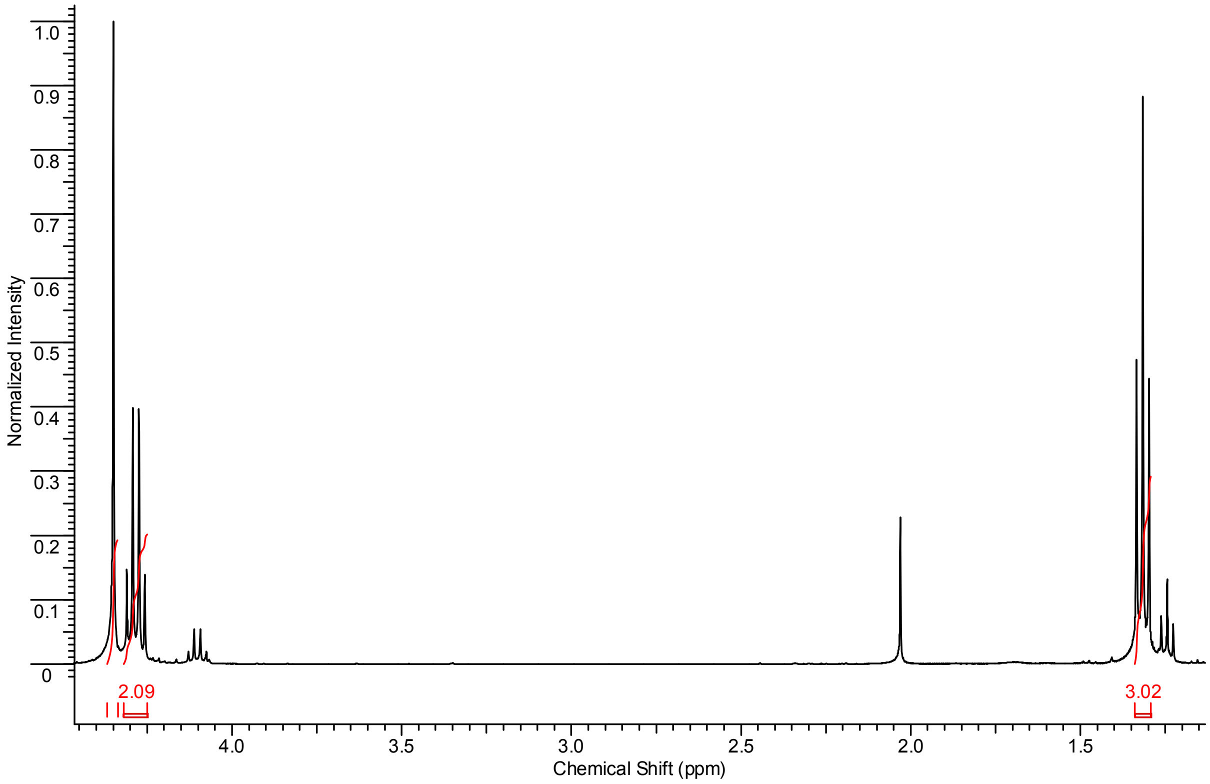

^{1}H NMR (400 MHz, $CDCl_3$, δ, ppm): 4.35 (s, 2H, CH_2), 4.28 (q, J=7.2 Hz, 2H, C(O)O$\underline{CH_2}$$CH_3$), 1.32 (t, J=7.2 Hz, 3H, C(O)O$CH_2$$\underline{CH_3}$).

Selenoureas and 2-selenoxohydantoins 2a-g *(general procedure).* Ethyl isoselenocyanatoacetate **1** (1 eq.) was dissolved in diethyl ether, then amine (1 eq.) was added in portions to the solution, after which 1 mol% of DMAP was added. The reaction mixture was stirred for 6 hours. In the case of precipitation, the precipitate was filtered off and washed with diethyl ether, and if necessary, purified by column chromatography. If a homogeneous solution forms, it was evaporated and the product was isolated by column chromatography

N-(4-ethoxyphenyl)-N'-carbethoxymethyl selenourea (2a)

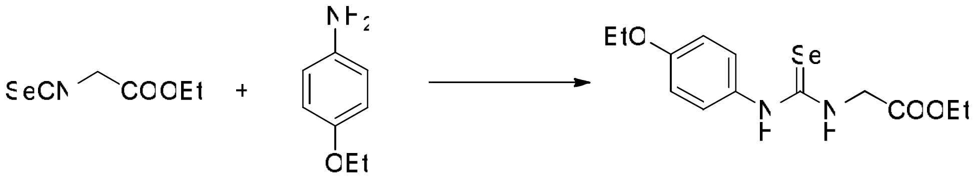

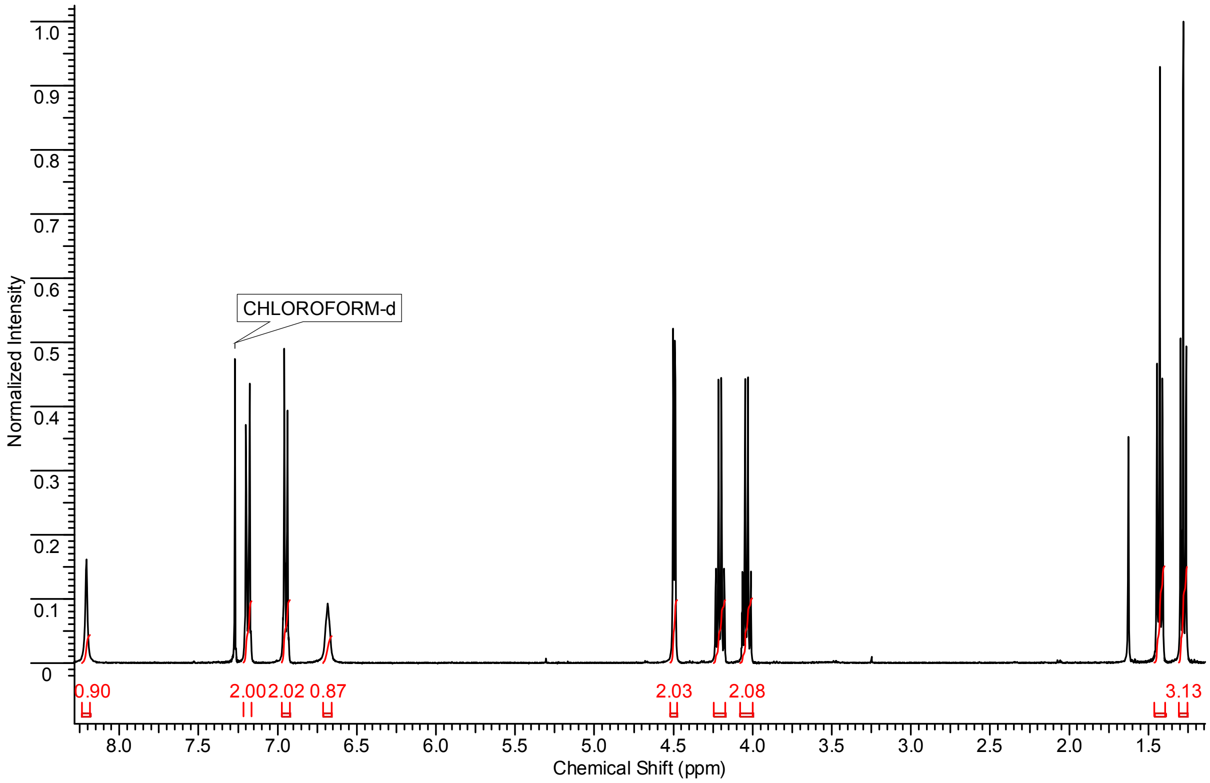

^{1}H NMR (400 MHz, $CDCl_3$, δ, ppm): 8.21 (s, 1H, NH), 7.19 (d, J=8.8 Hz, 2H, Ar), 6.95 (d, J=8.8 Hz, 2H, Ar), 6.68 (s, 1H, NH), 4.50 (d, J=4.7 Hz, 2H, CH_2), 4.21 (q, J=7.2 Hz, 2H, C(O)O$\underline{CH_2}$$CH_3$), 4.04 (q, J=7.0 Hz, 2H, ArO$\underline{CH_2}$$CH_3$), 1.43 (t, J=7.0 Hz, 3H, ArO$CH_2$$\underline{CH_3}$), 1.28 (t, J=7.2 Hz, 3H, C(O)O$CH_2$$\underline{CH_3}$).

N-(4-methoxyphenyl)-N'-carbethoxymethyl selenourea (2b)

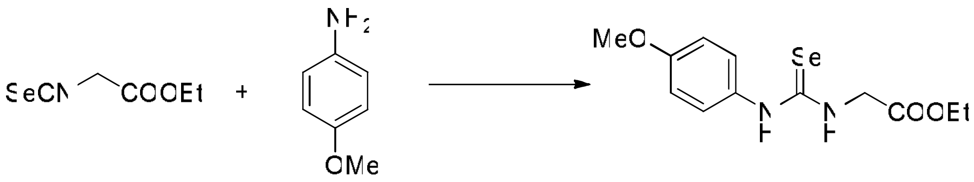

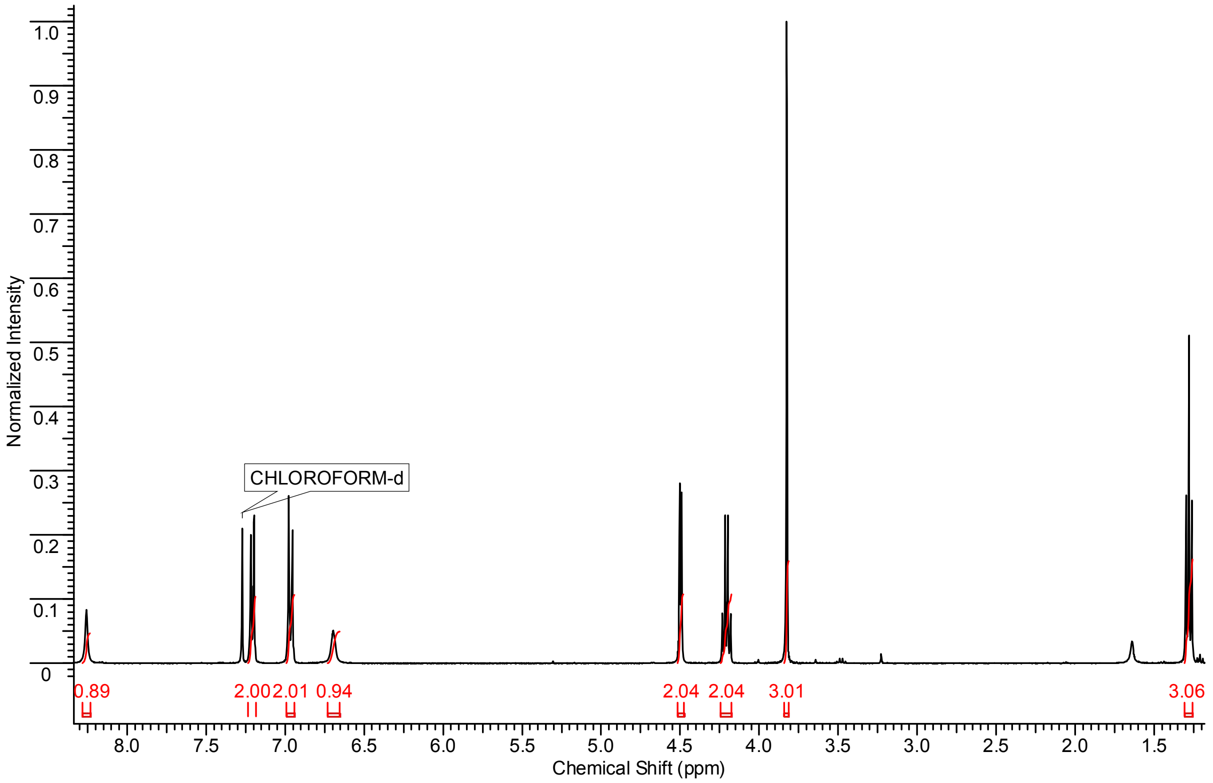

^{1}H NMR (400 MHz, $CDCl_3$, δ, ppm): 8.26 (s, 1H, NH), 7.21 (d, J=8.8 Hz, 2H, Ar), 6.97 (d, J=8.9 Hz, 2H, Ar), 6.69 (s, 1H, NH), 4.50 (d, J=4.7 Hz, 2H, CH_2), 4.21 (q, J=7.2 Hz, 2H, C(O)O$\underline{CH_2}CH_3$), 3.83 (s, 3H, OCH_3), 1.28 (t, J=7.2 Hz, 3H, C(O)$OCH_2\underline{CH_3}$).

N-(3-chloro-4-fluorophenyl)-N'-carbethoxymethyl selenourea (2c)

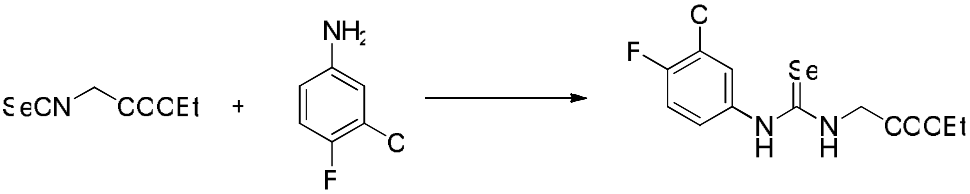

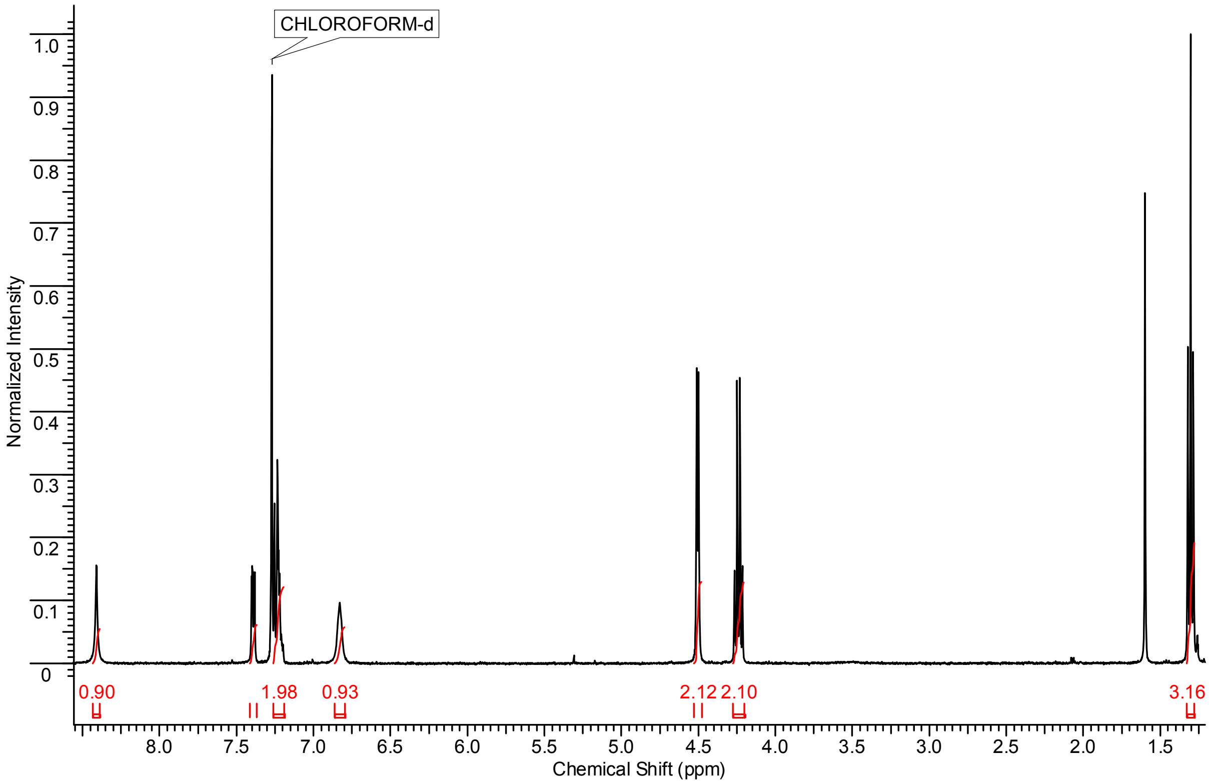

^{1}H NMR (400 MHz, $CDCl_3$, δ, ppm): 8.41 (s, 1H, NH), 7.39 (dd, J_1=2.2Hz, J_2=6.3 Hz, 1H, Ar), 7.26-7.19 (m, 2H, Ar) 6.83 (s, 1H, NH), 4.50 (d, J=4.6Hz, 2H, CH_2), 4.24 (q, J=7.2Hz, 2H, C(O)O$\underline{CH_2}$$CH_3$), 1.30 (t, J=7.2Hz, 3H, C(O)O$CH_2$$\underline{CH_3}$).

N-(4-chlorophenyl)-N'-carbethoxymethyl selenourea (2d)

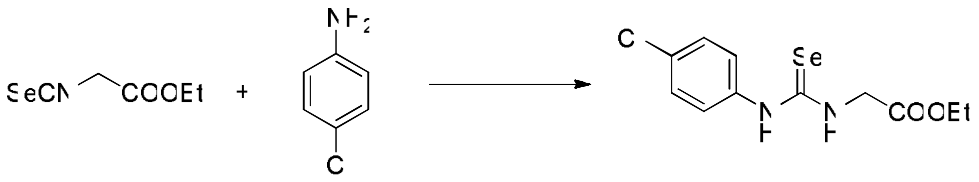

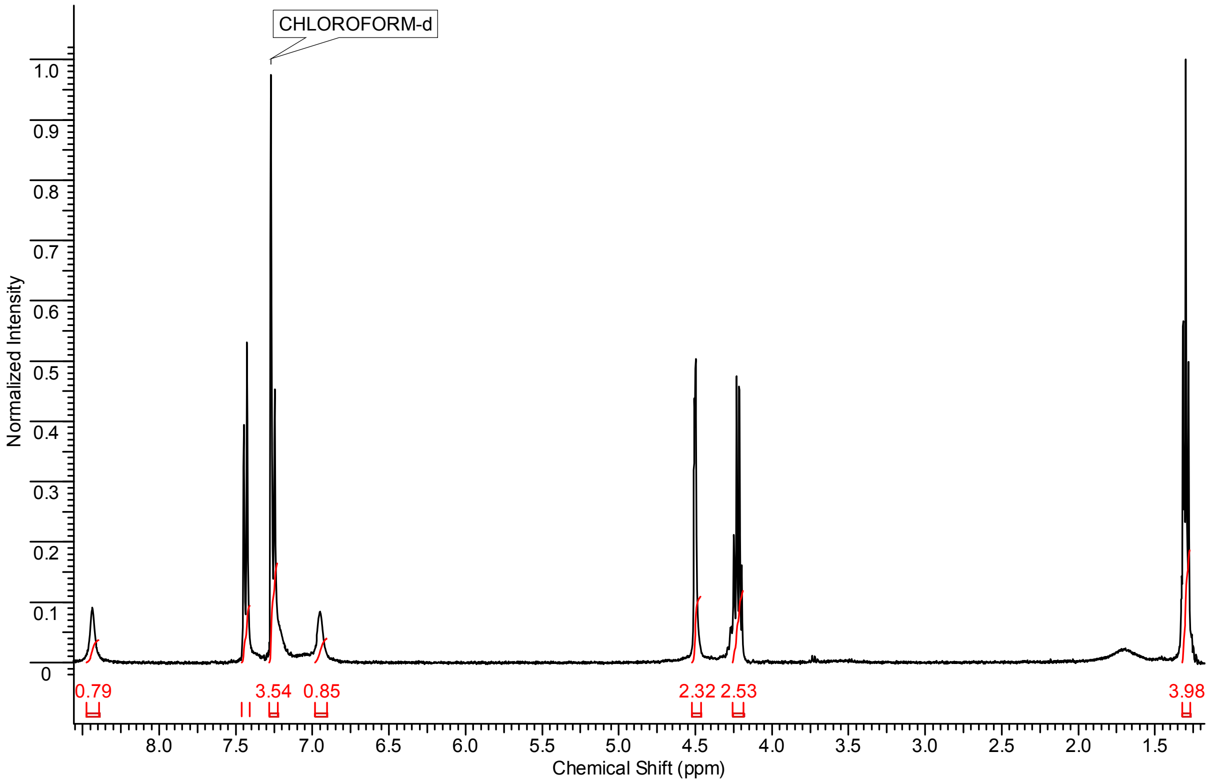

^{1}H NMR (400 MHz, $CDCl_3$, δ, ppm): 8.44 (s, 1H, NH), 7.44 (d, J=8.4 Hz, 2H, Ar), 7.26 (d, J=9,6 Hz, 2H, Ar), 6.95 (s, 1H, NH), 4.50 (d, J=3.6 Hz, 2H, CH_2), 4.22 (q, J=7.0 Hz, 2H, C(O)O$\underline{CH_2}$$CH_3$), 1.30 (t, J=7.2 Hz, 3H, C(O)O$CH_2$$\underline{CH_3}$).

N-cyclopropyl-N'-carbethoxymethyl selenourea (2e)

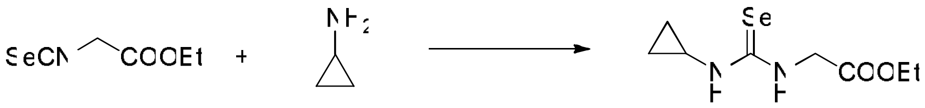

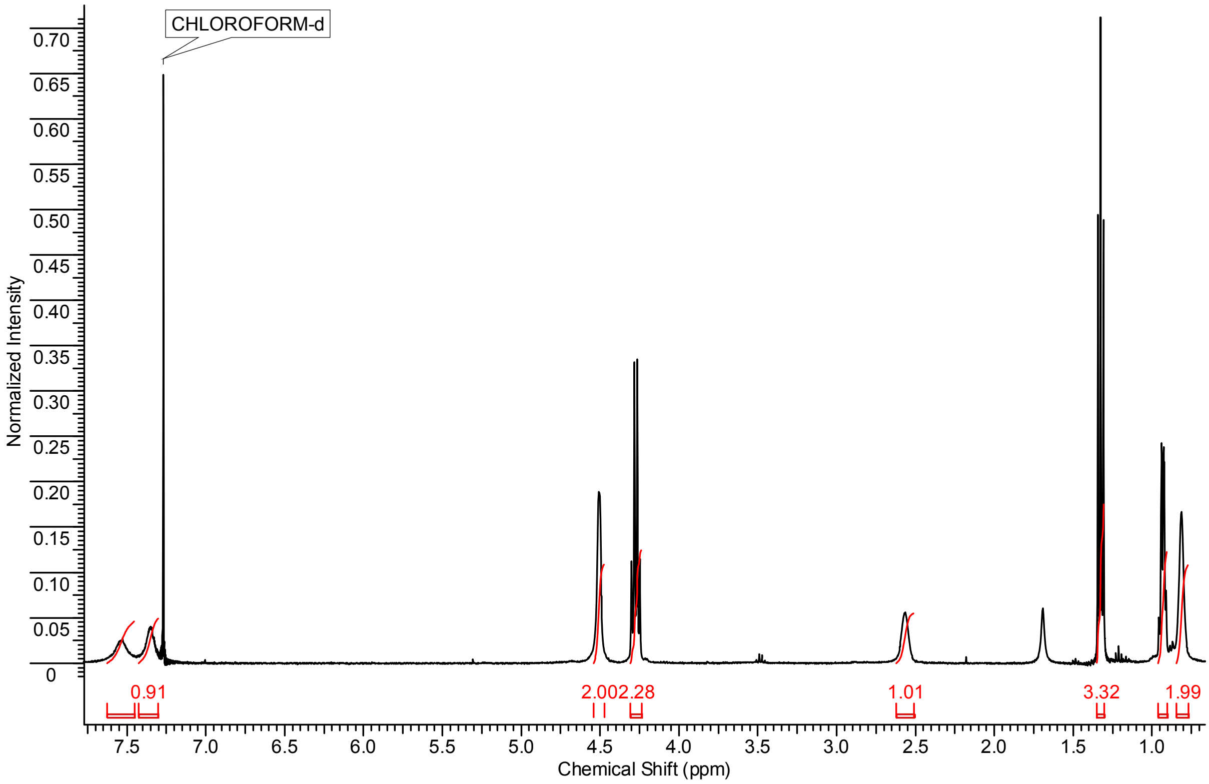

^{1}H NMR (400 MHz, $CDCl_3$, δ, ppm): 7.54 (bs, 1H, NH), 7.35 (bs, 1H, NH), 4.51 (s, 2H, CH_2), 4.27 (q, J=7.2 Hz, 2H, C(O)O<u>CH_2</u>CH_3), 2.57 (s, 1H, CHPr), 1.33 (t, J=7.2 Hz, 3H, C(O)OCH_2<u>CH_3</u>), 0.96-0.90 (m, 2H, Pr), 0.85-0.77 (m, 2H, Pr).

N-(3-chlorophenyl)-N'-carbethoxymethyl selenourea (2f)

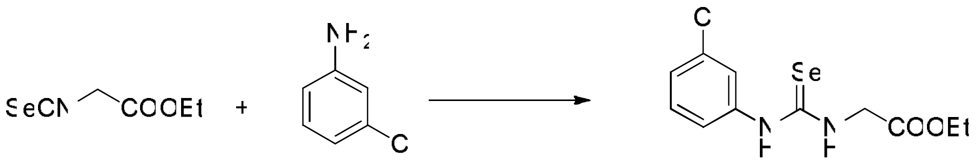

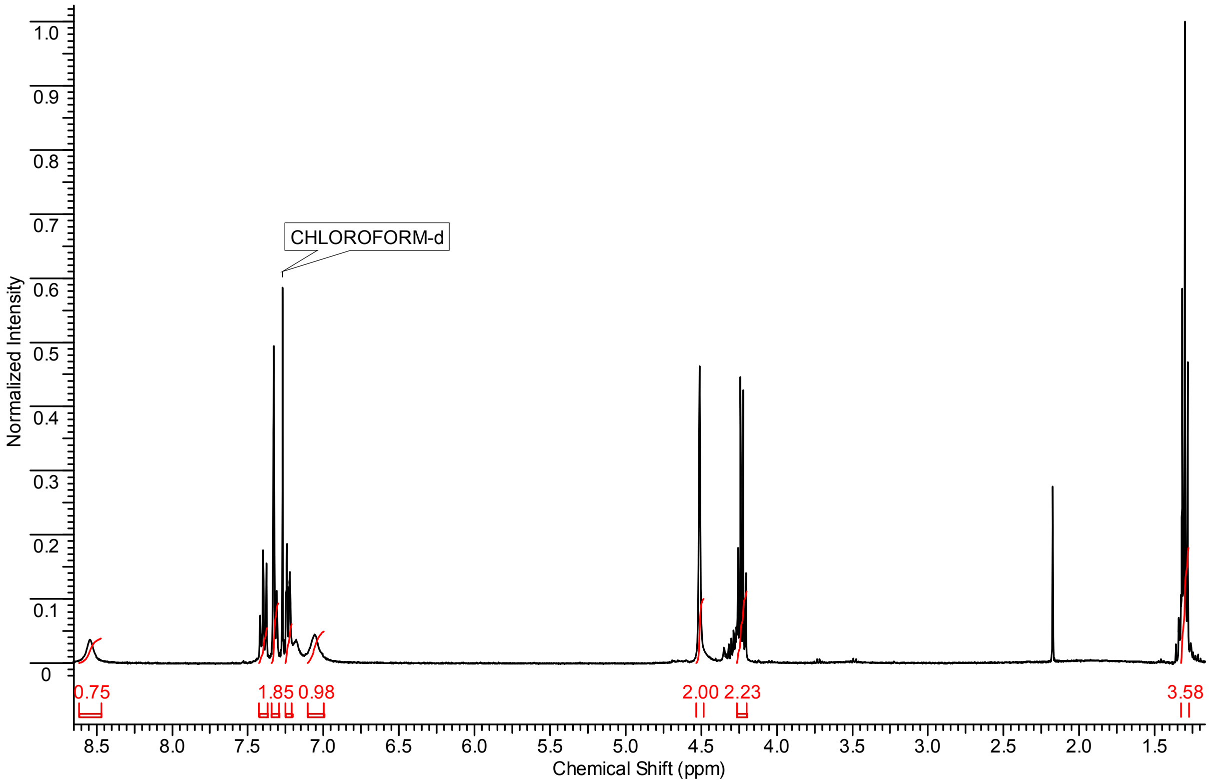

^{1}H NMR (400 MHz, $CDCl_3$, δ, ppm): 8.55 (bs, 1H, NH), 7.40 (t, J=8.6 Hz, 1H, Ar), 7.33 (s, 1H, Ar), 7.33-7.30 (m, 1H, Ar), 7.23 (d, J=8.3 Hz, 1H, Ar), 7.06 (bs, 1H, NH), 4.51 (s, 2H, CH_2), 4.23 (q, J=7.1 Hz, 2H, C(O)O$\underline{CH_2}$$CH_3$), 1.30 (t, J=7.2 Hz, 3H, C(O)O$CH_2$$\underline{CH_3}$).

3-(4-methoxybenzyl)-2-selenoxoimidazolidin-4-one (2g)

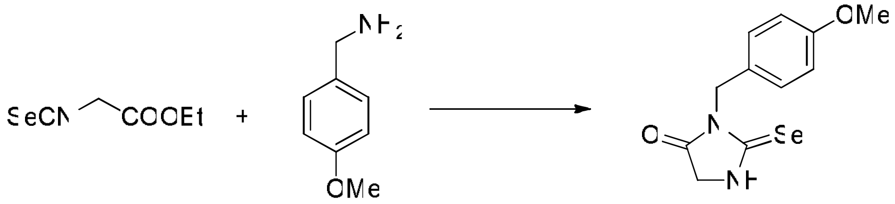

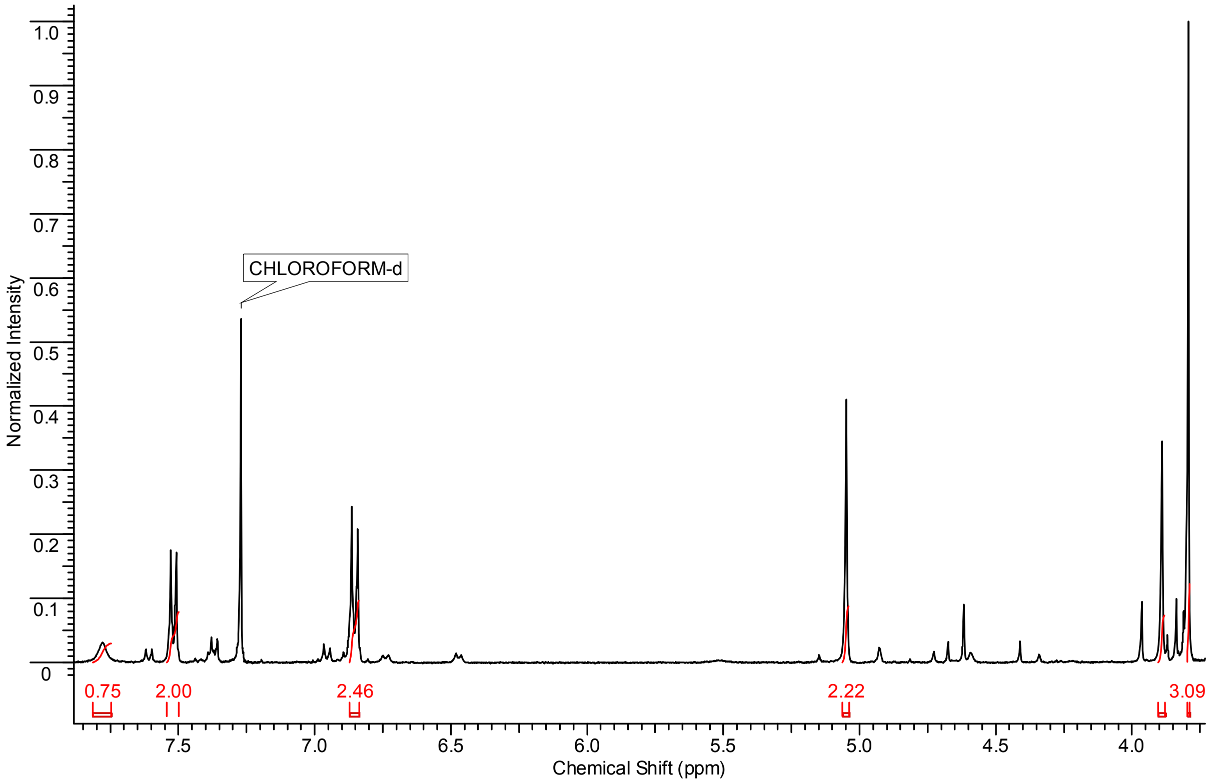

^{1}H NMR (400 MHz, $CDCl_3$, δ, ppm): 7.78 (bs, 1H, NH), 7.52 (d, J=8.7 Hz, 2H, Ar), 6.85 (d, J=8.6 Hz, 2H, Ar), 5.05 (s, 2H, CH_2), 3.89 (s, 2H, CH_2), 3.79 (s, 3H, OCH_3).

5-Arylidene-2-selenoxohydantoins 3a-g (*general procedure*). A 2% solution of KOH in ethanol (1 equivalent of KOH) was added to selenourea (or 2-selenoxohydantoin) **2** (1 equiv.), Then aldehyde (1 equiv.) was added to the solution. The reaction mixture was stirred for 5 hours. Then, a dilute hydrochloric acid solution (H_2O : HCl conc = 9:1) was added to the reaction mixture. The precipitate formed was filtered off, washed with water and diethyl ether. If the residue did not precipitate, the solvent was removed from the reaction mixture and then the product was purified by column chromatography.

(Z)-3-(4-ethoxyphenyl)-5-(4-chlorobenzylidene)-2-selenoxoimidazolidin-4-one (3a)

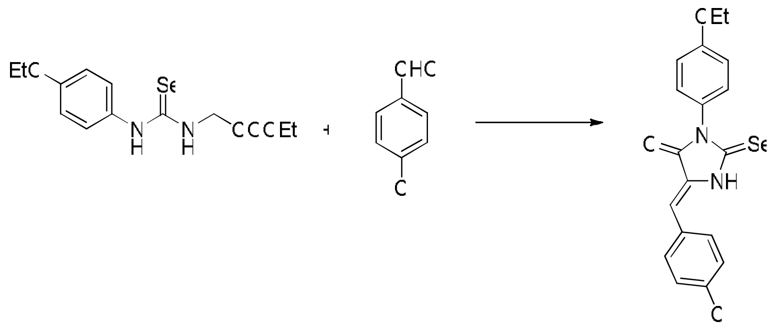

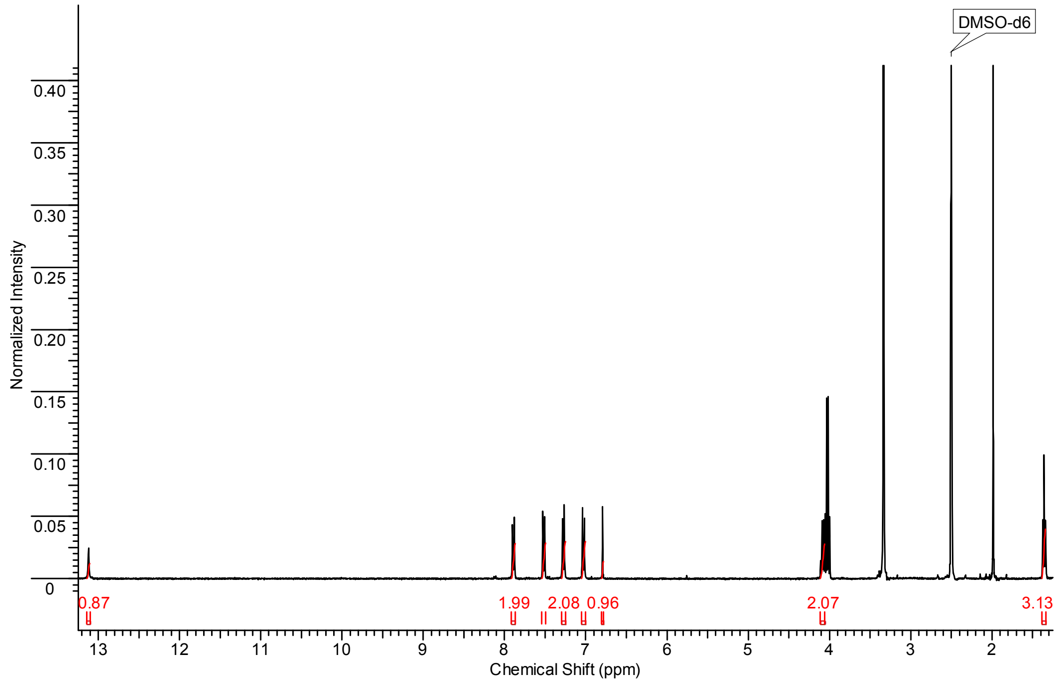

^{1}H NMR (400 MHz, DMSO-d_6, δ, ppm): 13.12 (s, 1H, NH), 7.89 (d, J=8.6 Hz, 2H, Ar), 7.52 (d, J=8.5 Hz, 2H, Ar), 7.27 (d, J=8.9 Hz, 2H, Ar), 7.03 (d, J=8.9 Hz, 2H, Ar), 6.79 (s, 1H, vinyl), 4.08 (q, J=6.9 Hz, 2H, ArO<u>CH$_2$</u>CH$_3$), 1.36 (t, J=6.9 Hz, 3H, ArOCH$_2$<u>CH$_3$</u>).

(Z)-3-(4-methoxyphenyl)-5-(4-chlorobenzylidene)-2-selenoxoimidazolidin-4-one (3b)

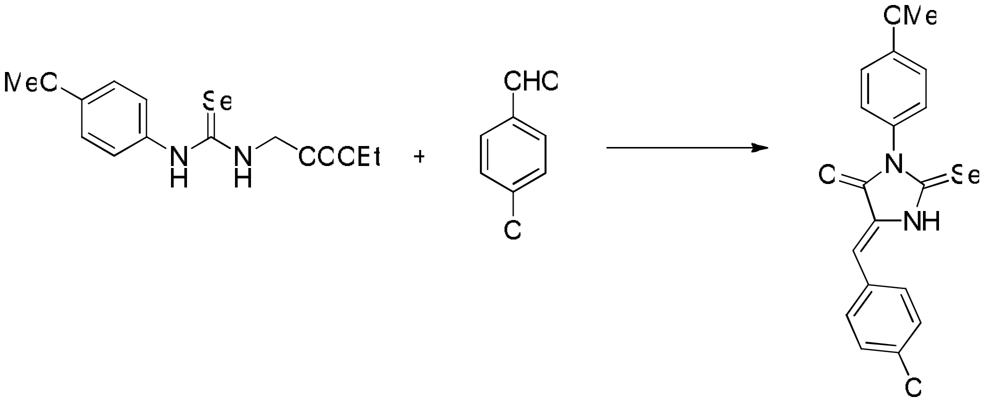

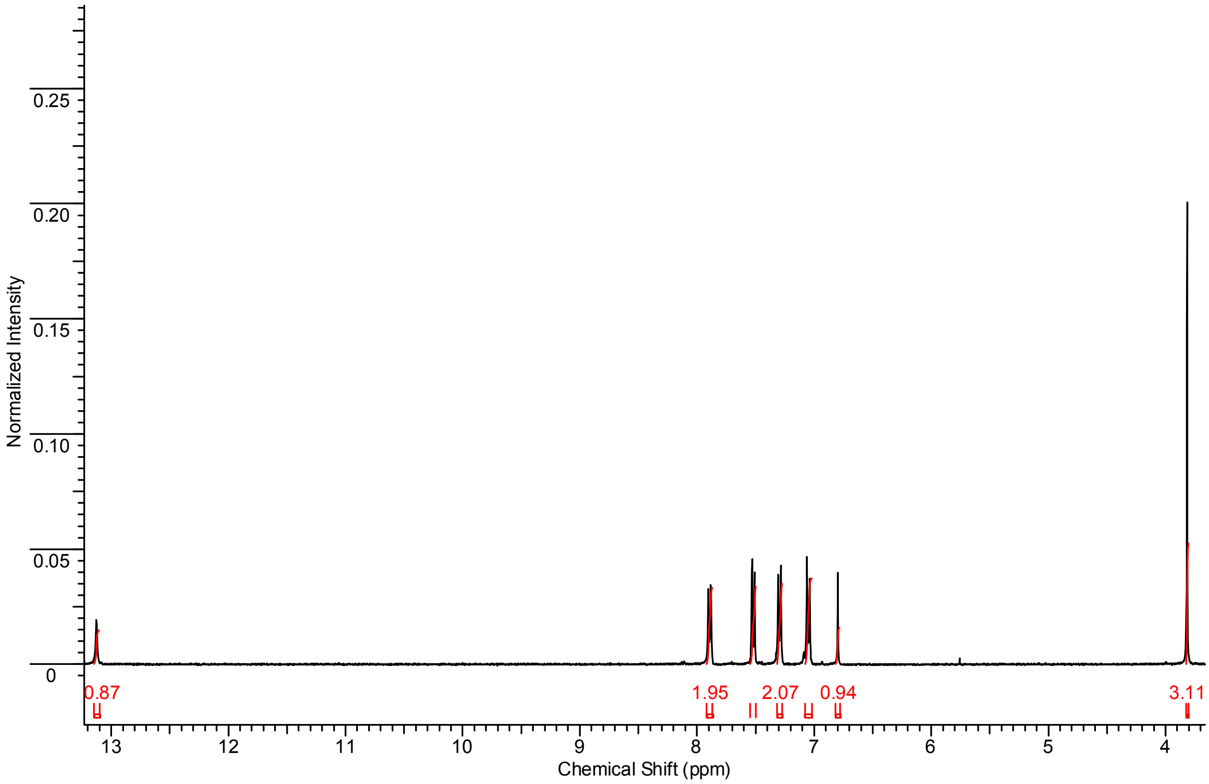

^{1}H NMR (400 MHz, DMSO-d$_6$, δ, ppm): 13.13 (s, 1H, NH), 7.89 (d, J=8.4 Hz, 2H, Ar), 7.52 (d, J=8.5 Hz, 2H, Ar), 7.30 (d, J=8.8 Hz, 2H, Ar), 7.05 (d, J=8.9 Hz, 2H, Ar), 6.80 (s, 1H, vinyl), 3.82 (s, 3H, OCH$_3$).

(Z)-3-(4-methoxyphenyl)-5-(4-methoxybenzylidene)-2-selenoxoimidazolidin-4-one (3c)

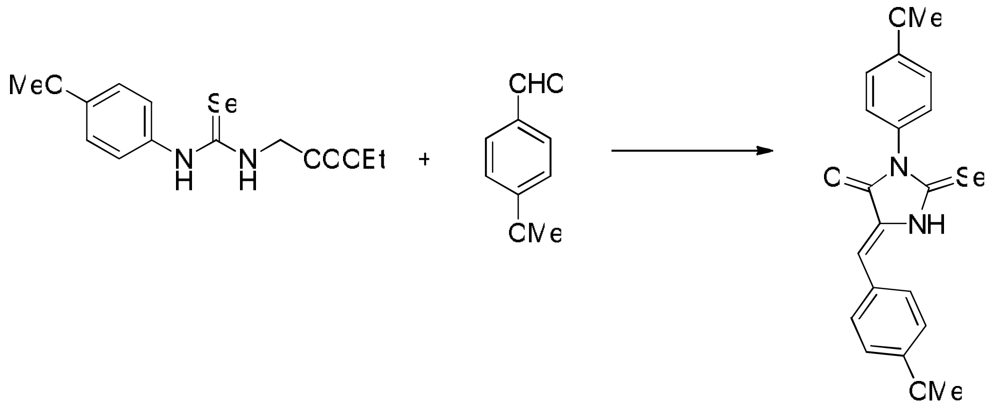

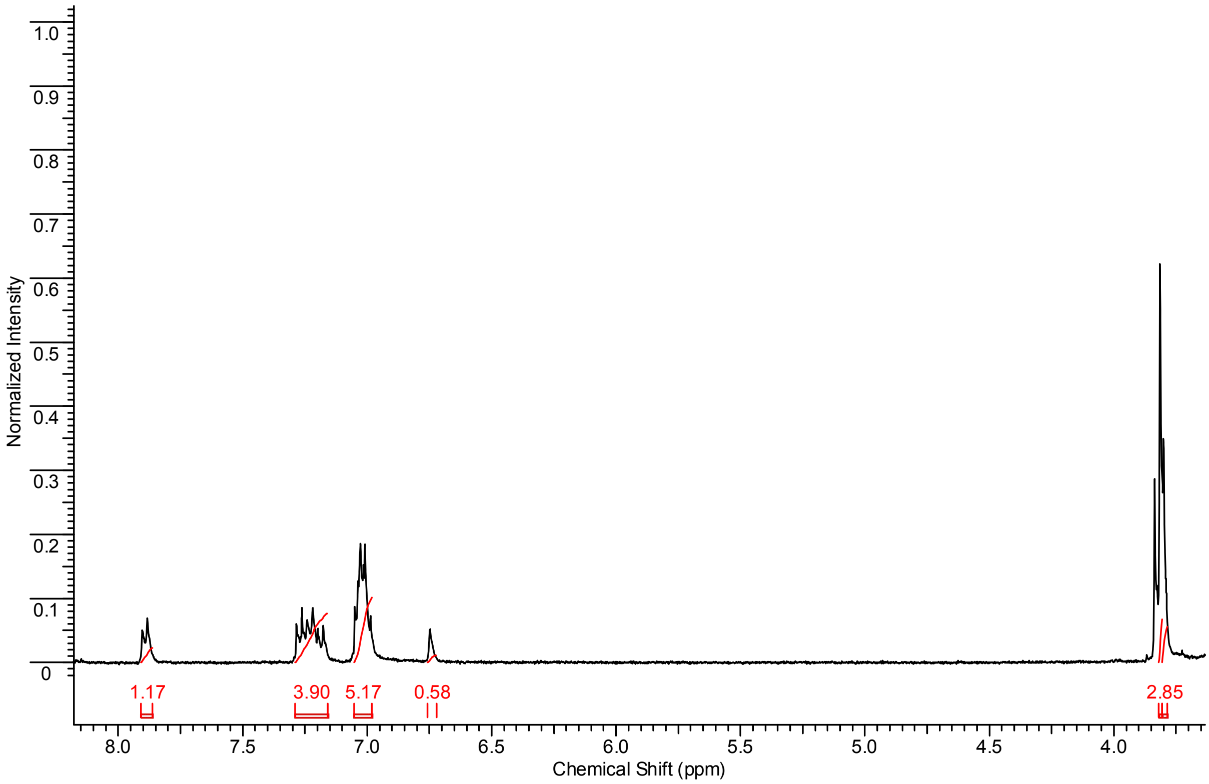

^{1}H NMR (400 MHz, DMSO-d$_6$, δ, ppm):7.88 (s, 1H, NH), 7.29-7.16 (m, 4H, Ar), 7.05-6.98 (m, 4H, Ar), 6.75 (s, 1H, vinyl), 3,81 (s, 3H, OCH_3), 3.80 (s, 3H, OCH_3).

(Z)-3-(4-methoxyphenyl)-5-(4-ethylbenzylidene)-2-selenoxoimidazolidin-4-one (3d)

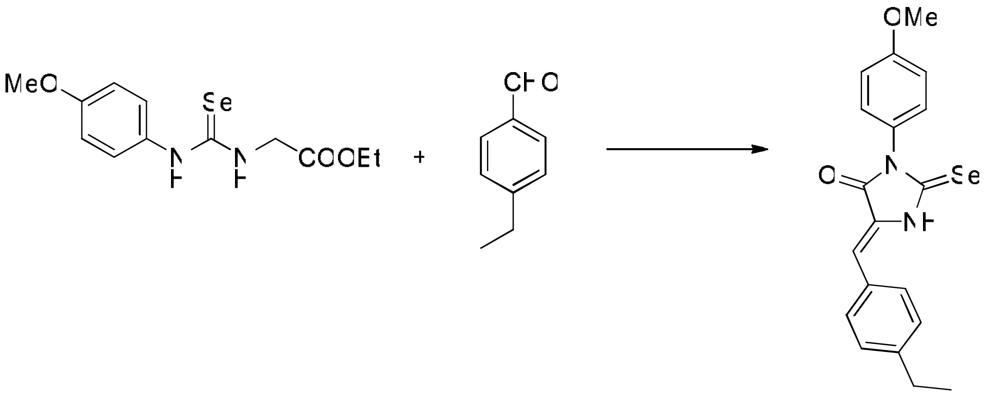

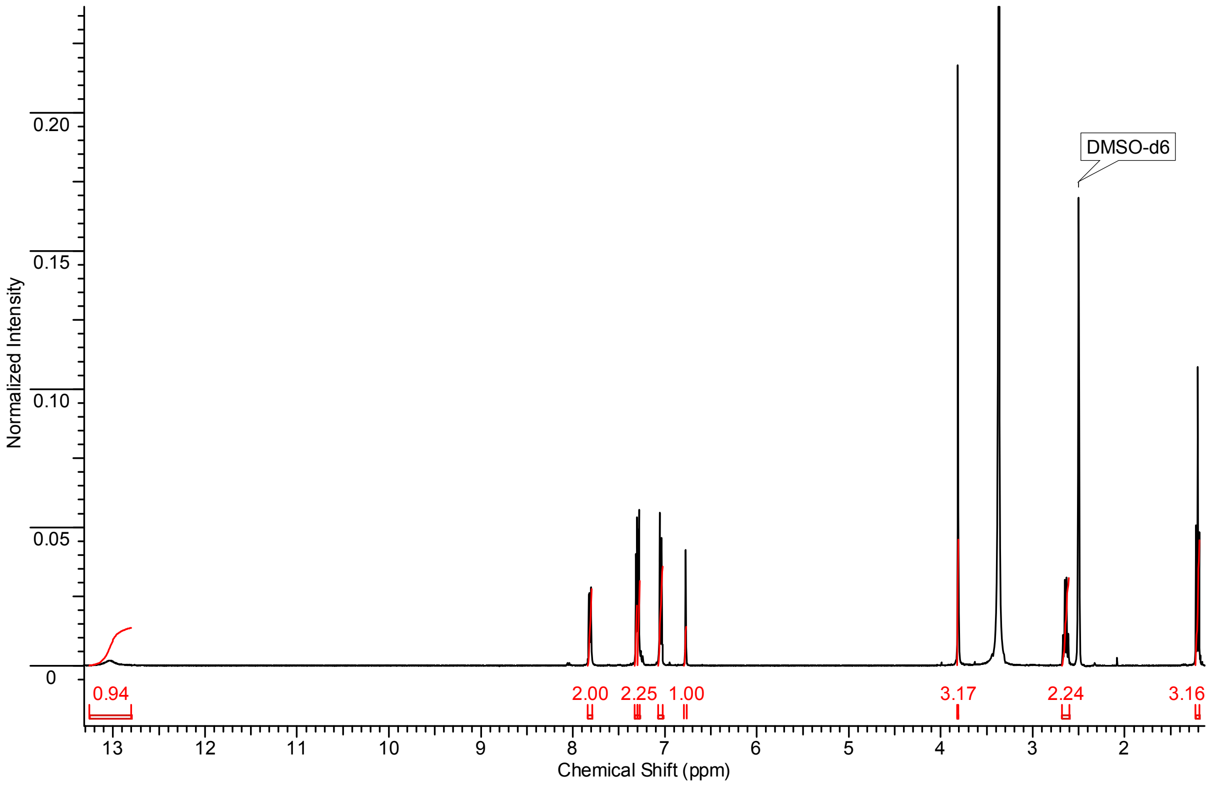

^{1}H NMR (400 MHz, DMSO-d$_6$, δ, ppm): 13.21-12.81 (m, 1H, NH), 7.81 (d, J=8.1 Hz, 2H, Ar), 7.31 (d, J=7.8 Hz, 2H, Ar), 7.29 (d, J=8.9 Hz, 2H, Ar), 7.05 (d, J=9.0 Hz, 2H, Ar), 6.78 (s, 1H, vinyl), 3.81 (s, 1H, OCH_3), 2.64 (q, J=7.6 Hz, 2H, $\underline{CH_2}CH_3$), 1.21 (t, J=7.6 Hz, 3H, $CH_2\underline{CH_3}$).

5-Indolinyliden-2-selenoxohydantoins 4a-m (*general procedure*). A 2% solution of KOH in ethanol (1 equivalent of KOH) was added to selenourea (or 2-selenoxohydantoin) **2**(1 equiv.), then isatin (1 equiv.) was added to the solution. The reaction mixture was stirred for 1 hour. Then, a dilute hydrochloric acid solution (H_2O : HCl conc = 9:1) was added to the reaction mixture. The precipitate formed was filtered off, washed with water and diethyl ether. If the precipitate did not precipitated, the product is isolated by column chromatography.

(Z)-3-(3-(4-ethoxyphenyl)-4-oxo-2-selenoxoimidazolidin-5-ylidene)indolin-2-one (4a)

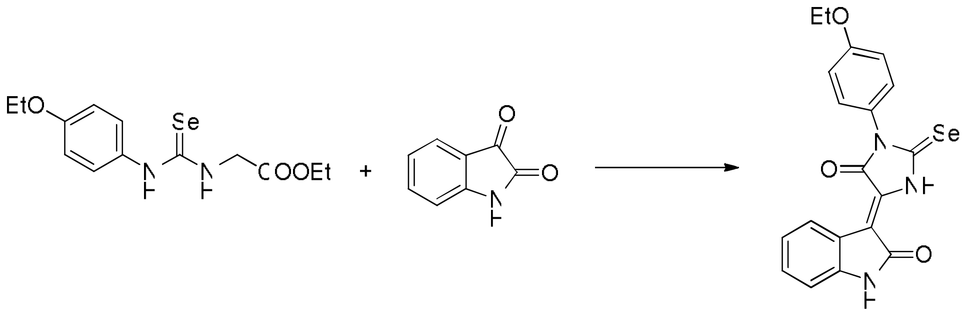

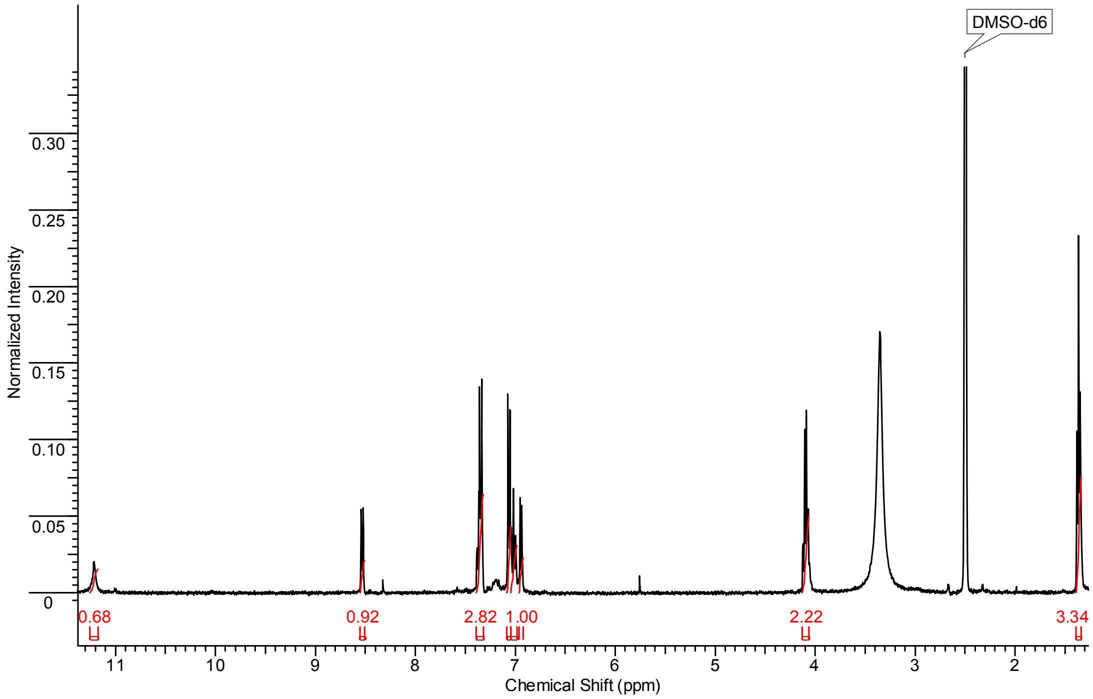

^{1}H NMR (400 MHz, DMSO-d_6, δ, ppm): 12.07-11.66 (m, 1H, NH), 11.21 (bs, 1H, NH), 8.53 (d, J=7.7Hz, 1H, isatin), 7.39-7.33 (m, 1H, isatin), 7.35 (d, J=8.9Hz, 2H, Ar), 7.06 (d, J=8.9Hz, 2H, Ar), 7.02 (t, J=8.2Hz, 1H, isatin), 6.94 (d, J=7.8Hz, 1H, isatin), 4.09 (q, J=6.9Hz, 2H, ArO<u>CH$_2$</u>CH$_3$), 1.36 (t, J=6.9 Hz, 3H, ArOCH$_2$<u>CH$_3$</u>).

(Z)-3-(3-(4-ethoxyphenyl)-4-oxo-2-selenoxoimidazolidin-5-ylidene)-5-chloroindolin-2-one (4b)

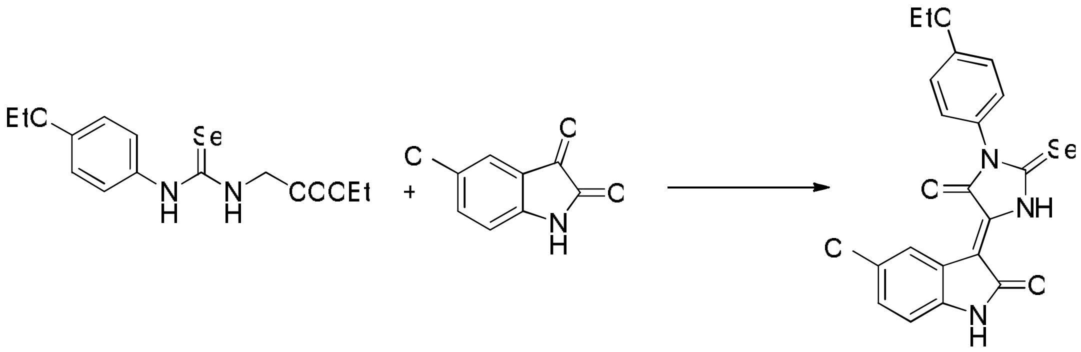

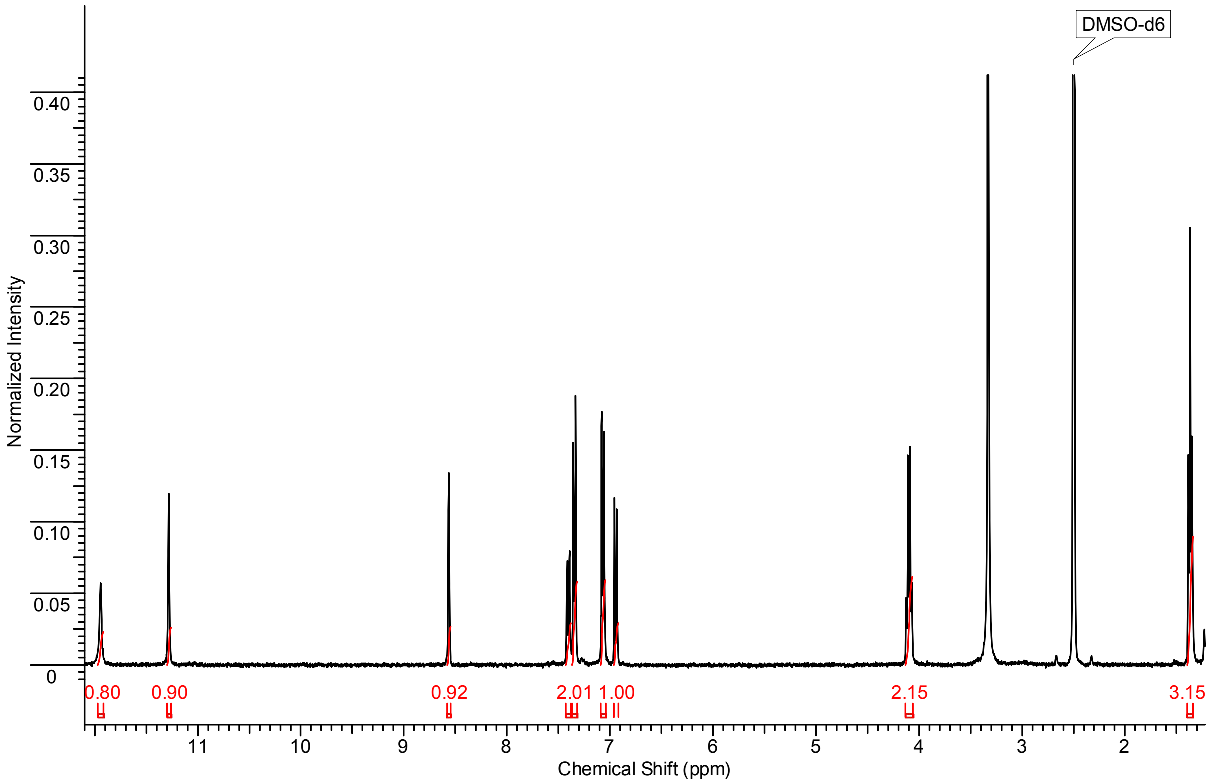

^{1}H NMR (400 MHz, DMSO-d6, δ, ppm): 11.95 (bs, 1H, NH), 11.28 (s, 1H, NH), 8.57 (d, J=1.9 Hz, 1H, isatin), 7.40 (dd, J_1=2.0 Hz, J_2=8.3 Hz, 1H, isatin), 7.34 (d, J=8.8 Hz, 2H, Ar), 7.07 (d, J=8.8 Hz, 2H, Ar), 6.94 (d, J=8.4 Hz, 1H, isatin), 4.10 (q, J=6.9 Hz, 2H, ArO<u>CH_2</u>CH_3), 1.37 (t, J=6.9 Hz, 3H, ArOCH_2<u>CH_3</u>).

(Z)-3-(3-(4-ethoxyphenyl)-4-oxo-2-selenoxoimidazolidin-5-ylidene)-5-bromoindolin-2-one (4c)

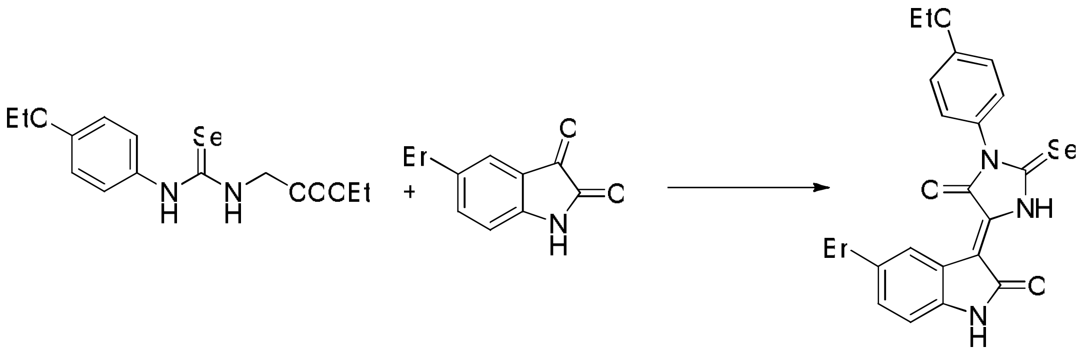

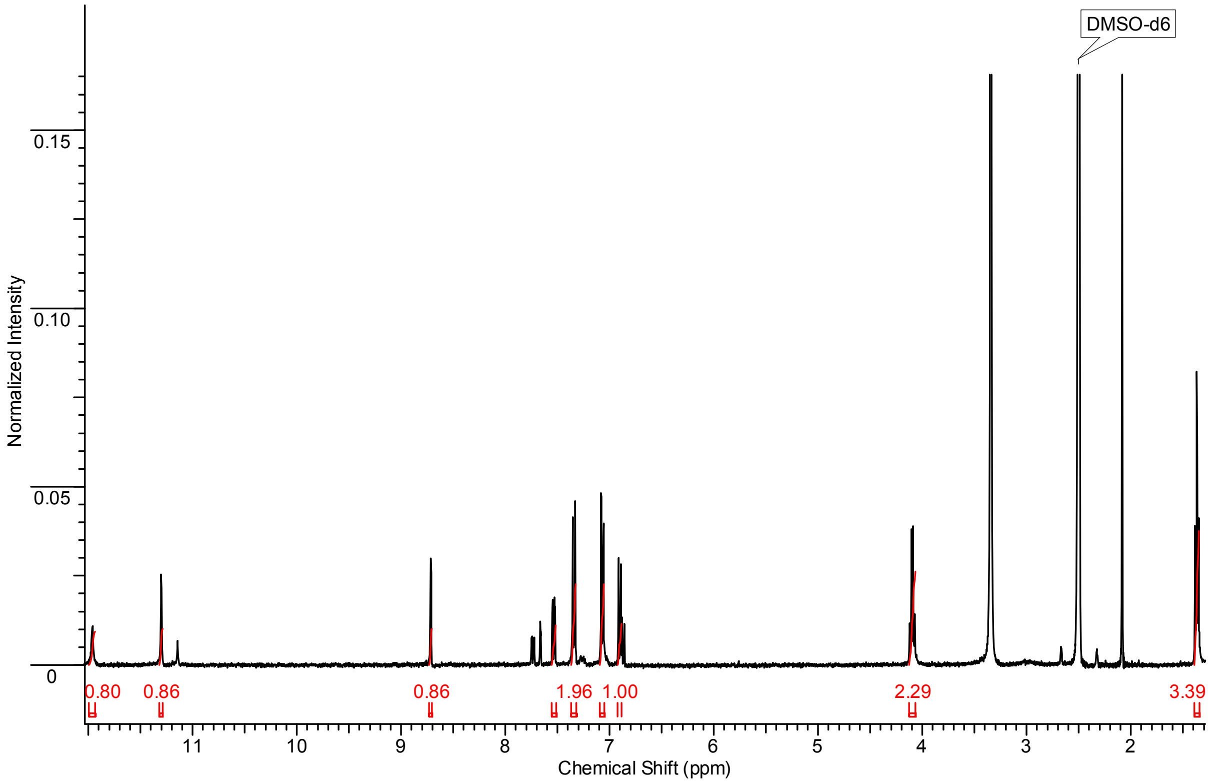

^{1}H NMR (400 MHz, DMSO-d6, δ, ppm): 11.96 (s, 1H, NH), 11.30 (s, 1H, NH), 8.71 (d, J=1.9 Hz, 1H, isatin), 7.53 (dd, J_1=2.0 Hz, J_2=8.4 Hz, 1H, isatin), 7.34 (d, J=8.9 Hz, 2H, Ar), 7.07 (d, J=9.9 Hz, 2H, Ar), 6.90 (d, J=8.3, 1H, isatin), 4.10 (q, J=7.0 Hz, 2H, ArO$\underline{CH_2}$$CH_3$), 1.37 (t, J=7.0 Hz, 3H, Ar$OCH_2$$\underline{CH_3}$).

(Z)-3-(3-(4-ethoxyphenyl)-4-oxo-2-selenoxoimidazolidin-5-ylidene)-5-nitroindolin-2-one (4d)

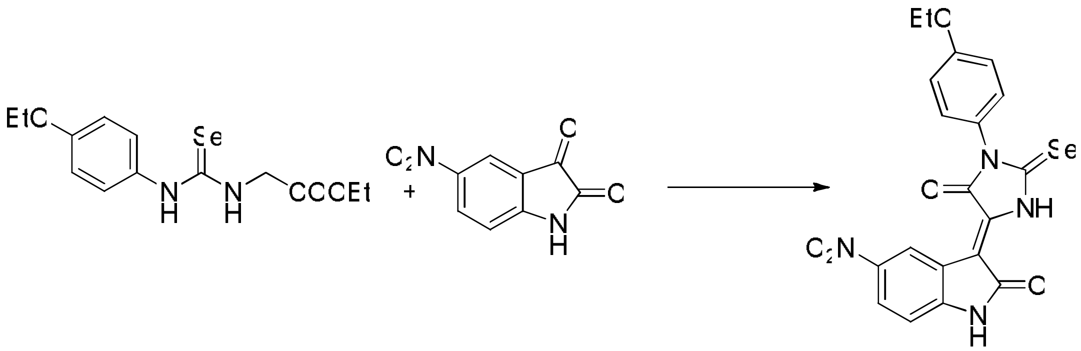

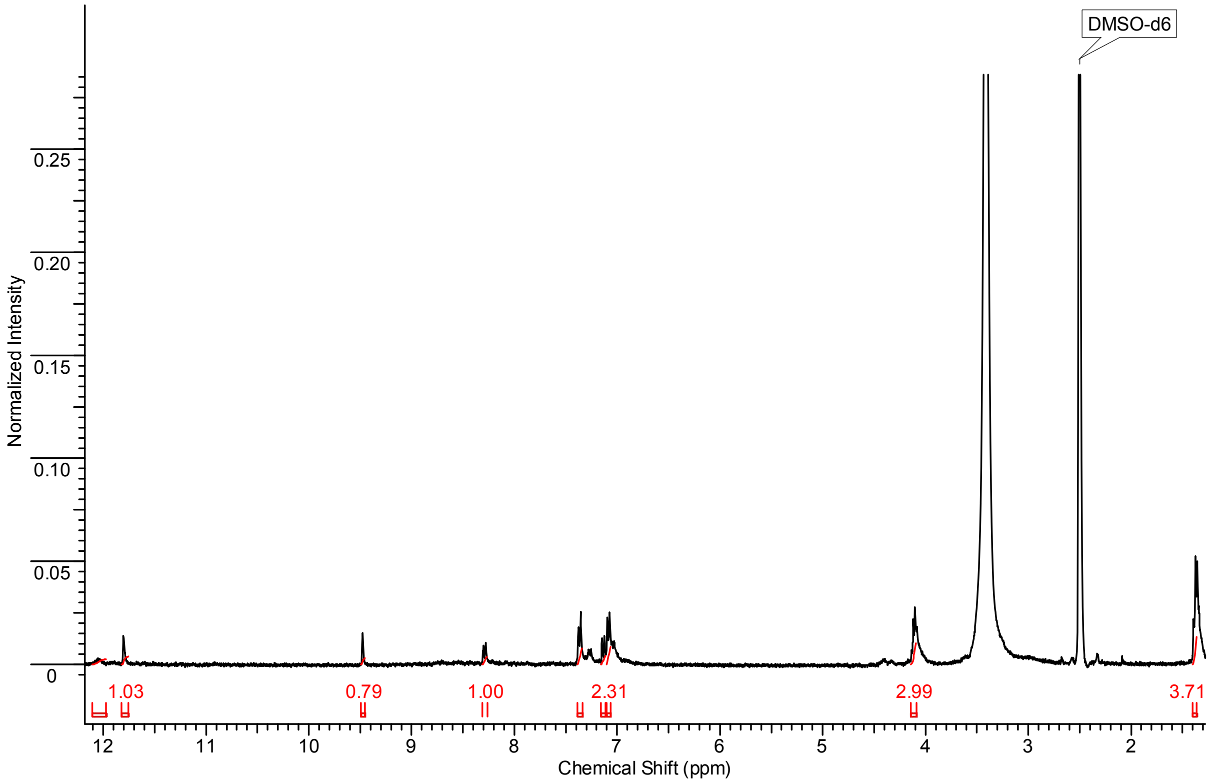

^{1}H NMR (400 MHz, DMSO-d6, δ, ppm): 12.11 (m, 1H, NH), 11.80 (s, 1H, NH), 9.48 (s, 1H, isatin), 8.29 (dd, J_1=2.0Hz, J_2=8.8Hz, 1H, isatin), 7.37 (d, J=8.8Hz, 2H, Ar), 7.14 (d, J=8.8Hz, 1H, isatin), 7.08 (d, J=8.8Hz, 2H, Ar). 4.11 (q, J=6.9Hz, 2H, ArO<u>CH$_2$</u>CH$_3$), 1.37 (t, J=6.9Hz, 3H, ArOCH$_2$<u>CH$_3$</u>).

(Z)-3-(3-(4-methoxyphenyl)-4-oxo-2-selenoxoimidazolidin-5-ylidene)-5-bromoindolin-2-one (4e)

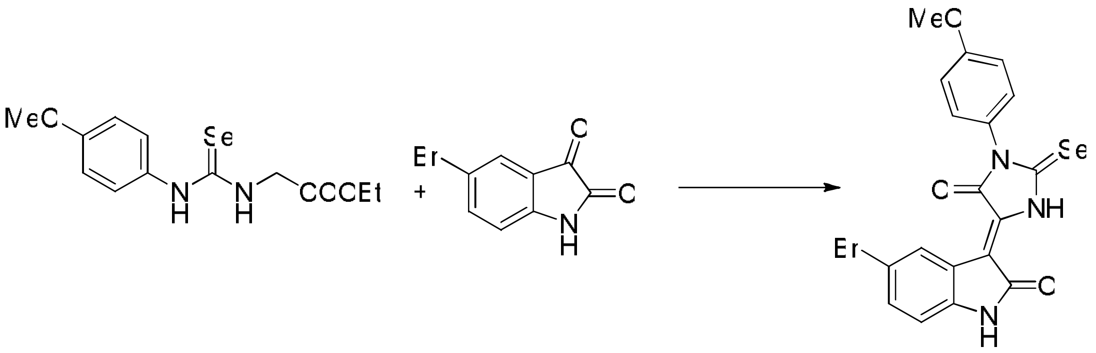

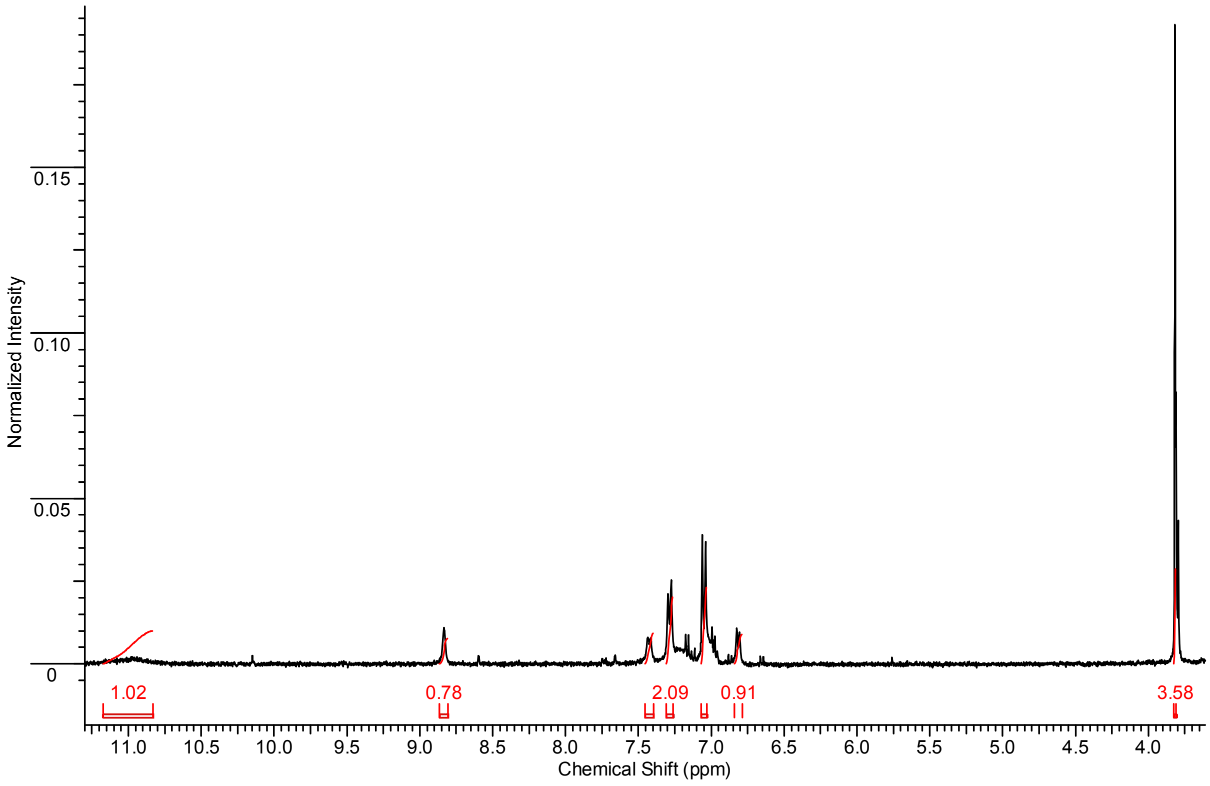

^{1}H NMR (400 MHz, DMSO-d6, δ, ppm): 10.98 (bs, 1H, NH), 8.83 (s, 1H, isatin), 7.43 (d, J=7.6 Hz, 1H, isatin), 7.29 (d, J=8.6 Hz, 2H, Ar), 7.05 (d, J=8.7 Hz, 2H, Ar), 6.81 (d, J=8.5 Hz, 1H, isatin), 3.82 (s, 3H, OCH_3).

(Z)-3-(3-(4-methoxyphenyl)-4-oxo-2-selenoxoimidazolidin-5-ylidene)-5-nitroindolin-2-one (4f)

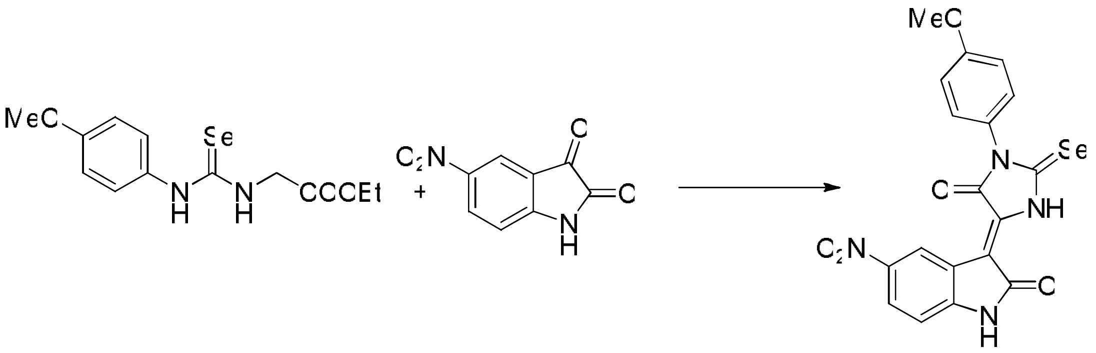

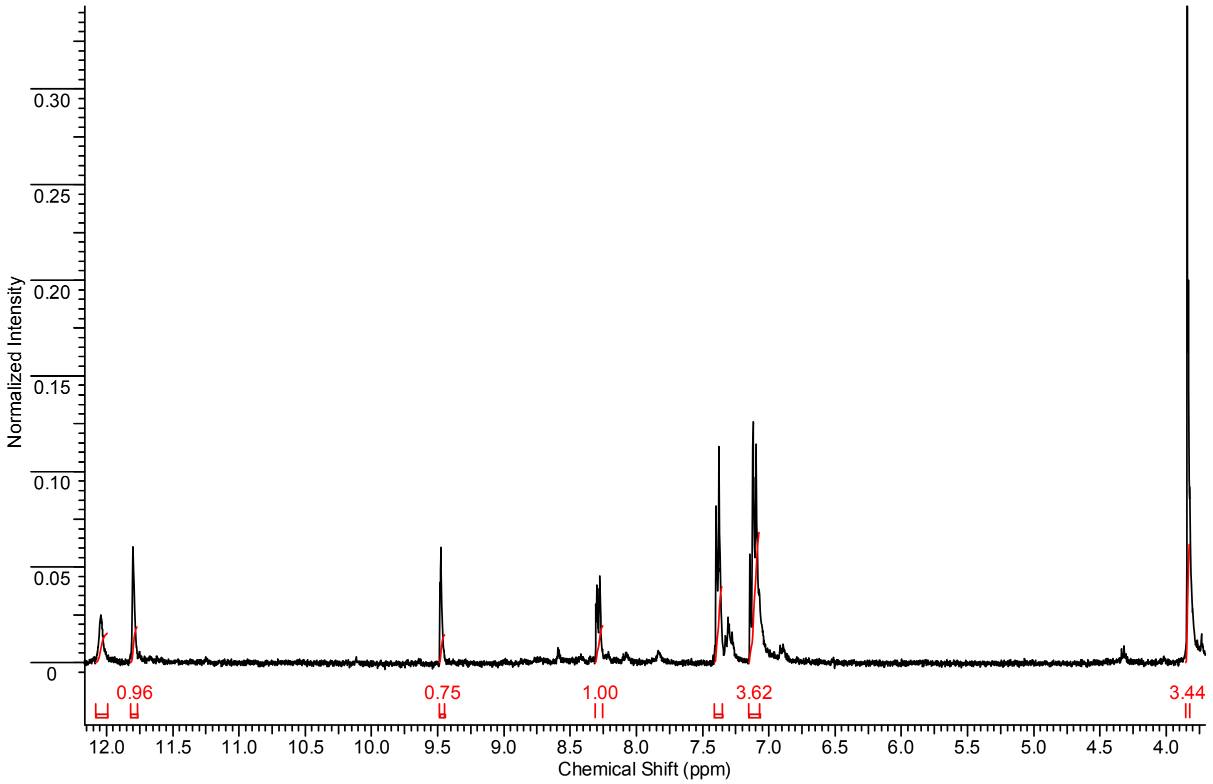

^{1}H NMR (400 MHz, DMSO-d6, δ, ppm):12.04 (bs, 1H, NH), 11.80 (s, 1H, NH), 9.48 (d, J=2.3Hz, 1H, isatin), 8.29 (dd, J_1=2.4Hz, J_2=8.7Hz, 1H, isatin), 7.39 (d, J=8.8Hz, 2H, Ar), 7.15-7.12 (m, 1H, isatin), 7.11 (d, J=9.1Hz, 2H, Ar), 3.84 (s, 3H, OCH_3).

(Z)-3-(3-(4-chlorophenyl)-4-oxo-2-selenoxoimidazolidin-5-ylidene)-5-bromoindolin-2-one (4g)

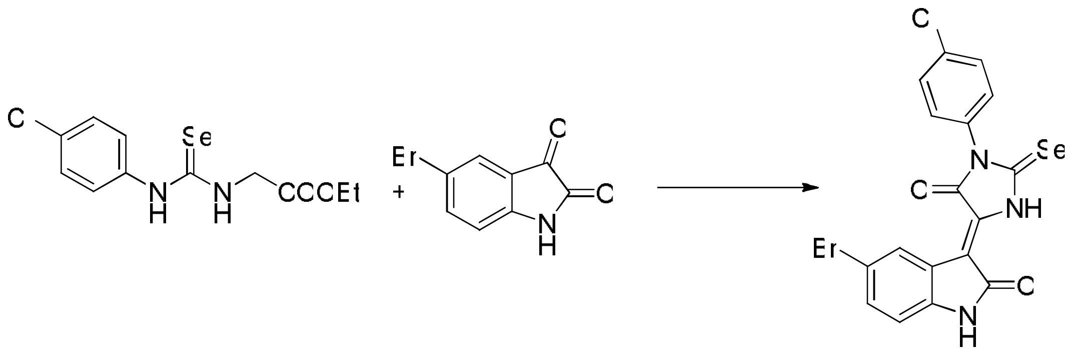

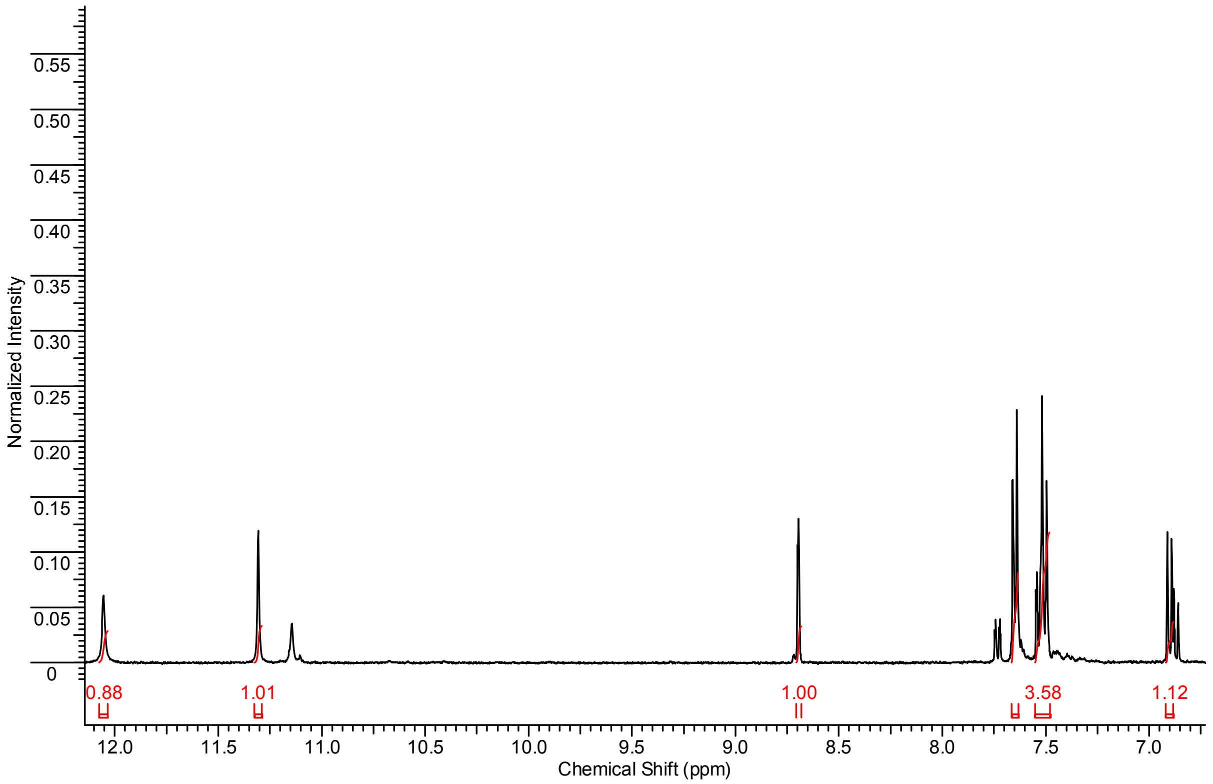

^{1}H NMR (400 MHz, DMSO-d6, δ, ppm): 12.06 (s, 1H, NH), 11.31 (s, 1H, NH), 8.70 (d, J=1.7 Hz, 1H, isatin), 7.65 (d, J=8.6 Hz, 2H, Ar), 7.55-7.52 (m, 1H, isatin), 7.51 (d, J=8.7 Hz, 2H, Ar), 6.90 (d, J=8.3 Hz, 1H, isatin).

(Z)-3-(3-(4-chlorophenyl)-4-oxo-2-selenoxoimidazolidin-5-ylidene)-5-nitroindolin-2-one (4h)

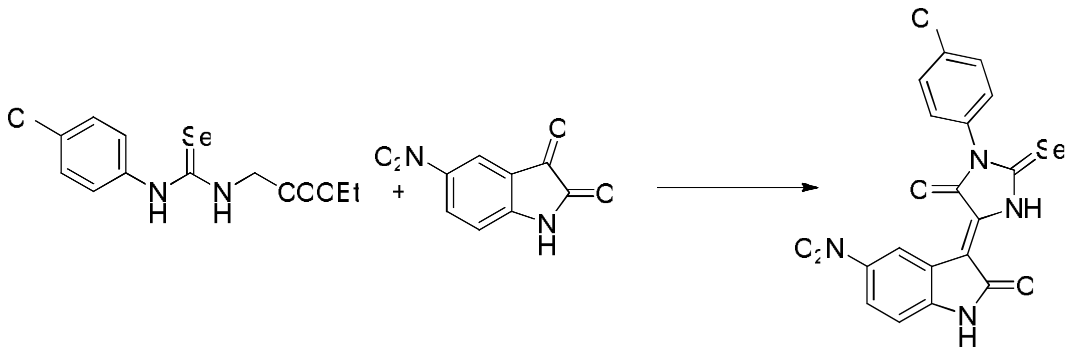

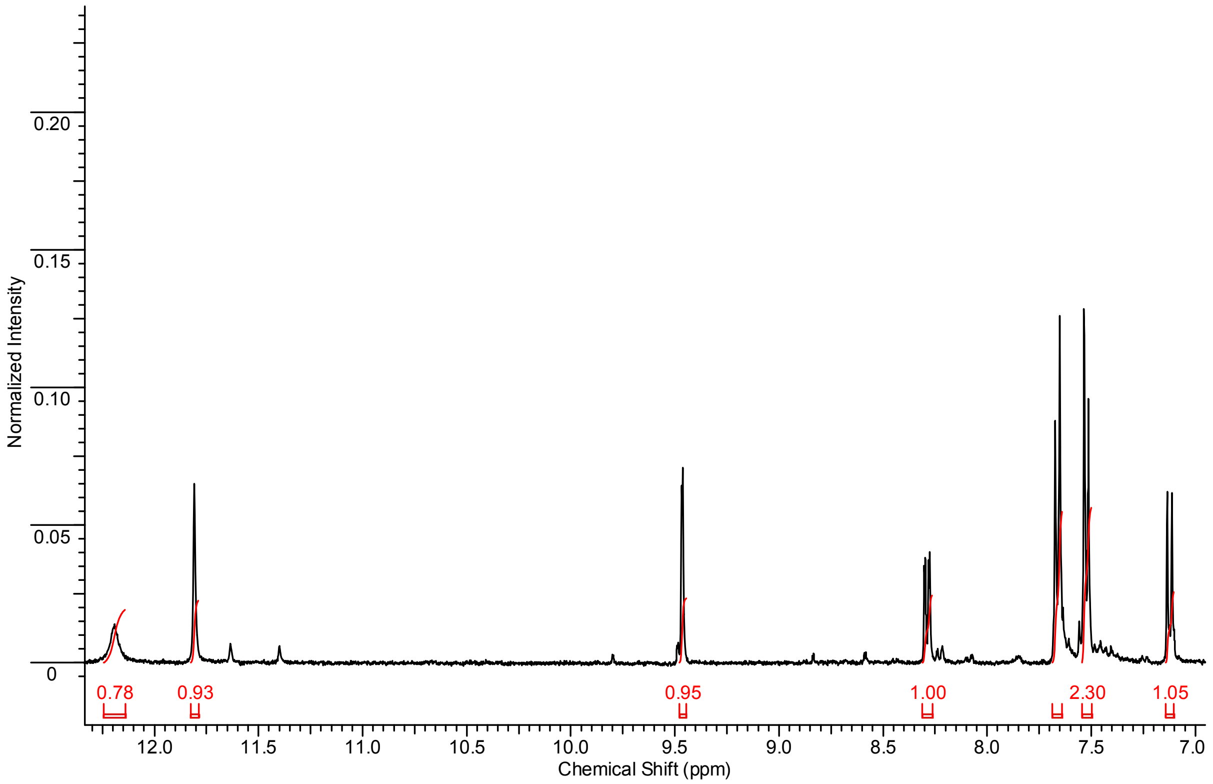

^{1}H NMR (400 MHz, DMSO-d6, δ, ppm):12.19 (bs, 1H, NH), 11.81 (s, 1H, NH), 9.46 (d, J=2.2Hz, 1H, isatin), 8.29 (dd, J_1=2.3Hz, J_2=8.7Hz, 1H, isatin), 7.66 (d, J=8.6Hz, 2H, Ar), 7.52 (d, J=8.6Hz, 2H, Ar), 7.12 (d, J=8.7Hz, 1H, isatin).

(Z)-3-(3-(3-chlorophenyl)-4-oxo-2-selenoxoimidazolidin-5-ylidene)indolin-2-one (4i)

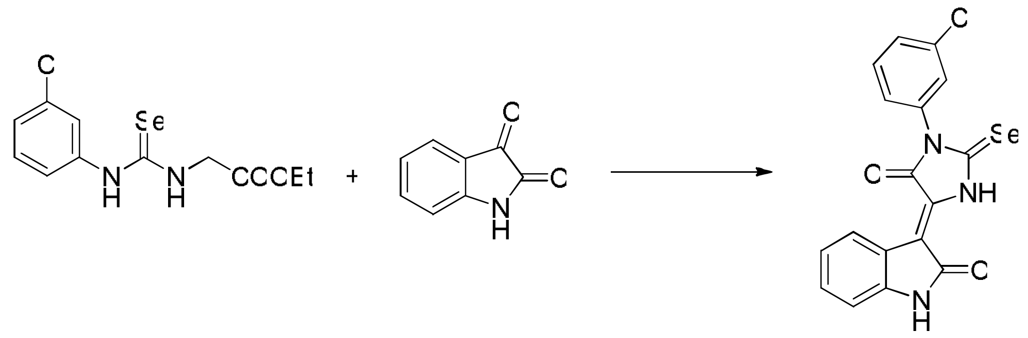

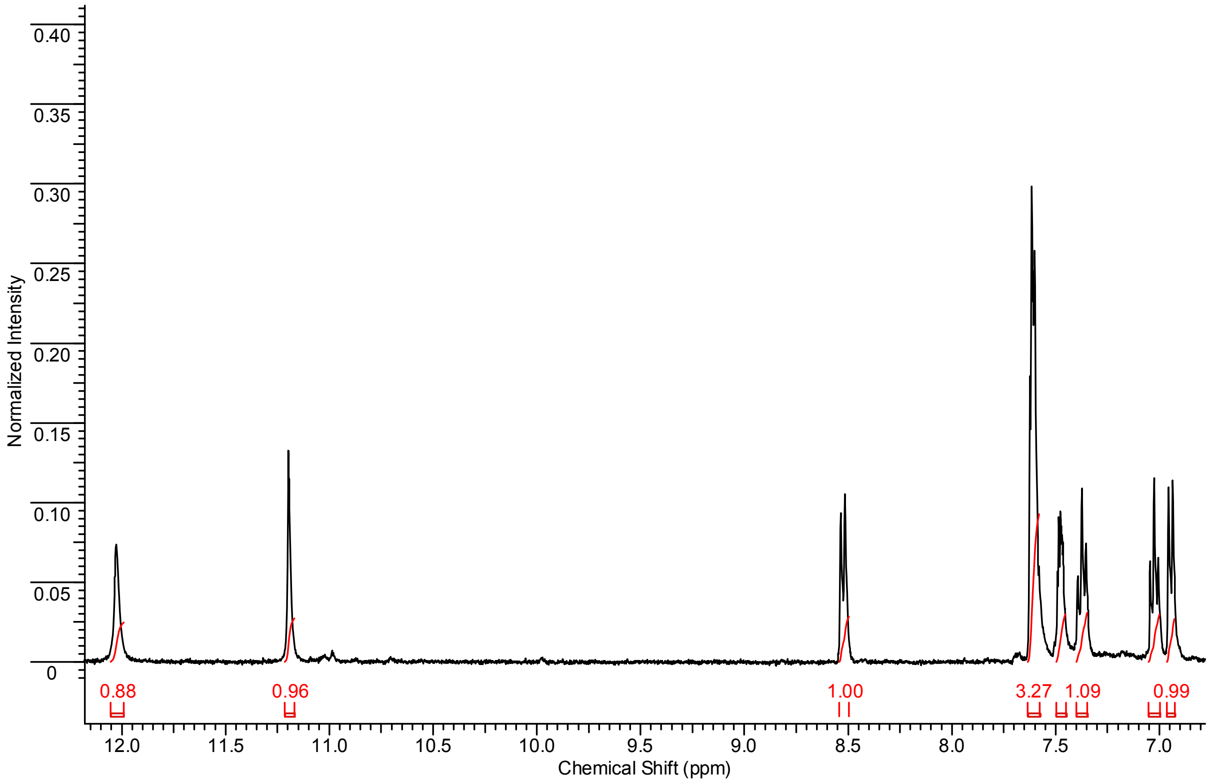

^{1}H NMR (400 MHz, DMSO-d6, δ, ppm): 12.03 (s, 1H, NH), 11.20 (s, 1H, NH), 8.53 (d, J=7.7 Hz, 1H, isatin), 7.64-7.57 (m, 3H, Ar), 7.50-7.45 (m, 1H, Ar), 7.37 (t, J=7.7 Hz, 1H, isatin), 7.02 (t, J=7.7 Hz, 1H, isatin), 6.95 (d, J=7.8 Hz, 1H, isatin).

(Z)-3-(3-(3-chloro-4-fluorophenyl)-4-oxo-2-selenoxoimidazolidin-5-ylidene)-5-chloroindolin-2-one (4j)

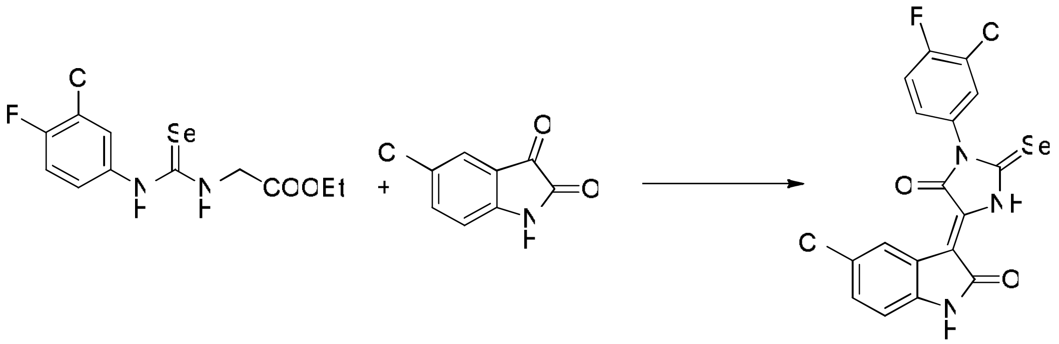

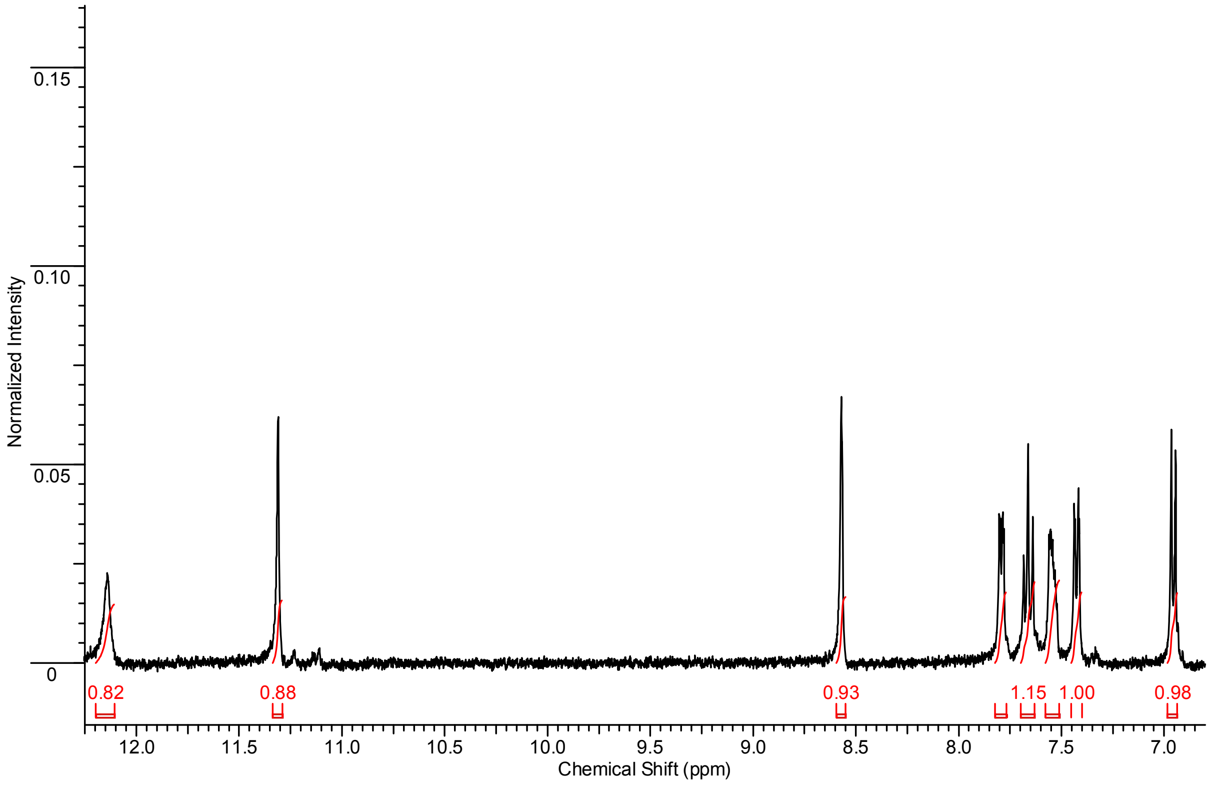

^{1}H NMR (400 MHz, DMSO-d6, δ, ppm): 12.14 (s, 1H, NH), 11.31 (s, 1H, NH), 8.57 (d, J=1.8 Hz, 1H, Ar), 7.79 (dd, J_1=2.3 Hz, J_2=6.9 Hz, 1H, Ar), 7.66 (t, J=8.9 Hz, 1H, Ar), 7.58-7.51 (m, 1H, Ar), 7.43 (dd, J_1=2.1 Hz, J_2=8.3 Hz, 1H, Ar), 6.96 (d, J=8.3 Hz, 1H, Ar).

(Z)-3-(3-cyclopropyl-4-oxo-2-selenoxoimidazolidin-5-ylidene)-5-bromoindolin-2-one (4k)

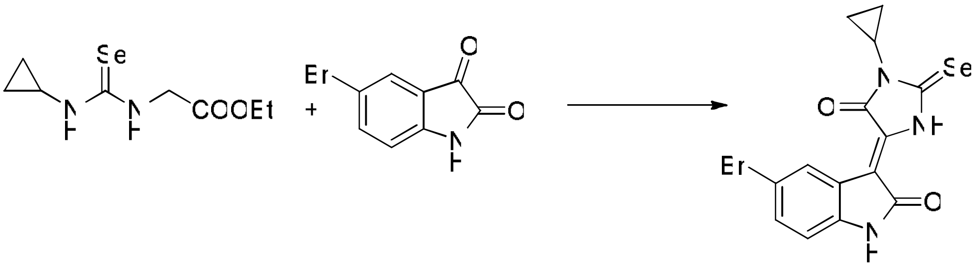

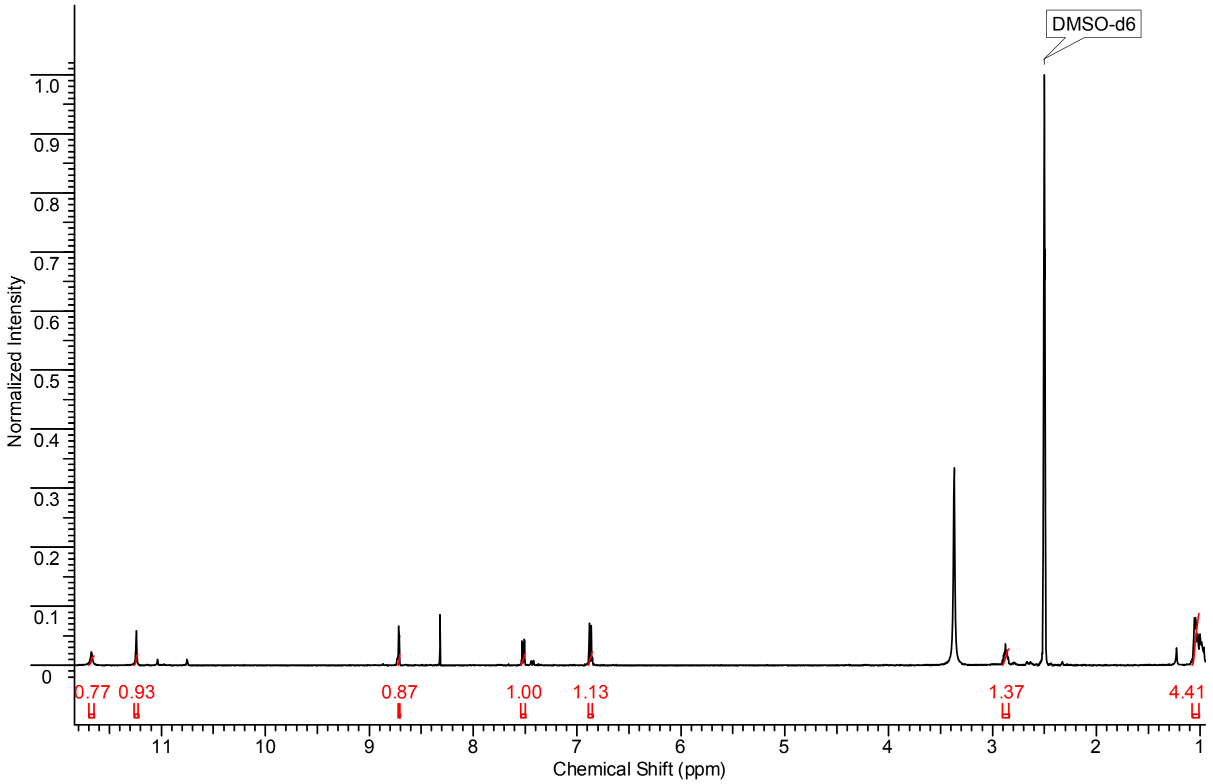

^{1}H NMR (400 MHz, DMSO-d6, δ, ppm): 11.67 (s, 1H, NH), 11.24 (s, 1H, NH), 8.71 (d, J=2.0 Hz, 1H, isatin), 7.52 (dd, J_1=2.0 Hz, J_2=8.3 Hz, 1H, isatin), 6.87 (d, J=8.3 Hz, 1H, isatin), 2.90-2.84 (m, 1H, Pr), 1.08-1.01 (m, 4H, Pr).

(Z)-3-(3-(4-methoxybenzyl)-4-oxo-2-selenoxoimidazolidin-5-ylidene)indolin-2-one (4l)

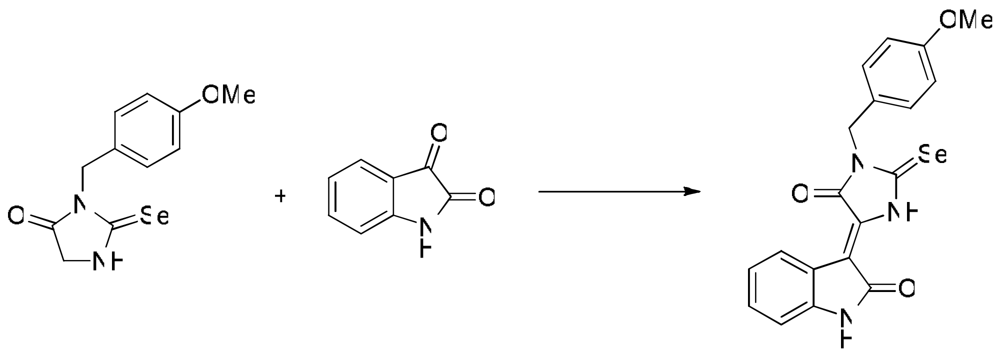

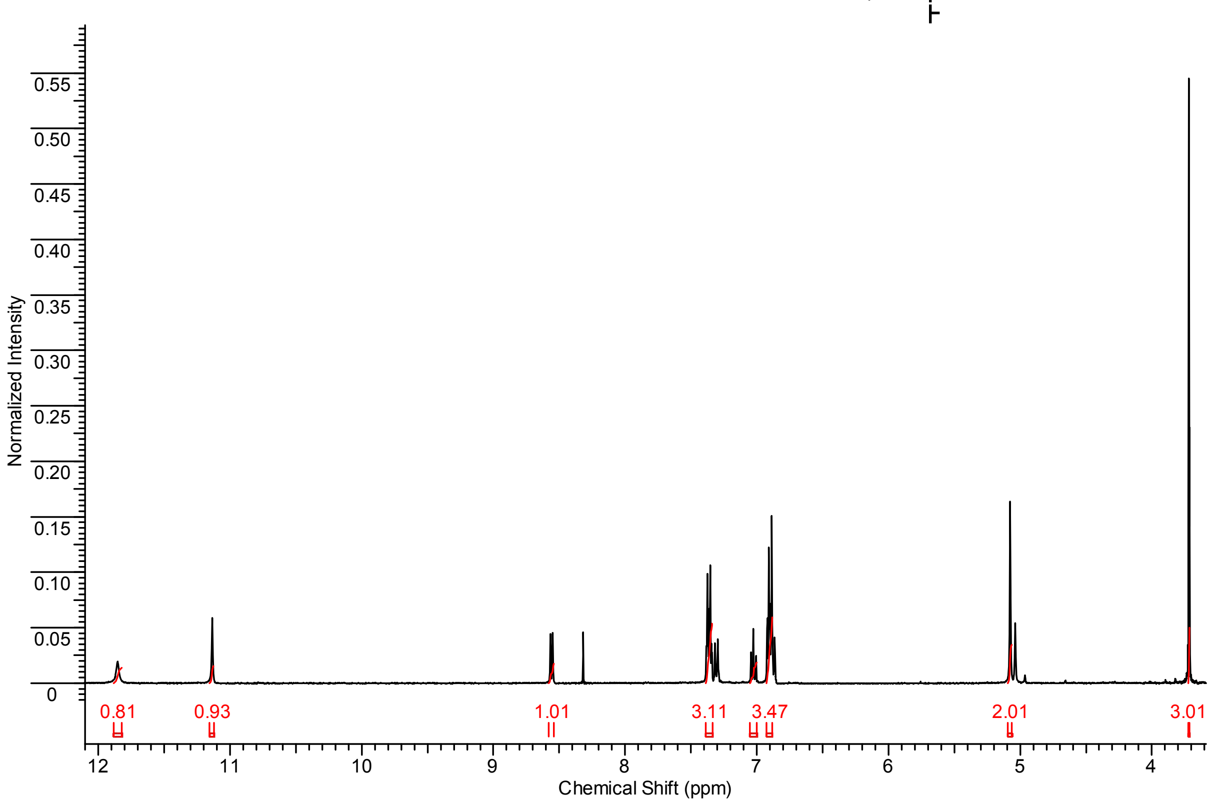

^{1}H NMR (400 MHz, DMSO-d6, δ, ppm):11.85 (bs, 1H, NH), 11.14 (s, 1H, NH), 8.56 (d, J=7.8Hz, 1H, isatin), 7.39-7.34 (m, 1H, isatin), 7.36 (d, J=8.7Hz, 2H, Ar), 7.03 (t, J=7.6Hz, 1H, isatin), 6.91 (d, J=7.1Hz, 1H, isatin), 6.90 (d, J=8.8Hz, 2H, Ar), 5.08 (s, 2H, CH_2), 3.72 (s, 3H, OCH_3).

(Z)-3-(3-(4-methoxybenzyl)-4-oxo-2-selenoxoimidazolidin-5-ylidene)-5-bromoindolin-2-one (4m)

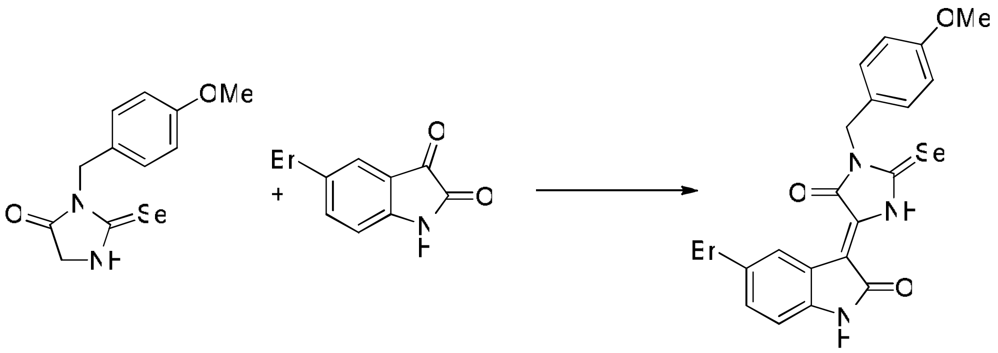

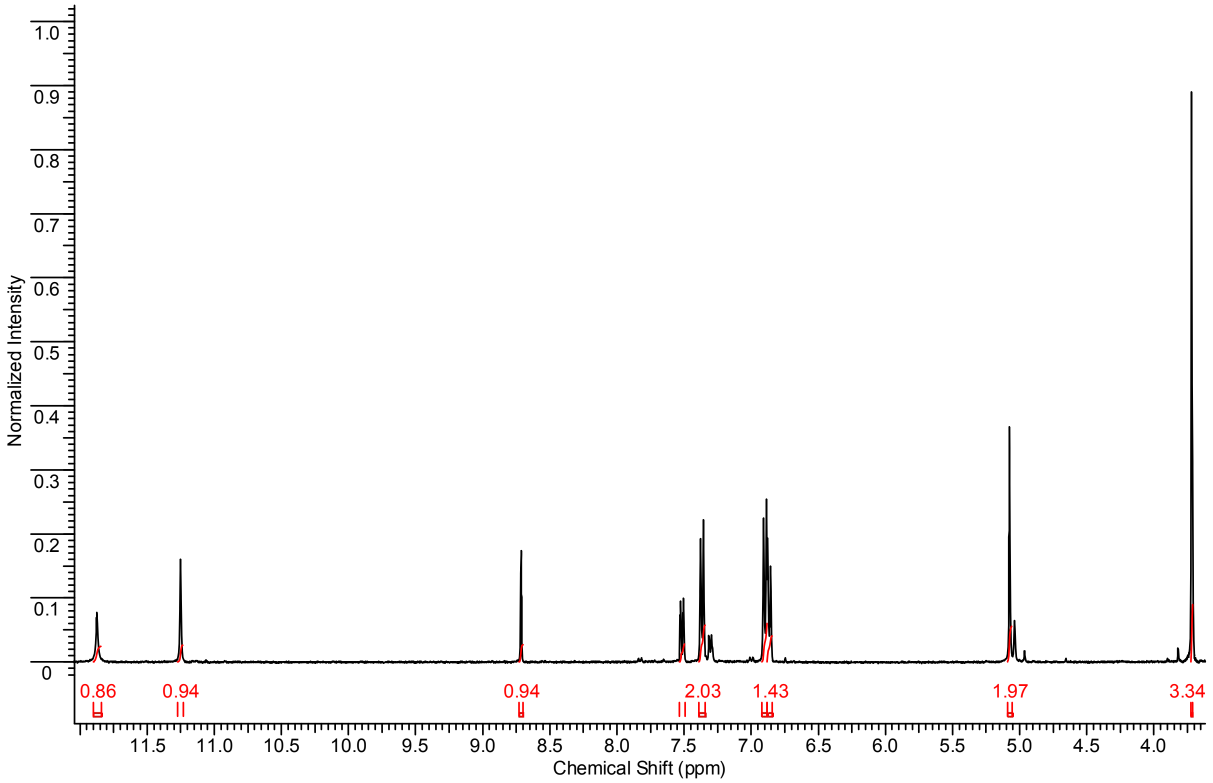

^{1}H NMR (400 MHz, DMSO-d6, δ, ppm): 11.87 (s, 1H, NH), 11.25 (s, 1H, NH), 8.71 (s, 1H, isatin), 7.51 (dd, J_1=1.4 Hz, J_2=8.4 Hz, 1H, isatin), 7.37 (d, J=8.5 Hz, 2H, Ar), 6.90 (d, J=8.7 Hz, 2H, Ar), 6.87 (d, J=8.5 Hz, 1H, isatin), 5.08 (s, 2H, CH_2), 3.72 (s, 3H, OCH_3).

Synthesis of dispiroindolinones of type I (compounds 5a-h; general procedure). Ethanol was added to 5-arylidene-2-selenoxohydantoin **3** (1 equiv.) and sarcosine (2 equiv.) and the mixture is brought to a boil. Then isatin (2 equiv.) was added. The reaction mixture was refluxed for 5 hours. The precipitate formed was filtered off and purified by flash chromatography (eluent MeOH : $CHCl_3$ = 1:50).

3-(4-ethoxyphenyl)-4'-(4-chlorophenyl)-1'-methyl-2-selenoxodispiro[imidazolidine-5,3'-pyrrolidine-2',3''-indoline]-2'',4-dione (5a)

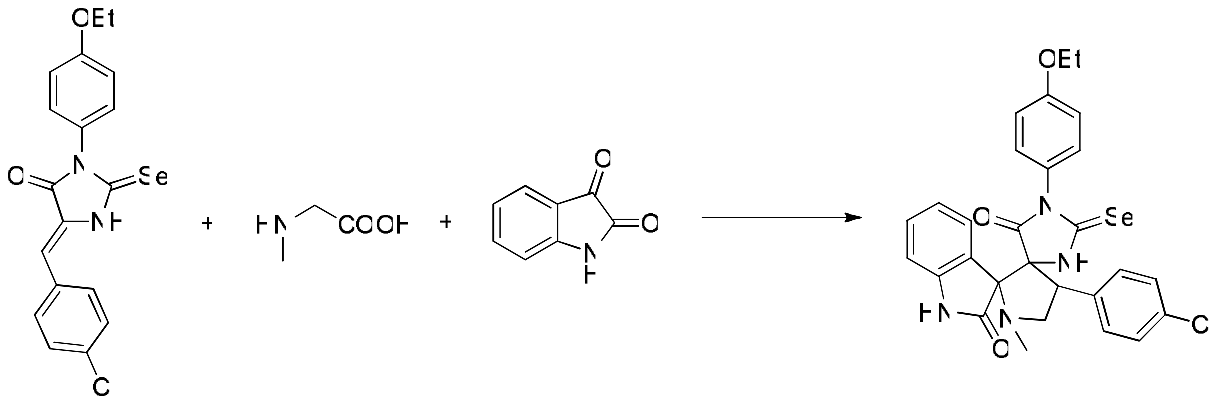

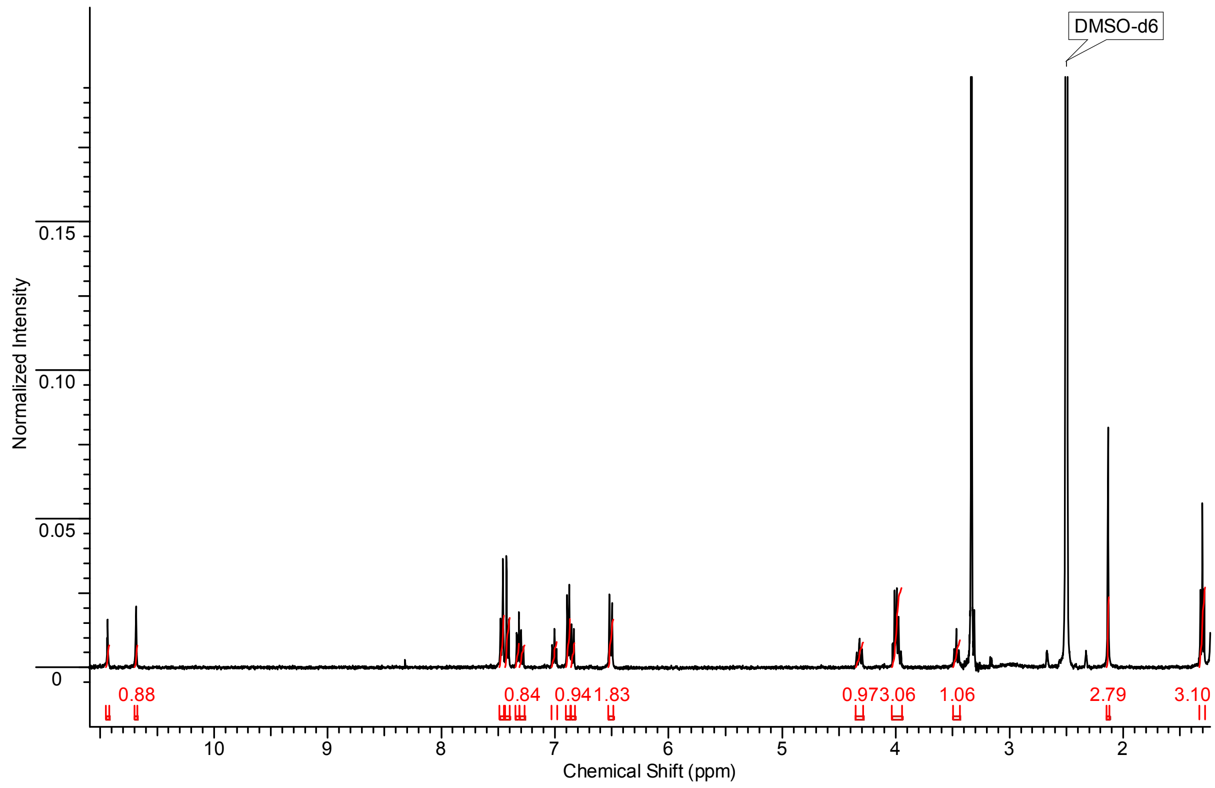

^{1}H NMR (400 MHz, DMSO-d6, δ, ppm):10.94 (s, 1H, NH), 10.69 (s, 1H, NH), 7.47 (d, J=8.6Hz, 2H, Ar), 7.42 (d, J=8.6Hz, 2H, Ar), 7.33 (d, J=7.5Hz, 1H, isatin), 7.30 (t, J=7.6Hz, 1H, isatin), 7.01 (t, J=7.6Hz, 1H, isatin), 6.88 (d, J=8.9Hz, 2H, Ar), 6.84 (d, J=7.6Hz, 1H, isatin), 6.51 (d, J=8.8Hz, 2H, Ar), 4.32 (t, J=9.1Hz, 1H, pyrrolidine), 4.00 (q, J=7.0Hz, ArO$\underline{CH_2}CH_3$), 3.98 (t, J=8.9Hz, pyrrolidine), 3.47 (t, J=8.8 Hz, 1H, pyrrolidine), 2.13 (s, 3H, NCH_3), 1.30 (t, J=6.9Hz, 3H, ArO$CH_2\underline{CH_3}$).

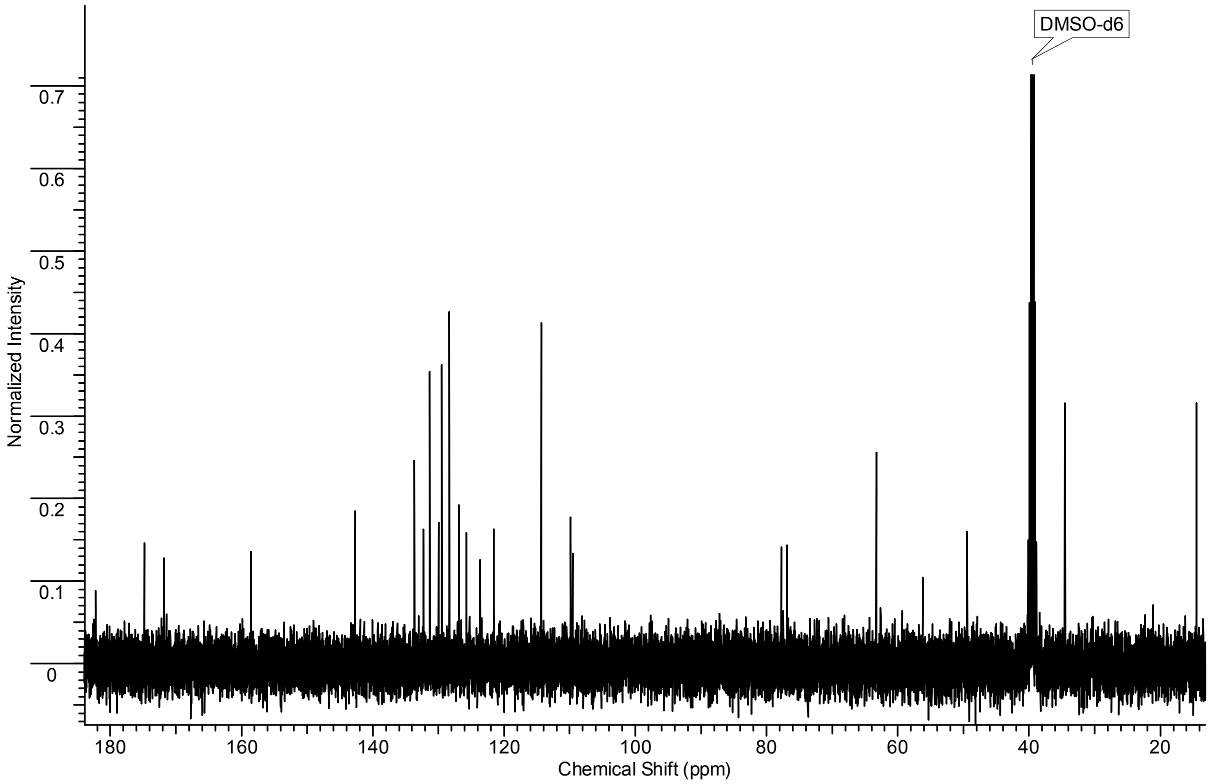

^{13}C NMR (101 MHz, DMSO-d6, δ, ppm): 182.2, 174.8, 171.8, 158.6, 142.7, 133.7, 132.3, 131.3, 130.0, 129.5, 128.4, 126.9, 125.7, 123.6, 121.6, 114.3, 109.9, 77.8, 76.9, 63.3, 56.2, 49.5, 34.6, 14.5.

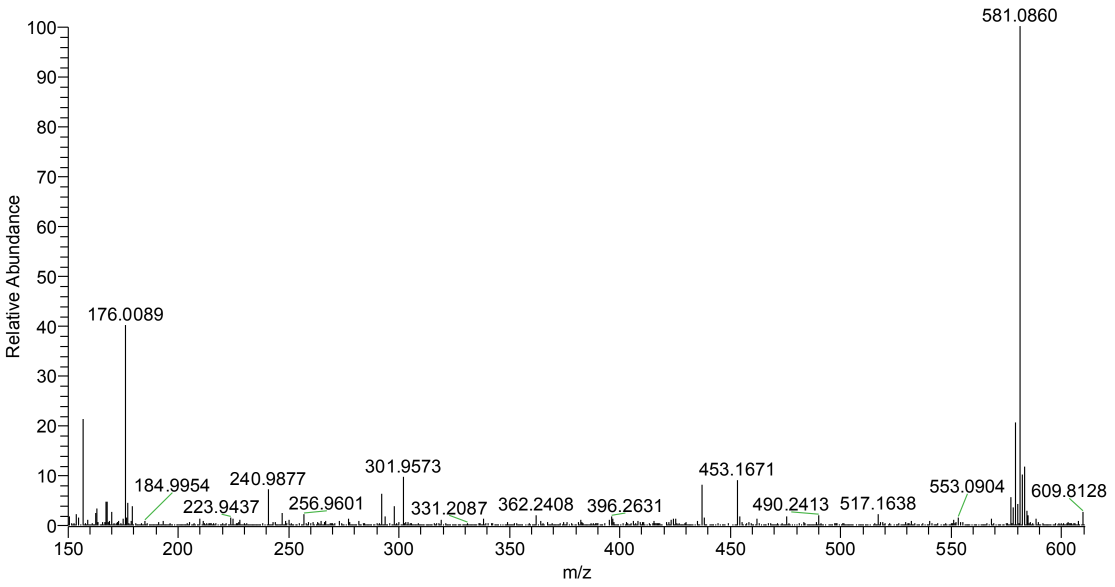

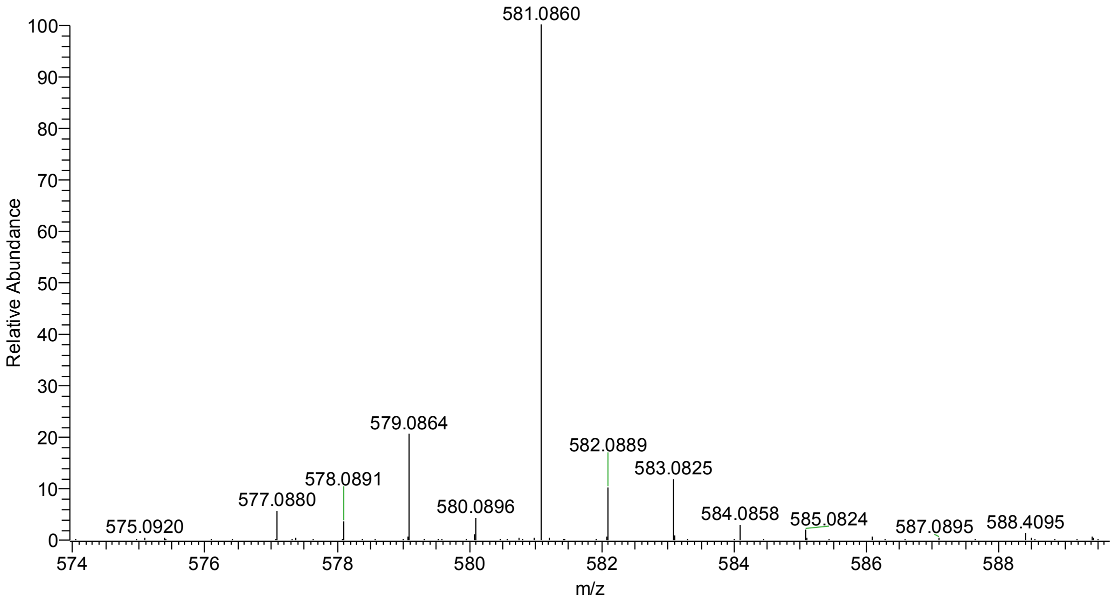

HRMS (ESI, m/Z): calculated ($C_{28}H_{25}ClN_4O_3Se$, M+H): 581.0853, found (M+H): 581.0860.

5''-chloro-3-(4-ethoxyphenyl)-4'-(4-chlorophenyl)-1'-methyl-2-selenoxodispiro[imidazolidine-5,3'-pyrrolidine-2',3''-indoline]-2'',4-dione (5b)

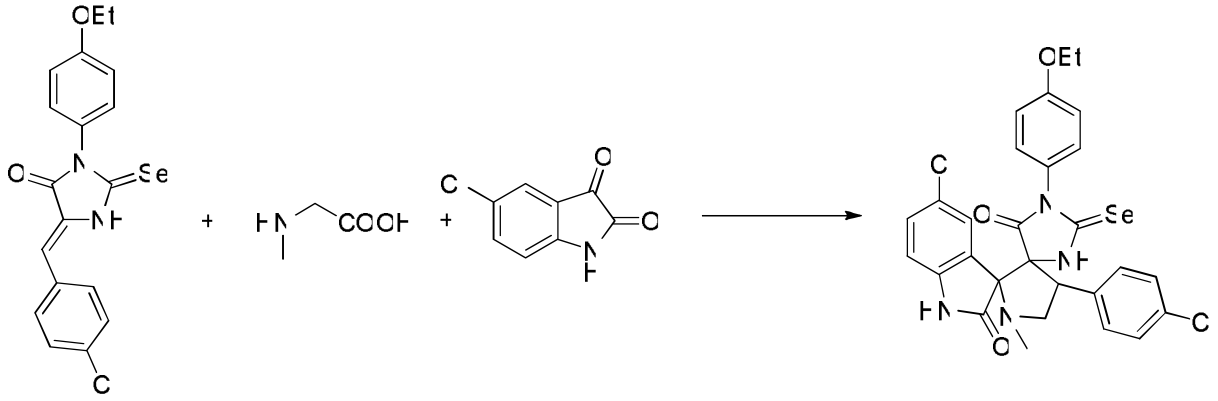

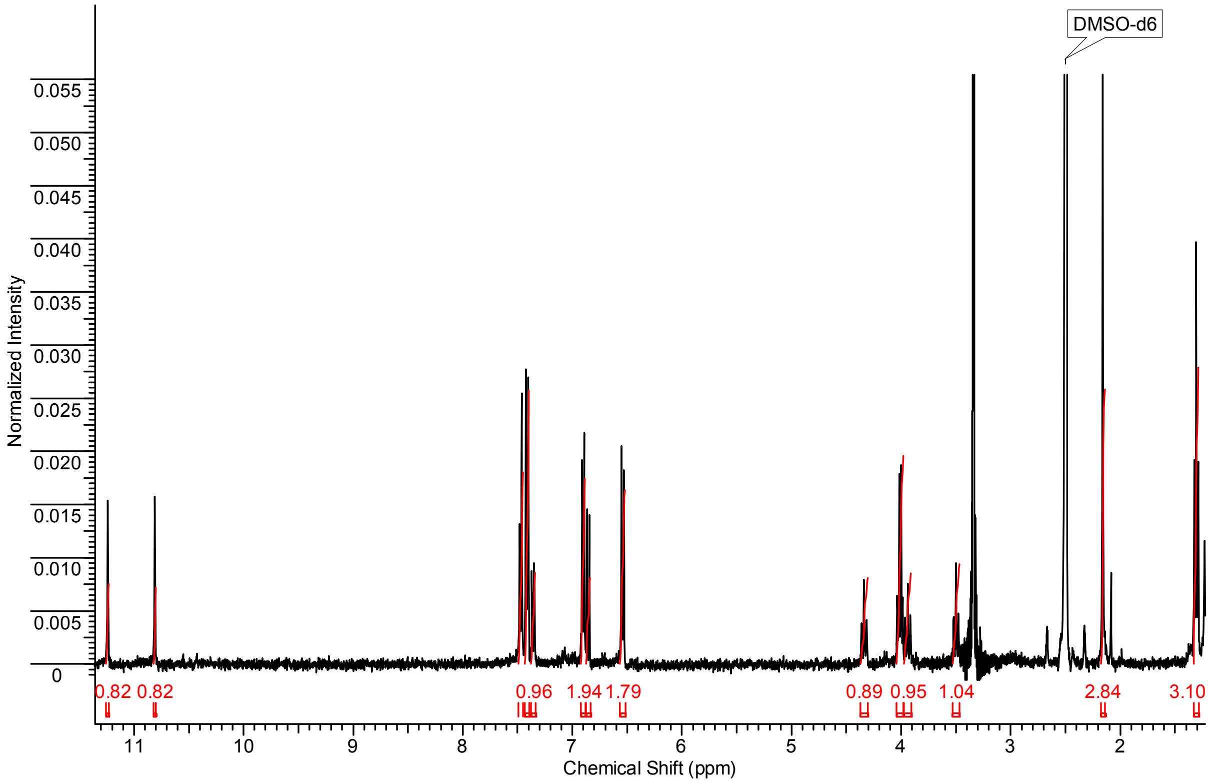

^1H NMR (400 MHz, DMSO-d6, δ, ppm):11.24 (s, 1H, NH), 10.81 (s, 1H, NH), 7.47 (d, J=8.6Hz, 2H, Ar), 7.41 (d, J=8.3Hz, 2H, Ar), 7.43-7.39(m, 1H, isatin), 7.36 (dd, J_1=2.1Hz, J_2=8.3Hz, 1H, isatin), 6.90 (d, J=8.9Hz, 2H, Ar), 6.86 (d, J=8.3Hz, 1H, isatin), 6.54 (d, J=8.8Hz, 2H, Ar), 4.34 (t, J=9.0Hz, 1H, pyrrolidine), 4.01 (q, J=6.9Hz, ArO<u>CH₂</u>CH₃), 3.94 (t, J=9.7Hz, 1H, pyrrolidine), 3.50 (t, J=8.6Hz, 1H, pyrrolidine), 2.16 (s, 3H, NCH_3), 1.31 (t, J=6.9Hz, 3H, $ArOCH_2\underline{CH_3}$).

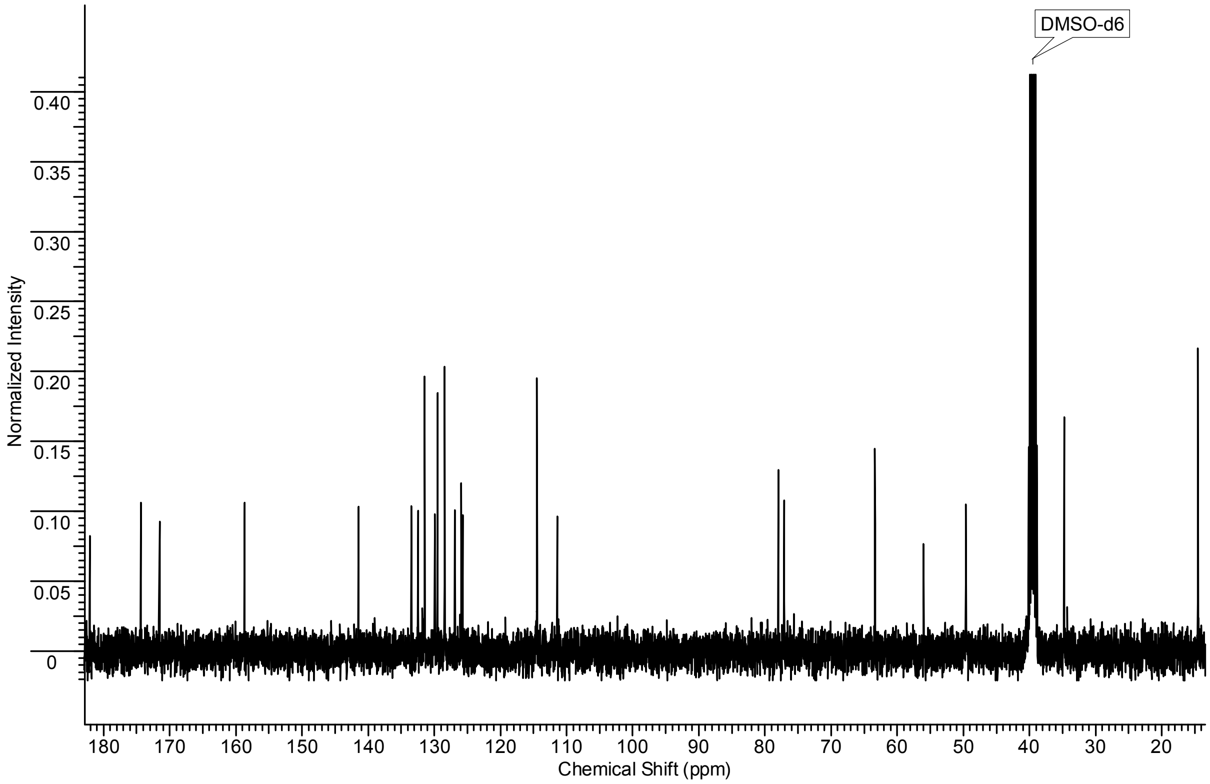

^{13}C NMR (101 MHz, DMSO-d6, δ, ppm): 182.1, 174.4, 171.5, 158.7, 141.5, 133.5, 132.4, 131.5, 129.9, 129.5, 128.4, 126.9, 125.9, 125.8, 125.7, 114.5, 111.4, 78.0, 77.1, 63.4, 56.0, 49.6, 34.8, 14.6.

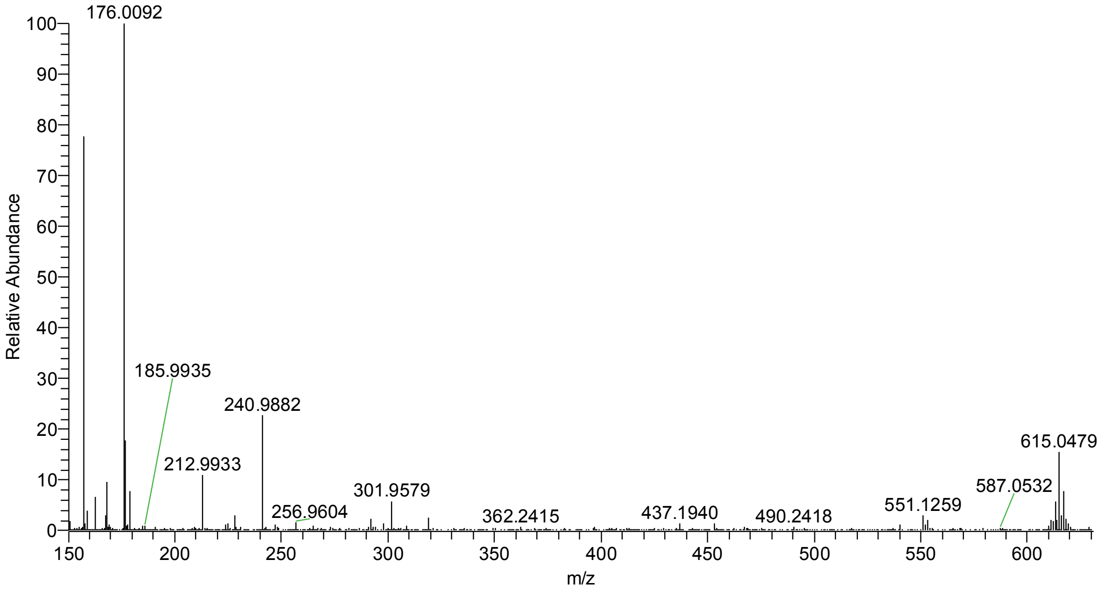

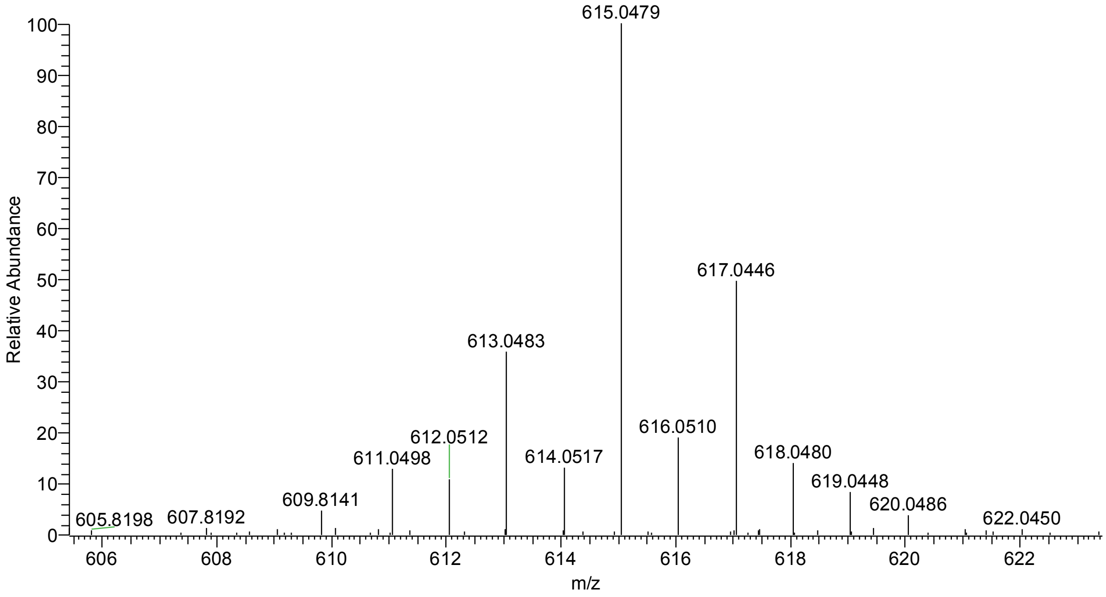

HRMS (ESI, m/Z): calculated ($C_{28}H_{24}Cl_2N_4O_3Se$, M+H): 615.0463, found (M+H): 615.0479.

5’’-bromo-3-(4-ethoxyphenyl)-4'-(4-chlorophenyl)-1'-methyl-2-selenoxodispiro[imidazolidine-5,3'-pyrrolidine-2',3''-indoline]-2'',4-dione (5c)

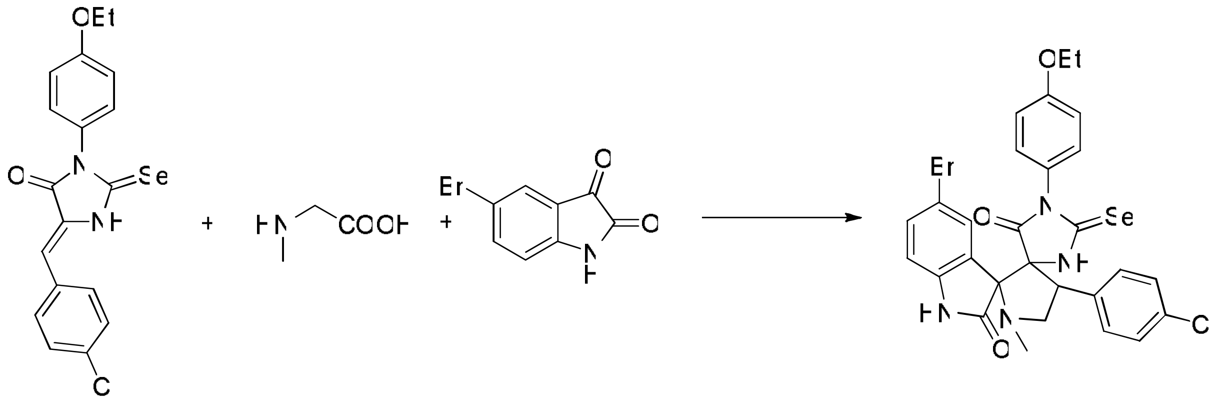

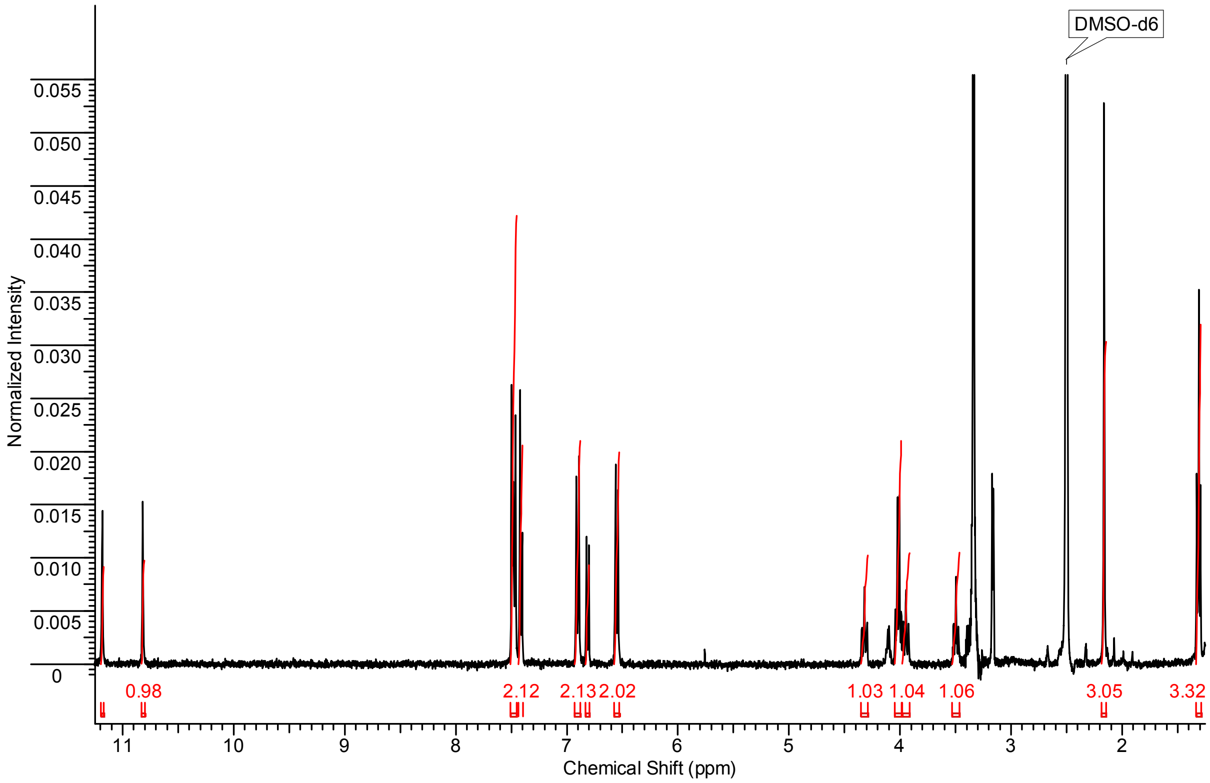

^{1}H NMR (400 MHz, DMSO-d6, δ, ppm):11.18 (s, 1H, NH), 10.82 (s, 1H, NH), 7.51-7.44 (m, 4H, Ar+isatin), 7.41 (d, J=8.6Hz, 2H, Ar), 6.90 (d, J=8.9Hz, 2H, Ar), 6.81 (d, J=8.8Hz, 1H, isatin), 6.55 (d, J=8.8Hz, 2H, Ar), 4.32 (t, J=9.2Hz, 1H, pyrrolidine), 4.01 (q, J=7.0Hz, ArO$\underline{CH_2}$CH$_3$), 3.95 (t, J=9.1Hz, 1H, pyrrolidine), 3.50 (t, J=9.1Hz, 1H, pyrrolidine), 2.16 (s, 3H, NCH$_3$), 1.31 (t, J=6.9Hz, 3H, ArOCH$_2$$\underline{CH_3}$).

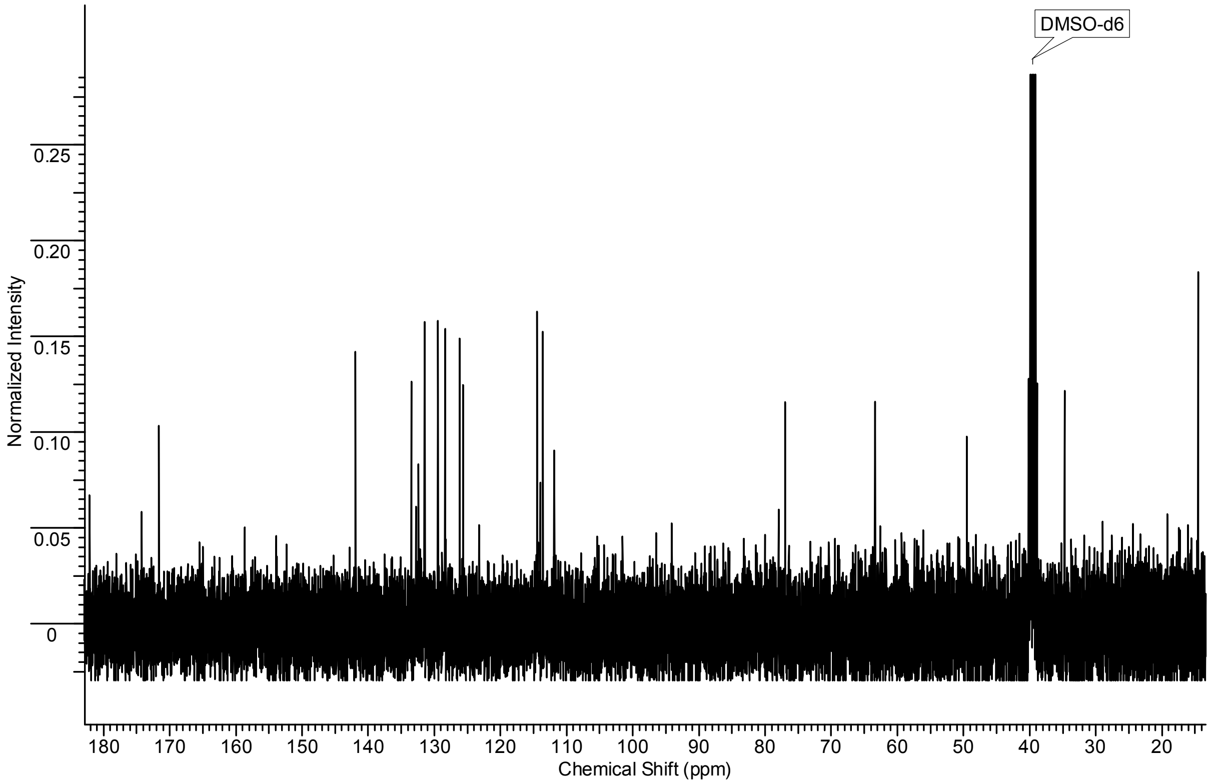

^{13}C NMR (101 MHz, DMSO-d6, δ, ppm): 182.1, 174.2, 171.6, 158.7, 141.9, 133.5, 132.8, 132.4, 131.4, 129.5, 129.4, 128.4, 126.2, 125.7, 123.2, 114.5, 113.6, 111.9, 77.0, 63.4, 49.5, 34.7, 14.6.

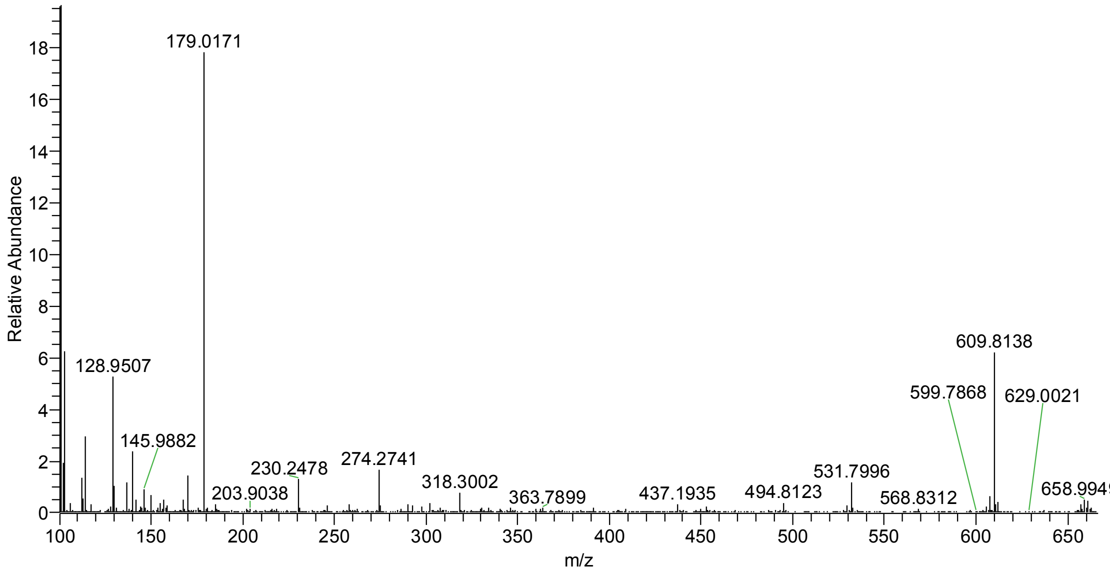

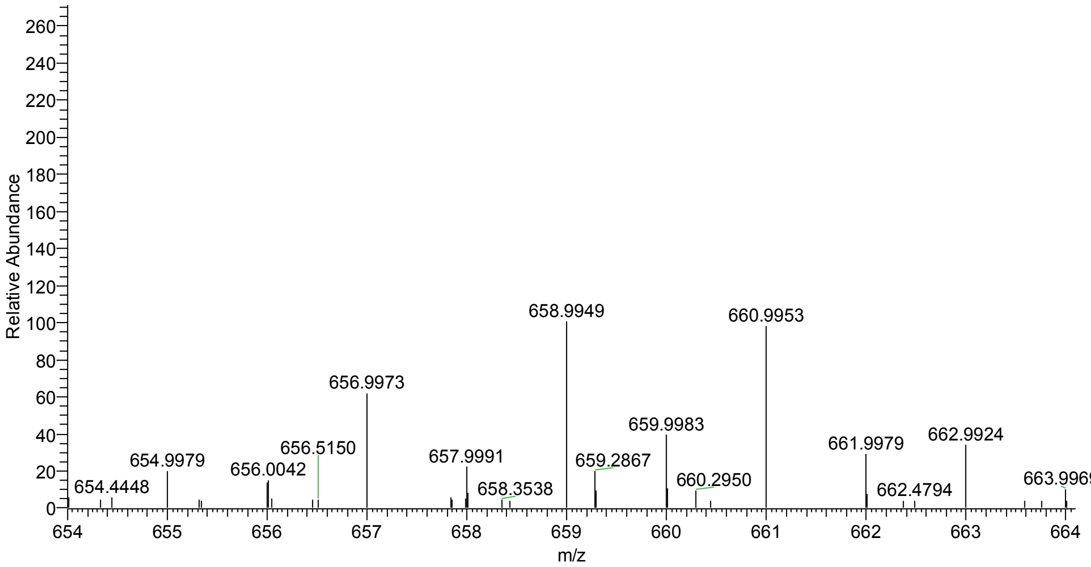

HRMS (ESI, m/Z): calculated ($C_{28}H_{24}BrClN_4O_3Se$, M+H): 658.9958, found (M+H): 658.9949.

3-(4-methoxyphenyl)-4'-(4-chlorophenyl)-1'-methyl-2-selenoxodispiro[imidazolidine-5,3'-pyrrolidine-2',3''-indoline]-2'',4-dione (5d)

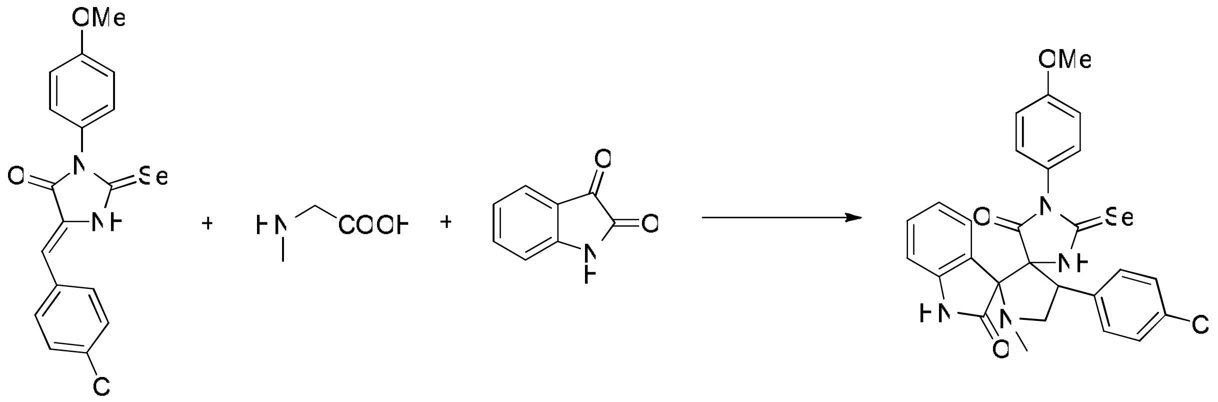

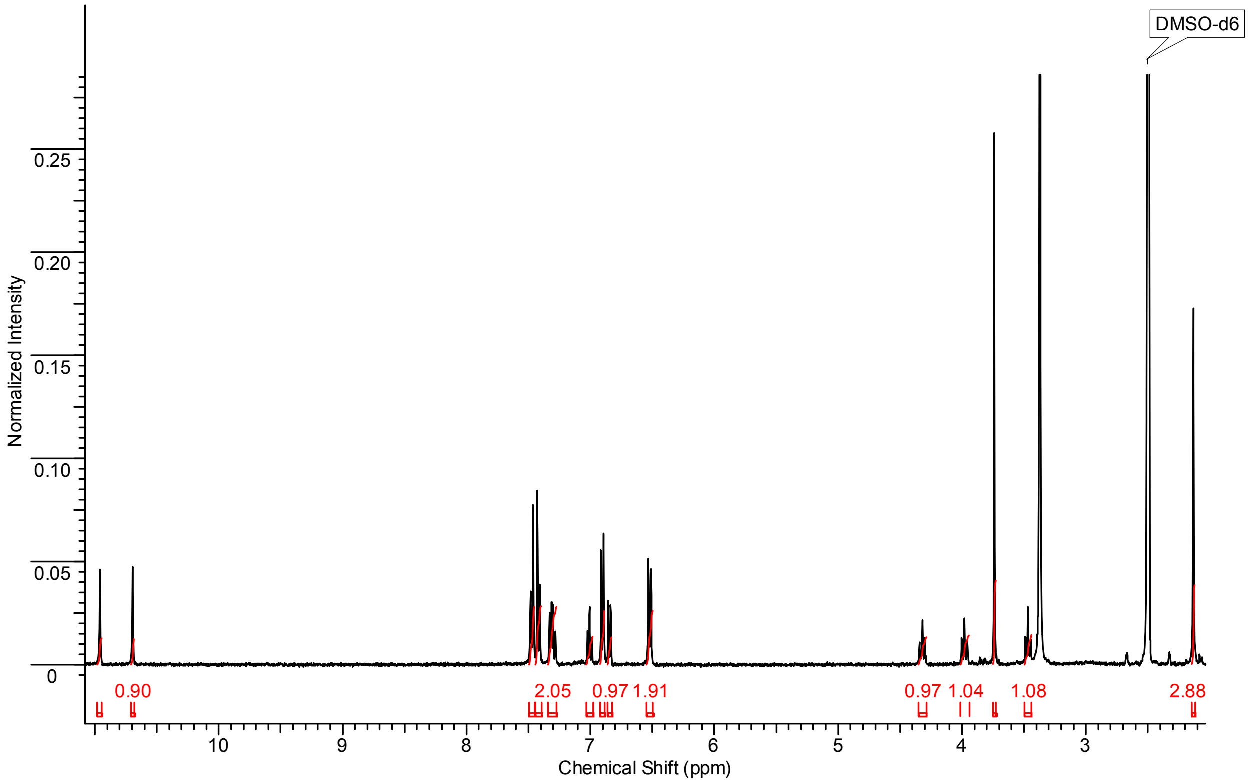

^{1}H NMR (400 MHz, DMSO-d6, δ, ppm):10.96 (s, 1H, NH), 10.69 (s, 1H, NH), 7.47 (d, J=8.6Hz, 2H, Ar), 7.42 (d, J=8.5Hz, 2H, Ar), 7.32 (d, J=7.3Hz, 1H, isatin), 7.30 (t, J=7.6Hz, 1H, isatin), 7.01 (t, J=7.5Hz, 1H, isatin), 6.90 (d, J=9.1Hz, 2H, Ar), 6.85 (d, J=7.6Hz, 1H, isatin), 6.52 (d, J=8.8Hz, 2H, Ar), 4.32 (t, J=9.1Hz, 1H, pyrrolidine), 3.98 (t, J=9.3Hz, 1H, pyrrolidine), 3.74 (s, 3H, OCH_3), 3.47 (t, J=8.7Hz, 1H, pyrrolidine), 2.13 (s, 3H, NCH_3).

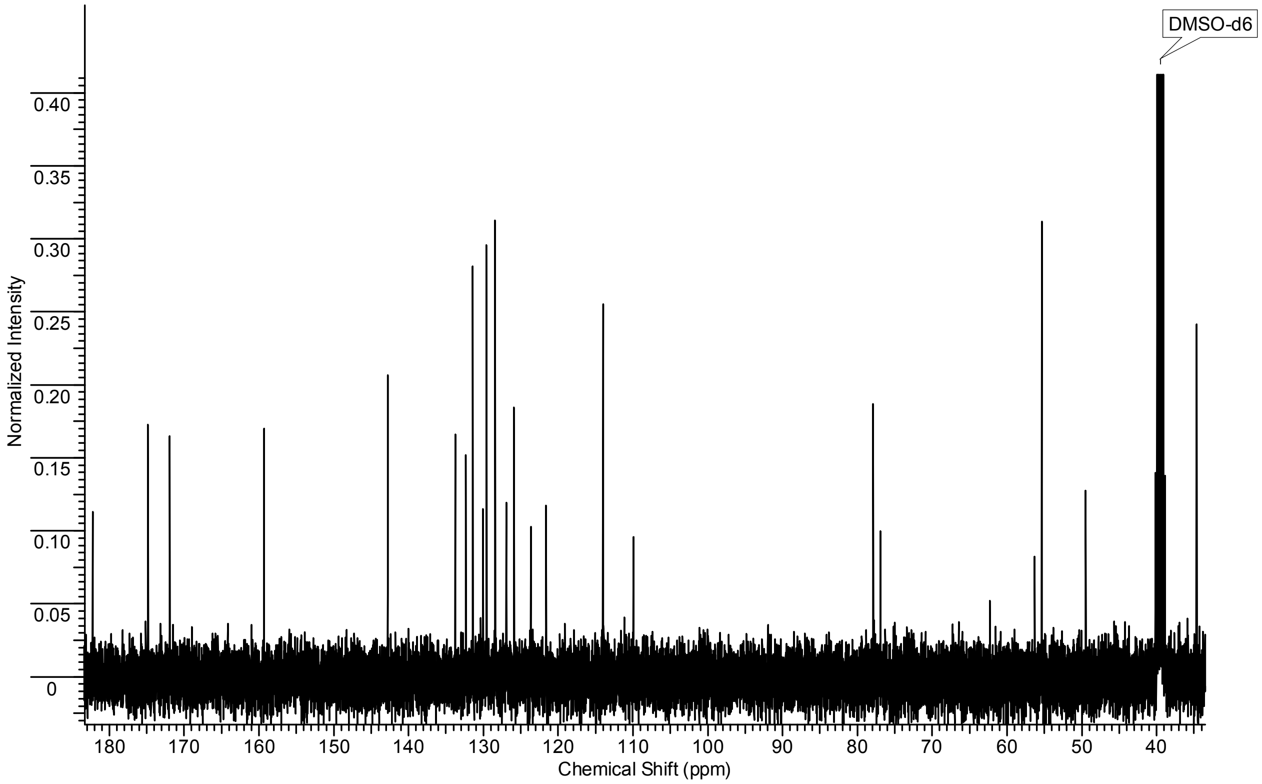

^{13}C NMR (101 MHz, DMSO-d6, δ, ppm): 182.2, 174.9, 171.9, 159.3, 142.8, 133.8, 132.3, 131.4, 130.0, 129.6, 128.4, 126.9, 125.9, 123.6, 121.6, 114.0, 109.9, 77.9, 76.9, 56.3, 55.4, 49.5, 34.6.

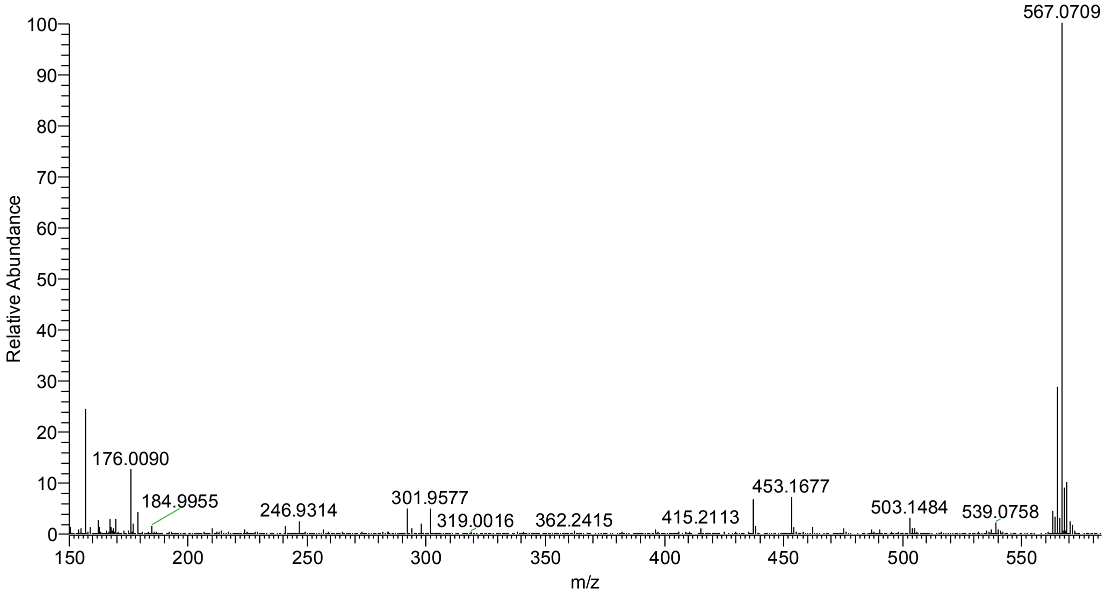

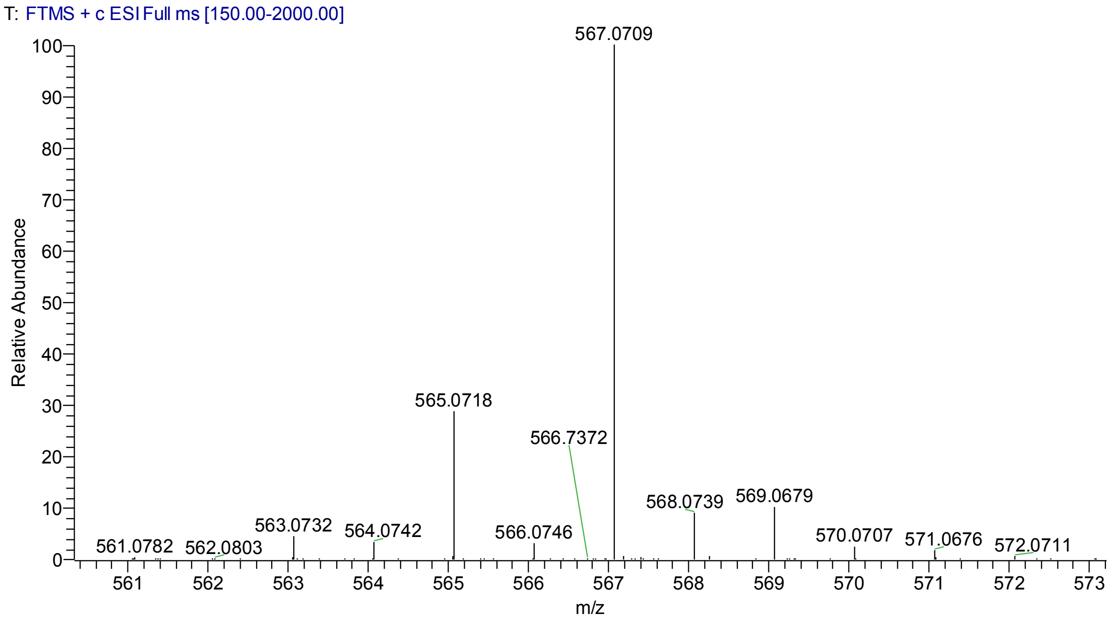

HRMS (ESI, m/Z): calculated ($C_{27}H_{23}ClN_4O_3Se$, M+H): 567.0697, found (M+H): 567.0709.

5’’-chloro-3-(4-methoxyphenyl)-4'-(4-chlorophenyl)-1'-methyl-2-selenoxodispiro[imidazolidine-5,3'-pyrrolidine-2',3''-indoline]-2'',4-dione (5e)

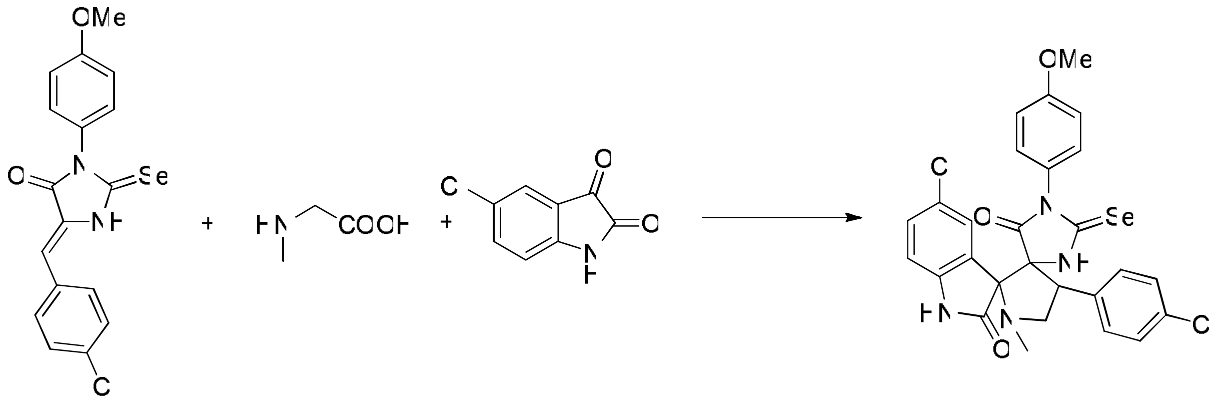

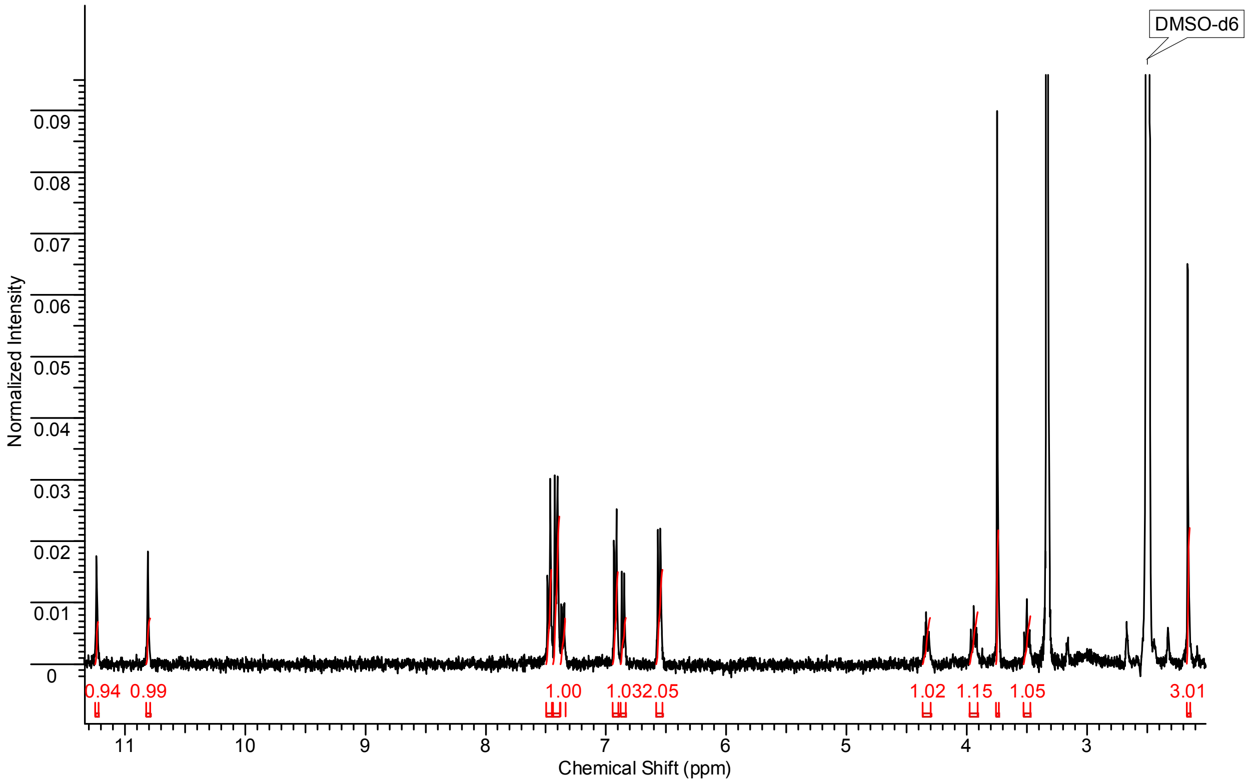

^{1}H NMR (400 MHz, DMSO-d6, δ, ppm):11.24 (s, 1H, NH), 10.81 (s, 1H, NH), 7.47 (d, J=8.7Hz, 2H, Ar), 7.41 (d, J=8.6Hz, 2H, Ar), 7.40 (s, 1H, isatin), 7.36 (dd, J_1=2.1Hz, J_2=8.3Hz, 1H, isatin), 6.92 (d, J=9.0Hz, 2H, Ar), 6.86 (d, J=8.2Hz, 1H, isatin), 6.56 (d, J=8.8Hz, 2H, Ar), 4.34 (t, J=8.8Hz, 1H, pyrrolidine), 3.94 (t, J=9.2Hz, 1H, pyrrolidine), 3.75 (s, 3H, OCH_3), 3.50 (t, J=9.1Hz, 1H, pyrrolidine), 2.16 (s, 3H, NCH_3).

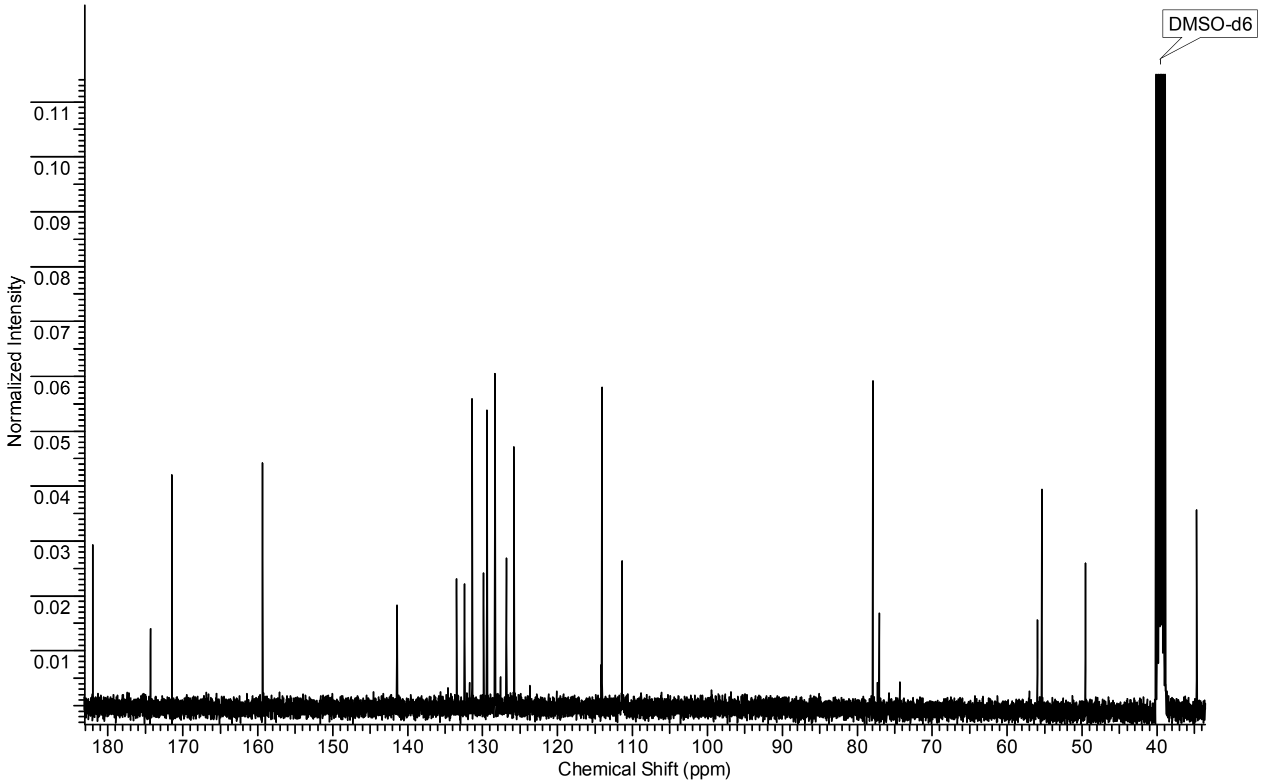

^{13}C NMR (101 MHz, DMSO-d6, δ, ppm): 182.0, 174.3, 171.5, 159.4, 141.4, 133.4, 132.4, 131.4, 129.9, 129.4, 128.4, 126.8, 125.9, 125.8, 114.1, 111.4, 77.9, 77.1, 56.0, 55.4, 49.6, 34.7.

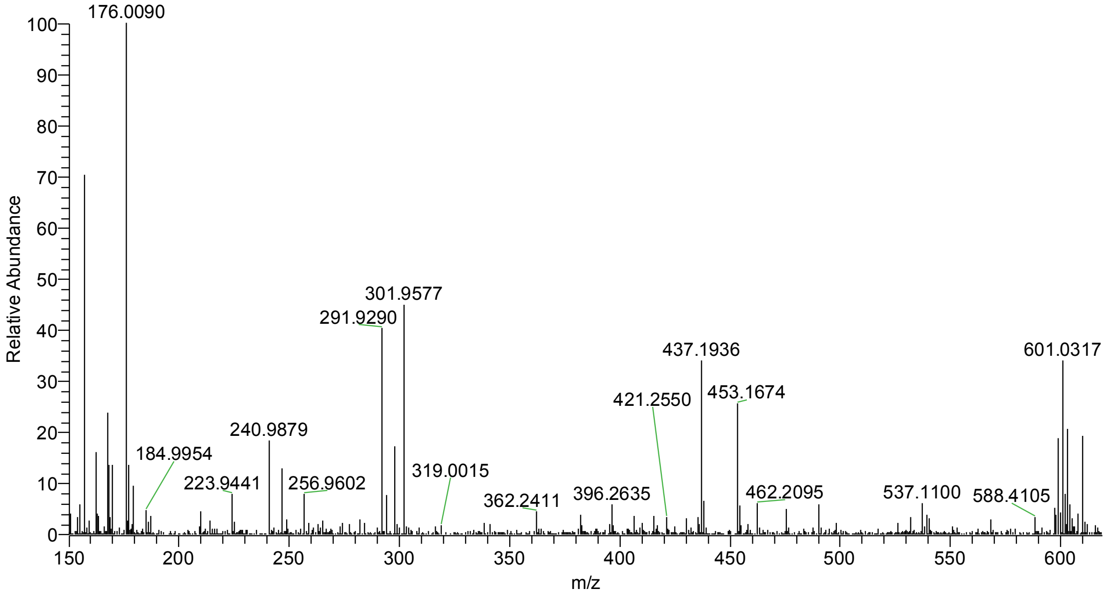

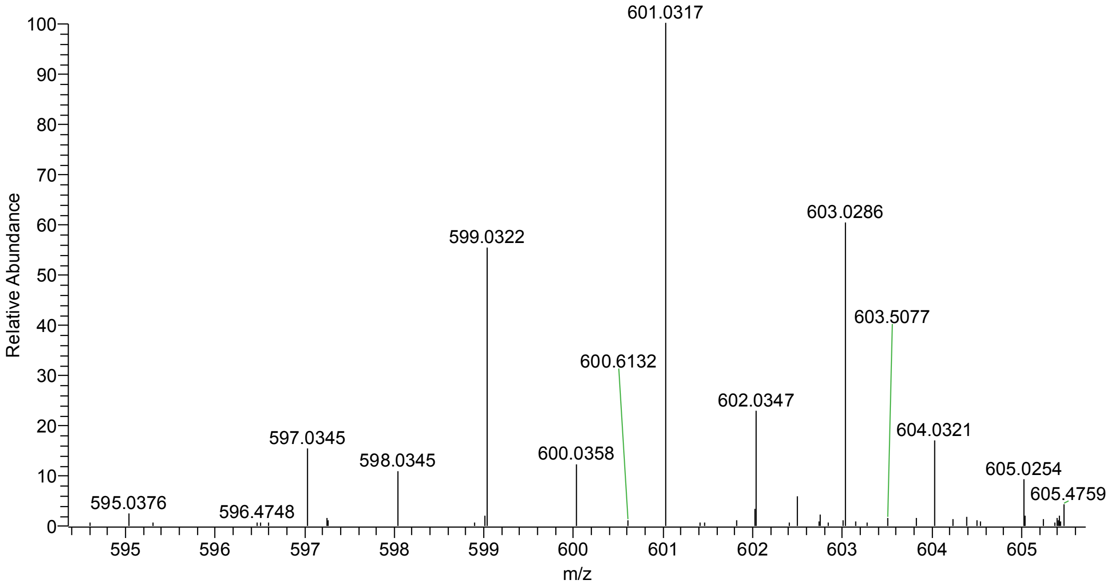

HRMS (ESI, m/Z): calculated ($C_{27}H_{22}Cl_2N_4O_3Se$, M+H): 601.0307, found (M+H): 601.0317.

5’’-bromo-3-(4-methoxyphenyl)-4'-(4-chlorophenyl)-1'-methyl-2-selenoxodispiro[imidazolidine-5,3'-pyrrolidine-2',3''-indoline]-2'',4-dione (5f)

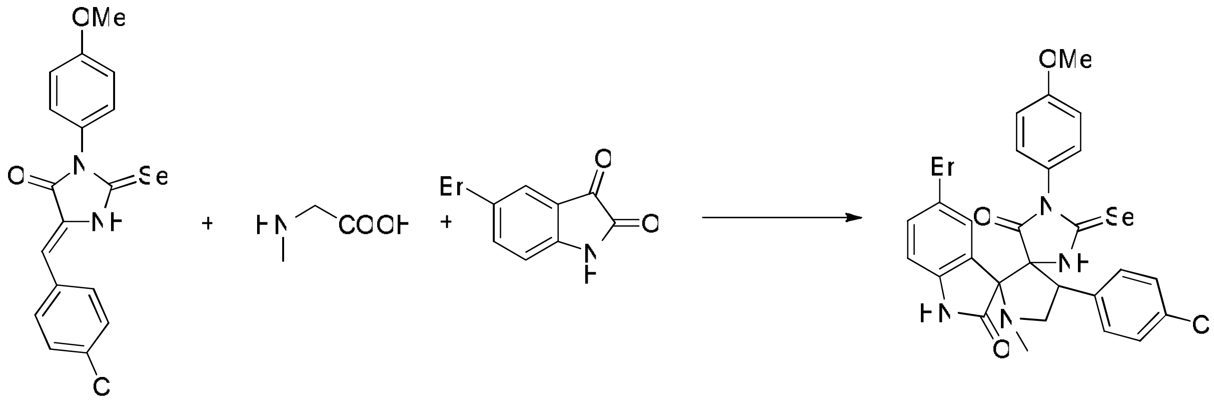

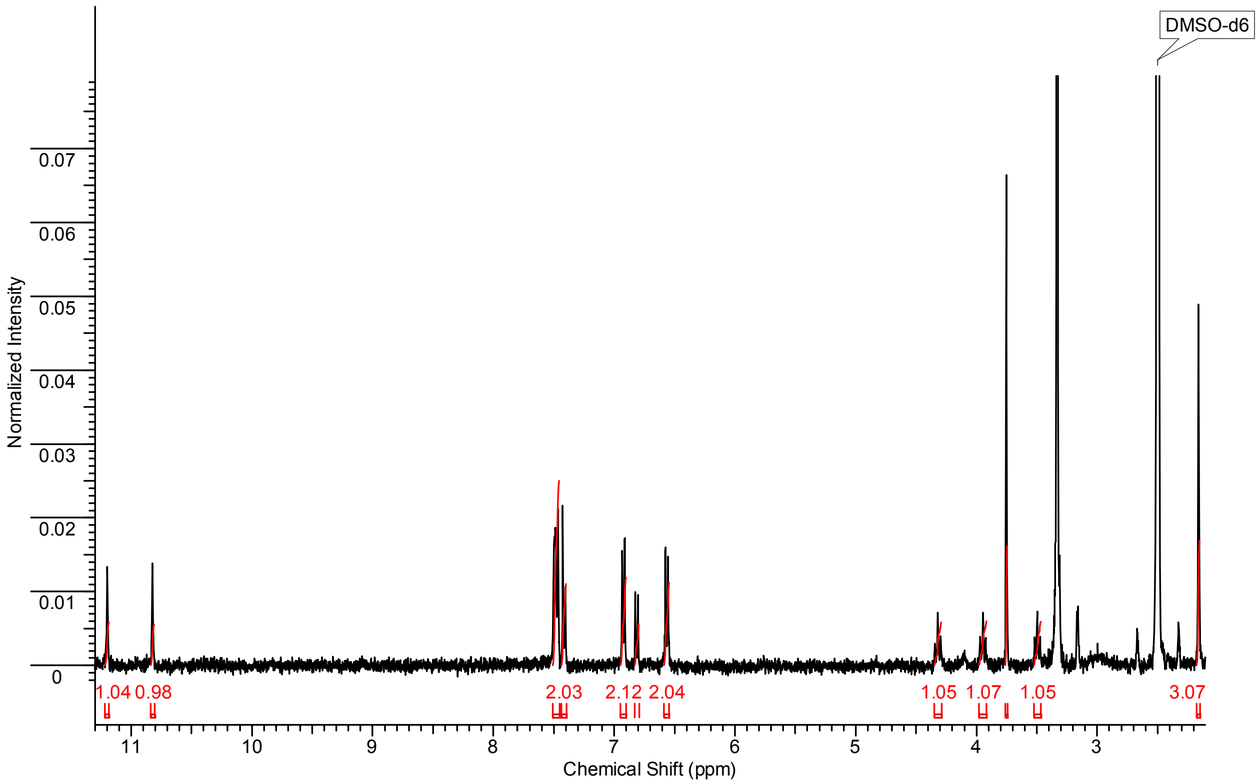

^{1}H NMR (400 MHz, DMSO-d6, δ, ppm):11.20 (s, 1H, NH), 10.82 (s, 1H, NH), 7.52-7.45 (m, 4H, Ar+isatin), 7.41 (d, J=8.7Hz, 2H, Ar), 6.92 (d, J=8.7Hz, 2H, Ar), 6.81 (d, J=8.7Hz, 1H, isatin), 6.56 (d, J=8.7Hz, 2H, Ar), 4.32 (t, J=8.9Hz, 1H, pyrrolidine), 3.95 (t, J=9.2Hz, 1H, pyrrolidine), 3.75 (s, 3H, OCH_3) 3.50 (t, J=9.2Hz, 1H, pyrrolidine), 2.16 (s, 3H, NCH_3).

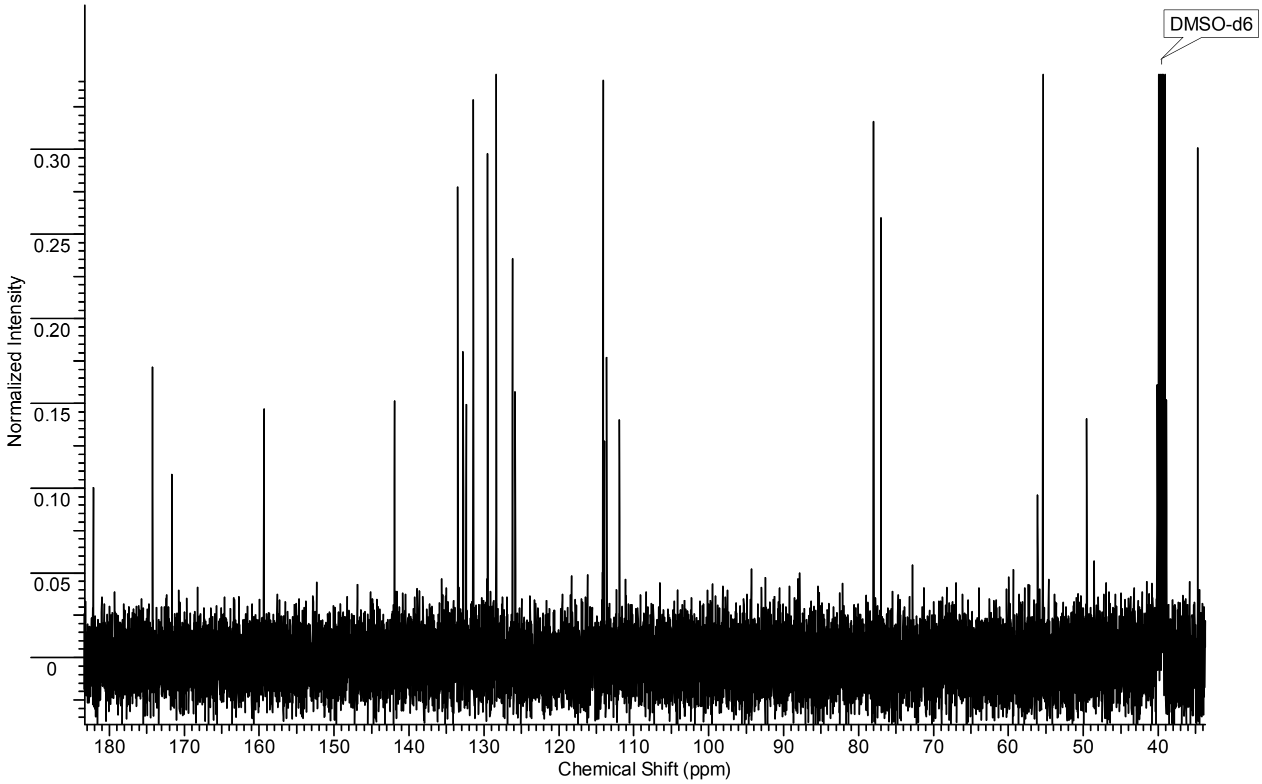

^{13}C NMR (101 MHz, DMSO-d6, δ, ppm): 182.1, 174.2, 171.7, 159.4, 141.9, 133.5, 132.8, 132.4, 131.5, 129.5, 129.4, 128.4, 126.2, 125.8, 114.1, 114.0, 113.6, 111.9, 78.0, 77.0, 56.1, 55.4, 49.5, 34.7.

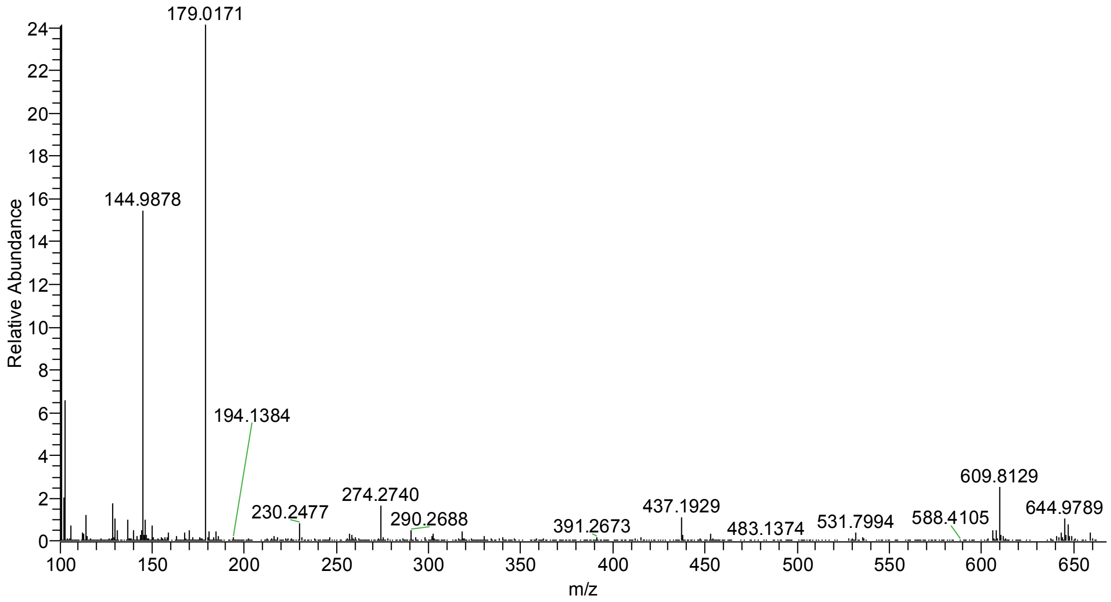

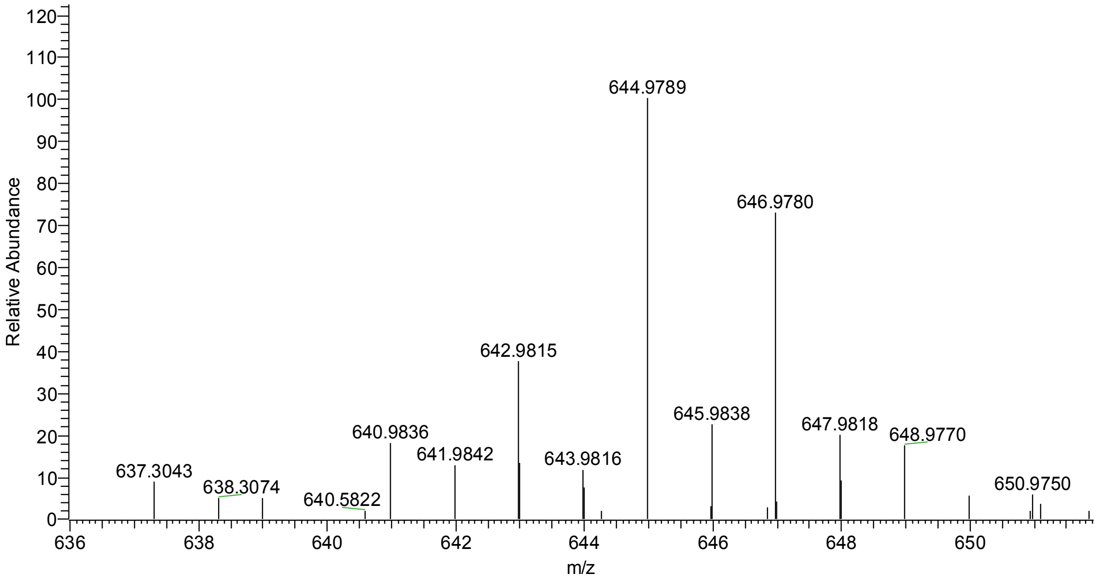

HRMS (ESI, m/Z): calculated ($C_{27}H_{22}BrClN_4O_3Se$, M+H): 644.9802, found (M+H): 644.9789.

5’’-bromo-3-(4-methoxyphenyl)-4'-(4-ethylphenyl)-1'-methyl-2-selenoxodispiro[imidazolidine-5,3'-pyrrolidine-2',3''-indoline]-2'',4-dione (5g)

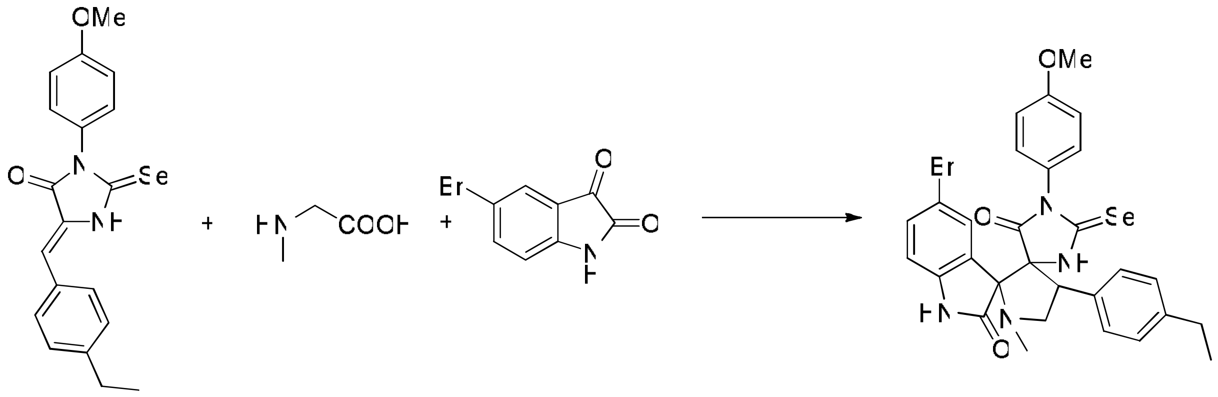

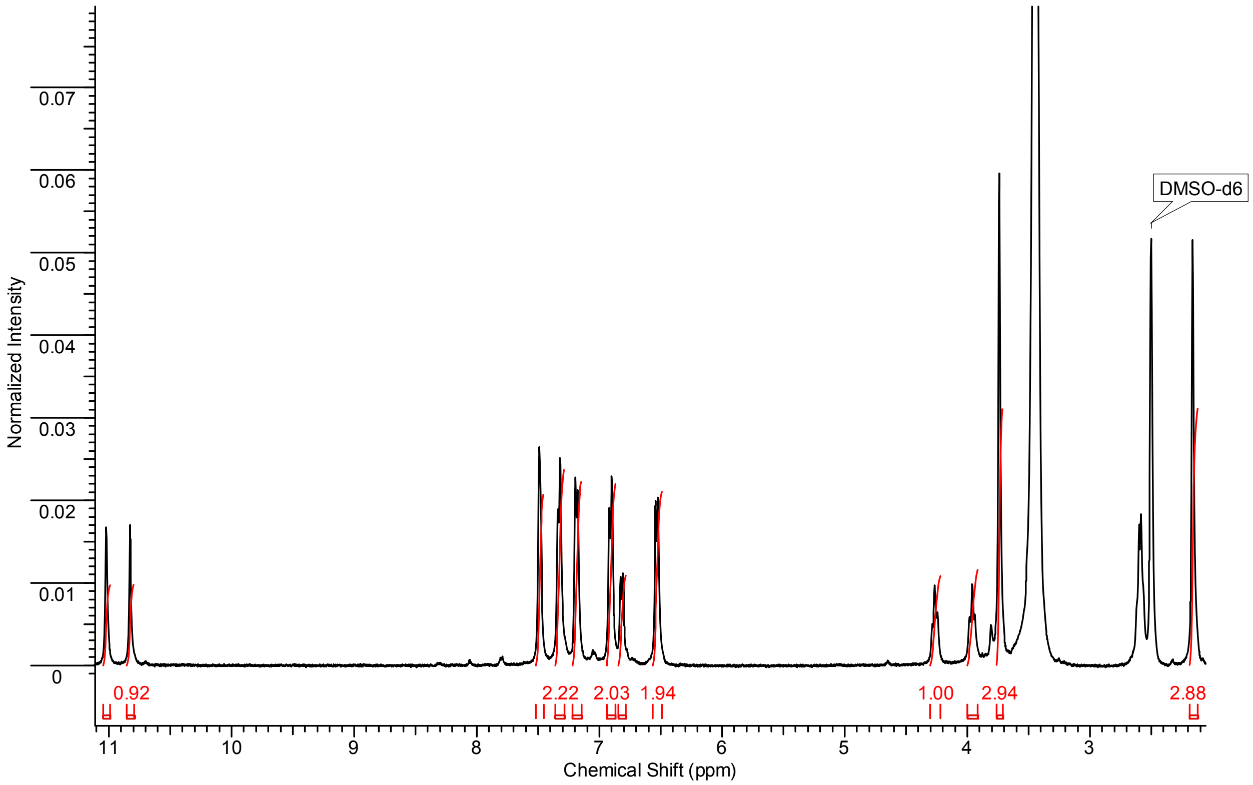

^{1}H NMR (400 MHz, DMSO-d6, δ, ppm): 11.02 (s, 1H, NH), 10.83 (s, 1H NH), 7.52-7.45 (m, 2H, isatin), 7.33 (d, J=6.6 Hz, 2H, Ar), 7.19 (d, J=6.6 Hz, 2H, Ar), 6.91 (d, J=7.7 Hz, 2H, Ar), 6.82 (d, J=8.2 Hz, 1H, isatin), 6,53 (d, J=7.5 Hz, 2H, Ar), 4.26 (t, J=8.8 Hz, 1H, pyrrolidine), 3,96 (t, J=8.9 Hz, 1H, pyrrolidine), 3,74 (s, 3H, OCH_3), 3.55-3.50 (m, 1H,

pyrrolidine), 2.59 (q, J=7.2 Hz, 2H, Ar$\underline{CH_2}$$CH_3$), 2.16 (s, 3H, NCH_3), 1.16 (t, J=7.1 Hz, 3H, $ArCH_2$$\underline{CH_3}$).

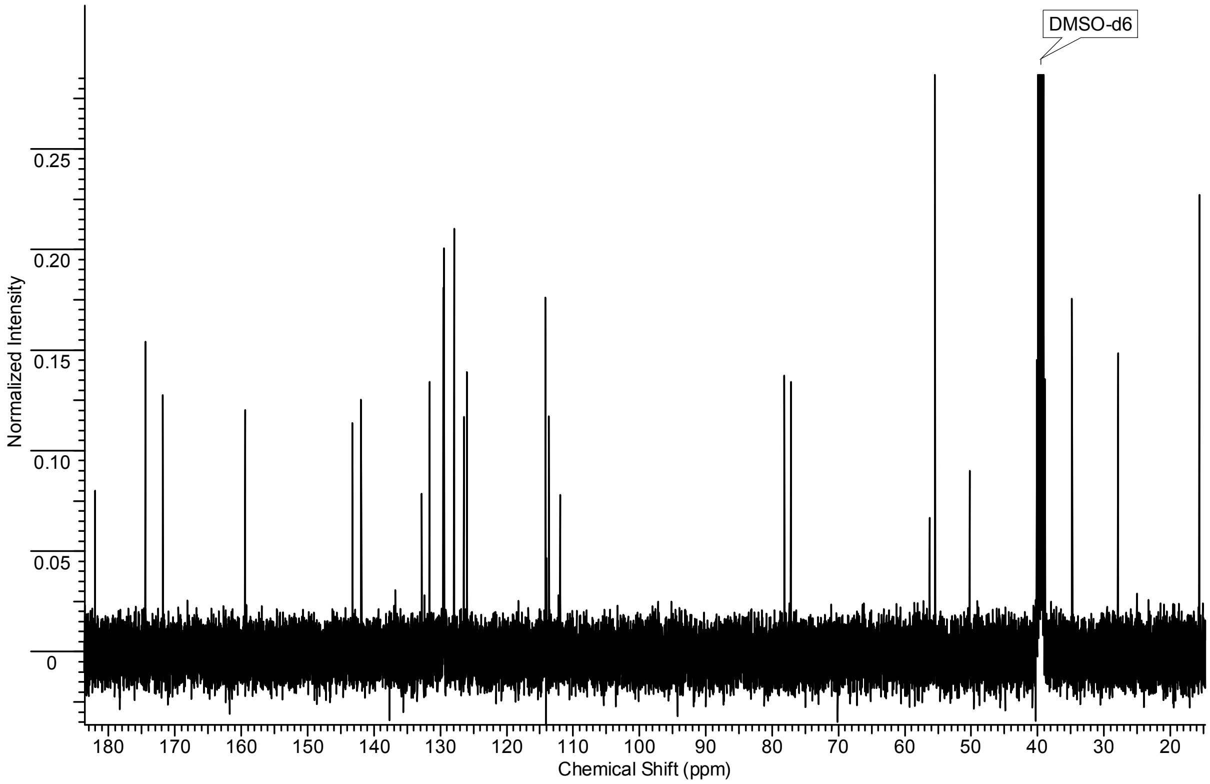

^{13}C NMR (101 MHz, DMSO-d6, δ, ppm):182.0, 174.5, 171.8, 159.4, 143.2, 142.0, 132.8, 131.6, 129.6, 129.4, 127.9, 126.5, 126.0, 114.1, 113.6, 112.0, 78.2, 77.2, 56.2, 55.5, 50.2, 34.8, 27.9, 15.6.

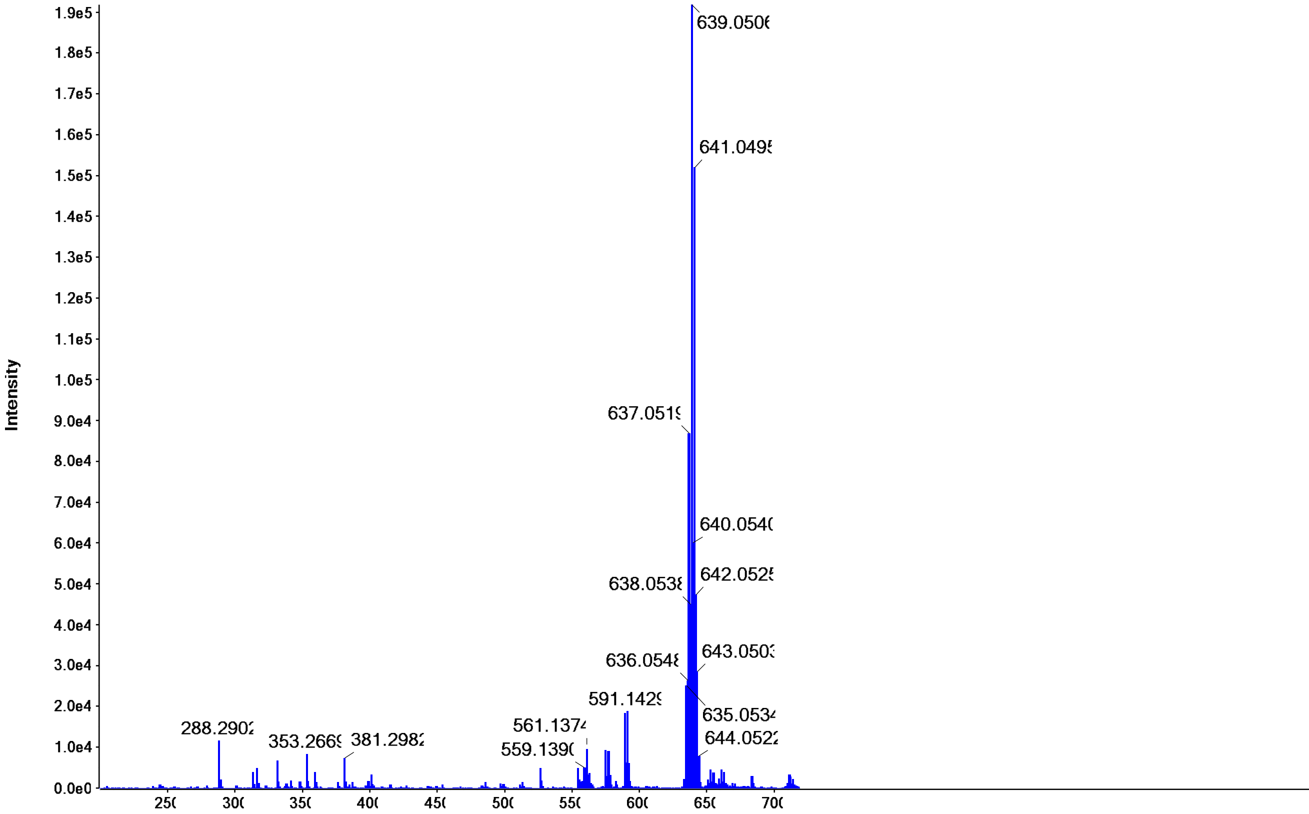

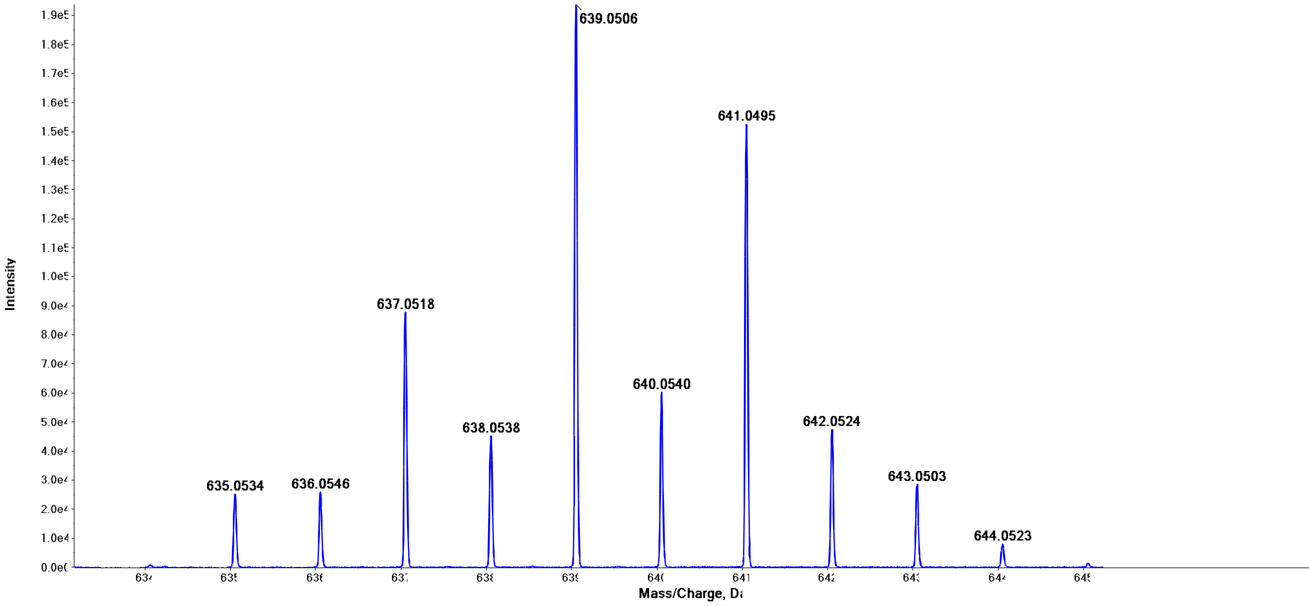

HRMS (ESI, m/Z): calculated ($C_{29}H_{27}BrN_4O_3Se$, M+H): 639.0342, found (M+H): 639.0506.

3-(4-methoxyphenyl)-4'-(4-methoxyphenyl)-1'-methyl-2-selenoxodispiro[imidazolidine-5,3'-pyrrolidine-2',3''-indoline]-2'',4-dione (**5h**)

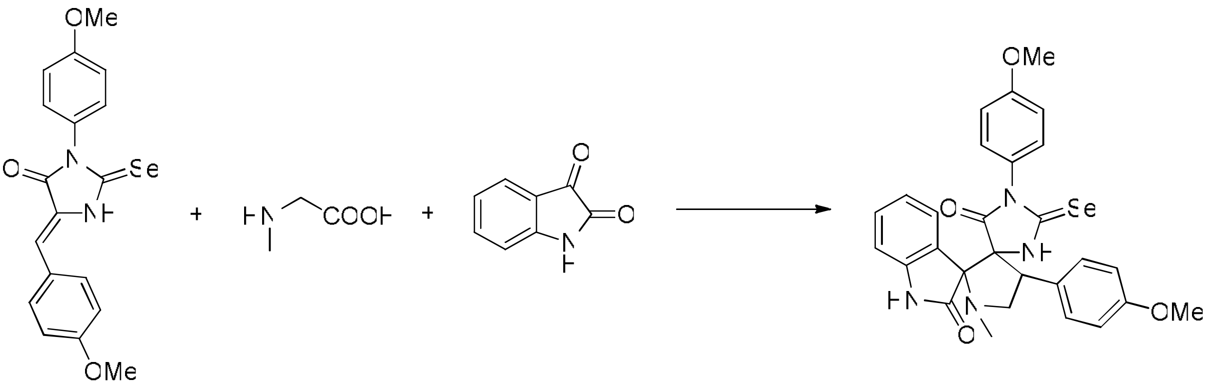

11H

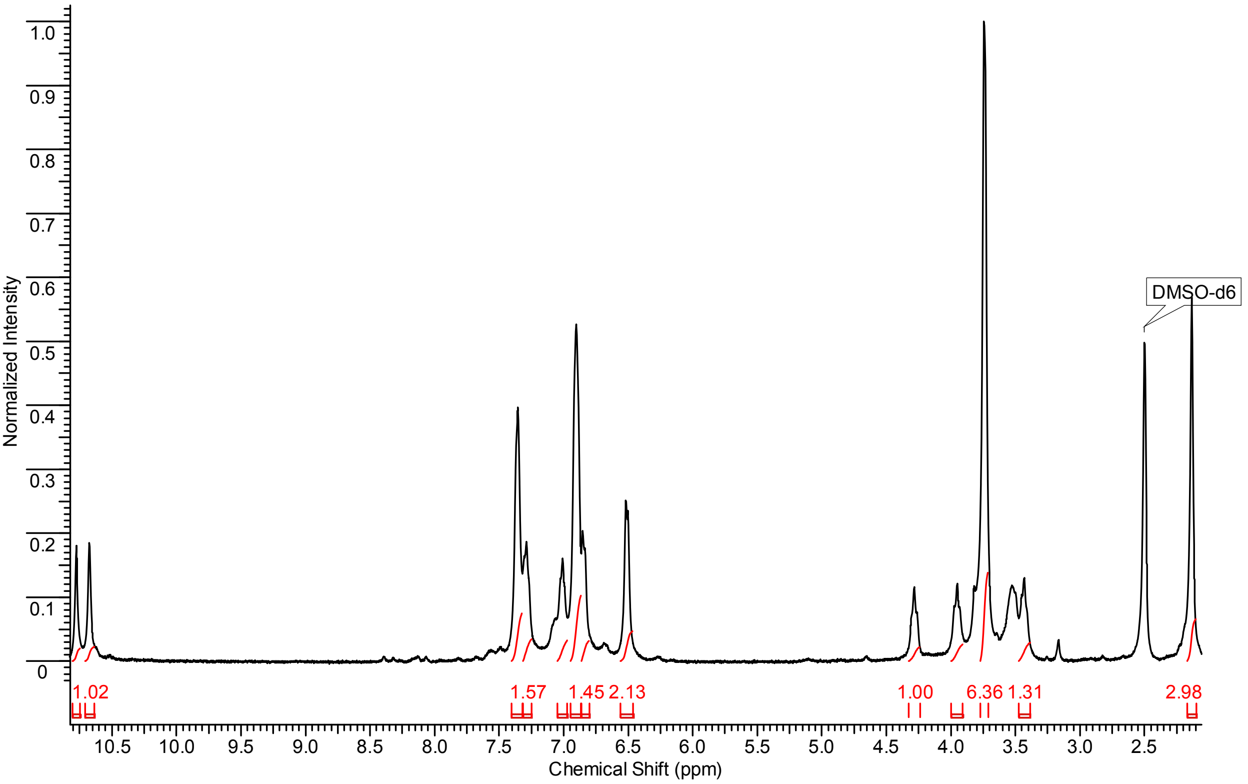

^{1}H NMR (400 MHz, DMSO-d6, δ, ppm): 10.78 (s, 1H, NH), 10.68 (s, 1H, NH), 7.40-7.32 (m, 3H, Ar+isatin), 7.29 (t, J=7.0 Hz, 1H, isatin), 7.01 (t, J=6.4 Hz, 1H, isatin), 6.95-6.86 (m, 4H, Ar), 6.84 (d, J=7.2 Hz, 1H, isatin), 6.51 (d, J=7.5 Hz, 2H, Ar), 4.28 (t, J=8.3 Hz, 1H,

pyrrolidine), 3.95 (t, J=8.9 Hz, 1H, pyrrolidine), 3,74 (s, 3H, OCH_3), 3.73 (s, 3H, OCH_3), 3.43 (t, J=8.7 Hz, 1H, pyrrolidine), 2.14 (s, 3H, NCH_3).

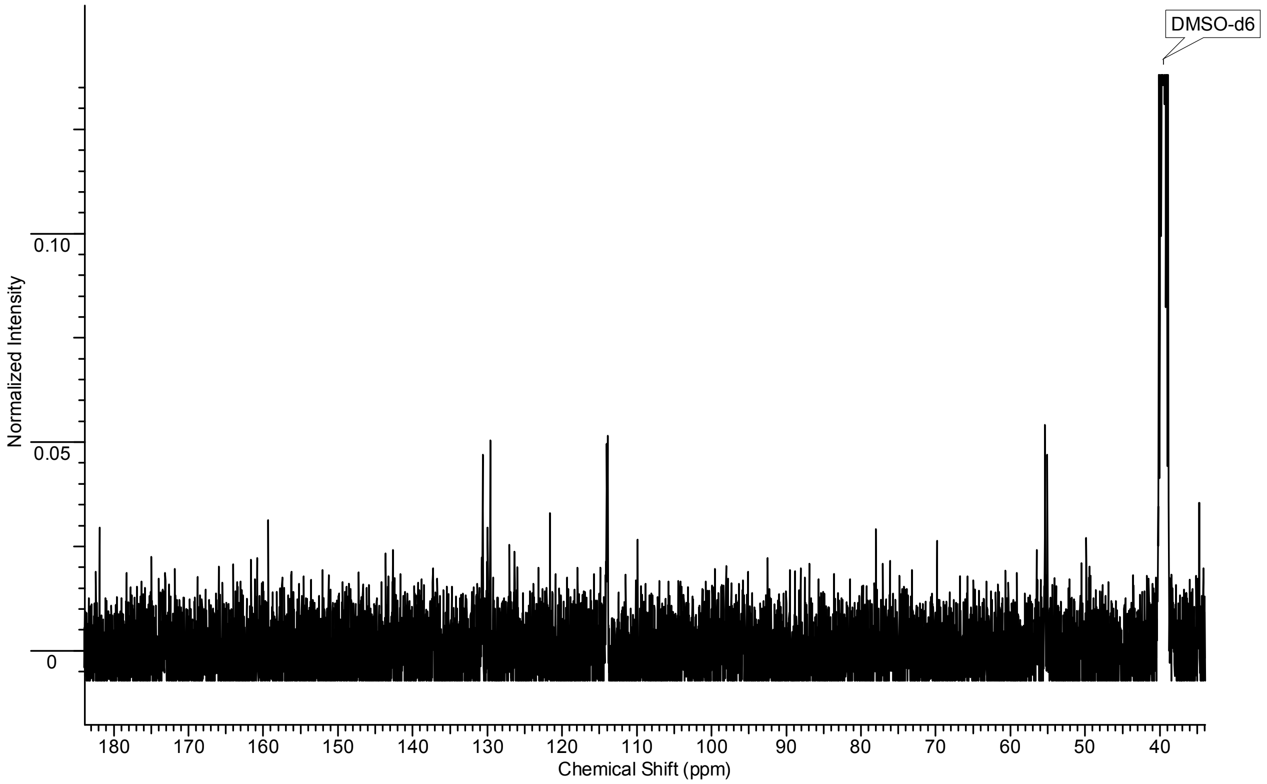

^{13}C NMR (101 MHz, DMSO-d6, δ, ppm):181.9, 175.0, 171.8, 159.4, 142.6, 133.3, 132.5, 130.6, 130.0, 129.6, 127.1, 126.4, 125.6, 123.5, 121.6, 114.0, 113.9, 109.9, 78.0, 76.9, 56.5, 55.4, 55.1, 34.8.

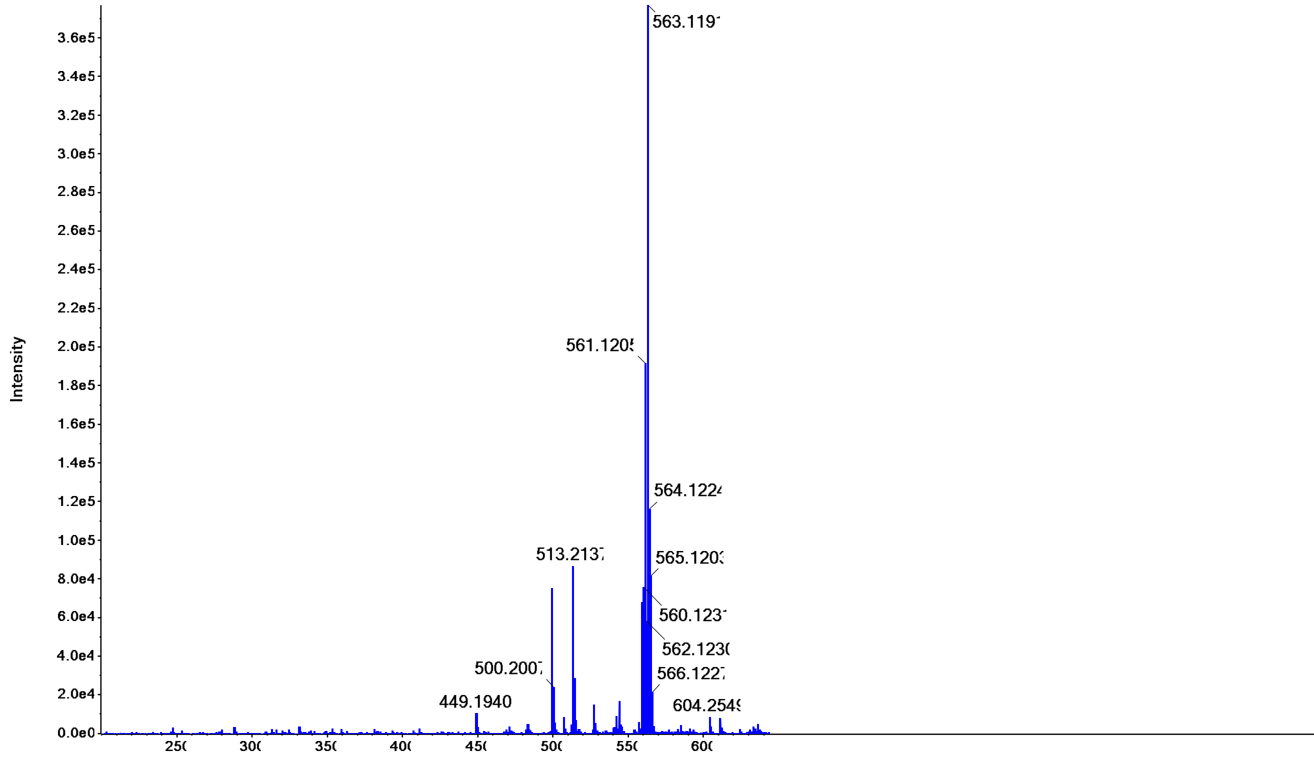

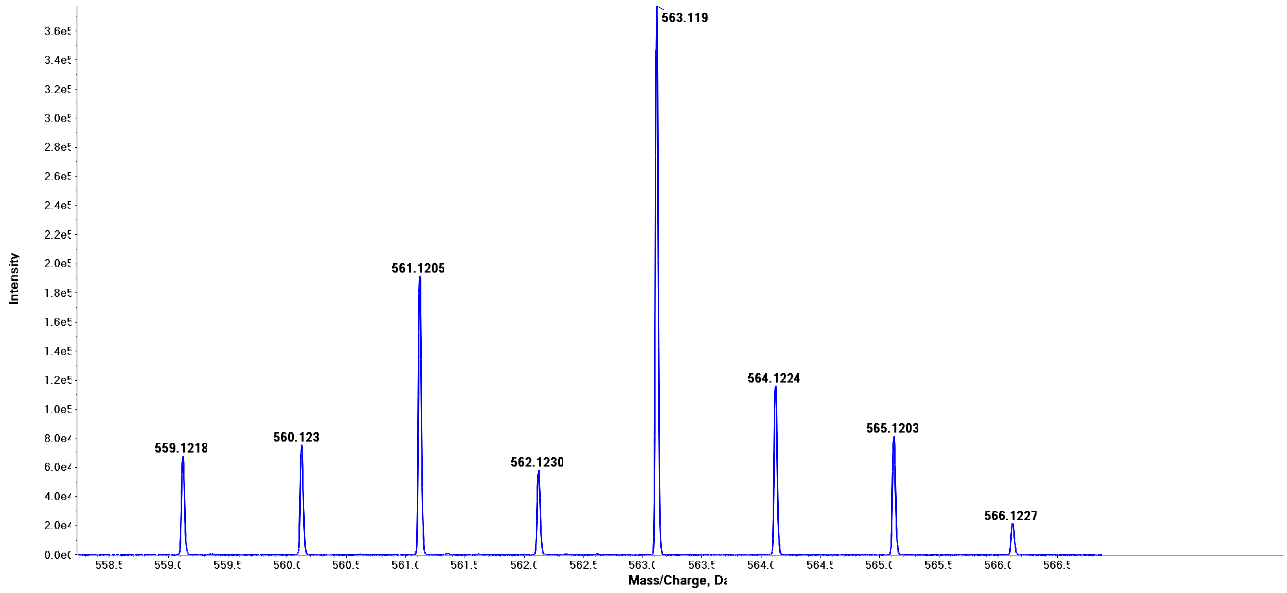

HRMS (ESI, m/Z): calculated ($C_{28}H_{26}N_4O_4Se$, M+H): 562.1119, found (M+H): 562.1191.

Synthesis of dispiroindolinones of type I (compounds 6a-m; general procedure). Toluene is added to 5-indolinylidene-2-selenoxohydantoin (1 equiv.) and sarcosine (8 equiv.) and the mixture was brought to a boil. Paraform (8 eq.) was then added. The reaction mixture was refluxed for 6 hours, then evaporated and the residue was purified by column chromatography (MeOH : $CHCl_3$ = 1:50).

1'-methyl-3-(4-ethoxyphenyl)-2-selenoxodispiro[imidazolidine-5,3'-pyrrolidine-4',3''-indoline]-2'',4-dione (**6a**)

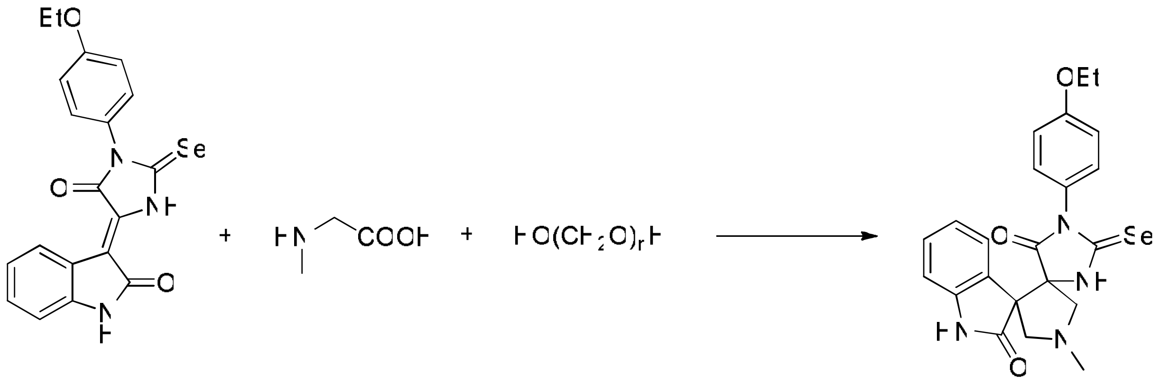

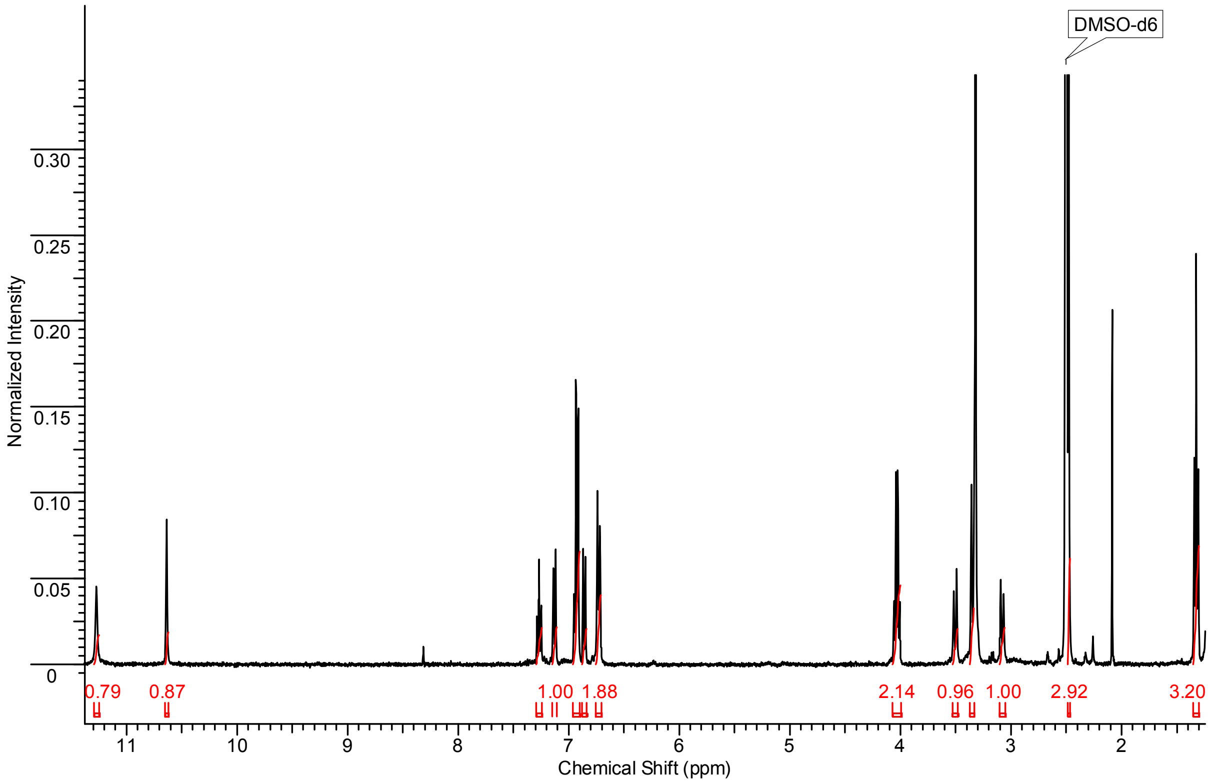

^{1}H NMR (400 MHz, DMSO-d6, δ, ppm):11.27 (s, 1H, NH), 10.63 (s, 1H, NH), 7.27 (t, J=7.8Hz, 1H, isatin), 7.13 (d, J=7.5Hz, 1H, isatin), 6.95-6.92 (m, 1H, isatin), 6.93 (d, J=8.6Hz, 2H, Ar), 6.86 (d, J=7.6Hz, 1H, isatin), 6.73 (d, J=8.6Hz, 2H, Ar), 4.03 (q, J=7.0Hz, 2H, ArO<u>CH$_2$</u>CH$_3$), 3.50 (d, J=10.0Hz, 1H, pyrrolidine), 3.34 (m, 1H, pyrrolidine), 3.33-3.29 (m, 1H, pyrrolidine), 3.08 (d, J=10.1Hz, 1H, pyrrolidine), 2.48 (s, 3H, NCH$_3$), 1.32 (t, J=6.9Hz, 3H, ArOCH$_2$<u>CH$_3$</u>).

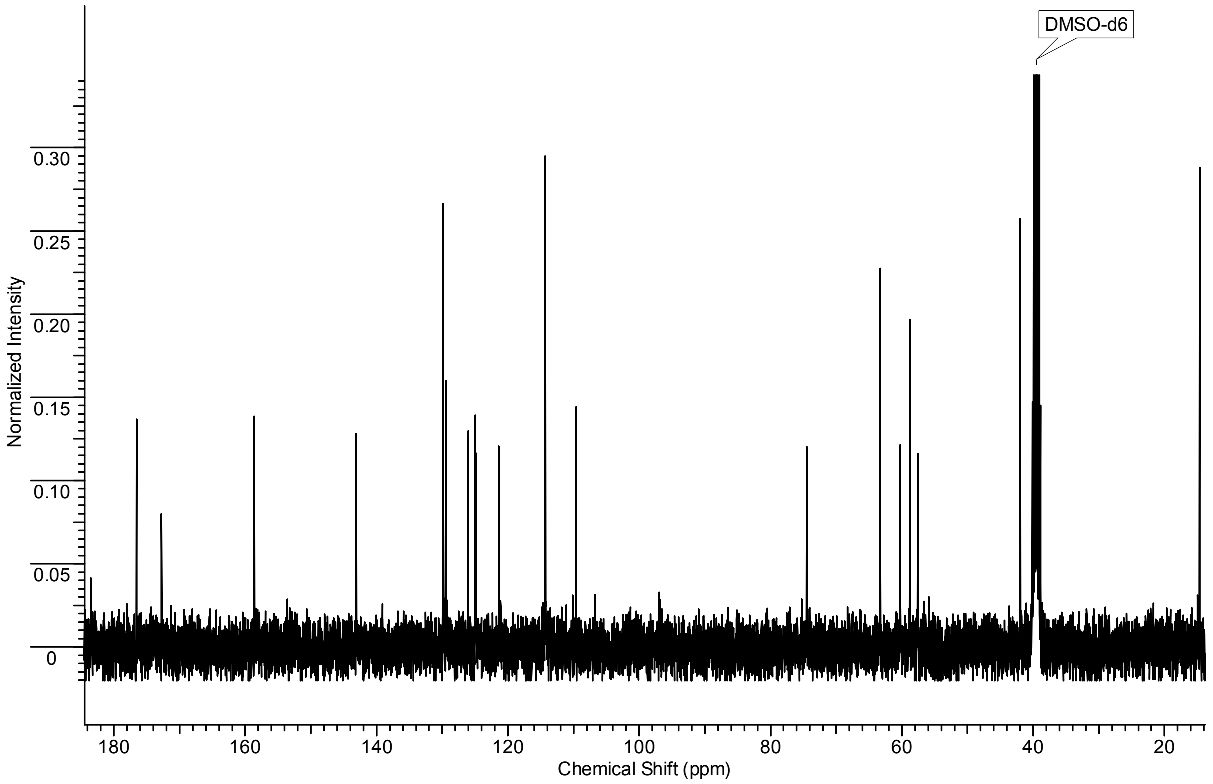

^{13}C NMR (101 MHz, DMSO-d6, δ, ppm): 183.5, 176.6, 172.7, 158.6, 143.0, 129.9, 129.5, 126.0, 125.0, 124.9, 121.4, 114.3, 109.6, 74.5, 63.3, 60.3, 58.8, 57.6, 42.0, 14.6.

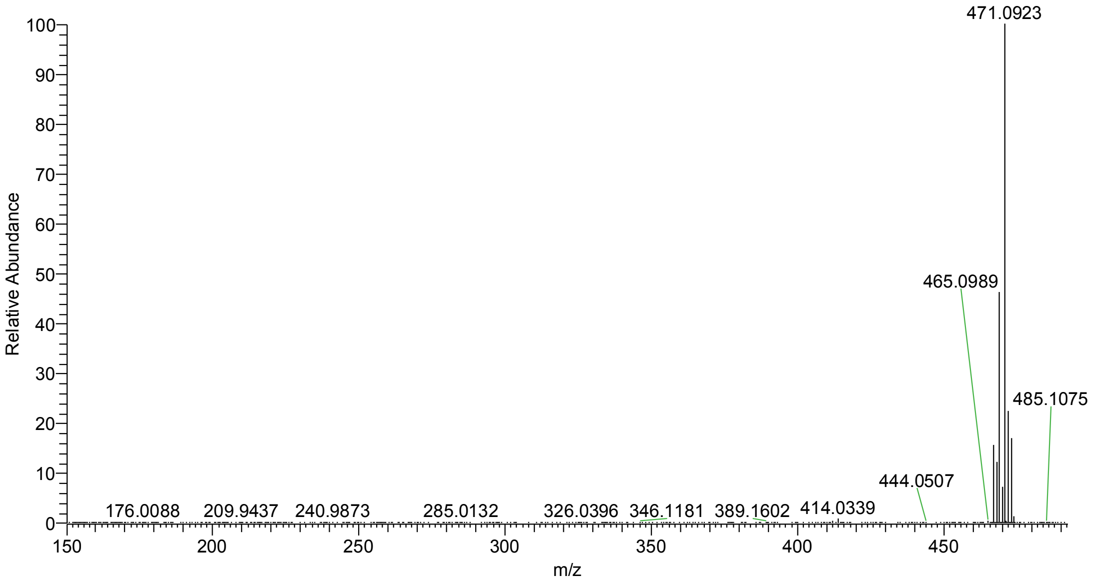

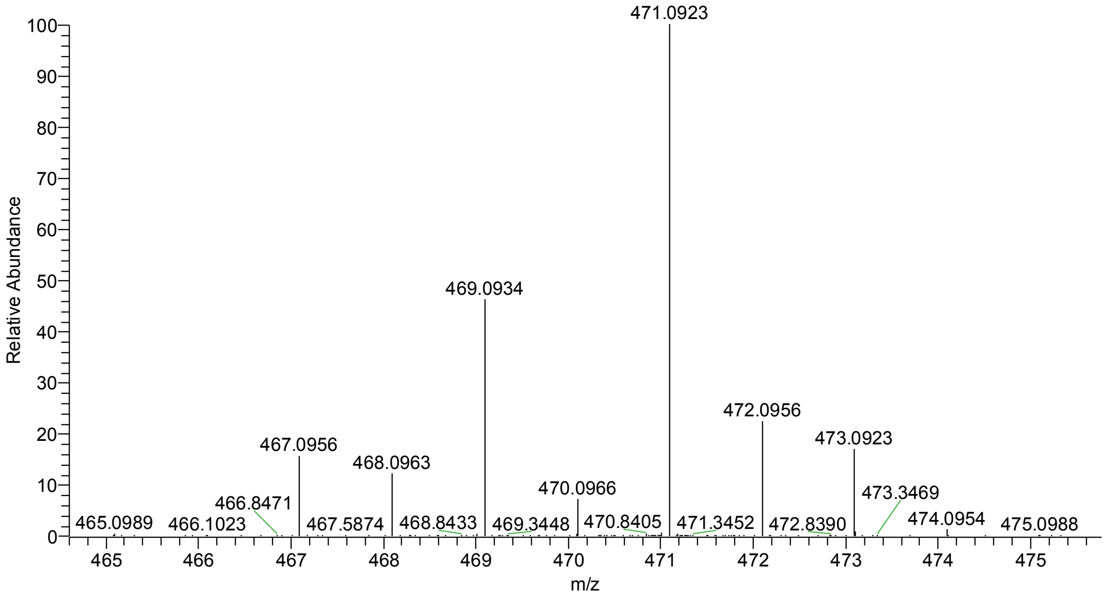

HRMS (ESI, m/Z): calculated ($C_{22}H_{22}N_4O_3Se$, M+H): 471.0930, found (M+H): 471.0923.

5''-chloro-1'-methyl-3-(4-ethoxyphenyl)-2-selenoxodispiro[imidazolidine-5,3'-pyrrolidine-4',3''-indoline]-2'',4-dione (6b)

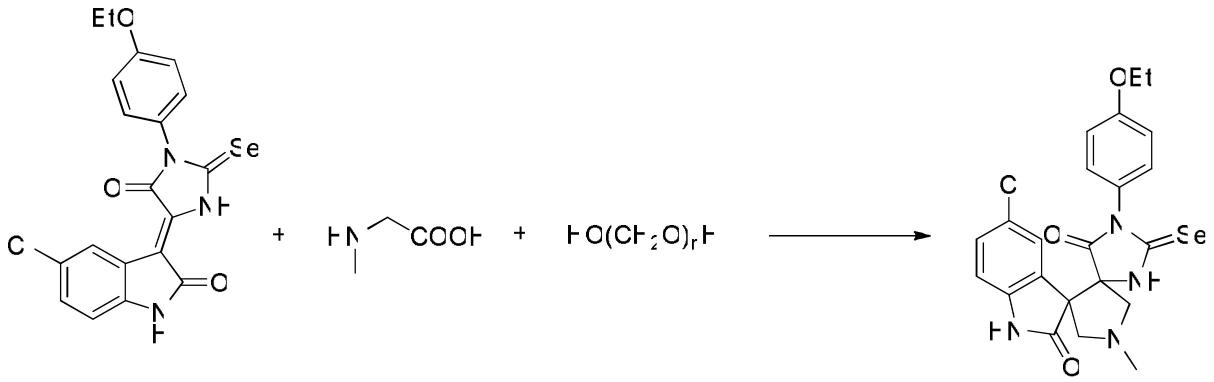

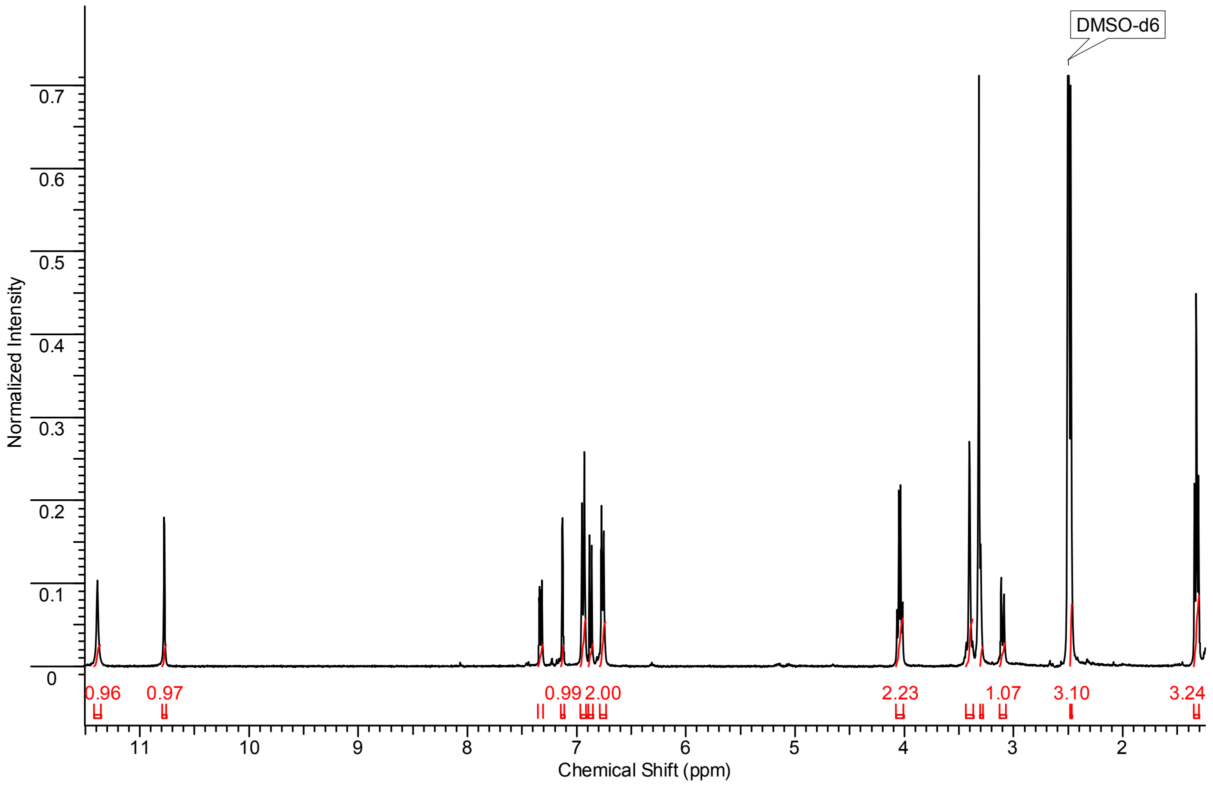

^{1}H NMR (400 MHz, DMSO-d6, δ, ppm):11.39 (s, 1H, NH), 10.78 (s, 1H, NH), 7.33 (dd, J_1=2.0Hz, J_2=8.3Hz, 1H, isatin), 7.13 (d, J=1.8Hz, 1H, isatin), 6.94 (d, J=8.8Hz, 2H, Ar), 6.87 (d, J=8.3Hz, 1H, isatin), 6.76 (d, J=8.6Hz, 2H, Ar), 4.04 (q, J=6.9Hz, 2H, ArO<u>CH_2</u>CH_3), 3.42 (d, J=11.7Hz, 1H, pyrrolidine), 3.39 (d, J=10.7Hz, 1H, pyrrolidine), 3.29 (m, 1H, pyrrolidine), 3.10 (d, J=10.1Hz, 1H, pyrrolidine), 2.48 (s, 3H, NCH_3), 1.33 (t, J=6.9Hz, 3H, $ArOCH_2$<u>CH_3</u>).

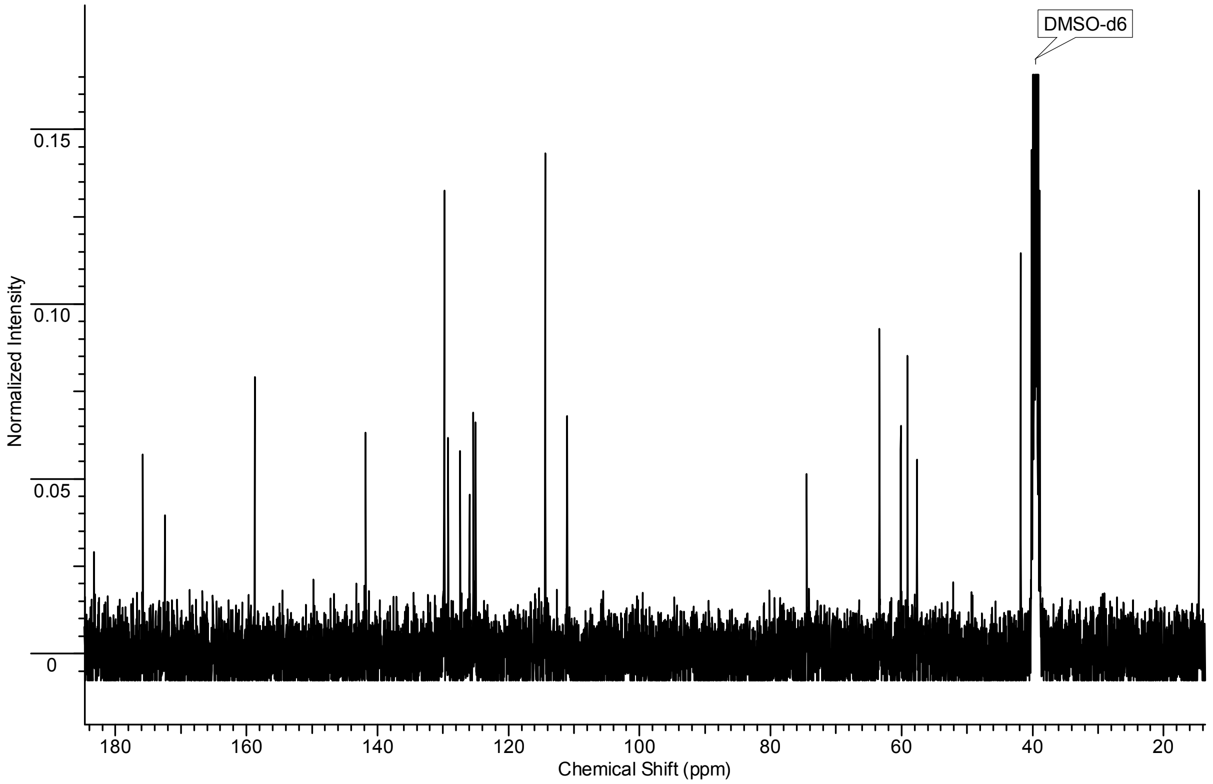

^{13}C NMR (101 MHz, DMSO-d6, δ, ppm): 183.3, 175.8, 172.5, 158.7, 141.8, 129.8, 129.2, 127.4, 126.0, 125.4, 125.0, 114.4, 111.0, 74.5, 63.4, 60.1, 59.1, 57.6, 41.8, 14.6.

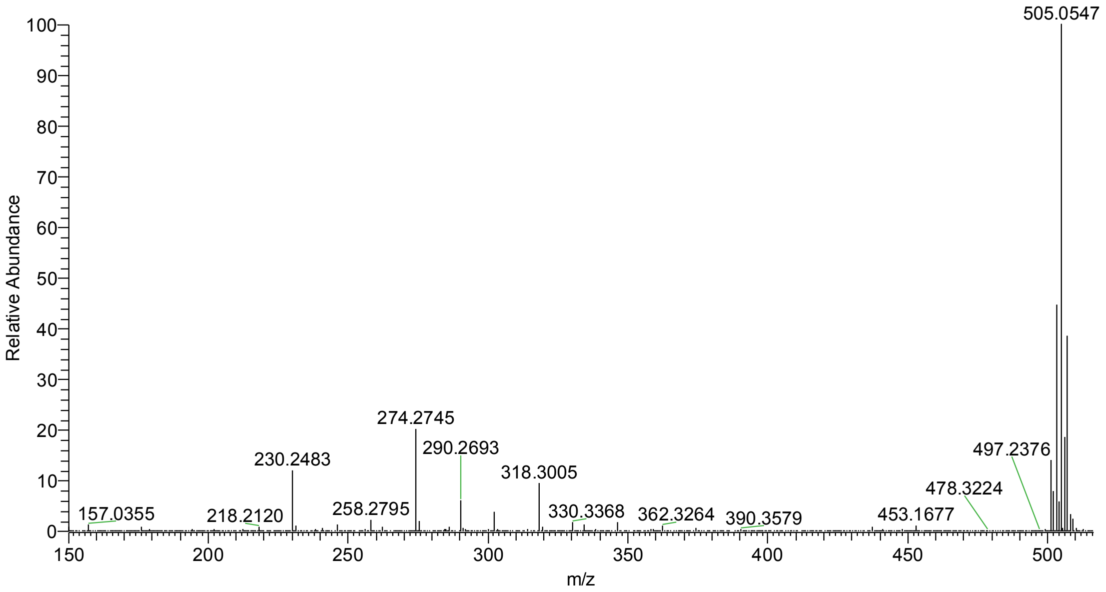

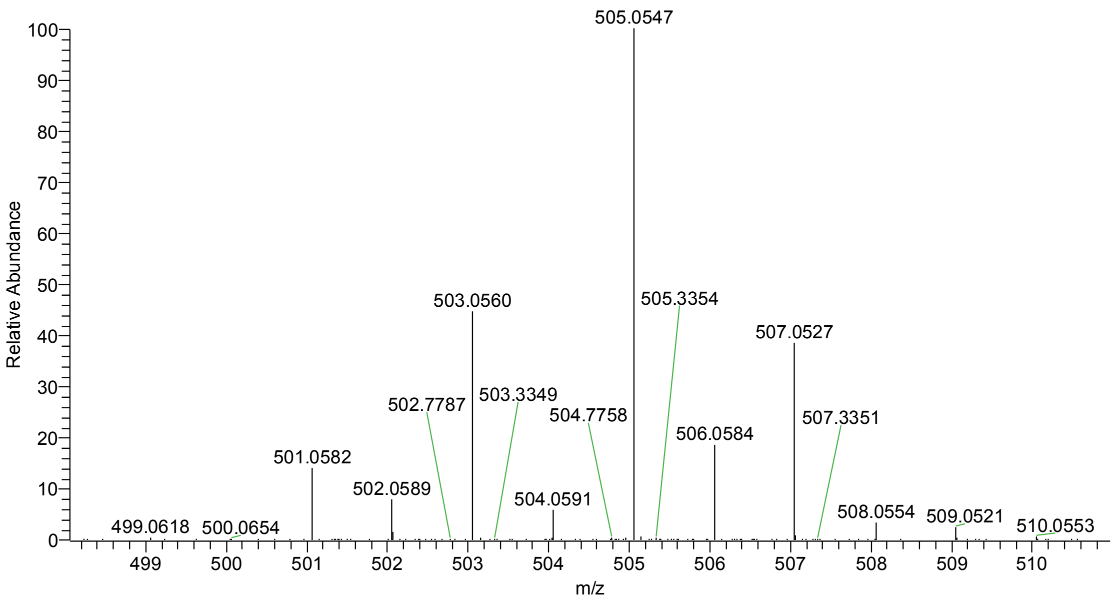

HRMS (ESI, m/Z): calculated ($C_{22}H_{21}ClN_4O_3Se$, M+H): 505.0540, found (M+H): 505.0547.

5’’-bromo-1'-methyl-3-(4-ethoxyphenyl)-2-selenoxodispiro[imidazolidine-5,3'-pyrrolidine-4',3''-indoline]-2'',4-dione (6c)

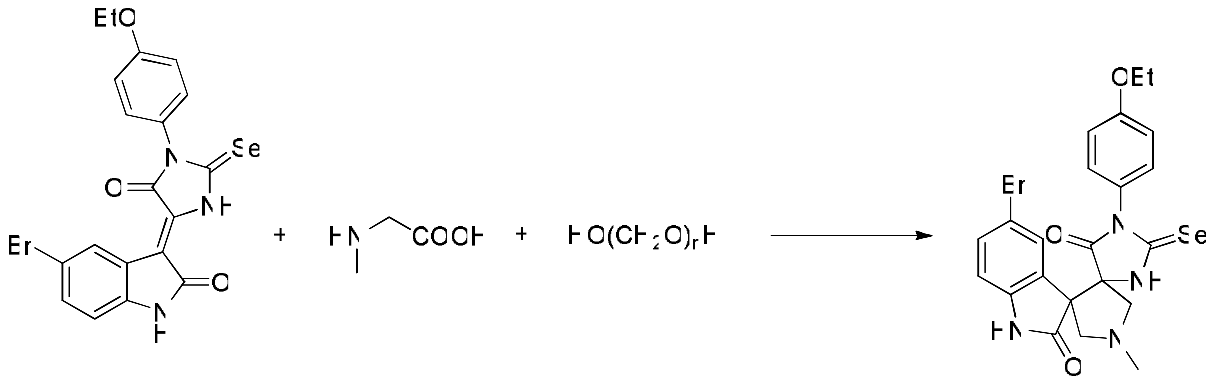

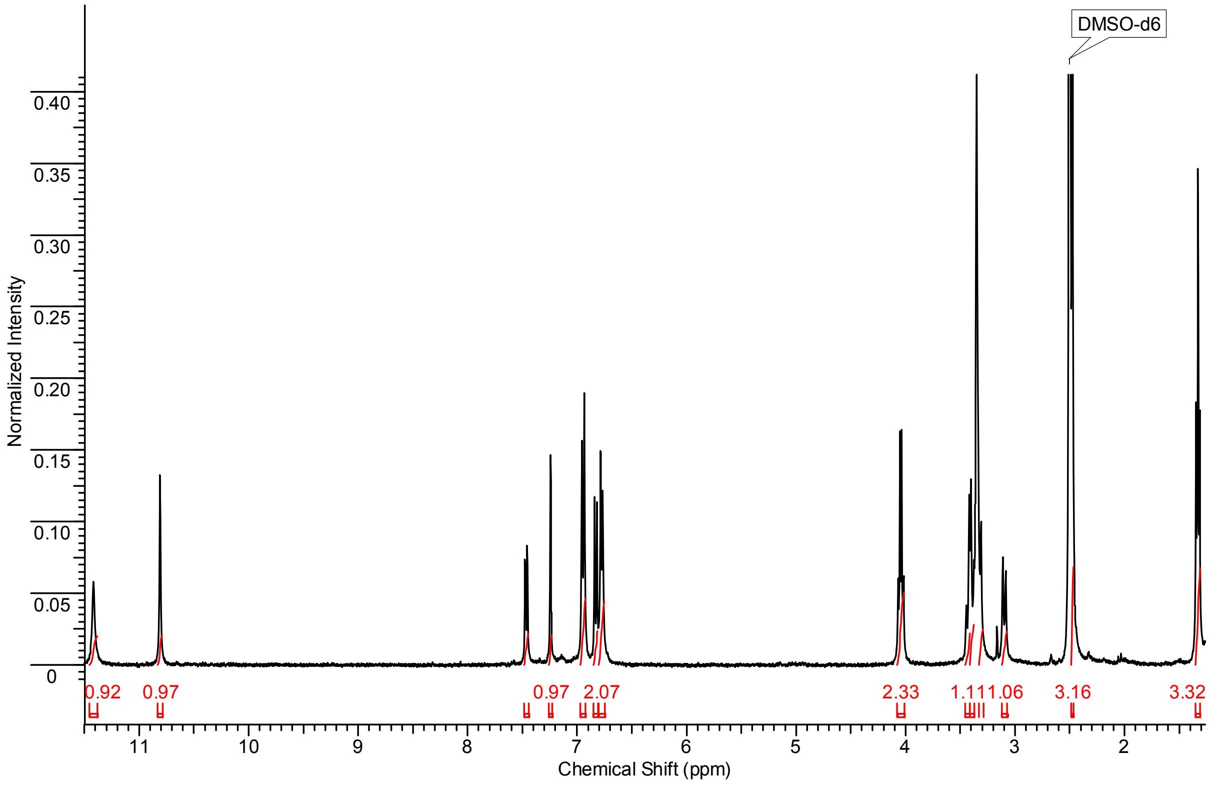

^{1}H NMR (400 MHz, DMSO-d6, δ, ppm):11.42 (s, 1H, NH), 10.81(s, 1H, NH), 7.46 (dd, J_1=1.4Hz, J_2=8.3Hz, 1H, isatin), 7.24 (s, 1H, isatin), 6.94 (d, J=8.6Hz, 2H, Ar), 6.83 (d, J=8.3Hz, 1H, isatin), 6.77 (d, J=8.4Hz, 2H, Ar), 4.04 (q, J=6.9Hz, 2H, ArO$\underline{CH_2}$$CH_3$), 3.43 (d, J=10.3Hz, 1H, pyrrolidine), 3.39 (d, J=10.5Hz, 1H, pyrrolidine), 3.32 (m, 1H, pyrrolidine), 3.10 (d, J=10.1Hz, 1H, pyrrolidine), 2.48 (s, 3H, NCH_3), 1.33 (t, J=6.9Hz, 3H, ArO$CH_2$$\underline{CH_3}$).

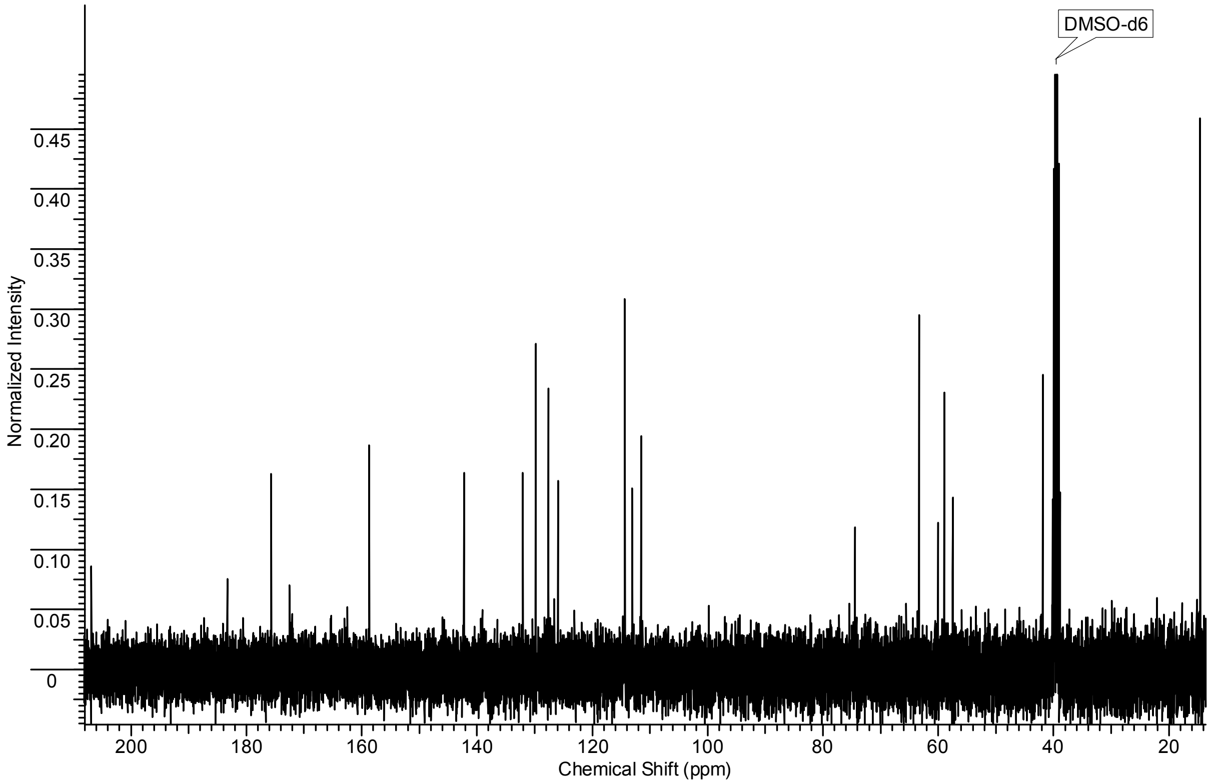

^{13}C NMR (101 MHz, DMSO-d6, δ, ppm):206.9, 183.3, 175.7, 172.5, 158.7, 142.3, 142.2, 132.0, 129.8, 127.7, 125.9, 114.4, 113.1, 111.5, 74.4, 63.3, 60.0, 59.0, 57.5, 41.9, 14.6.

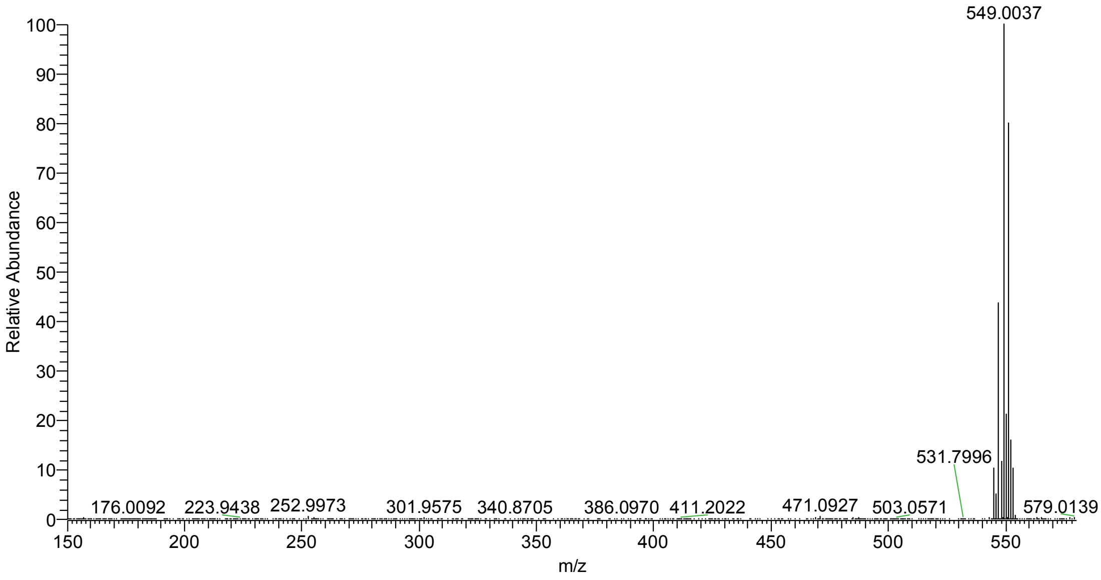

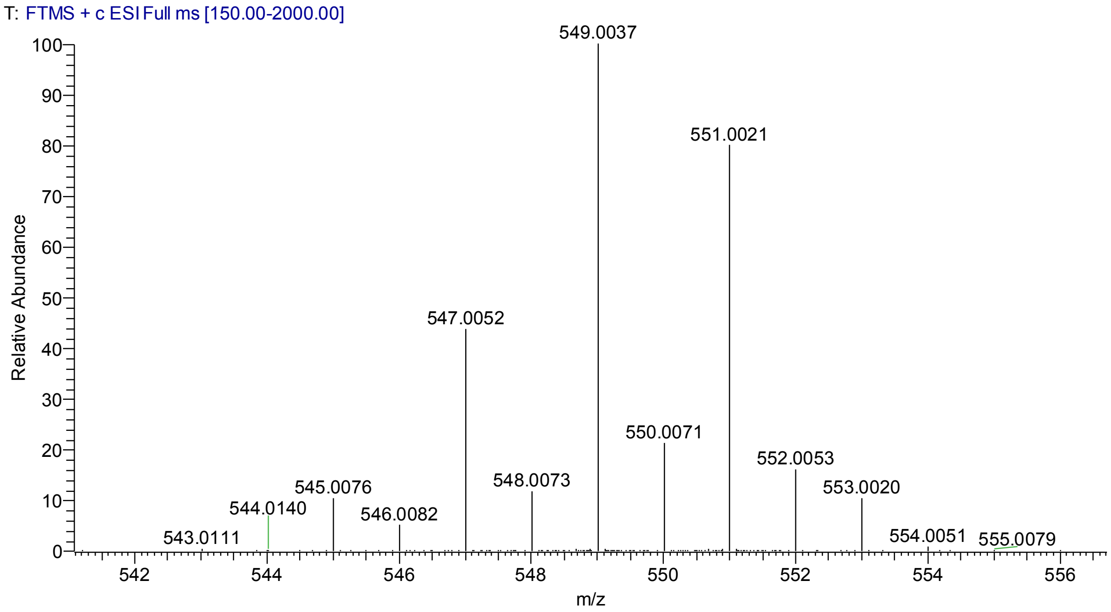

HRMS (ESI, m/Z): calculated ($C_{22}H_{21}BrN_4O_3Se$, M+H): 549.0035, found (M+H): 549.0037.

5’’-nitro-1'-methyl-3-(4-ethoxyphenyl)-2-selenoxodispiro[imidazolidine-5,3'-pyrrolidine-4',3''-indoline]-2'',4-dione (6d)

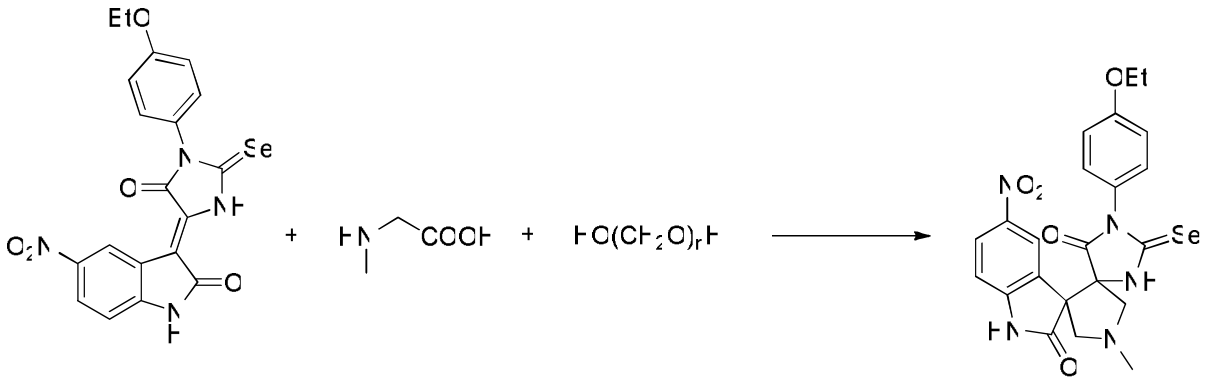

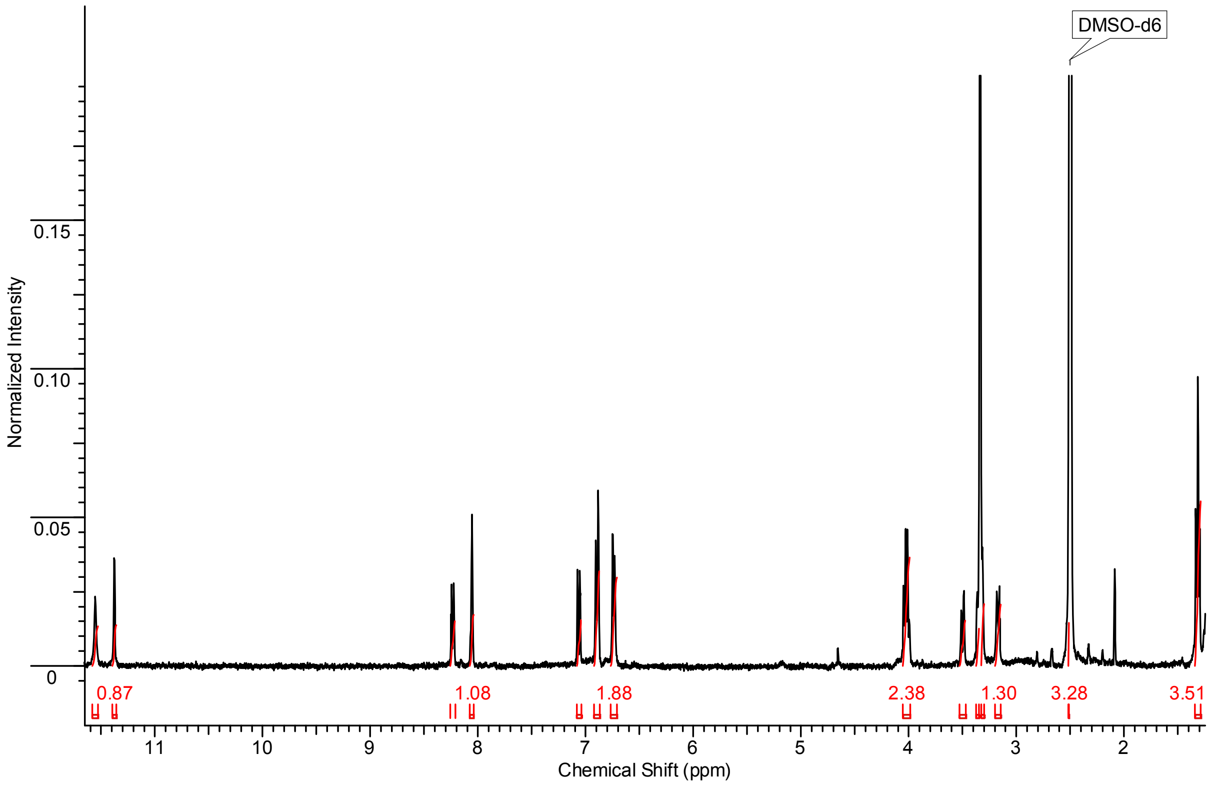

^{1}H NMR (400 MHz, DMSO-d6, δ, ppm):11.55 (s, 1H, NH), 11.38 (s, 1H, NH), 8.23 (dd, J_1=2.1Hz, J_2=8.7Hz, 1H, isatin), 8.06 (s, 1H, isatin), 7.06 (dd, J_1=1.6Hz, J_2=8.6Hz, 1H, isatin), 6.89 (d, J=8.9Hz, 2H, Ar), 6.74 (d, J=8.6Hz, 2H, Ar), 4.02 (q, J=6.6Hz, 2H, ArO$\underline{CH_2}$CH$_3$), 3.50 (d, J=10.9Hz, 1H, pyrrolidine), 3.38-3.30 (m, 2H, pyrrolidine), 3.17 (d, J=9.6Hz, 1H, pyrrolidine), 2.50 (s, 3H, NCH$_3$), 1.32 (t, J=6.9 Hz, 3H, ArOCH$_2$$\underline{CH_3}$).

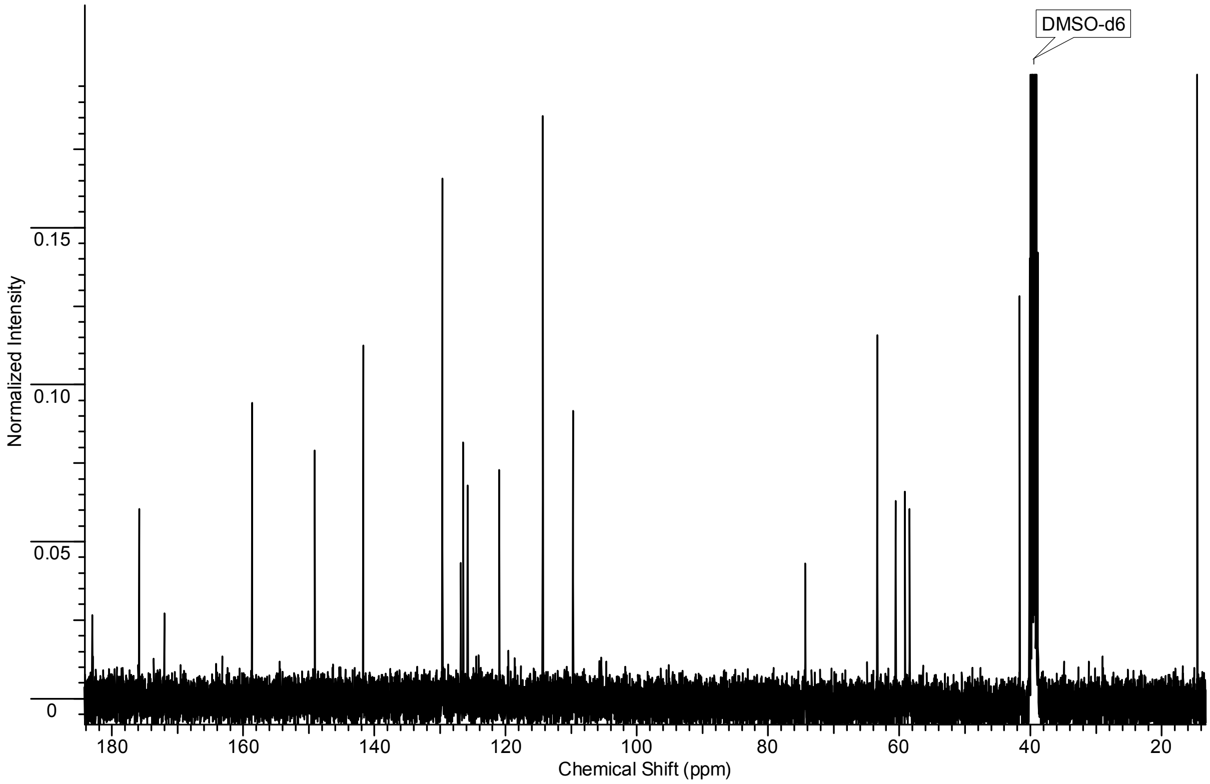

^{13}C NMR (101 MHz, DMSO-d6, δ, ppm): 183.0, 175.9, 172.0, 158.6, 149.1, 141.7, 129.6, 126.9, 126.5, 125.8, 121.0, 114.3, 109.8, 74.3, 63.3, 60.6, 59.1, 58.4, 41.6, 14.6.

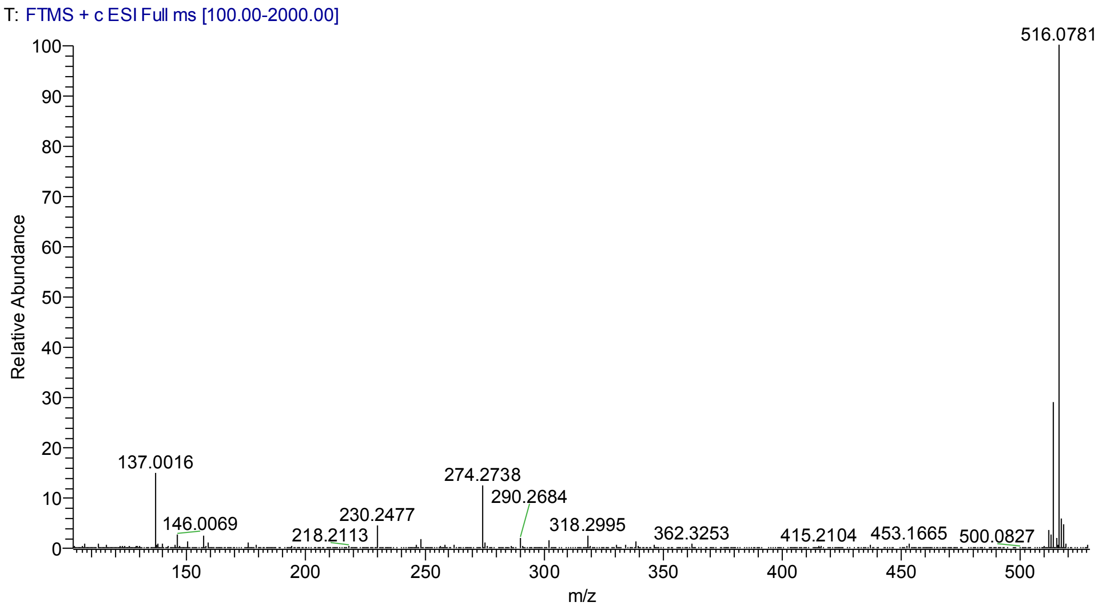

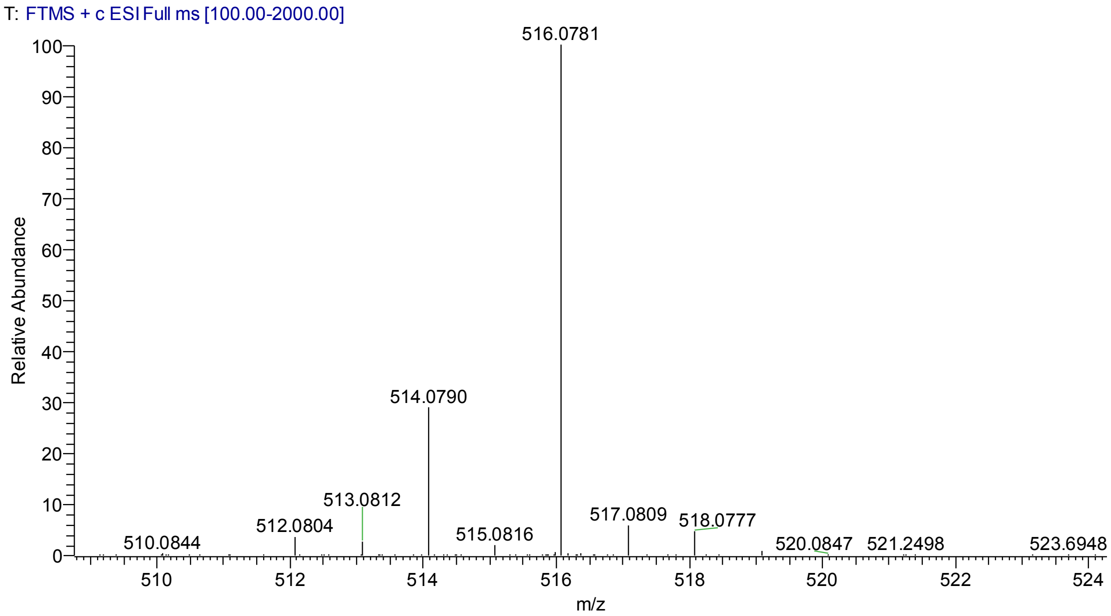

HRMS (ESI, m/Z): calculated ($C_{22}H_{21}N_5O_5Se$, M+H): 516.0781, found (M+H): 516.0781.

5''-bromo-1'-methyl-3-(4-methoxyphenyl)-2-selenoxodispiro[imidazolidine-5,3'-pyrrolidine-4',3''-indoline]-2'',4-dione (6e)

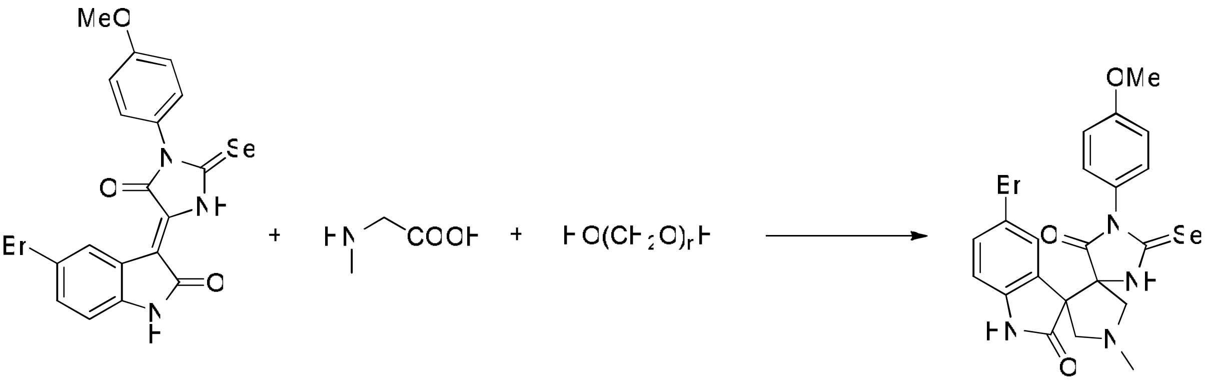

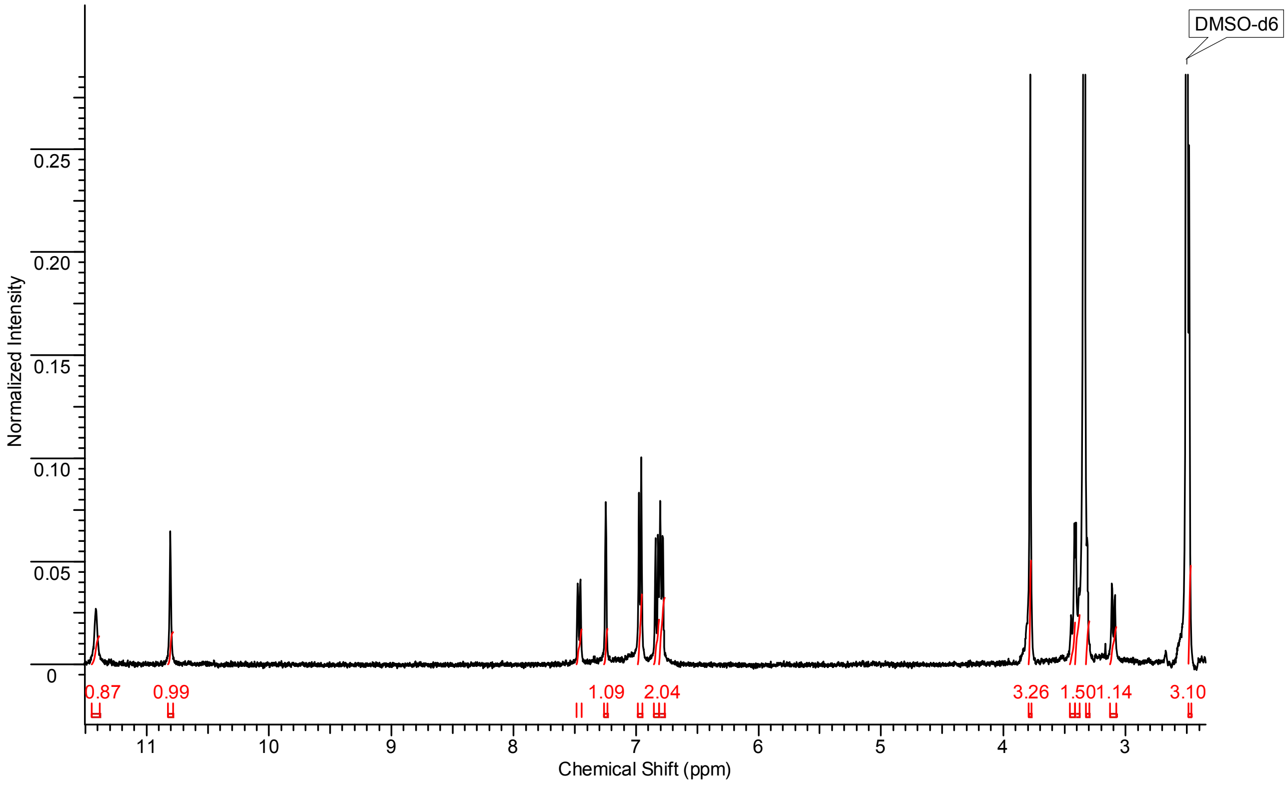

^{1}HNMR (400 MHz, DMSO-d6, δ, ppm):11.41 (s, 1H, NH), 10.81 (s, 1H, NH), 7.47 (d, J=8.3Hz, 1H, isatin), 7.25 (s, 1H, isatin), 6.97 (d, J=8.4Hz, 2H, Ar), 6.83 (d, J=8.4Hz, 1H, isatin), 6.79 (d, J=8.5Hz, 2H, Ar), 3.78 (s, 3H, OCH_3), 3.44 (d, J=10.1Hz, 1H, pyrrolidine), 3.39 (d, J=10.4Hz, 1H, pyrrolidine), 3.31 (m, 1H, pyrrolidine), 3.10 (d, J=10.5Hz, 1H, pyrrolidine), 2.48 (s, 3H, NCH_3).

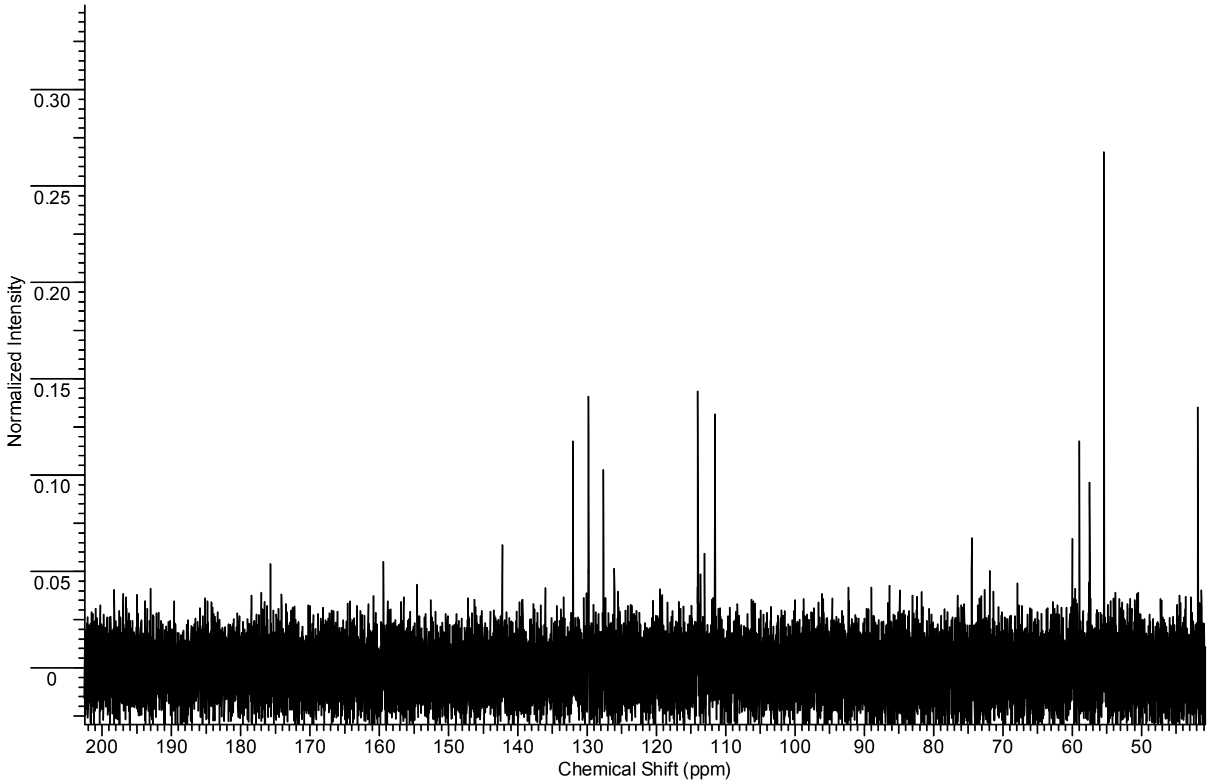

^{13}CNMR (101 MHz, DMSO-d6, δ, ppm): 206.9, 184.2, 175.7, 172.5, 159.4, 142.3, 132.0, 129.8, 127.7, 126.1, 114.0, 113.1, 111.5, 74.5, 63.7, 60.0, 59.0, 57.5, 55.4, 41.9.

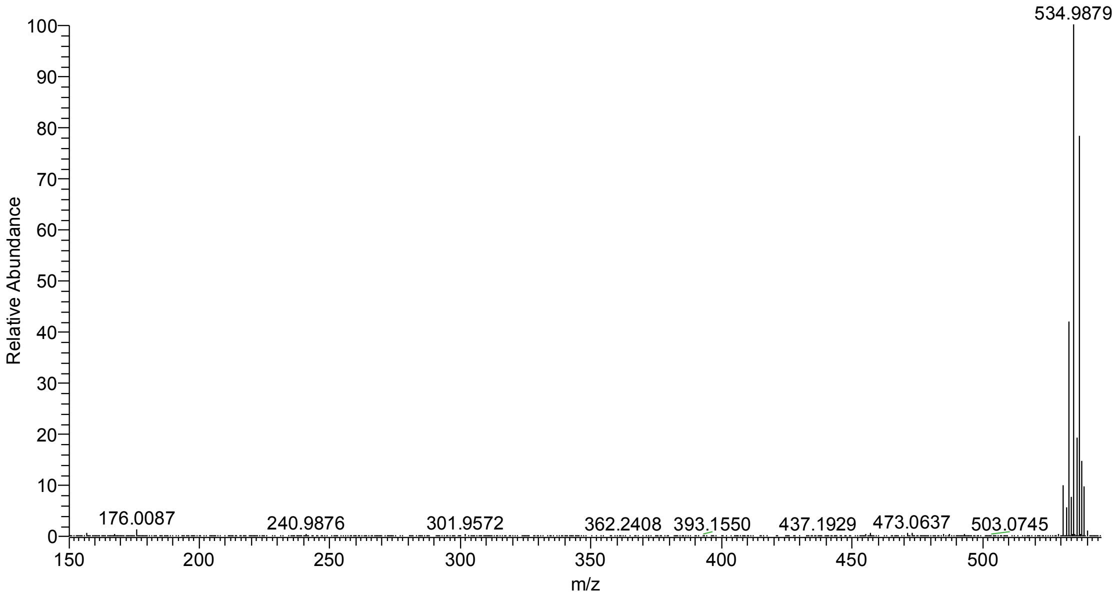

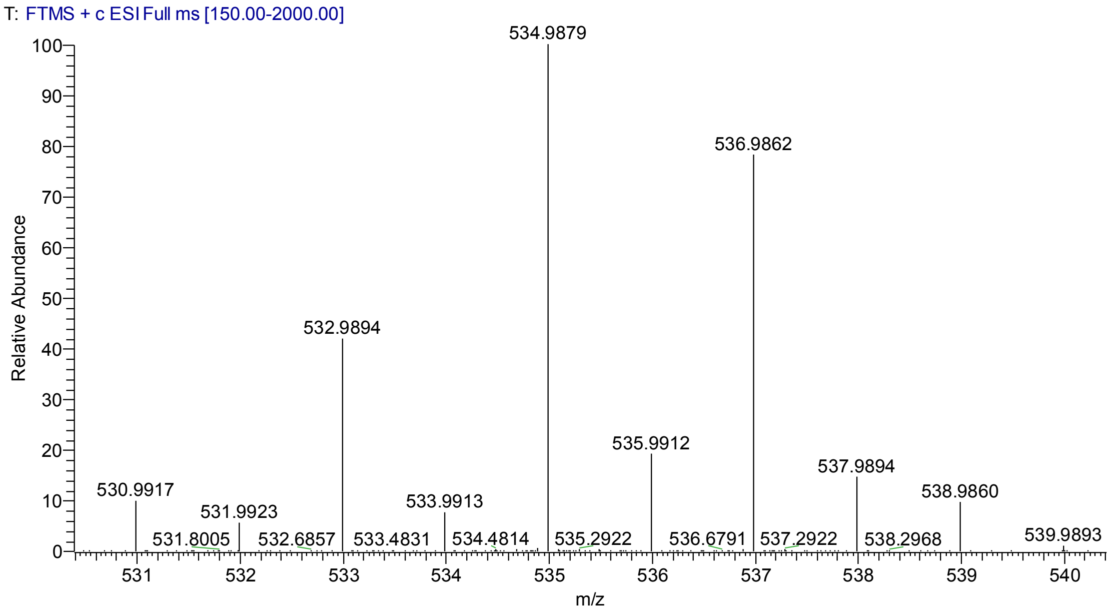

HRMS (ESI, m/Z): calculated ($C_{21}H_{19}BrN_4O_3Se$, M+H): 534.9876, found (M+H): 534.9879.

5''-nitro-1'-methyl-3-(4-methoxyphenyl)-2-selenoxodispiro[imidazolidine-5,3'-pyrrolidine-4',3''-indoline]-2'',4-dione (6f)

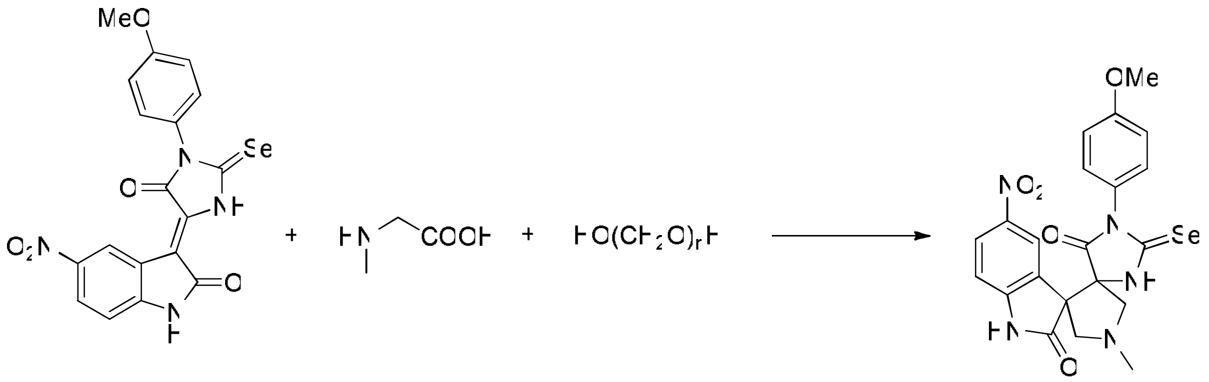

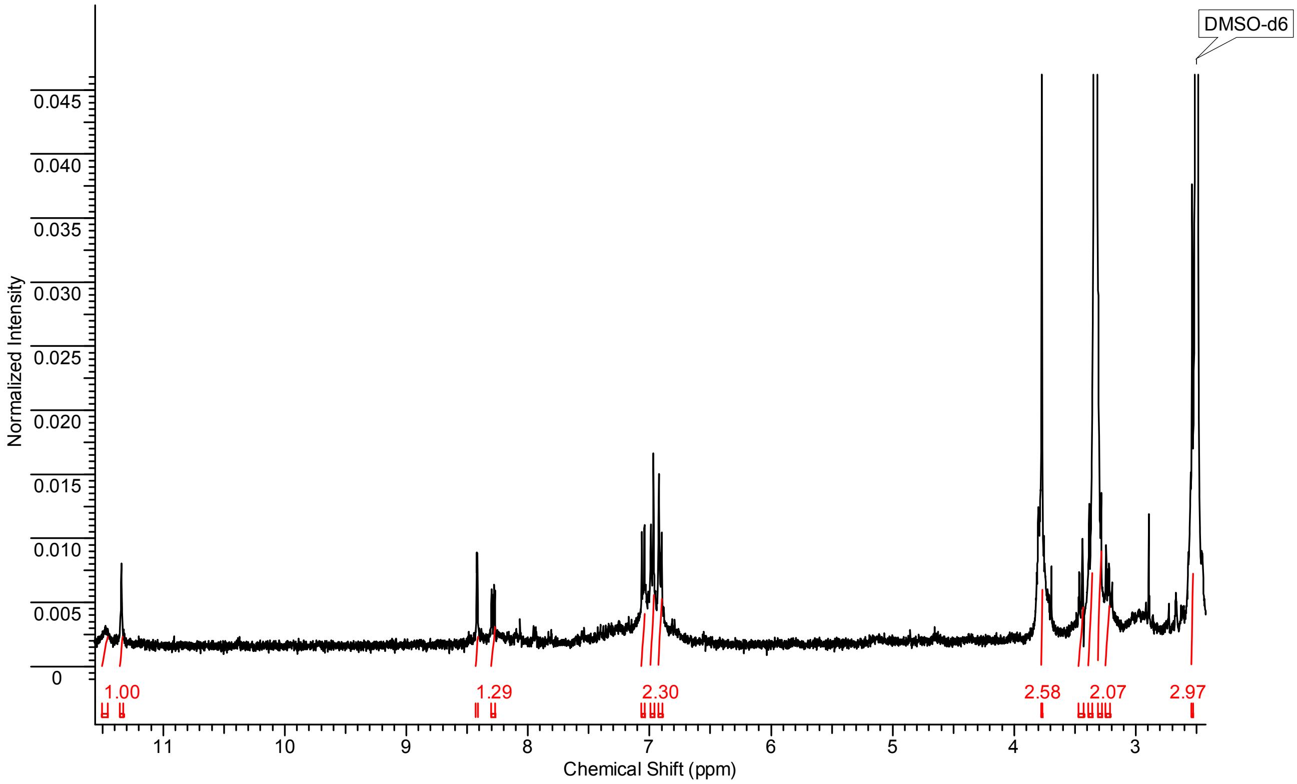

^{1}HNMR (400 MHz, DMSO-d6, δ, ppm):11.48 (bs, 1H, NH), 11.34 (s, 1H, NH), 8.42 (d, J=2.4Hz, 1H, isatin), 8.29 (dd, J_1=2.4Hz, J_2=8.6Hz, 1H, isatin), 7.05 (d, J=9.0Hz, 1H, isatin), 6.98 (d, J=9.0Hz, 2H, Ar), 6.91 (d, J=9.0Hz, 2H, Ar), 3.77 (s, 3H, OCH_3), 3.45 (d, J=11.0Hz, 1H, pyrrolidine), 3.37 (m, 1H, pyrrolidine), 3.30 (m, 1H, pyrrolidine), 3.23 (d, J=9.4Hz, 1H, pyrrolidine), 2.54 (s, 3H, NCH_3).

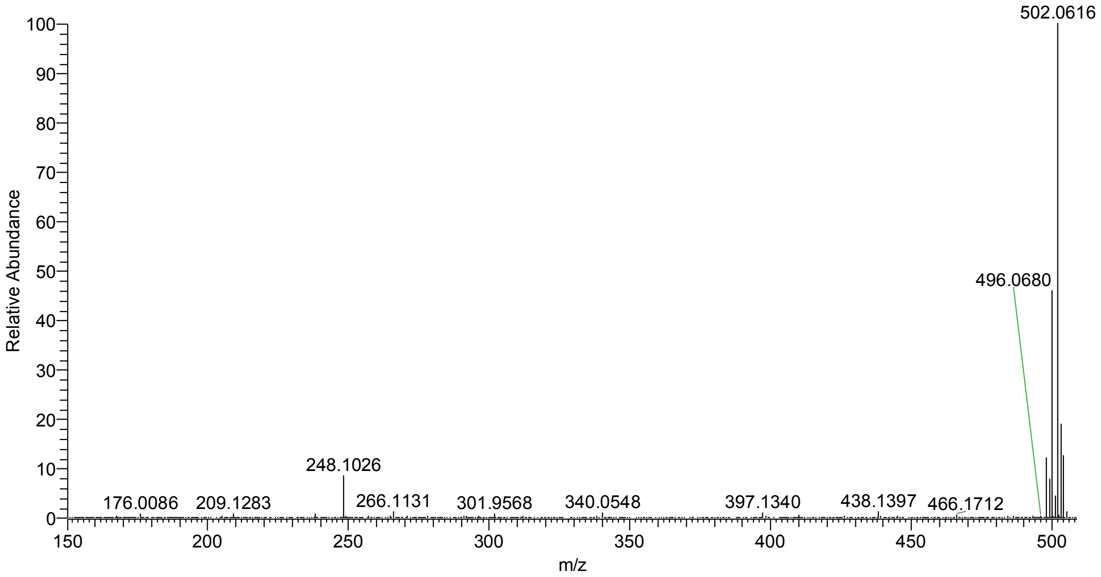

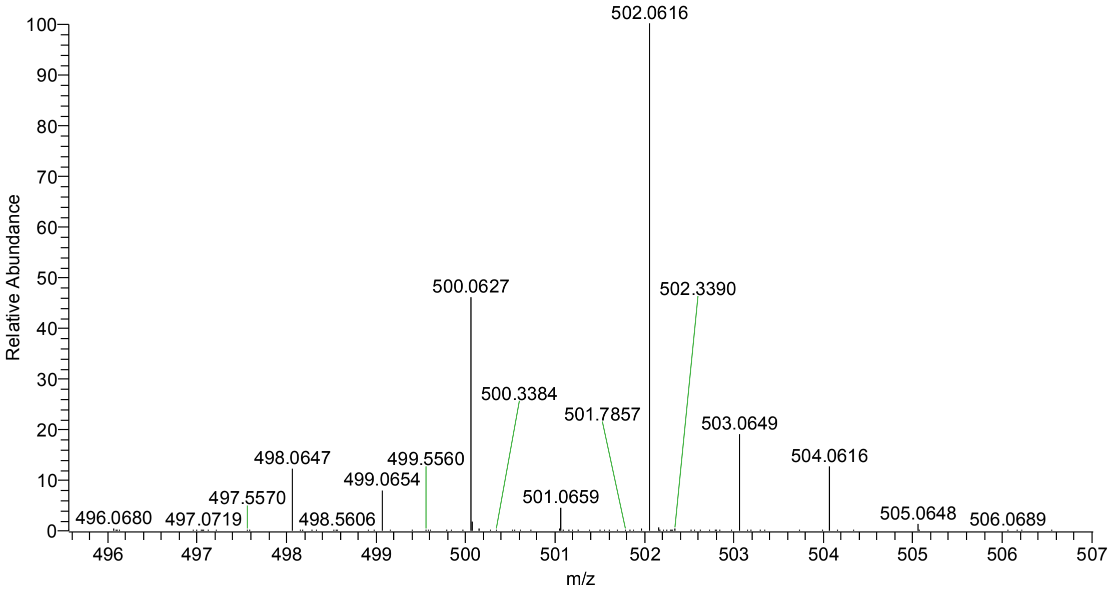

HRMS (ESI, m/Z): calculated ($C_{21}H_{19}N_5O_5Se$, M+H): 502.0624, found (M+H): 502.0616.

5’’-bromo-1'-methyl-3-(4-chlorophenyl)-2-selenoxodispiro[imidazolidine-5,3'-pyrrolidine-4',3''-indoline]-2'',4-dione (6g)

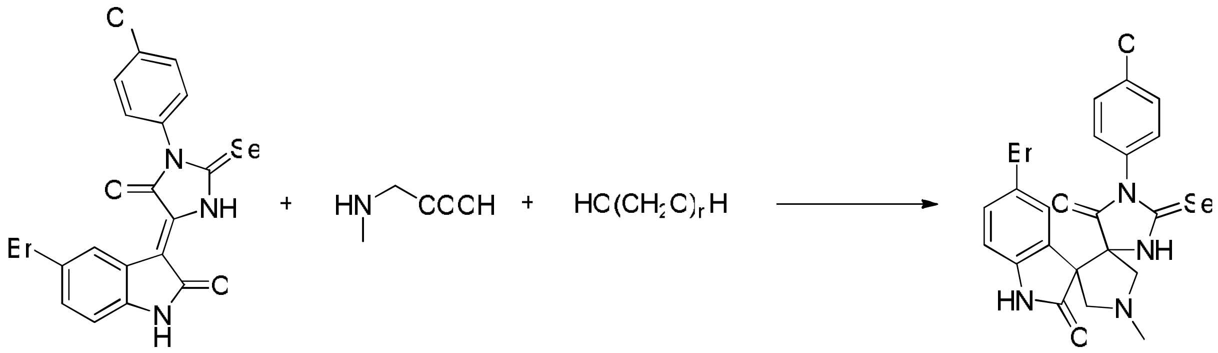

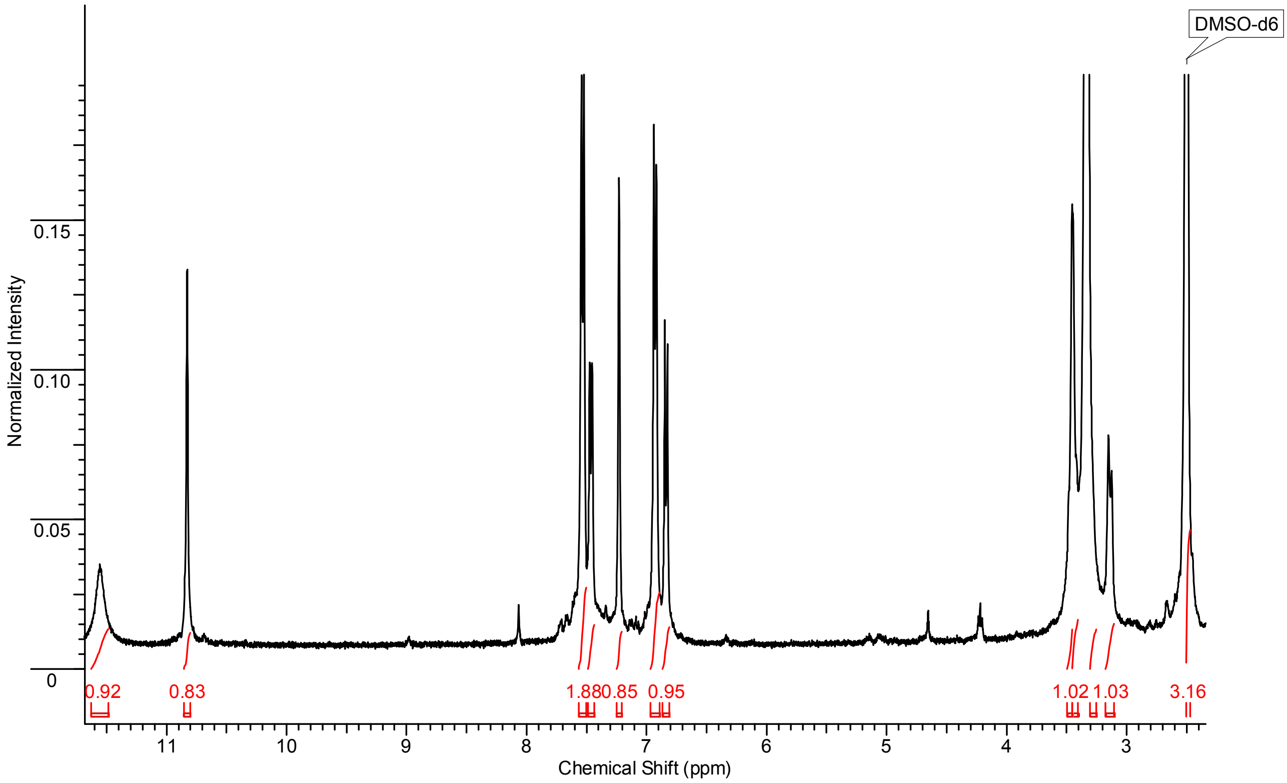

^{1}HNMR (400 MHz, DMSO-d6, δ, ppm):11.57 (s, 1H, NH), 10.83 (s, 1H, NH), 7.53 (d, J=8.8Hz, 2H, Ar), 7.46 (dd, J_1=1.8Hz, J=8.3Hz, 1H, isatin), 7.22 (s, 1H, isatin), 6.92 (d, J=8.5Hz, 2H, Ar), 6.83 (d, J=8.3Hz, 1H, isatin), 3.47 (d, J=9.8 Hz, 1H, pyrrolidine), 3.43 (d, J=10.2 Hz, 1H, pyrrolidine), 3.31-3.25(m, 1H, pyrrolidine), 3.14 (d, J=9.8 Hz, 1H, pyrrolidine), 2.49 (s, 3H, NCH_3).

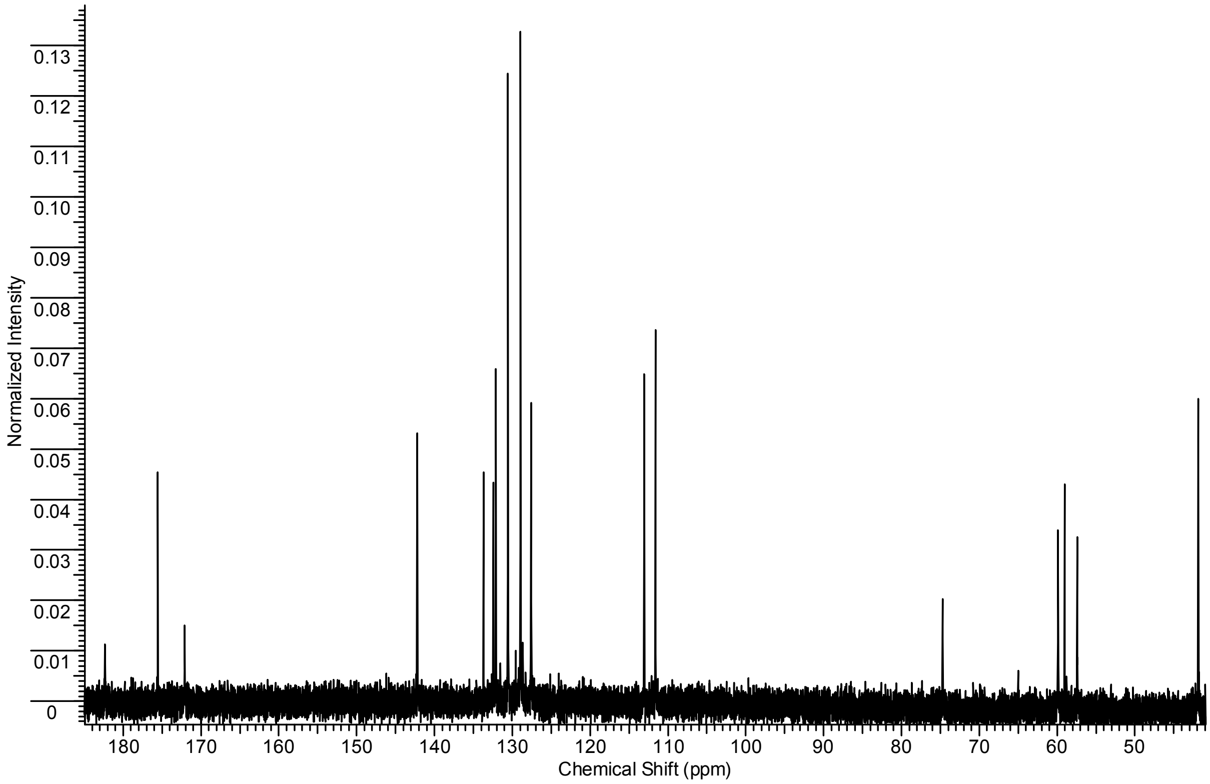

^{13}C NMR (101 MHz, DMSO-d6, δ, ppm): 182.3, 175.6, 172.1, 142.2, 133.7, 132.5, 132.1, 130.6, 128.9, 127.6, 127.5, 113.1, 111.6, 74.7, 59.9, 59.0, 57.4, 41.9.

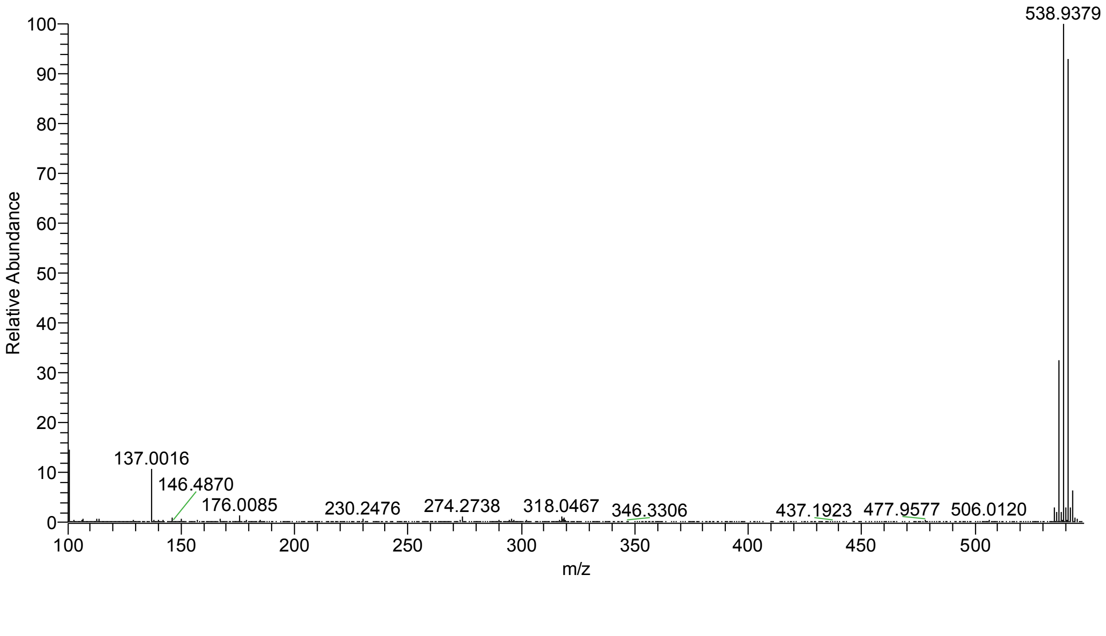

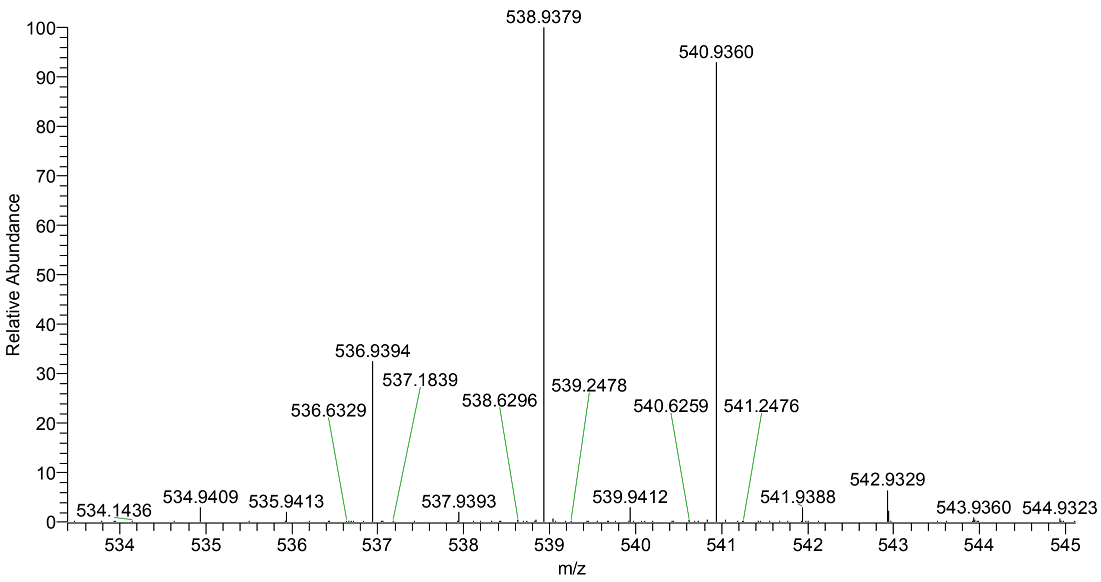

HRMS (ESI, m/Z): calculated ($C_{20}H_{16}BrClN_4O_2Se$, M+H): 538.9383, found (M+H): 538.9379.

5’’-nitro-1'-methyl-3-(4-chlorophenyl)-2-selenoxodispiro[imidazolidine-5,3'-pyrrolidine-4',3''-indoline]-2'',4-dione (6h)

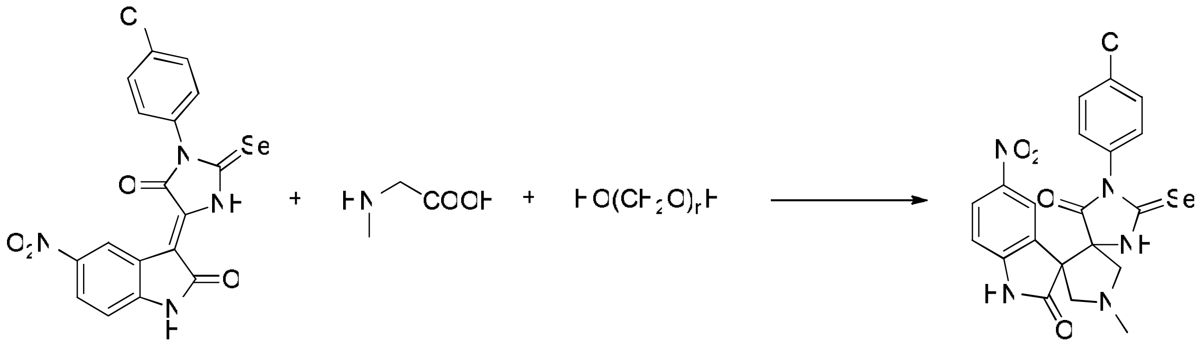

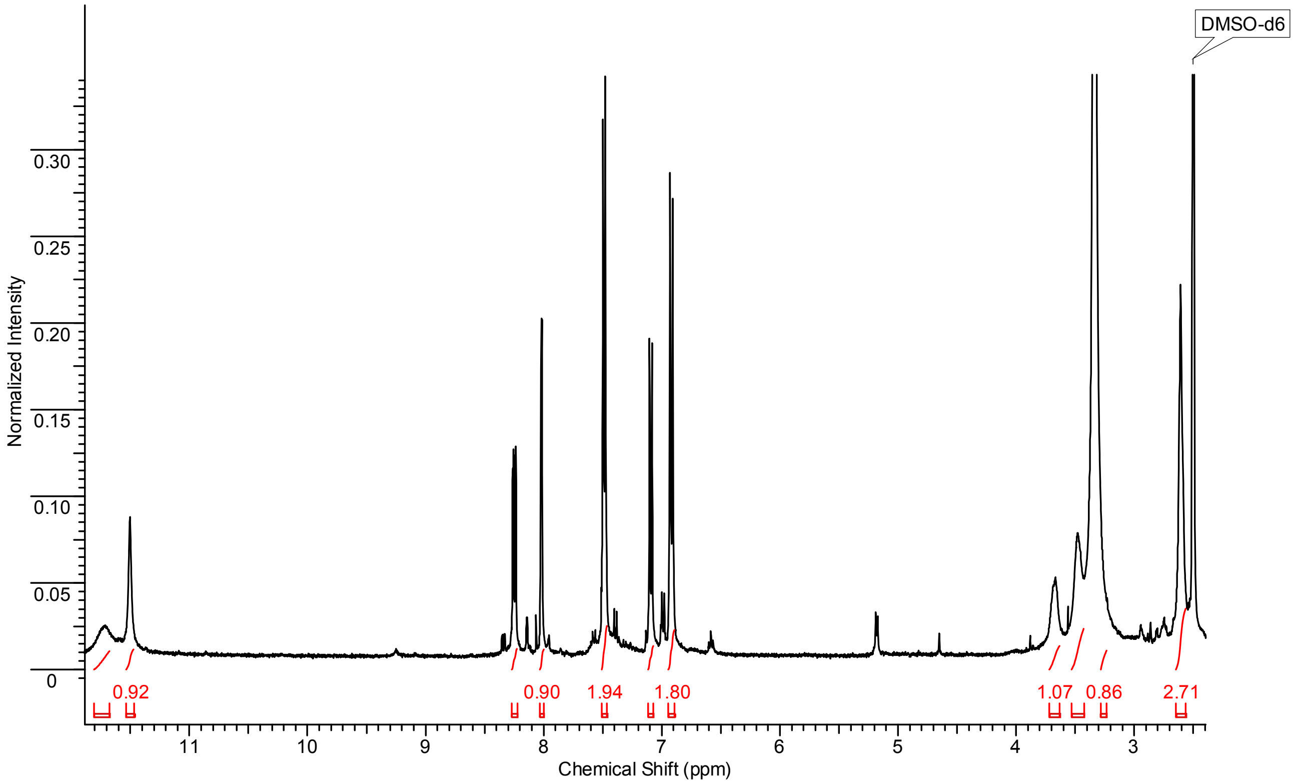

^{1}HNMR (400 MHz, DMSO-d6, δ, ppm):11.74 (bs, 1H, NH), 11.48 (s, 1H, NH), 8.25 (dd, J_1=2.4Hz, J_2=8.6 Hz, 1H, isatin), 8.02 (d, J=2.0 Hz, 1H, isatin), 7,49 (d, J=8.6Hz, 2H, Ar), 7.09 (d, J=9.0 Hz, 1H, isatin), 6.92 (d, J=8.2Hz, 2H, Ar), 3.72-3.63(m, 1H, pyrrolidine), 3.53-3.42(m, 2H, pyrrolidine), 3.28-3.23(m, 1H, pyrrolidine), 2.61 (bs, 3H, NCH_3).

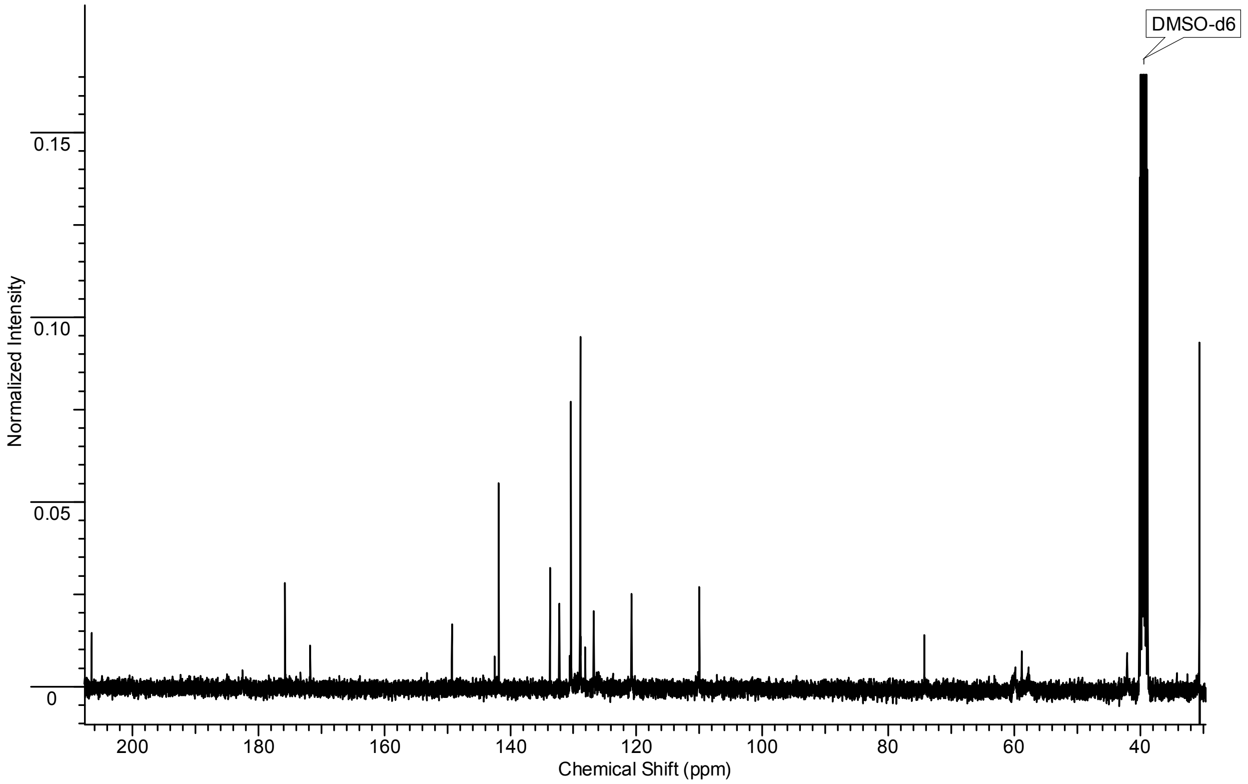

^{13}C NMR (101 MHz, DMSO-d6, δ, ppm): 206.5, 175.8, 171.8, 149.3, 141.8, 133.7, 132.2, 130.4, 128.9, 126.8, 120.8, 110.0, 74.3, 58.9, 42.1, 30.7.

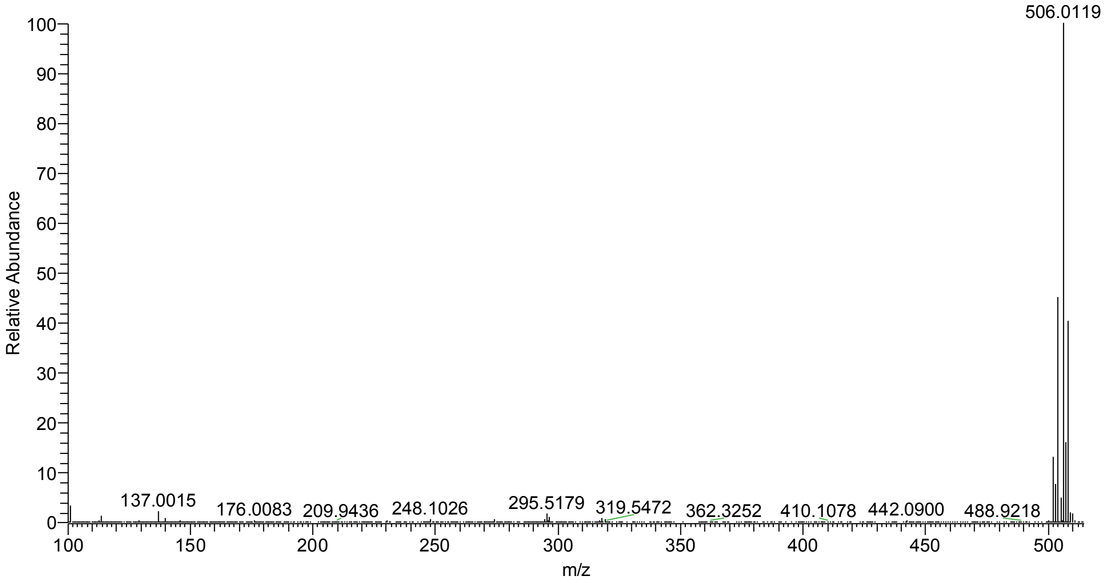

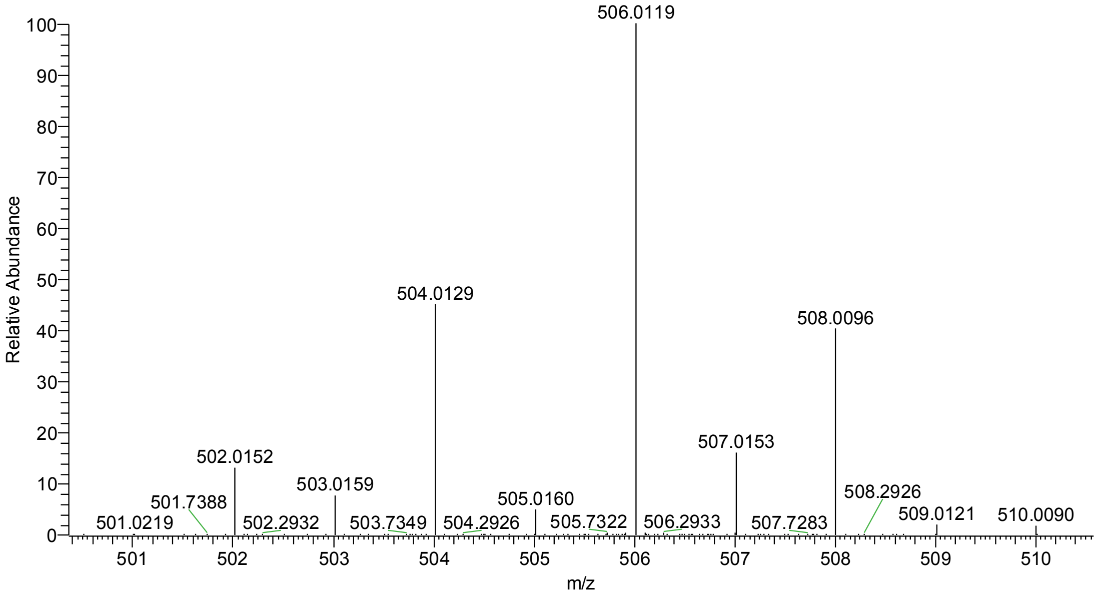

HRMS (ESI, m/Z): calculated ($C_{20}H_{16}ClN_5O_4Se$, M+H): 506.0129, found (M+H): 506.0119.

1'-methyl-3-(3-chlorophenyl)-2-selenoxodispiro[imidazolidine-5,3'-pyrrolidine-4',3''-indoline]-2'',4-dione (6i)

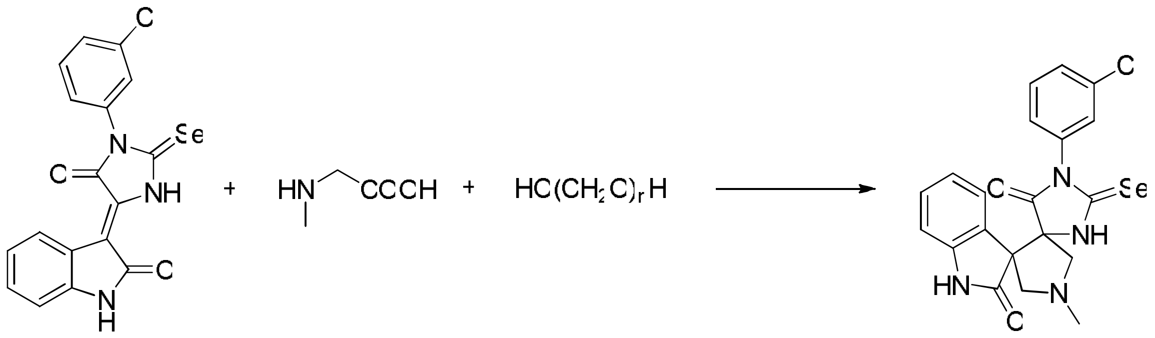

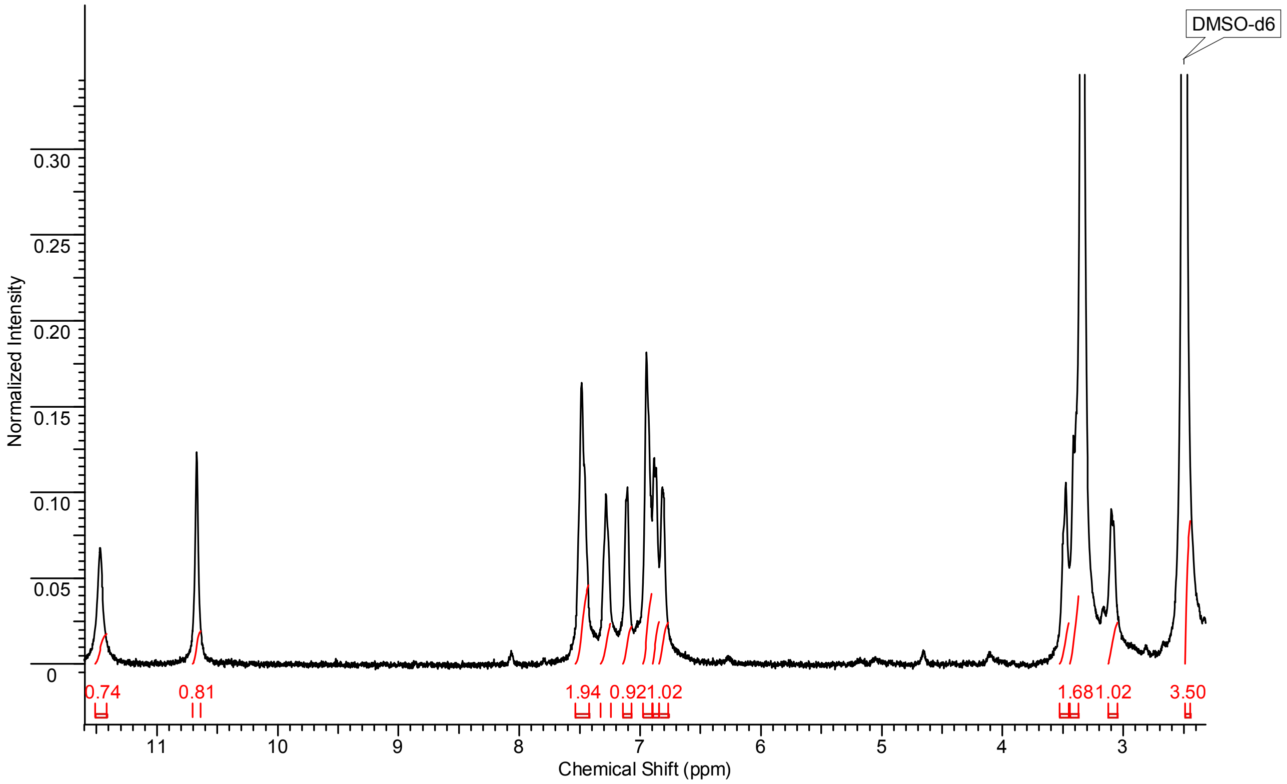

^{1}H NMR (400 MHz, DMSO-d6, δ, ppm): 11.47 (s, 1H, NH), 10.67 (s, 1H, NH), 7.54-7.41 (m, 2H, Ar), 7.28 (m, 1H, Ar), 7.11 (m, 1H, Ar), 6.99-6,76 (m, 4H, Ar), 3.49 (m, 1H, pyrrolidine), 3.44-3.37 (m, 2H, pyrrolidine), 3.09 (m, 1H, pyrrolidine), 2.49 (m, 3H, NCH_3).

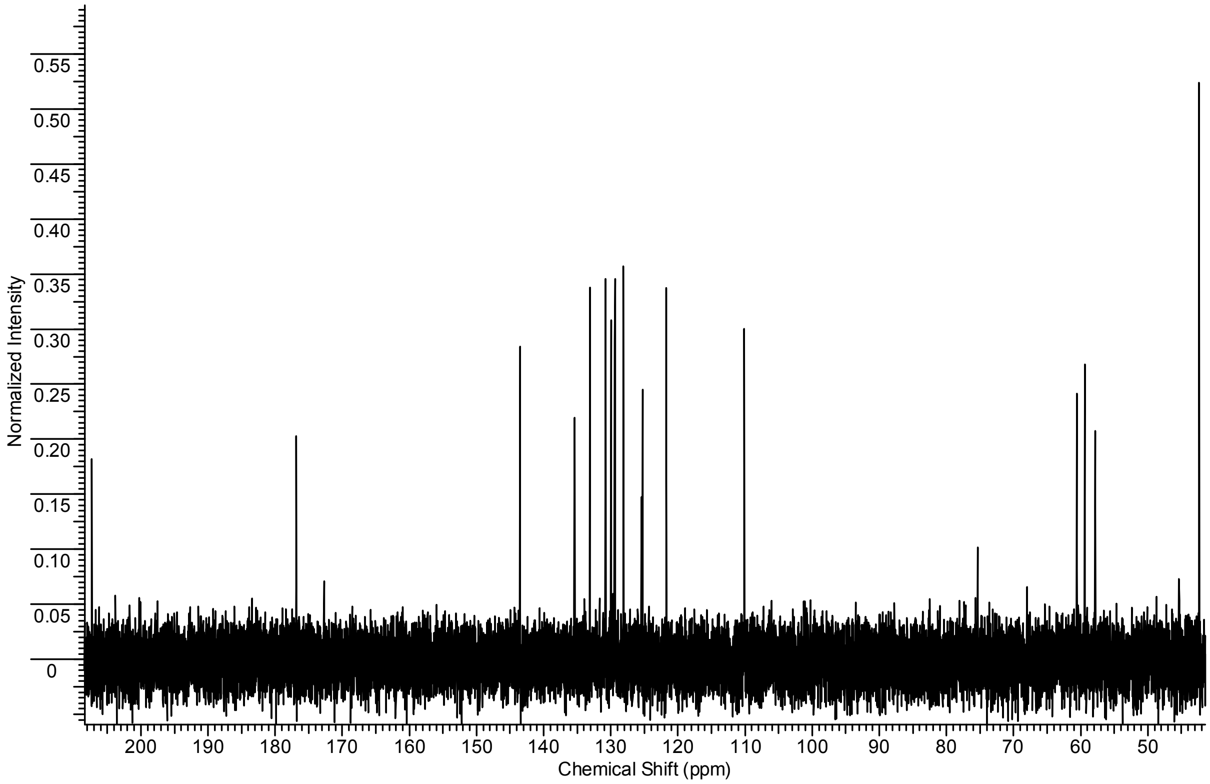

^{13}C NMR (101 MHz, DMSO-d6, δ, ppm): 206.9, 176.4, 143.1, 135.0, 132.7, 130.3, 129.5, 129.0, 128.9, 127.7, 125.0, 124.8, 121.3, 109.7, 74.9, 60.1, 58.9, 57.4, 41.9.

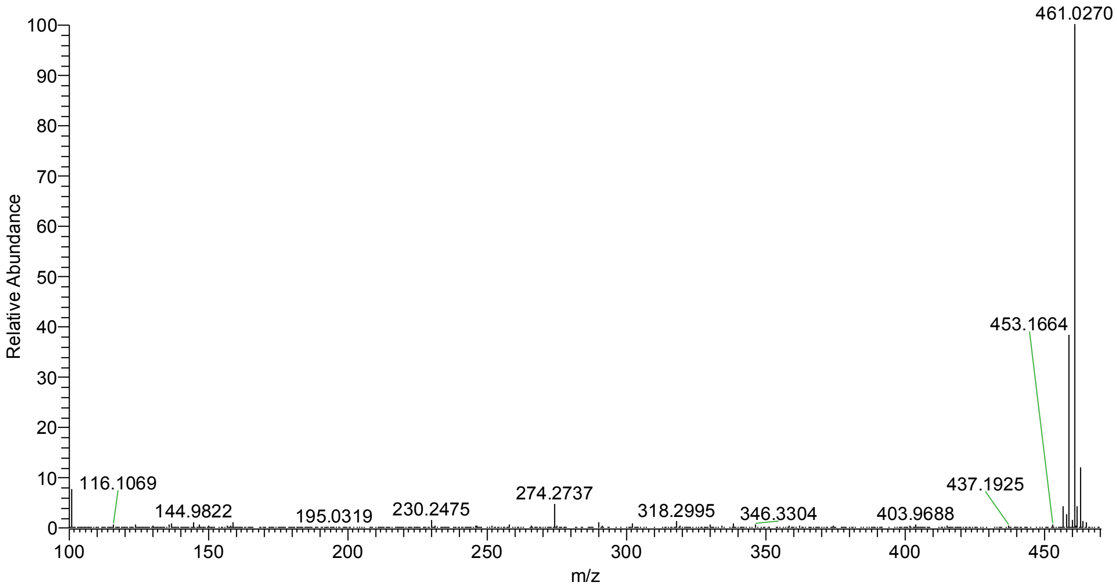

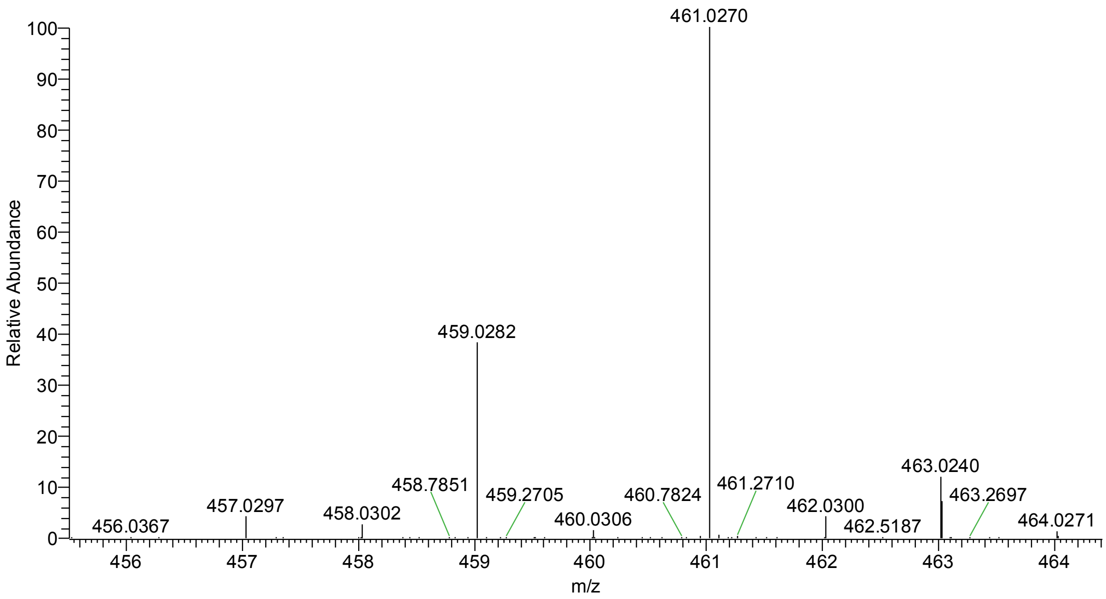

HRMS (ESI, m/Z): calculated ($C_{20}H_{17}ClN_4O_2Se$, M+H): 461.0278, found (M+H): 461.0270.

5’’-chloro-1'-methyl-3-(3-chloro-4-fluorophenyl)-2-selenoxodispiro[imidazolidine-5,3'-pyrrolidine-4',3''-indoline]-2'',4-dione (6j)

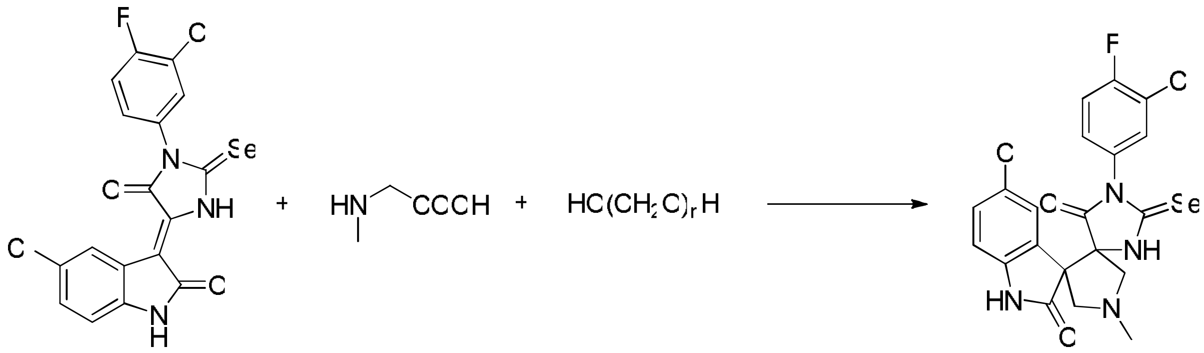

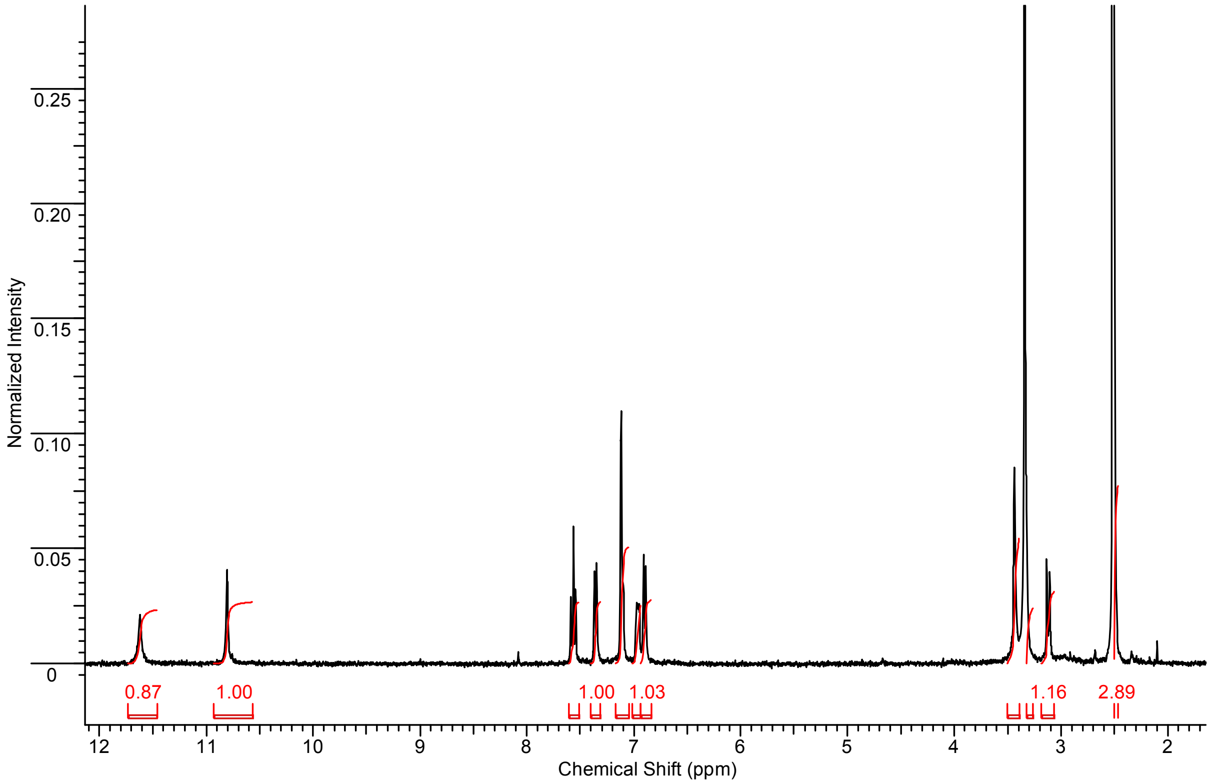

^{1}H NMR (400 MHz, DMSO-d6, δ, ppm): 11.62 (s, 1H, NH), 10.81 (s, 1H, NH), 7.57 (t, J=9.0 Hz, 1H, Ar), 7.36 (dd, J_1=1.8 Hz, J_2=8.3 Hz, 1H, Ar), 7.12 (m, 1H, Ar), 7.10 (m, 1H, Ar), 6.96 (m, 1H, Ar), 6.90 (d, J=8,3 Hz, 1H, Ar), 3.43 (m, 2H, pyrrolidine), 3.32 (m, 1H, pyrrolidine), 3.12 (d, J=10,2 Hz, 1H, pyrrolidine), 2.49 (s, 3H, NCH_3).

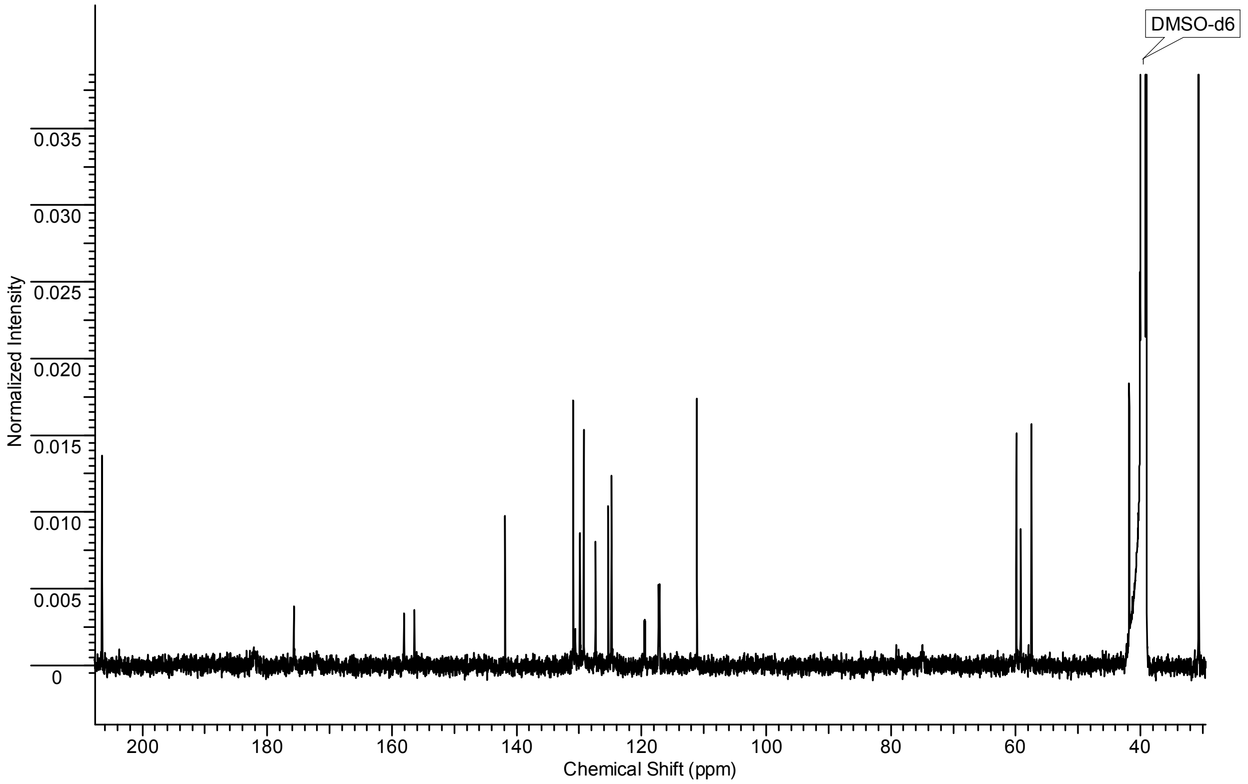

^{13}C NMR (101 MHz, DMSO-d6, δ, ppm): 206.5, 175.7, 158.1, 156.4, 141.9, 130.9, 129.9, 129.2, 127.3, 125.3, 124.8, 119.5, 117.1, 111.1, 59.9, 59.2, 57.5, 41.8, 30.7.

HRMS (ESI, m/Z): calculated ($C_{20}H_{15}Cl_2FN_4O_2Se$, M+H): 512.9794, found (M+H): 512.9798.

5’’-bromo-1'-methyl-3-cyclopropyl-2-selenoxodispiro[imidazolidine-5,3'-pyrrolidine-4',3''-indoline]-2'',4-dione (6k)

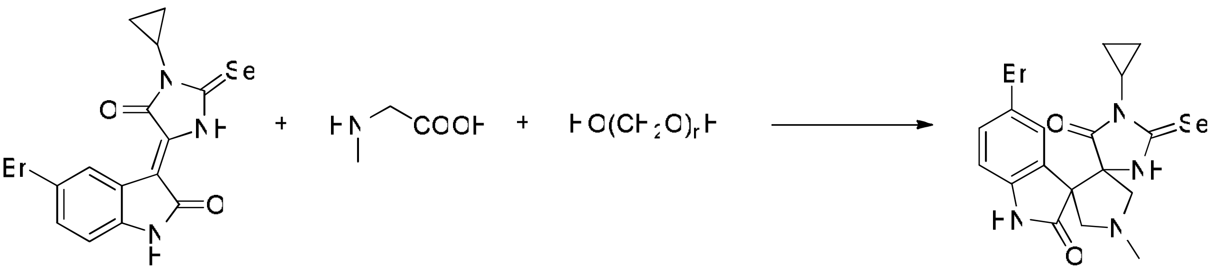

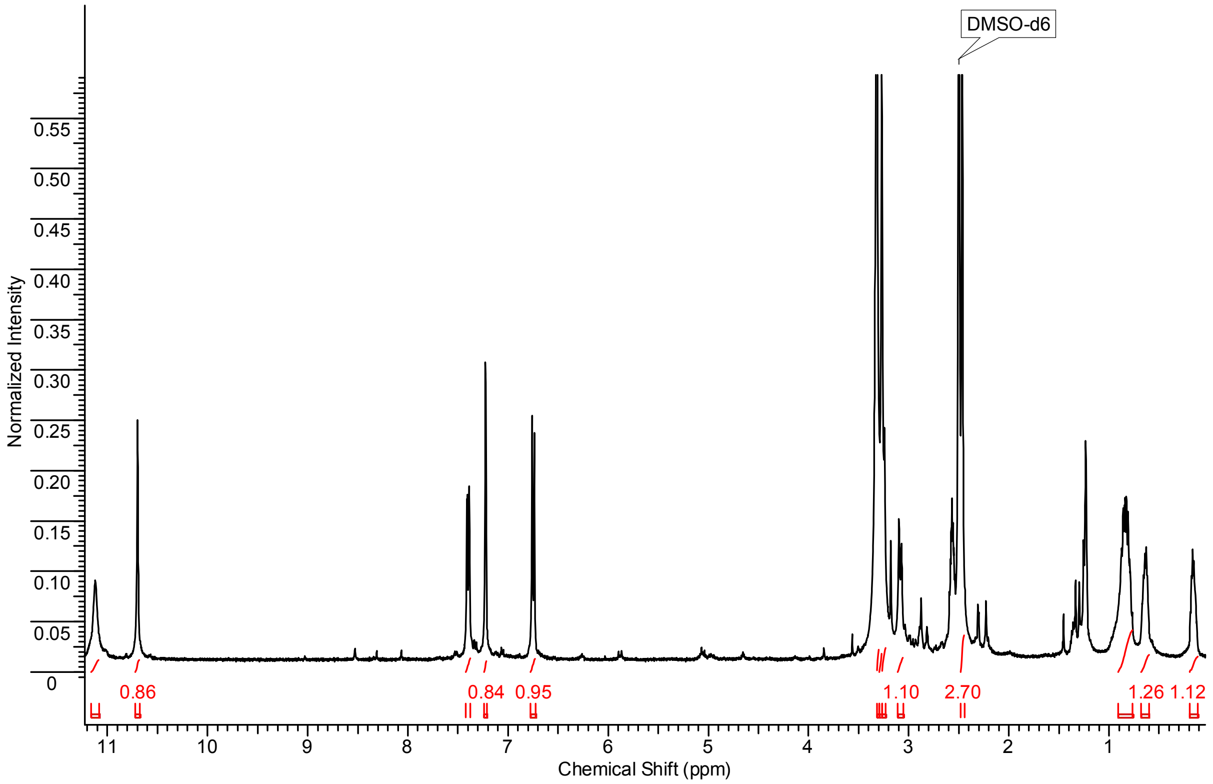

^{1}HNMR (400 MHz, DMSO-d6, δ, ppm): 11.12 (bs, 1H, NH), 10.70(s, 1H, NH), 7.40 (dd,J_1=1.2 Hz, J_2=8.2 Hz, 1H, isatin), 7.22 (d,J=1.6 Hz, 1H, isatin), 6.75 (d,J=8.2 Hz, 1H, isatin), 3.32-3.29 (m, 1H, pyrrolidine), 3.29-3.26 (m, 1H, pyrrolidine), 3.25 (d, J=10.2 Hz, 1H, pyrrolidine), 3.09 (d, J=9.8 Hz, 1H, pyrrolidine), 2.47(s, 3H, NCH_3), 0.91-0.76 (m, 3H, Pr), 0,68-0.60(m, 1H, Pr), 0.20-0.12(m, 1H, Pr).

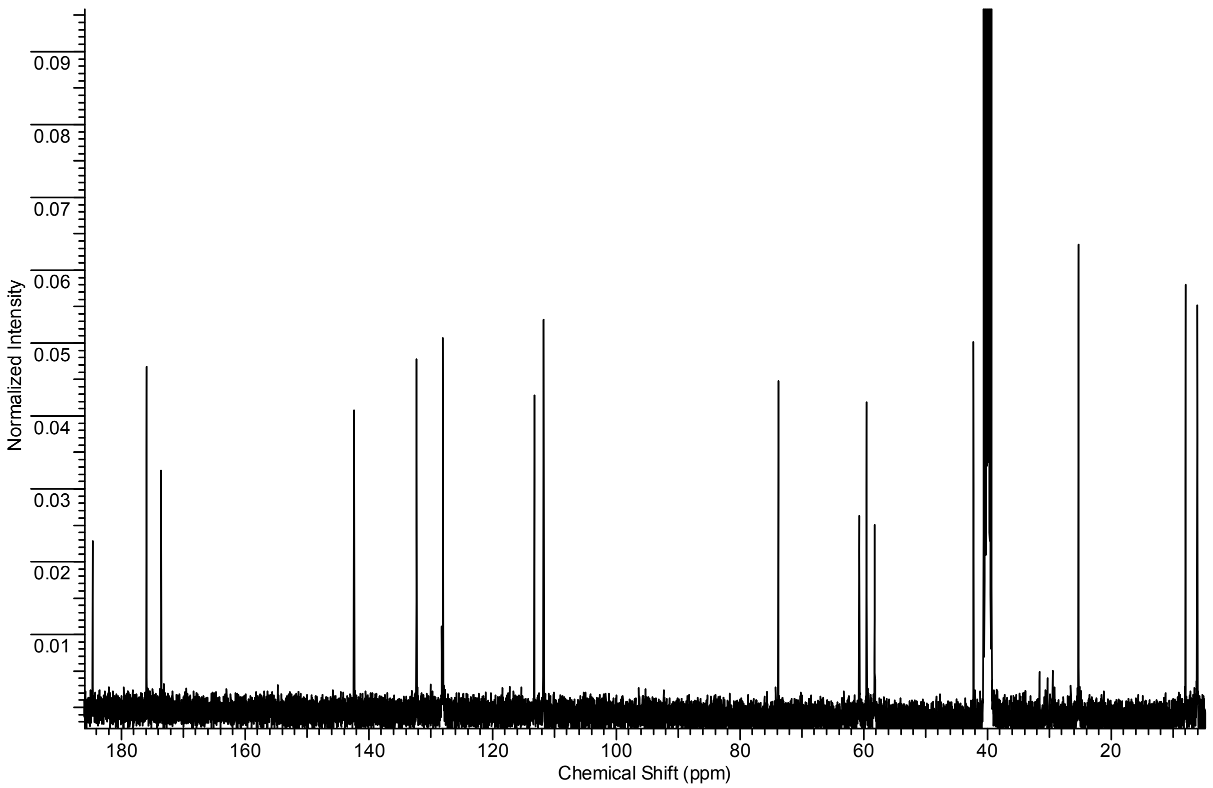

^{13}CNMR (101 MHz, DMSO-d6, δ, ppm): 184.2, 175.5, 173.1, 142.0, 131.8, 127.7, 127.5, 112.8, 111.3, 73.4, 60.3, 59.0, 57.8, 41.8, 24.8, 7.5, 5.7.

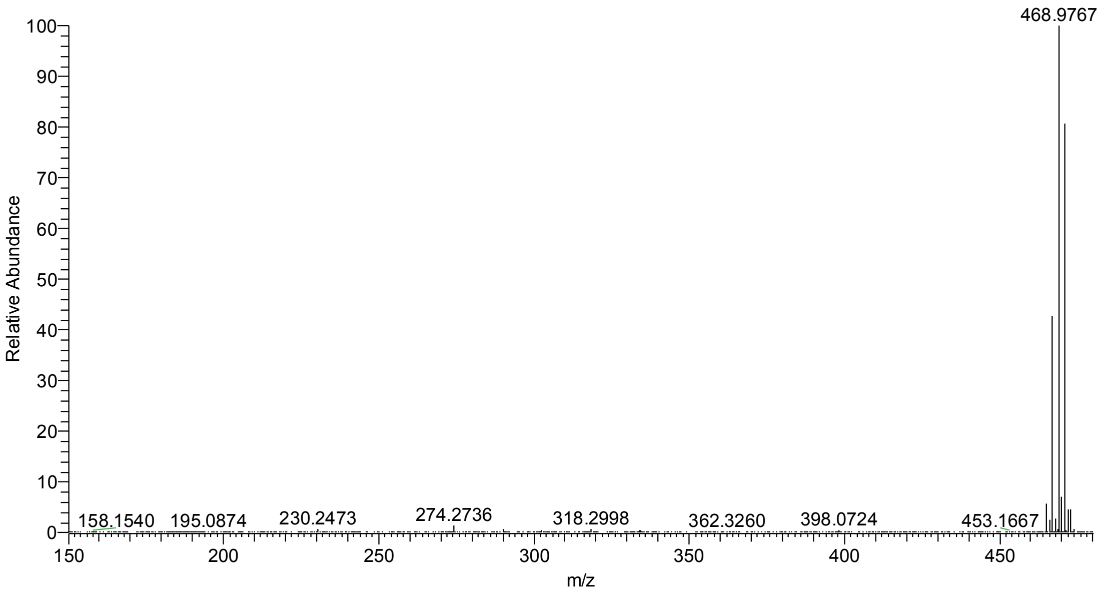

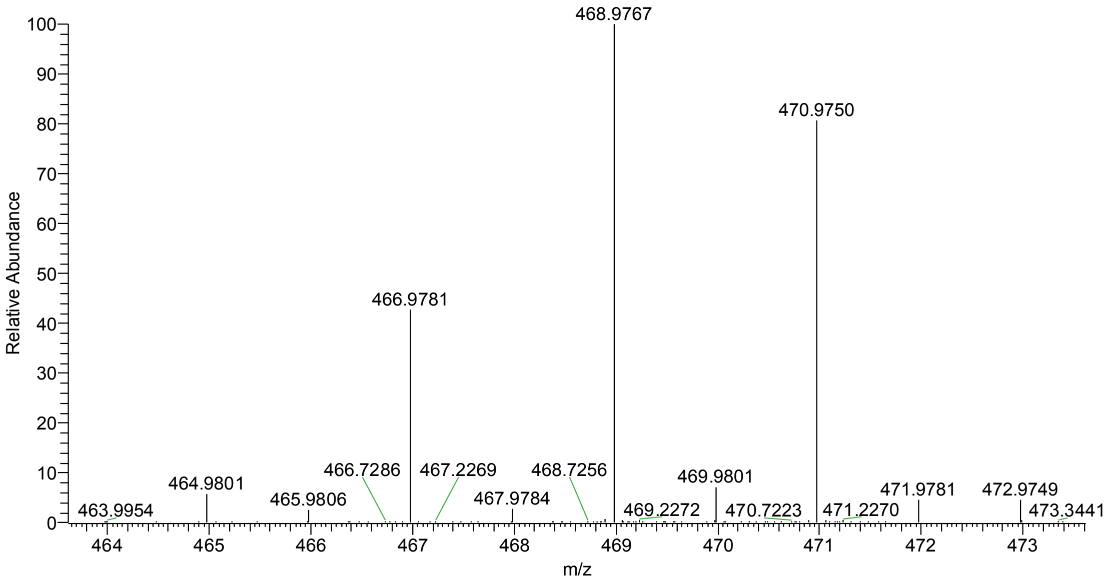

HRMS (ESI, m/Z): calculated ($C_{17}H_{17}BrN_4O_2Se$, M+H): 468.9773, found (M+H): 468.9767.

1'-methyl-3-(4-methoxybenzyl)-2-selenoxodispiro[imidazolidine-5,3'-pyrrolidine-4',3''-indoline]-2'',4-dione (6l)

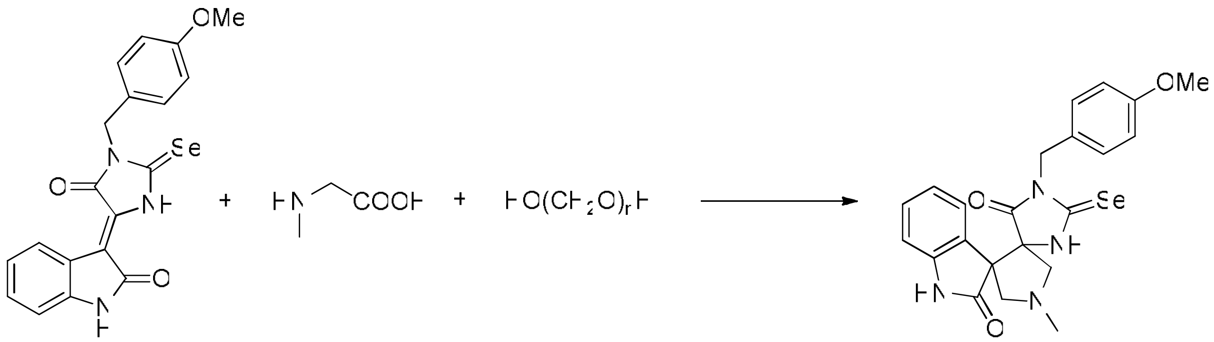

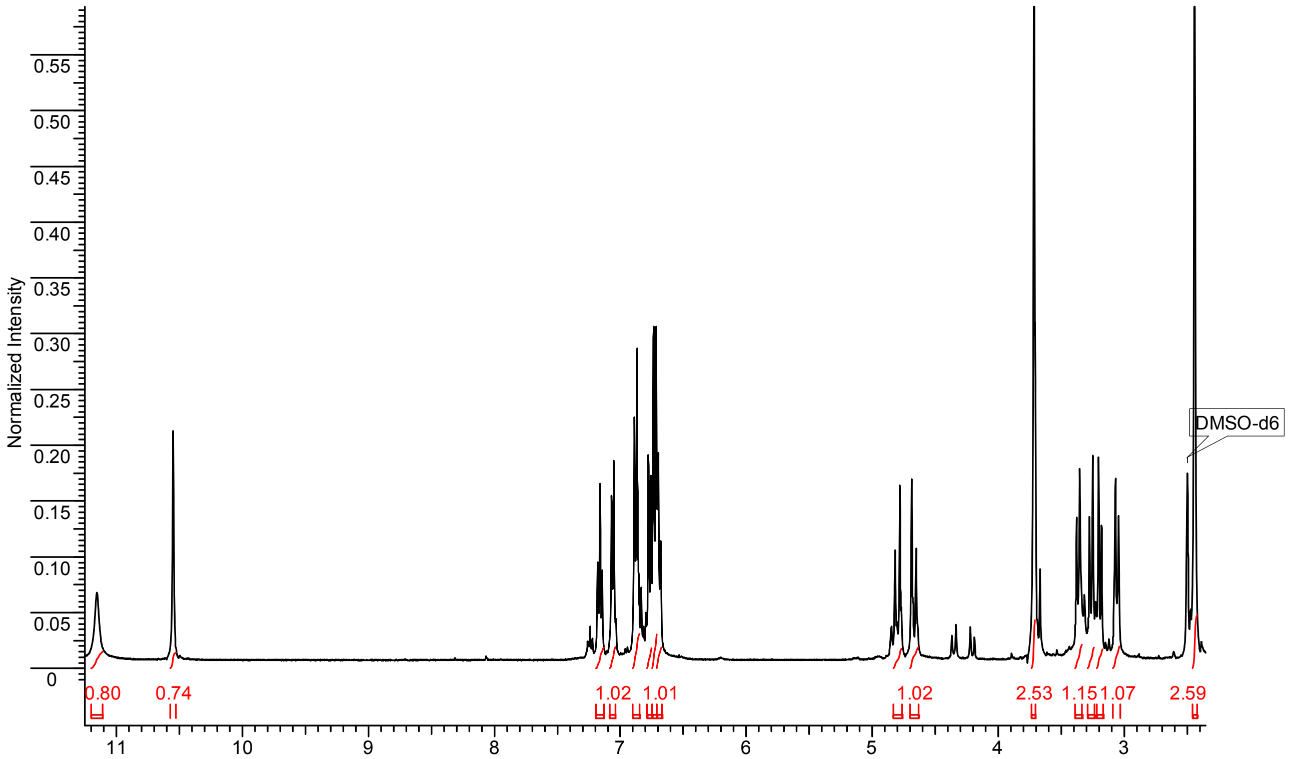

^{1}HNMR (400 MHz, DMSO-d6, δ, ppm):11.16 (bs, 1H, NH), 10.55 (s, 1H, NH), 7.16 (t, J=7.5 Hz, 1H, isatin), 7.06 (d, J=7.3 Hz, 1H, isatin), 6.88 (d, J=8.4 Hz, 2H, Ar), 6.77 (d, J=7.7 Hz, 1H, isatin), 6.73 (d, J=8.3 Hz, 2H, Ar), 6.70, (t, J=7.2 Hz, 1H, isatin), 4.80 (d, J=14.9 Hz, 1H, CH_2), 4.67 (d, J=14.9 Hz, 1H, CH_2), 3.72 (s, 3H, OCH_3), 3.36 (d, J=10.1 Hz, 1H, pyrrolidine), 3.26 (d, J=9.9 Hz, 1H, pyrrolidine), 3.19 (d, J=10.1 Hz, 1H, pyrrolidine), 3.06 (d, J=9.9 Hz, 1H, pyrrolidine), 2.44 (s, 3H, NCH_3).

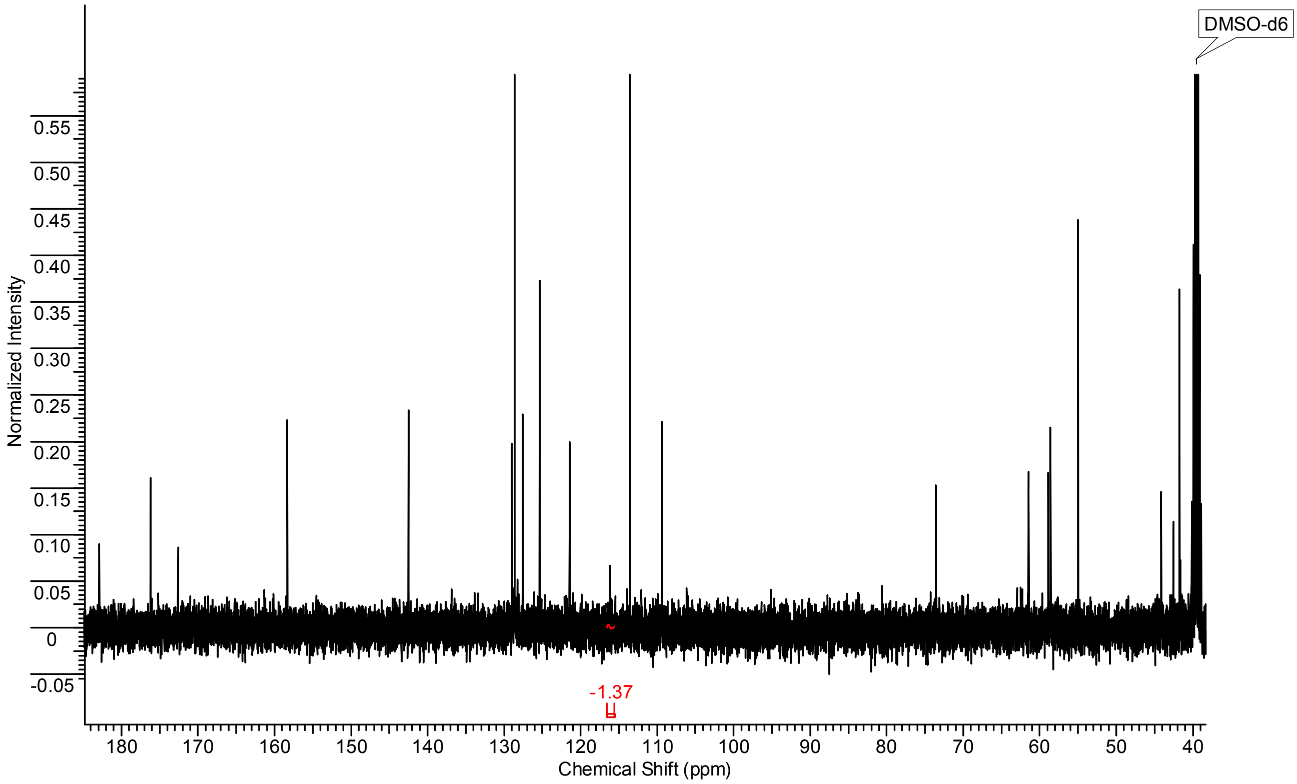

^{13}C NMR (101 MHz, DMSO-d6, δ, ppm): 182.9, 176.2, 172.6, 158.3, 142.5, 129.0, 128.7, 128.6, 127.6, 125.3, 121.4, 113.5, 109.4, 73.6, 61.4, 58.9, 58.6, 55.0, 44.1, 42.6, 41.8.

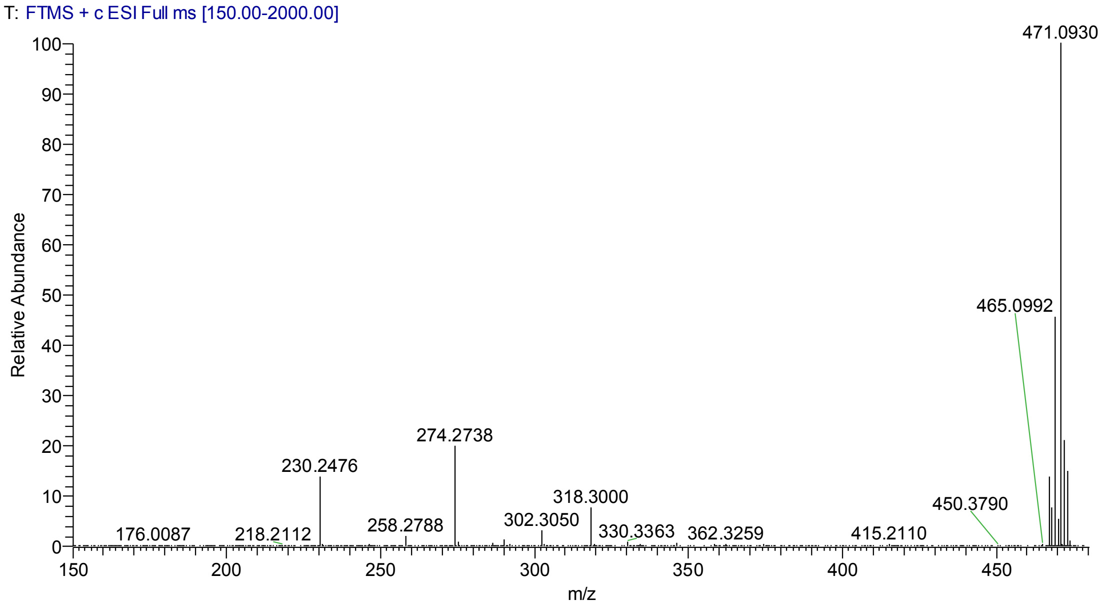

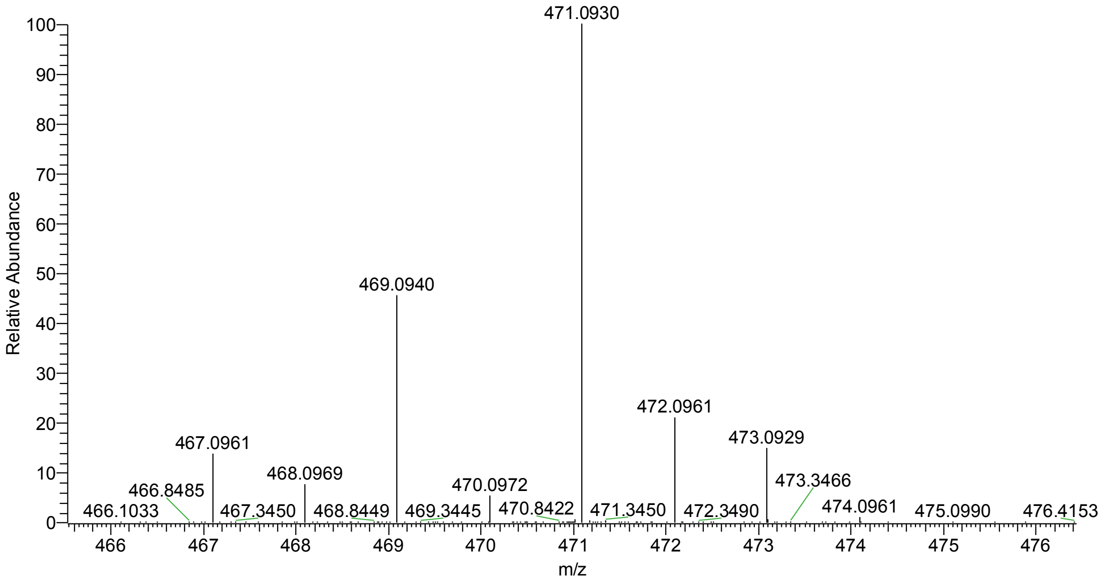

HRMS (ESI, m/Z): calculated ($C_{22}H_{22}N_4O_3Se$, M+H): 471.0930, found (M+H): 471.0930.

5’’-bromo-1'-methyl-3-(4-methoxybenzyl)-2-selenoxodispiro[imidazolidine-5,3'-pyrrolidine-4',3''-indoline]-2'',4-dione (**6m**)

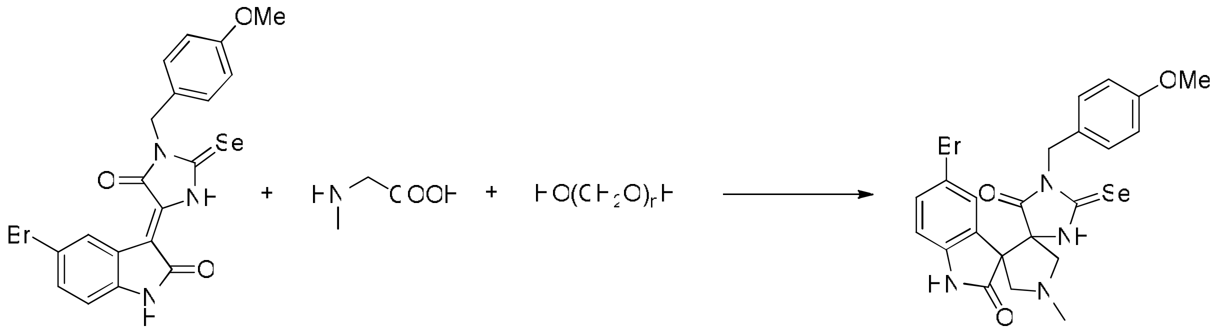

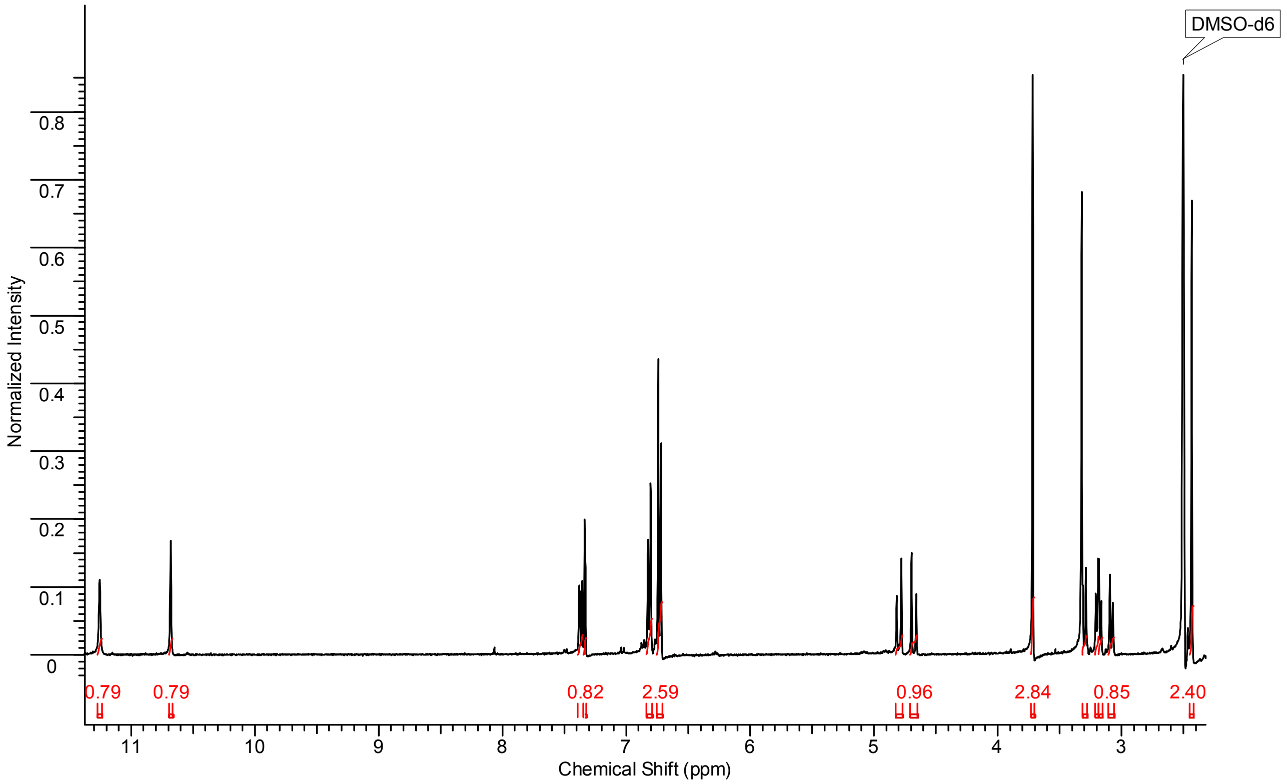

^{1}HNMR (400 MHz, DMSO-d6, δ, ppm):11.25 (s, 1H,NH), 10.68 (s, 1H,NH), 7.37 (dd, J_1=2.0 Hz, J_2=7.8 Hz, 1H, isatin), 7.34 (s, 1H, isatin), 6.82 (d, J=7.8 Hz, 2H, Ar), 6.75-6.73 (m, 1H, isatin), 6.73 (d, J=8.8 Hz, 2H, Ar), 4.80 (d, J=14.7 Hz, 1H, CH_2), 4.68 (d, J=14.7 Hz, 1H, CH_2), 3.72 (s, 3H, OCH_3), 3.30 (m, 1H, pyrrolidine), 3.20 (d, J=6.9 Hz, 1H, pyrrolidine), 3.17 (d, J=7.8 Hz, 1H, pyrrolidine), 3.08 (d, J=9.8 Hz, 1H, pyrrolidine), 2.43 (s, 3H, NCH_3).

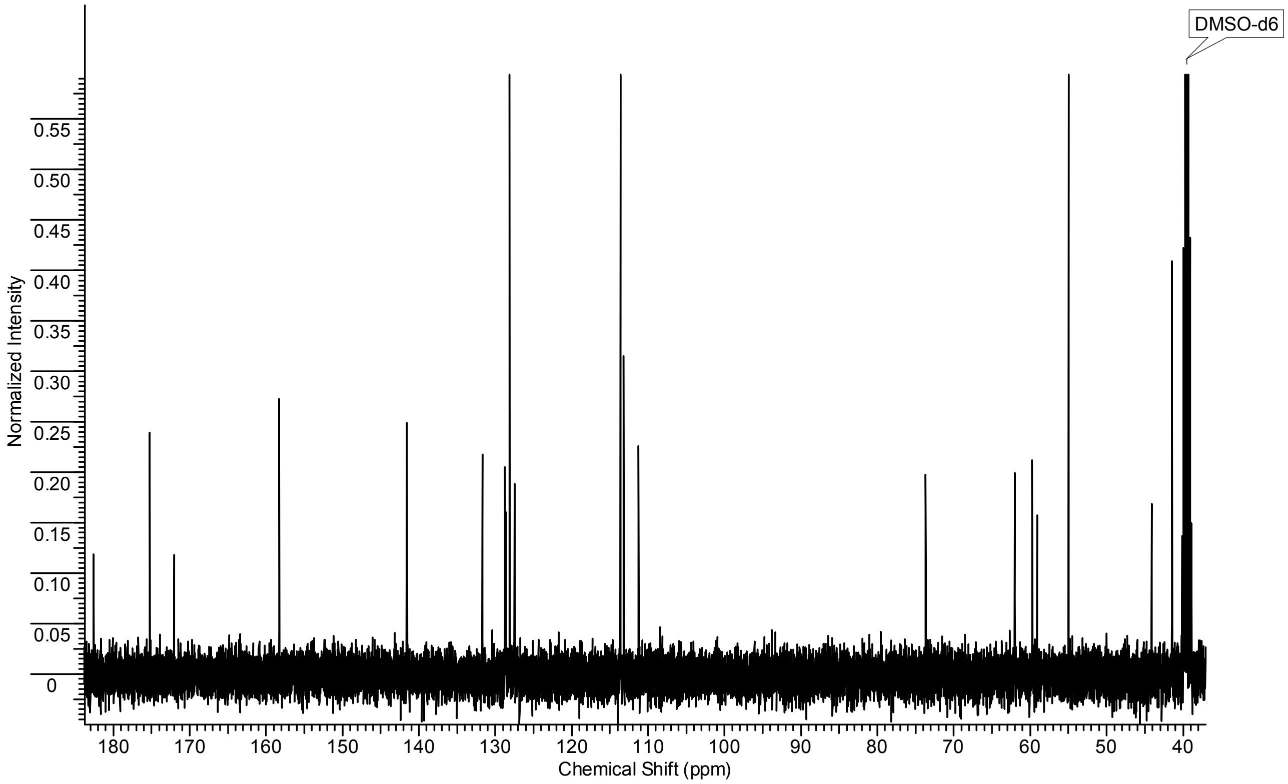

^{13}C NMR (101 MHz, DMSO-d6, δ, ppm):182.6, 175.2, 172.1, 158.3, 141.6, 131.7, 128.7, 128.6, 128.1, 127.5, 113.6, 113.2, 111.3, 73.7, 62.0, 59.7, 59.1, 55.0, 44.1, 41.5.

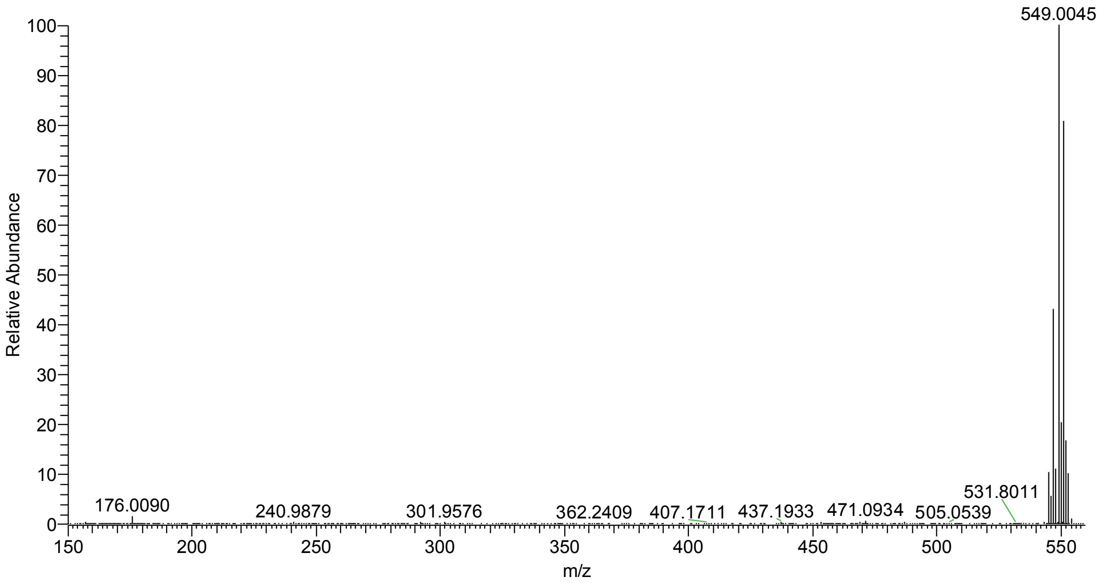

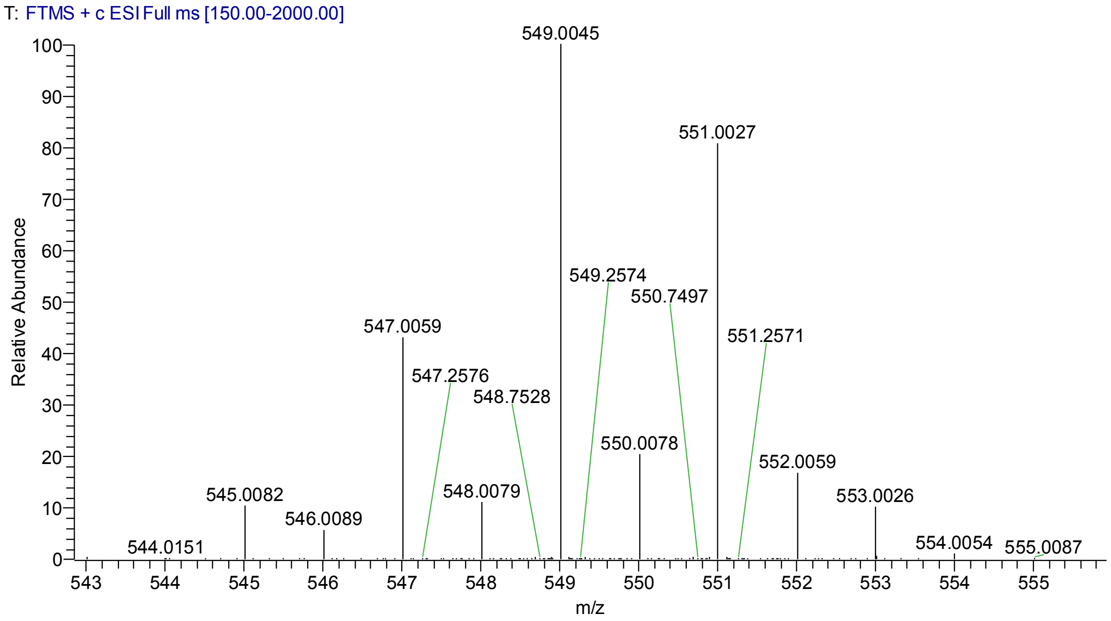

HRMS (ESI, m/Z): calculated ($C_{22}H_{21}BrN_4O_3Se$, M+H): 549.0035, found (M+H): 549.0045.

Additional experiments for the determination of cytotoxicity

A. Fluorescent cells based cytotoxicity assay (FCT assay).

The cytotoxicity of leader substances was retested using the FCT assay [https://doi.org/10.1007/s11307-017-1152-0]. Briefly, 3000 cells per well for A549_tdTomato cell line, or 5000 cells per well for VA13_Katushka cell line were plated out in 140 µl of F12 media (Paneco LLC, Russia) in 96-well plate and incubated in the 5% CO2 incubator for first 16 h without treating. Then 10 µl of media-DMSO solutions of tested substances to the cells (final DMSO concentrations in the media were 0.5% or less) and treated cells 72 h with 1.56 - 50 µM (six dilutions) of our substances (triplicate each). To receive images of the cells, plates were scanned with a TYPHOON FLA950 (GE Healthcare) with the 532 nm laser and the BPG1 emission filter (560-580 nm) to image A549_tdTomato, and the 635 nm laser and the LPR-Ch2 emission filter (≥665 nm) to image VA13_Katushka. The voltage of the laser was adjusted to 600V for tdTomato and 850V for Katushka2S. The maximum 10-micron resolution of the scanner was used. Processing of plate images was carried out using the ImageJ editor (https://imagej.nih.gov/ij/). The plug-in from [https://doi.org/10.1371/journal.pone.0092444] for automation of image processing was changed according to [DOI: 10.1007/s11307-017-1152-0]. The results were used to construct a dose-response graph and to estimate CC_{50} value (GraphPad Software, Inc., San Diego, CA).

B. CalceinAM assay.

CalceinAM assay was based on Live&Dead assay protocol (ThermoFisher). 3000 cells per well for A549 cell line, or 5000 cells per well for VA-13 cell line were plated out in 140 µl of F12 media (Paneco LLC, Russia) in 96-well plate and incubated in the 5% CO_2 incubator for first 16 h without treating. Then 10 µl of media-DMSO solutions of tested substances to the cells (final DMSO concentrations in the media were 0.5% or less) and treated cells 72 h with 1.56 - 50 µM (six dilutions) of our substances (triplicate each). Cells in 96-well plate after incubation with tested compounds were rinsed with PBS, then incubated with 3 µM CalceinAM (ThermoFisher) solution in PBS for 30 min. After incubation cells were rinsed with PBS and then fluorescence was measured in microplate fluorimeter (VICTOR X5 Light Plate Reader, PerkinElmer, USA) with excitation 485 nm and emission 535 nm. The results were used to construct a dose-response graph and to estimate CC_{50} value (GraphPad Software, Inc., San Diego, CA).

Table S1. CC50 of leader compounds on A549_tdTomato and VA13_Katushka cells evaluated by FCT assay.

**Compound**	**CC50 (µM) on A549_tdTomato cells**	**CC50 (µM) on VA13_Katushka cells**
**5a**	15.5±2.7	>50
**5b**	10.6±1.5	35.2±18.1
**5c**	13.3±2.7	>50
**5d**	8.7±3.7	44.4±13.6
**5e**	12.4±1.8	23.9±7.6
**6b**	10.6±1.3	17.1±2.6
**6c**	12±2	22+-11

Table S2. CC50 of compounds with high, medium and no selectivity indexes in MTT assay, retested with detection of viable A549 and VA13 cells by CalceinAM staining.

**Compound**	**CC50 (µM) on A549 cells**	**CC50 (µM) on VA13 cells**
5b	2.9±1	34.7±3.3
5e	15.4±3.4	34.5±2.8
6c	35.2±9.1	31.7±6.8

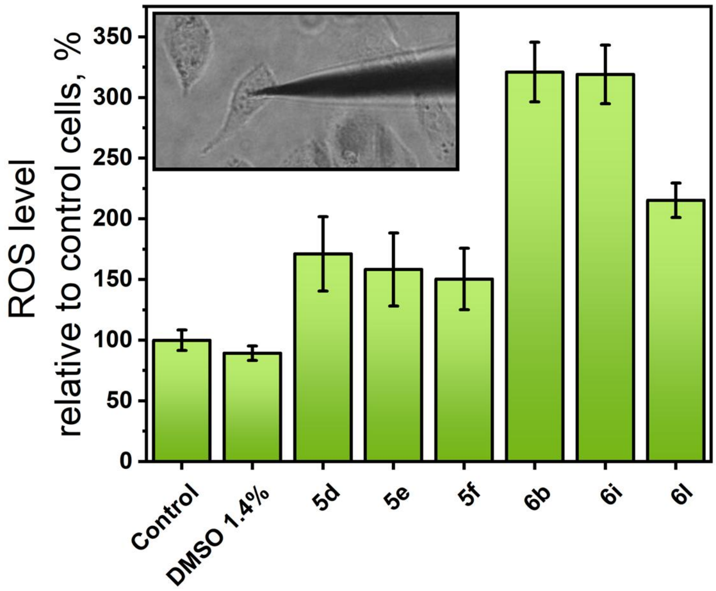

Figure S1. Comparison of the reactive oxygen species (ROS) level inside the PC3 tumor cells incubated with compounds **5** and **6**; *, $p < 0.05$ (one-way ANOVA).

Pt nanoelectrodes fabrication

Disk-shaped carbon nanoelectrode exhibited normal steady state behavior at oxidation potentials in ferrocene-methanol solution before etching due to limited diffusion to the surface (Figure S2A). In the process of etching, an increase in current is observed (at each subsequent cycle) which associated with a gradual increase in the effective surface of the electrode (Figure S2B). The use of high oxidation potentials causes the oxidation of carbon, water and chloride, leading to the formation of carbon oxides, molecular chlorine (Cl_2), molecular oxygen (O_2), etc. As a result, the carbon layer is oxidized.

It is worth noting that there is no plateau on the cyclic voltammograms (figure S2C), as was observed in Figure S2A. After etching, almost symmetric peaks of Fc oxidation and reduction appear due to the presence of cavities (Figure S2C).The amount of the reduced Fc form inside the cavity decreases (during its oxidation), which leads to a decrease in the current after the complete consumption of its reduced form and, as a result, to the appearance of peaks in the cyclic voltammogram.

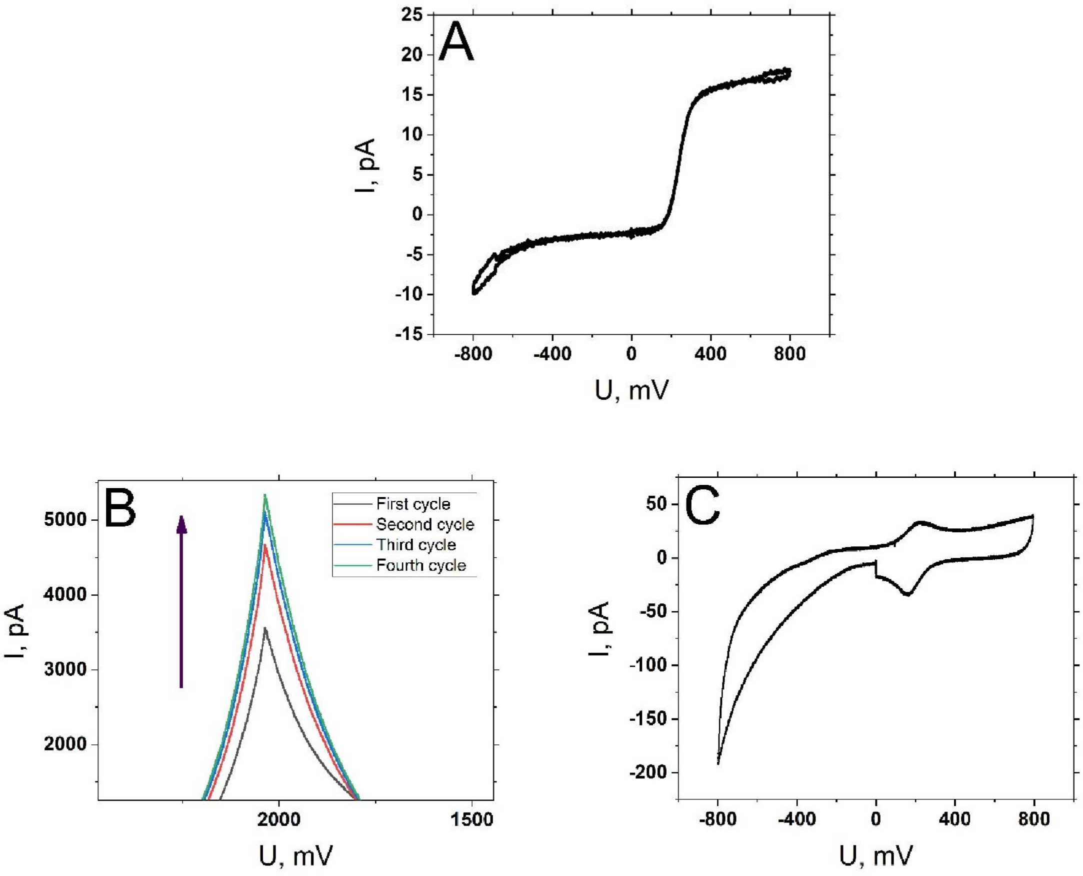

Figure S2. Cyclic current-voltage characteristics of the sensor at different stages of manufacturing. A – a carbon nanoelectrode before etching (1mM FcMeOH, 400 mV/s), B – during the process of etching the carbon nanoelectrode (0.1 M NaOH, 220 mV/s), C - after etching (1mM FcMeOH, 400 mV/s)

The platinization process was accompanied by an increase in the current at each deposition cycle (figure S2A), which ultimately leads to the filling of nanocavities and an increase in conductivity and catalytic activity of the electrode (Figure S3B).

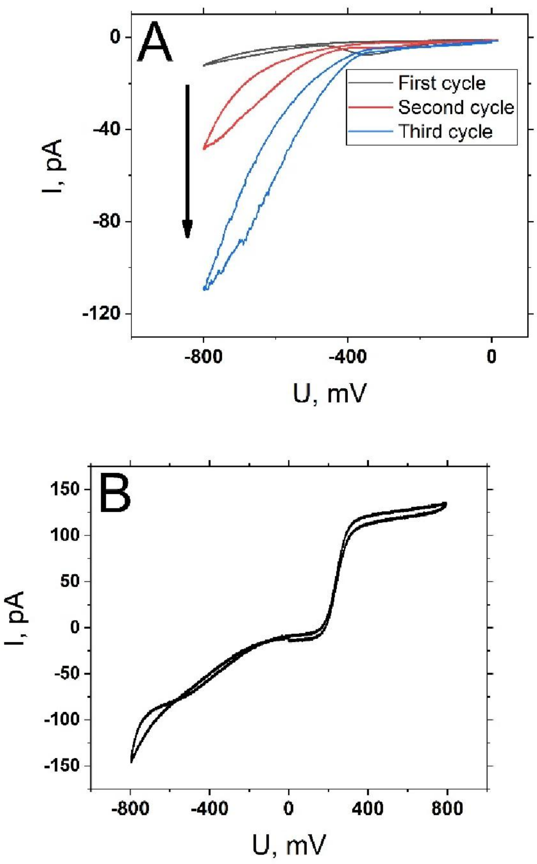

Figure S3. Cyclic current-voltage characteristics of platinization process A – process of platinization (2 mM H_2PtCl_6, 200 mV/s), B – Pt nanoelectrode (1mM FcMeOH, 400 mV/s)

Prior to the measurements, each platinum electrode was calibrated using a series of standard H_2O_2 solutions. Intercellular ROS level were determined based on the calibration curve (Figure S4). Under the given conditions, the platinum catalyzed reaction $2H_2O_2 = 2H_2O + O_2$

occurs, and since the superoxide radical is rapidly converted in solution into hydrogen peroxide, the total concentration of peroxide determines the general background of the oxidation processes.

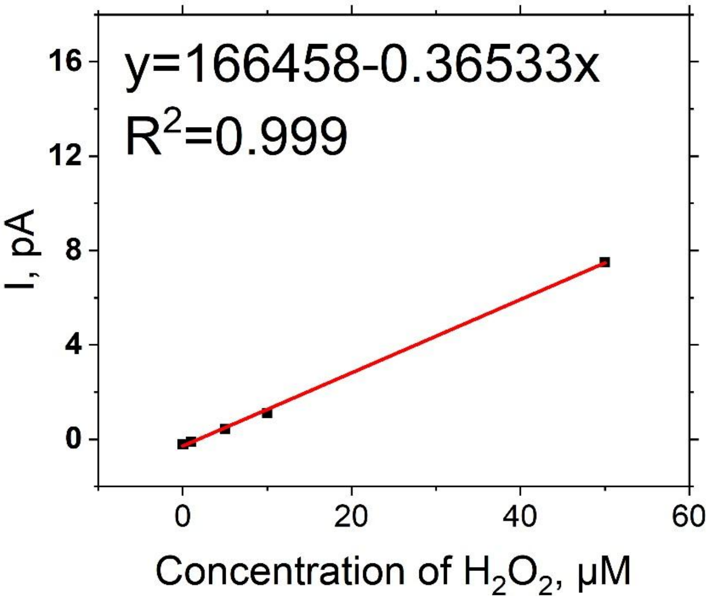

Figure S4. Calibration curve (current vs H_2O_2 concentration at +800 mV (vs. Ag/AgCl)) showing good linear response.

X-Ray Data for compound 5b

Table S3. Crystal data and structure refinement for **5b**.

Empirical formula	C28 H24 Cl1.50 N4 O3 Se	
Formula weight	596.65	
Temperature	293(2) K	
Wavelength	1.54186 E	
Crystal system	Triclinic	
Space group	P -1	
Unit cell dimensions	a = 7.9730(10) E	α= 98.800(10)°.
	b = 16.742(7) E	β= 95.700(10)°.
	c = 23.680(10) E	γ = 90.300(10)°.
Volume	3107.6(19) E^3	
Z	4	
Density (calculated)	1.275 Mg/m^3	
Absorption coefficient	3.089 mm^{-1}	
F(000)	1214	
Theta range for data collection	3.797 to 67.996°.	
Index ranges	-8<=h<=9, -19<=k<=13, -28<=l<=28	
Reflections collected	18520	
Independent reflections	7151 [R(int) = 0.1620]	
Completeness to theta = 67.686°	63.5 %	
Refinement method	Full-matrix least-squares on F^2	
Data / restraints / parameters	7151 / 682 / 618	
Goodness-of-fit on F^2	1.115	
Final R indices [I>2sigma(I)]	R1 = 0.1513, wR2 = 0.3389	
R indices (all data)	R1 = 0.3218, wR2 = 0.3844	
Extinction coefficient	0.0044(4)	
Largest diff. peak and hole	1.095 and -0.524 e.E^{-3}	

Table S4. Bond lengths [E] and angles [°] for **5b**.

Se(1A)-C(1A)	1.91(3)
Cl(1A)-C(10A)	1.811(14)
Cl(2A)-C(18A)	1.65(2)
O(1A)-C(2A)	1.33(3)
O(2A)-C(7A)	1.20(3)
O(3A)-C(27A)	1.29(3)
O(3A)-C(24A)	1.310(17)
N(2A)-C(2A)	1.26(3)
N(2A)-C(1A)	1.31(3)
N(2A)-C(21A)	1.38(2)
N(5A)-C(1A)	1.42(3)
N(5A)-C(3A)	1.44(3)
N(5A)-H(5A)	0.8600
N(3A)-C(5A)	1.37(3)
N(3A)-C(4A)	1.48(3)
N(3A)-C(14A)	1.51(3)
N(4A)-C(7A)	1.35(4)
N(4A)-C(13A)	1.36(3)
N(4A)-H(4A)	0.8600
C(1A)-C(2A)	1.97(5)
C(2A)-C(3A)	1.35(3)
C(3A)-C(6A)	1.44(3)
C(3A)-C(4A)	1.53(3)
C(4A)-C(8A)	1.55(3)
C(4A)-C(7A)	1.61(4)
C(5A)-C(6A)	1.62(3)
C(5A)-H(5A1)	0.9700
C(5A)-H(5A2)	0.9700
C(6A)-C(15A)	1.61(3)
C(6A)-H(6A)	0.9800
C(8A)-C(9A)	1.3900
C(8A)-C(13A)	1.3900
C(9A)-C(10A)	1.3900
C(9A)-H(9A)	0.9300
C(10A)-C(11A)	1.3900
C(11A)-C(12A)	1.3900
C(11A)-H(11A)	0.9300
C(12A)-C(13A)	1.3900
C(12A)-H(12A)	0.9300
C(14A)-H(14A)	0.9633
C(14A)-H(14B)	0.9634
C(14A)-H(14C)	0.9634
C(15A)-C(16A)	1.3900
C(15A)-C(20A)	1.3900
C(16A)-C(17A)	1.3900
C(16A)-H(16A)	0.9300
C(17A)-C(18A)	1.3900
C(17A)-H(17A)	0.9300
C(18A)-C(19A)	1.3900
C(19A)-C(20A)	1.3900
C(19A)-H(19A)	0.9300
C(20A)-H(20A)	0.9300
C(21A)-C(22A)	1.3900
C(21A)-C(26A)	1.3900
C(22A)-C(23A)	1.3900
C(22A)-H(22A)	0.9300
C(23A)-C(24A)	1.3900

C(23A)-H(23A)	0.9300
C(24A)-C(25A)	1.3900
C(25A)-C(26A)	1.3900
C(25A)-H(25A)	0.9300
C(26A)-H(26A)	0.9300
C(27A)-C(28A)	1.54(4)
C(27A)-H(27A)	0.9700
C(27A)-H(27B)	0.9700
C(28A)-H(28A)	1.0080
C(28A)-H(28B)	1.0080
C(28A)-H(28C)	1.0059
Se(1B)-C(1B)	1.62(3)
Cl(1B)-C(10B)	1.813(13)
Cl(2B)-C(18B)	1.631(19)
O(1B)-C(2B)	1.25(3)
O(2B)-C(7B)	1.16(2)
O(3B)-C(24B)	1.31(2)
O(3B)-C(27B)	1.45(3)
N(2B)-C(1B)	1.30(3)
N(2B)-C(2B)	1.35(3)
N(2B)-C(21B)	1.45(3)
N(5B)-C(1B)	1.46(4)
N(5B)-C(3B)	1.64(3)
N(5B)-H(5B)	0.8600
N(3B)-C(5B)	1.49(3)
N(3B)-C(14B)	1.53(3)
N(3B)-C(4B)	1.57(3)
N(4B)-C(7B)	1.3799(12)
N(4B)-C(13B)	1.3801(11)
N(4B)-H(4B)	0.8600
C(2B)-C(3B)	1.40(3)
C(3B)-C(6B)	1.48(3)
C(3B)-C(4B)	1.56(3)
C(4B)-C(8B)	1.40(3)
C(4B)-C(7B)	1.77(3)
C(5B)-C(6B)	1.68(4)
C(5B)-H(5B1)	0.9700
C(5B)-H(5B2)	0.9700
C(6B)-C(15B)	1.56(3)
C(6B)-H(6B)	0.9800
C(8B)-C(9B)	1.3900
C(8B)-C(13B)	1.3900
C(9B)-C(10B)	1.3900
C(9B)-H(9B)	0.9300
C(10B)-C(11B)	1.3900
C(11B)-C(12B)	1.3900
C(11B)-H(11B)	0.9300
C(12B)-C(13B)	1.3900
C(12B)-H(12B)	0.9300
C(14B)-H(14D)	1.0936
C(14B)-H(14E)	1.0989
C(14B)-H(14F)	1.0998
C(15B)-C(16B)	1.3900
C(15B)-C(20B)	1.3900
C(16B)-C(17B)	1.3900
C(16B)-H(16B)	0.9300
C(17B)-C(18B)	1.3900
C(17B)-H(17B)	0.9300
C(18B)-C(19B)	1.3900

C(19B)-C(20B)	1.3900
C(19B)-H(19B)	0.9300
C(20B)-H(20B)	0.9300
C(21B)-C(22B)	1.3900
C(21B)-C(26B)	1.3900
C(22B)-C(23B)	1.3900
C(22B)-H(22B)	0.9300
C(23B)-C(24B)	1.3900
C(23B)-H(23B)	0.9300
C(24B)-C(25B)	1.3900
C(25B)-C(26B)	1.3900
C(25B)-H(25B)	0.9300
C(26B)-H(26B)	0.9300
C(27B)-C(28B)	1.61(4)
C(27B)-H(27C)	0.9700
C(27B)-H(27D)	0.9700
C(28B)-H(28D)	0.9602
C(28B)-H(28E)	0.9602
C(28B)-H(28F)	0.9602

C(27A)-O(3A)-C(24A)	120(2)
C(2A)-N(2A)-C(1A)	100(3)
C(2A)-N(2A)-C(21A)	138(3)
C(1A)-N(2A)-C(21A)	120(3)
C(1A)-N(5A)-C(3A)	105(2)
C(1A)-N(5A)-H(5A)	127.5
C(3A)-N(5A)-H(5A)	127.5
C(5A)-N(3A)-C(4A)	108(2)
C(5A)-N(3A)-C(14A)	111(2)
C(4A)-N(3A)-C(14A)	123(2)
C(7A)-N(4A)-C(13A)	120(3)
C(7A)-N(4A)-H(4A)	120.1
C(13A)-N(4A)-H(4A)	120.1
N(2A)-C(1A)-N(5A)	112(3)
N(2A)-C(1A)-Se(1A)	130(2)
N(5A)-C(1A)-Se(1A)	117(2)
N(2A)-C(1A)-C(2A)	38.9(16)
N(5A)-C(1A)-C(2A)	73.8(18)
Se(1A)-C(1A)-C(2A)	169(2)
N(2A)-C(2A)-O(1A)	111(3)
N(2A)-C(2A)-C(3A)	124(4)
O(1A)-C(2A)-C(3A)	125(3)
N(2A)-C(2A)-C(1A)	40.7(18)
O(1A)-C(2A)-C(1A)	152(3)
C(3A)-C(2A)-C(1A)	84(2)
C(2A)-C(3A)-N(5A)	97(3)
C(2A)-C(3A)-C(6A)	111(3)
N(5A)-C(3A)-C(6A)	117(2)
C(2A)-C(3A)-C(4A)	114(3)
N(5A)-C(3A)-C(4A)	111(2)
C(6A)-C(3A)-C(4A)	106(2)
N(3A)-C(4A)-C(3A)	105(2)
N(3A)-C(4A)-C(8A)	110(2)
C(3A)-C(4A)-C(8A)	120(2)
N(3A)-C(4A)-C(7A)	110(2)
C(3A)-C(4A)-C(7A)	109(2)
C(8A)-C(4A)-C(7A)	103(2)
N(3A)-C(5A)-C(6A)	104(2)
N(3A)-C(5A)-H(5A1)	111.0

C(6A)-C(5A)-H(5A1)	111.1
N(3A)-C(5A)-H(5A2)	111.0
C(6A)-C(5A)-H(5A2)	110.9
H(5A1)-C(5A)-H(5A2)	109.0
C(3A)-C(6A)-C(15A)	117(2)
C(3A)-C(6A)-C(5A)	106(2)
C(15A)-C(6A)-C(5A)	119(2)
C(3A)-C(6A)-H(6A)	104.5
C(15A)-C(6A)-H(6A)	104.5
C(5A)-C(6A)-H(6A)	104.5
O(2A)-C(7A)-N(4A)	131(4)
O(2A)-C(7A)-C(4A)	128(3)
N(4A)-C(7A)-C(4A)	101(3)
C(9A)-C(8A)-C(13A)	120.0
C(9A)-C(8A)-C(4A)	132.4(16)
C(13A)-C(8A)-C(4A)	107.5(16)
C(8A)-C(9A)-C(10A)	120.0
C(8A)-C(9A)-H(9A)	120.0
C(10A)-C(9A)-H(9A)	120.0
C(11A)-C(10A)-C(9A)	120.0
C(11A)-C(10A)-Cl(1A)	119.8(12)
C(9A)-C(10A)-Cl(1A)	120.1(12)
C(10A)-C(11A)-C(12A)	120.0
C(10A)-C(11A)-H(11A)	120.0
C(12A)-C(11A)-H(11A)	120.0
C(13A)-C(12A)-C(11A)	120.0
C(13A)-C(12A)-H(12A)	120.0
C(11A)-C(12A)-H(12A)	120.0
N(4A)-C(13A)-C(12A)	132(2)
N(4A)-C(13A)-C(8A)	108(2)
C(12A)-C(13A)-C(8A)	120.0
N(3A)-C(14A)-H(14A)	109.7
N(3A)-C(14A)-H(14B)	109.9
H(14A)-C(14A)-H(14B)	109.1
N(3A)-C(14A)-H(14C)	109.8
H(14A)-C(14A)-H(14C)	109.1
H(14B)-C(14A)-H(14C)	109.1
C(16A)-C(15A)-C(20A)	120.0
C(16A)-C(15A)-C(6A)	120.4(17)
C(20A)-C(15A)-C(6A)	119.5(17)
C(15A)-C(16A)-C(17A)	120.0
C(15A)-C(16A)-H(16A)	120.0
C(17A)-C(16A)-H(16A)	120.0
C(16A)-C(17A)-C(18A)	120.0
C(16A)-C(17A)-H(17A)	120.0
C(18A)-C(17A)-H(17A)	120.0
C(19A)-C(18A)-C(17A)	120.0
C(19A)-C(18A)-Cl(2A)	119.6(17)
C(17A)-C(18A)-Cl(2A)	120.3(17)
C(18A)-C(19A)-C(20A)	120.0
C(18A)-C(19A)-H(19A)	120.0
C(20A)-C(19A)-H(19A)	120.0
C(19A)-C(20A)-C(15A)	120.0
C(19A)-C(20A)-H(20A)	120.0
C(15A)-C(20A)-H(20A)	120.0
N(2A)-C(21A)-C(22A)	121.4(17)
N(2A)-C(21A)-C(26A)	118.3(17)
C(22A)-C(21A)-C(26A)	120.0
C(21A)-C(22A)-C(23A)	120.0

C(21A)-C(22A)-H(22A)	120.0
C(23A)-C(22A)-H(22A)	120.0
C(24A)-C(23A)-C(22A)	120.0
C(24A)-C(23A)-H(23A)	120.0
C(22A)-C(23A)-H(23A)	120.0
O(3A)-C(24A)-C(23A)	126.0(17)
O(3A)-C(24A)-C(25A)	114.0(17)
C(23A)-C(24A)-C(25A)	120.0
C(24A)-C(25A)-C(26A)	120.0
C(24A)-C(25A)-H(25A)	120.0
C(26A)-C(25A)-H(25A)	120.0
C(25A)-C(26A)-C(21A)	120.0
C(25A)-C(26A)-H(26A)	120.0
C(21A)-C(26A)-H(26A)	120.0
O(3A)-C(27A)-C(28A)	115(3)
O(3A)-C(27A)-H(27A)	108.6
C(28A)-C(27A)-H(27A)	108.5
O(3A)-C(27A)-H(27B)	108.6
C(28A)-C(27A)-H(27B)	108.9
H(27A)-C(27A)-H(27B)	107.6
C(27A)-C(28A)-H(28A)	114.2
C(27A)-C(28A)-H(28B)	114.2
H(28A)-C(28A)-H(28B)	104.5
C(27A)-C(28A)-H(28C)	113.7
H(28A)-C(28A)-H(28C)	104.7
H(28B)-C(28A)-H(28C)	104.5
C(24B)-O(3B)-C(27B)	112(2)
C(1B)-N(2B)-C(2B)	127(3)
C(1B)-N(2B)-C(21B)	115(3)
C(2B)-N(2B)-C(21B)	118(2)
C(1B)-N(5B)-C(3B)	115(2)
C(1B)-N(5B)-H(5B)	122.5
C(3B)-N(5B)-H(5B)	122.5
C(5B)-N(3B)-C(14B)	116(3)
C(5B)-N(3B)-C(4B)	106.0(18)
C(14B)-N(3B)-C(4B)	118(2)
C(7B)-N(4B)-C(13B)	115.7(8)
C(7B)-N(4B)-H(4B)	122.1
C(13B)-N(4B)-H(4B)	122.1
N(2B)-C(1B)-N(5B)	94(3)
N(2B)-C(1B)-Se(1B)	144(3)
N(5B)-C(1B)-Se(1B)	119(3)
O(1B)-C(2B)-N(2B)	129(3)
O(1B)-C(2B)-C(3B)	121(3)
N(2B)-C(2B)-C(3B)	110(3)
C(2B)-C(3B)-C(6B)	116(3)
C(2B)-C(3B)-C(4B)	110(3)
C(6B)-C(3B)-C(4B)	113(2)
C(2B)-C(3B)-N(5B)	94(2)
C(6B)-C(3B)-N(5B)	115(2)
C(4B)-C(3B)-N(5B)	107(2)
C(8B)-C(4B)-C(3B)	123(2)
C(8B)-C(4B)-N(3B)	110.9(19)
C(3B)-C(4B)-N(3B)	98(2)
C(8B)-C(4B)-C(7B)	105.1(18)
C(3B)-C(4B)-C(7B)	111.9(18)
N(3B)-C(4B)-C(7B)	107.0(19)
N(3B)-C(5B)-C(6B)	100(2)
N(3B)-C(5B)-H(5B1)	111.7

C(6B)-C(5B)-H(5B1)	111.7
N(3B)-C(5B)-H(5B2)	111.7
C(6B)-C(5B)-H(5B2)	111.6
H(5B1)-C(5B)-H(5B2)	109.4
C(3B)-C(6B)-C(15B)	118(2)
C(3B)-C(6B)-C(5B)	102(2)
C(15B)-C(6B)-C(5B)	118(2)
C(3B)-C(6B)-H(6B)	105.9
C(15B)-C(6B)-H(6B)	106.0
C(5B)-C(6B)-H(6B)	106.0
O(2B)-C(7B)-N(4B)	141(3)
O(2B)-C(7B)-C(4B)	122(2)
N(4B)-C(7B)-C(4B)	97.0(13)
C(9B)-C(8B)-C(13B)	120.0
C(9B)-C(8B)-C(4B)	131.4(17)
C(13B)-C(8B)-C(4B)	108.2(17)
C(8B)-C(9B)-C(10B)	120.0
C(8B)-C(9B)-H(9B)	120.0
C(10B)-C(9B)-H(9B)	120.0
C(11B)-C(10B)-C(9B)	120.0
C(11B)-C(10B)-Cl(1B)	117.5(12)
C(9B)-C(10B)-Cl(1B)	122.4(12)
C(10B)-C(11B)-C(12B)	120.0
C(10B)-C(11B)-H(11B)	120.0
C(12B)-C(11B)-H(11B)	120.0
C(13B)-C(12B)-C(11B)	120.0
C(13B)-C(12B)-H(12B)	120.0
C(11B)-C(12B)-H(12B)	120.0
N(4B)-C(13B)-C(12B)	126.9(13)
N(4B)-C(13B)-C(8B)	113.1(13)
C(12B)-C(13B)-C(8B)	120.0
N(3B)-C(14B)-H(14D)	122.6
N(3B)-C(14B)-H(14E)	123.2
H(14D)-C(14B)-H(14E)	93.0
N(3B)-C(14B)-H(14F)	123.3
H(14D)-C(14B)-H(14F)	93.5
H(14E)-C(14B)-H(14F)	92.9
C(16B)-C(15B)-C(20B)	120.0
C(16B)-C(15B)-C(6B)	122.3(18)
C(20B)-C(15B)-C(6B)	117.7(18)
C(15B)-C(16B)-C(17B)	120.0
C(15B)-C(16B)-H(16B)	120.0
C(17B)-C(16B)-H(16B)	120.0
C(18B)-C(17B)-C(16B)	120.0
C(18B)-C(17B)-H(17B)	120.0
C(16B)-C(17B)-H(17B)	120.0
C(17B)-C(18B)-C(19B)	120.0
C(17B)-C(18B)-Cl(2B)	118.4(16)
C(19B)-C(18B)-Cl(2B)	121.3(16)
C(18B)-C(19B)-C(20B)	120.0
C(18B)-C(19B)-H(19B)	120.0
C(20B)-C(19B)-H(19B)	120.0
C(19B)-C(20B)-C(15B)	120.0
C(19B)-C(20B)-H(20B)	120.0
C(15B)-C(20B)-H(20B)	120.0
C(22B)-C(21B)-C(26B)	120.0
C(22B)-C(21B)-N(2B)	118(2)
C(26B)-C(21B)-N(2B)	122(2)
C(21B)-C(22B)-C(23B)	120.0

C(21B)-C(22B)-H(22B)	120.0
C(23B)-C(22B)-H(22B)	120.0
C(24B)-C(23B)-C(22B)	120.0
C(24B)-C(23B)-H(23B)	120.0
C(22B)-C(23B)-H(23B)	120.0
O(3B)-C(24B)-C(23B)	125(2)
O(3B)-C(24B)-C(25B)	114(2)
C(23B)-C(24B)-C(25B)	120.0
C(24B)-C(25B)-C(26B)	120.0
C(24B)-C(25B)-H(25B)	120.0
C(26B)-C(25B)-H(25B)	120.0
C(25B)-C(26B)-C(21B)	120.0
C(25B)-C(26B)-H(26B)	120.0
C(21B)-C(26B)-H(26B)	120.0
O(3B)-C(27B)-C(28B)	103(2)
O(3B)-C(27B)-H(27C)	111.4
C(28B)-C(27B)-H(27C)	111.4
O(3B)-C(27B)-H(27D)	111.1
C(28B)-C(27B)-H(27D)	111.1
H(27C)-C(27B)-H(27D)	109.1
C(27B)-C(28B)-H(28D)	109.4
C(27B)-C(28B)-H(28E)	109.4
H(28D)-C(28B)-H(28E)	109.5
C(27B)-C(28B)-H(28F)	109.7
H(28D)-C(28B)-H(28F)	109.5
H(28E)-C(28B)-H(28F)	109.5

Symmetry transformations used to generate equivalent atoms:

Table S5. Torsion angles [°] for **5b**.

C(2A)-N(2A)-C(1A)-N(5A)	-8(4)
C(21A)-N(2A)-C(1A)-N(5A)	-175(2)
C(2A)-N(2A)-C(1A)-Se(1A)	177(3)
C(21A)-N(2A)-C(1A)-Se(1A)	11(5)
C(21A)-N(2A)-C(1A)-C(2A)	-167(4)
C(3A)-N(5A)-C(1A)-N(2A)	1(3)
C(3A)-N(5A)-C(1A)-Se(1A)	176(2)
C(3A)-N(5A)-C(1A)-C(2A)	-4(2)
C(1A)-N(2A)-C(2A)-O(1A)	-172(3)
C(21A)-N(2A)-C(2A)-O(1A)	-9(6)
C(1A)-N(2A)-C(2A)-C(3A)	15(4)
C(21A)-N(2A)-C(2A)-C(3A)	177(3)
C(21A)-N(2A)-C(2A)-C(1A)	163(5)
N(2A)-C(2A)-C(3A)-N(5A)	-14(4)
O(1A)-C(2A)-C(3A)-N(5A)	174(3)
C(1A)-C(2A)-C(3A)-N(5A)	-4(2)
N(2A)-C(2A)-C(3A)-C(6A)	-137(4)
O(1A)-C(2A)-C(3A)-C(6A)	51(4)
C(1A)-C(2A)-C(3A)-C(6A)	-127(3)
N(2A)-C(2A)-C(3A)-C(4A)	103(4)
O(1A)-C(2A)-C(3A)-C(4A)	-69(5)
C(1A)-C(2A)-C(3A)-C(4A)	113(3)
C(1A)-N(5A)-C(3A)-C(2A)	6(3)
C(1A)-N(5A)-C(3A)-C(6A)	124(3)
C(1A)-N(5A)-C(3A)-C(4A)	-113(2)
C(5A)-N(3A)-C(4A)-C(3A)	35(3)
C(14A)-N(3A)-C(4A)-C(3A)	166(3)
C(5A)-N(3A)-C(4A)-C(8A)	165(2)
C(14A)-N(3A)-C(4A)-C(8A)	-64(3)
C(5A)-N(3A)-C(4A)-C(7A)	-82(3)
C(14A)-N(3A)-C(4A)-C(7A)	48(4)
C(2A)-C(3A)-C(4A)-N(3A)	102(3)
N(5A)-C(3A)-C(4A)-N(3A)	-150(2)
C(6A)-C(3A)-C(4A)-N(3A)	-21(3)
C(2A)-C(3A)-C(4A)-C(8A)	-22(4)
N(5A)-C(3A)-C(4A)-C(8A)	87(3)
C(6A)-C(3A)-C(4A)-C(8A)	-145(2)
C(2A)-C(3A)-C(4A)-C(7A)	-140(3)
N(5A)-C(3A)-C(4A)-C(7A)	-32(3)
C(6A)-C(3A)-C(4A)-C(7A)	97(3)
C(4A)-N(3A)-C(5A)-C(6A)	-33(3)
C(14A)-N(3A)-C(5A)-C(6A)	-170(2)
C(2A)-C(3A)-C(6A)-C(15A)	103(3)
N(5A)-C(3A)-C(6A)-C(15A)	-8(4)
C(4A)-C(3A)-C(6A)-C(15A)	-133(2)
C(2A)-C(3A)-C(6A)-C(5A)	-122(3)
N(5A)-C(3A)-C(6A)-C(5A)	127(2)
C(4A)-C(3A)-C(6A)-C(5A)	2(3)
N(3A)-C(5A)-C(6A)-C(3A)	19(3)
N(3A)-C(5A)-C(6A)-C(15A)	153(2)
C(13A)-N(4A)-C(7A)-O(2A)	-175(3)
C(13A)-N(4A)-C(7A)-C(4A)	2(3)
N(3A)-C(4A)-C(7A)-O(2A)	56(4)
C(3A)-C(4A)-C(7A)-O(2A)	-59(4)
C(8A)-C(4A)-C(7A)-O(2A)	173(3)
N(3A)-C(4A)-C(7A)-N(4A)	-122(3)
C(3A)-C(4A)-C(7A)-N(4A)	123(2)

C(8A)-C(4A)-C(7A)-N(4A)	-5(3)
N(3A)-C(4A)-C(8A)-C(9A)	-60(3)
C(3A)-C(4A)-C(8A)-C(9A)	61(3)
C(7A)-C(4A)-C(8A)-C(9A)	-177.4(16)
N(3A)-C(4A)-C(8A)-C(13A)	124.0(17)
C(3A)-C(4A)-C(8A)-C(13A)	-115(2)
C(7A)-C(4A)-C(8A)-C(13A)	7(2)
C(13A)-C(8A)-C(9A)-C(10A)	0.0
C(4A)-C(8A)-C(9A)-C(10A)	-176(2)
C(8A)-C(9A)-C(10A)-C(11A)	0.0
C(8A)-C(9A)-C(10A)-Cl(1A)	177.1(13)
C(9A)-C(10A)-C(11A)-C(12A)	0.0
Cl(1A)-C(10A)-C(11A)-C(12A)	-177.1(13)
C(10A)-C(11A)-C(12A)-C(13A)	0.0
C(7A)-N(4A)-C(13A)-C(12A)	179.6(19)
C(7A)-N(4A)-C(13A)-C(8A)	2(3)
C(11A)-C(12A)-C(13A)-N(4A)	-177(3)
C(11A)-C(12A)-C(13A)-C(8A)	0.0
C(9A)-C(8A)-C(13A)-N(4A)	178(2)
C(4A)-C(8A)-C(13A)-N(4A)	-5.7(19)
C(9A)-C(8A)-C(13A)-C(12A)	0.0
C(4A)-C(8A)-C(13A)-C(12A)	176.6(15)
C(3A)-C(6A)-C(15A)-C(16A)	76(3)
C(5A)-C(6A)-C(15A)-C(16A)	-53(3)
C(3A)-C(6A)-C(15A)-C(20A)	-100(2)
C(5A)-C(6A)-C(15A)-C(20A)	131(2)
C(20A)-C(15A)-C(16A)-C(17A)	0.0
C(6A)-C(15A)-C(16A)-C(17A)	-177(2)
C(15A)-C(16A)-C(17A)-C(18A)	0.0
C(16A)-C(17A)-C(18A)-C(19A)	0.0
C(16A)-C(17A)-C(18A)-Cl(2A)	-176(2)
C(17A)-C(18A)-C(19A)-C(20A)	0.0
Cl(2A)-C(18A)-C(19A)-C(20A)	177(2)
C(18A)-C(19A)-C(20A)-C(15A)	0.0
C(16A)-C(15A)-C(20A)-C(19A)	0.0
C(6A)-C(15A)-C(20A)-C(19A)	177(2)
C(2A)-N(2A)-C(21A)-C(22A)	-68(4)
C(1A)-N(2A)-C(21A)-C(22A)	93(3)
C(2A)-N(2A)-C(21A)-C(26A)	106(4)
C(1A)-N(2A)-C(21A)-C(26A)	-94(3)
N(2A)-C(21A)-C(22A)-C(23A)	174(2)
C(26A)-C(21A)-C(22A)-C(23A)	0.0
C(21A)-C(22A)-C(23A)-C(24A)	0.0
C(27A)-O(3A)-C(24A)-C(23A)	-4(3)
C(27A)-O(3A)-C(24A)-C(25A)	175(2)
C(22A)-C(23A)-C(24A)-O(3A)	179.2(18)
C(22A)-C(23A)-C(24A)-C(25A)	0.0
O(3A)-C(24A)-C(25A)-C(26A)	-179.3(16)
C(23A)-C(24A)-C(25A)-C(26A)	0.0
C(24A)-C(25A)-C(26A)-C(21A)	0.0
N(2A)-C(21A)-C(26A)-C(25A)	-173.9(19)
C(22A)-C(21A)-C(26A)-C(25A)	0.0
C(24A)-O(3A)-C(27A)-C(28A)	-171(3)
C(2B)-N(2B)-C(1B)-N(5B)	9(4)
C(21B)-N(2B)-C(1B)-N(5B)	-176(2)
C(2B)-N(2B)-C(1B)-Se(1B)	165(4)
C(21B)-N(2B)-C(1B)-Se(1B)	-20(7)
C(3B)-N(5B)-C(1B)-N(2B)	-6(3)
C(3B)-N(5B)-C(1B)-Se(1B)	-170.3(19)

C(1B)-N(2B)-C(2B)-O(1B)	171(3)
C(21B)-N(2B)-C(2B)-O(1B)	-4(4)
C(1B)-N(2B)-C(2B)-C(3B)	-8(4)
C(21B)-N(2B)-C(2B)-C(3B)	176(2)
O(1B)-C(2B)-C(3B)-C(6B)	-56(4)
N(2B)-C(2B)-C(3B)-C(6B)	123(3)
O(1B)-C(2B)-C(3B)-C(4B)	73(3)
N(2B)-C(2B)-C(3B)-C(4B)	-108(3)
O(1B)-C(2B)-C(3B)-N(5B)	-177(3)
N(2B)-C(2B)-C(3B)-N(5B)	2(3)
C(1B)-N(5B)-C(3B)-C(2B)	2(3)
C(1B)-N(5B)-C(3B)-C(6B)	-119(3)
C(1B)-N(5B)-C(3B)-C(4B)	115(3)
C(2B)-C(3B)-C(4B)-C(8B)	17(4)
C(6B)-C(3B)-C(4B)-C(8B)	147(3)
N(5B)-C(3B)-C(4B)-C(8B)	-84(3)
C(2B)-C(3B)-C(4B)-N(3B)	-104(3)
C(6B)-C(3B)-C(4B)-N(3B)	26(3)
N(5B)-C(3B)-C(4B)-N(3B)	154.3(19)
C(2B)-C(3B)-C(4B)-C(7B)	144(3)
C(6B)-C(3B)-C(4B)-C(7B)	-86(3)
N(5B)-C(3B)-C(4B)-C(7B)	42(3)
C(5B)-N(3B)-C(4B)-C(8B)	-174(2)
C(14B)-N(3B)-C(4B)-C(8B)	54(4)
C(5B)-N(3B)-C(4B)-C(3B)	-44(2)
C(14B)-N(3B)-C(4B)-C(3B)	-176(3)
C(5B)-N(3B)-C(4B)-C(7B)	72(2)
C(14B)-N(3B)-C(4B)-C(7B)	-60(4)
C(14B)-N(3B)-C(5B)-C(6B)	178(2)
C(4B)-N(3B)-C(5B)-C(6B)	44(2)
C(2B)-C(3B)-C(6B)-C(15B)	-103(3)
C(4B)-C(3B)-C(6B)-C(15B)	130(2)
N(5B)-C(3B)-C(6B)-C(15B)	6(4)
C(2B)-C(3B)-C(6B)-C(5B)	126(3)
C(4B)-C(3B)-C(6B)-C(5B)	-1(3)
N(5B)-C(3B)-C(6B)-C(5B)	-125(2)
N(3B)-C(5B)-C(6B)-C(3B)	-26(3)
N(3B)-C(5B)-C(6B)-C(15B)	-157(2)
C(13B)-N(4B)-C(7B)-O(2B)	175(5)
C(13B)-N(4B)-C(7B)-C(4B)	-5(3)
C(8B)-C(4B)-C(7B)-O(2B)	-172(3)
C(3B)-C(4B)-C(7B)-O(2B)	52(4)
N(3B)-C(4B)-C(7B)-O(2B)	-54(4)
C(8B)-C(4B)-C(7B)-N(4B)	8(3)
C(3B)-C(4B)-C(7B)-N(4B)	-128(3)
N(3B)-C(4B)-C(7B)-N(4B)	126(2)
C(3B)-C(4B)-C(8B)-C(9B)	-51(3)
N(3B)-C(4B)-C(8B)-C(9B)	64(3)
C(7B)-C(4B)-C(8B)-C(9B)	178.8(16)
C(3B)-C(4B)-C(8B)-C(13B)	121(2)
N(3B)-C(4B)-C(8B)-C(13B)	-124.2(18)
C(7B)-C(4B)-C(8B)-C(13B)	-9(2)
C(13B)-C(8B)-C(9B)-C(10B)	0.0
C(4B)-C(8B)-C(9B)-C(10B)	172(3)
C(8B)-C(9B)-C(10B)-C(11B)	0.0
C(8B)-C(9B)-C(10B)-Cl(1B)	-178.8(16)
C(9B)-C(10B)-C(11B)-C(12B)	0.0
Cl(1B)-C(10B)-C(11B)-C(12B)	178.9(15)
C(10B)-C(11B)-C(12B)-C(13B)	0.0

C(7B)-N(4B)-C(13B)-C(12B)	180(2)
C(7B)-N(4B)-C(13B)-C(8B)	0(3)
C(11B)-C(12B)-C(13B)-N(4B)	-180(3)
C(11B)-C(12B)-C(13B)-C(8B)	0.0
C(9B)-C(8B)-C(13B)-N(4B)	180(2)
C(4B)-C(8B)-C(13B)-N(4B)	6(2)
C(9B)-C(8B)-C(13B)-C(12B)	0.0
C(4B)-C(8B)-C(13B)-C(12B)	-173(2)
C(3B)-C(6B)-C(15B)-C(16B)	-77(3)
C(5B)-C(6B)-C(15B)-C(16B)	46(3)
C(3B)-C(6B)-C(15B)-C(20B)	102(3)
C(5B)-C(6B)-C(15B)-C(20B)	-135(2)
C(20B)-C(15B)-C(16B)-C(17B)	0.0
C(6B)-C(15B)-C(16B)-C(17B)	179(2)
C(15B)-C(16B)-C(17B)-C(18B)	0.0
C(16B)-C(17B)-C(18B)-C(19B)	0.0
C(16B)-C(17B)-C(18B)-Cl(2B)	174(2)
C(17B)-C(18B)-C(19B)-C(20B)	0.0
Cl(2B)-C(18B)-C(19B)-C(20B)	-173(2)
C(18B)-C(19B)-C(20B)-C(15B)	0.0
C(16B)-C(15B)-C(20B)-C(19B)	0.0
C(6B)-C(15B)-C(20B)-C(19B)	-179(2)
C(1B)-N(2B)-C(21B)-C(22B)	-85(3)
C(2B)-N(2B)-C(21B)-C(22B)	91(3)
C(1B)-N(2B)-C(21B)-C(26B)	93(3)
C(2B)-N(2B)-C(21B)-C(26B)	-91(3)
C(26B)-C(21B)-C(22B)-C(23B)	0.0
N(2B)-C(21B)-C(22B)-C(23B)	179(2)
C(21B)-C(22B)-C(23B)-C(24B)	0.0
C(27B)-O(3B)-C(24B)-C(23B)	46(3)
C(27B)-O(3B)-C(24B)-C(25B)	-143(3)
C(22B)-C(23B)-C(24B)-O(3B)	171(2)
C(22B)-C(23B)-C(24B)-C(25B)	0.0
O(3B)-C(24B)-C(25B)-C(26B)	-172(2)
C(23B)-C(24B)-C(25B)-C(26B)	0.0
C(24B)-C(25B)-C(26B)-C(21B)	0.0
C(22B)-C(21B)-C(26B)-C(25B)	0.0
N(2B)-C(21B)-C(26B)-C(25B)	-179(2)
C(24B)-O(3B)-C(27B)-C(28B)	-170(3)

Symmetry transformations used to generate equivalent atoms:

Table S6. Hydrogen bonds for **5b** [E and °].

D-H...A	d(D-H)	d(H...A)	d(D...A)	<(DHA)
N(4A)-H(4A)...O(1A)#1	0.86	2.13	2.93(3)	154.7
C(6A)-H(6A)...O(1A)	0.98	2.32	2.84(3)	112.5
C(9A)-H(9A)...O(1A)	0.93	2.49	3.06(2)	119.9
C(23A)-H(23A)...Se(1A)#2	0.93	3.06	3.970(14)	167.5
N(4B)-H(4B)...O(1B)#1	0.86	2.11	2.85(2)	144.5
C(5B)-H(5B1)...O(2B)	0.97	2.36	2.98(4)	121.5
C(5B)-H(5B2)...Cl(2A)#3	0.97	2.89	3.54(3)	125.2
C(9B)-H(9B)...O(1B)	0.93	2.25	2.87(2)	123.5
C(23B)-H(23B)...Se(1B)#2	0.93	3.09	3.990(16)	164.0

Symmetry transformations used to generate equivalent atoms:
#1 x-1,y,z #2 x+1,y,z #3 -x+1,-y+2,-z+1
